# Synthesis of Benzofuropyridines and Dibenzofurans by a Metalation/Negishi Cross-Coupling/S_N_Ar Reaction Sequence

**DOI:** 10.1021/acs.joc.2c02111

**Published:** 2022-12-09

**Authors:** Guy J. Clarkson, Stefan Roesner

**Affiliations:** Department of Chemistry, University of Warwick, Gibbet Hill Road, Coventry CV4 7AL, United Kingdom

## Abstract

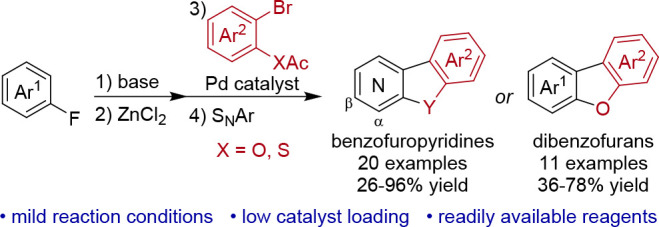

An efficient methodology for the synthesis of benzofuropyridines and dibenzofurans from fluoropyridines or fluoroarenes and 2-bromophenyl acetates is reported. This streamlined one-pot procedure consists of a four-step directed *ortho*-lithiation, zincation, Negishi cross-coupling, and intramolecular nucleophilic aromatic substitution, allowing for the facile assembly of a diverse set of fused benzofuro heterocycles.

Nitrogen heterocycles are among the most significant structural motifs of pharmaceuticals with more than half of all FDA approved small-molecule drugs containing at least one N-heterocycle.^1^ Among them, benzofuropyridines are tricyclic compounds containing an annulated pyridine, furan, and benzene ring. However, compared to the synthesis and biological evaluation of dibenzofurans,^2,3^ benzofuropyridines have been far less explored. This is surprising as this class of compounds shows diverse biological activity and interesting properties for potential applications in materials science. Examples include elbfluorene (**I**) and its derivatives that possess high activity as cyclin-dependent kinase (CDK) inhibitors^4^ and benzofuro[2,3-*c*]pyridine **II** with potential applications as an MDR modulator (Figure 1).^5^ In addition to their diverse biological activity, benzofuro- and benzothienopyridine derivatives possess interesting fluorescence properties suitable to be applied as green or blue OLED emitters.^6^ Moreover, dyes with a benzothieno[2,3-*c*]pyridine (**III**) anchoring group have received considerable attention in the development of dye-sensitized solar cells.^7^

**Figure 1:**
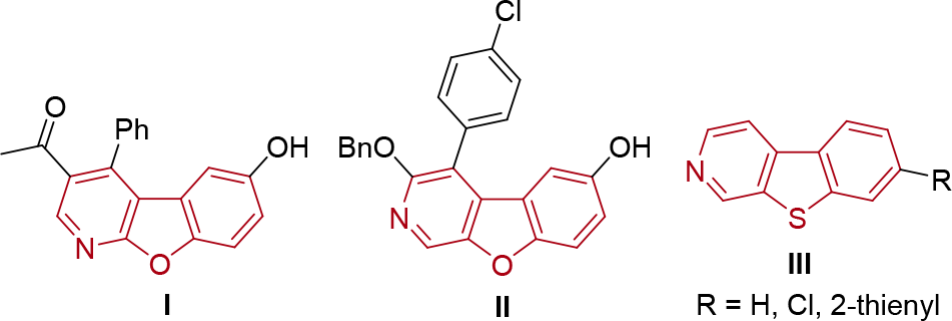
Benzofuropyridines and benzothienopyridines with interesting biological properties and applications in materials science.

Several synthetic strategies toward the preparation of benzofuropyridines have been reported. Intramolecular C–C bond formation in diaryl ethers^8,9^ and C–O bond formation in 2-biaryl phenols by intramolecular nucleophilic aromatic substitution (S_N_Ar)^8,10^ are the most common strategies. The former approach was successfully applied by Yue and Li, who synthesized all four benzofuropyridine regioisomers.^8^ Here, a palladium catalyzed Stille coupling using toxic organotin reagents was a key step. Liu et al. reported a more environmental benign strategy generating biaryl phenols from dihalopyridines and 2-hydroxyphenylboronic acids via regioselective Suzuki cross-coupling followed by copper catalyzed intramolecular cyclization.^9b^ Alternative strategies for the synthesis of benzofuropyridines include the construction of the pyridine ring from benzofuran derivatives^11^ or cascade reactions that generate two annulated rings in the same synthetic operation.^12^ However, these approaches often rely on elaborated substrates. Consequently, the development of a concise and general synthesis of benzofuropyridines and its derivatives from readily available starting materials is still of considerable interest.

Recently, we reported an efficient synthesis of tricyclic carbolines employing a four-step *ortho*-lithiation/zincation/Negishi cross-coupling/S_N_Ar reaction sequence (Scheme 1a).^13^ In this methodology a fluorine substituent serves both as directing group for the metalation^14^ as well as facile leaving group in the intramolecular cyclization. As a continuation of our efforts to develop new synthetic procedures for the construction of heterocyclic frameworks, we were curious if we could extend this procedure by using phenols and thiophenols as a route to tricyclic systems (Scheme 1b). Herein, we describe the first one-pot synthesis of benzofuro[2,3-*b*]- and benzofuro[2,3-*c*]pyridines **5** via a telescoped metalation/cross-coupling/S_N_Ar reaction sequence from commercially available fluoropyridines and readily accessible 2-bromophenyl acetates.^15^ This strategy was expanded to benzothieno[2,3-*b*]pyridines. In addition, directed lithiation of fluoroarenes provided facile access to a range of functionalized dibenzofurans **6**.

**Scheme 1:**
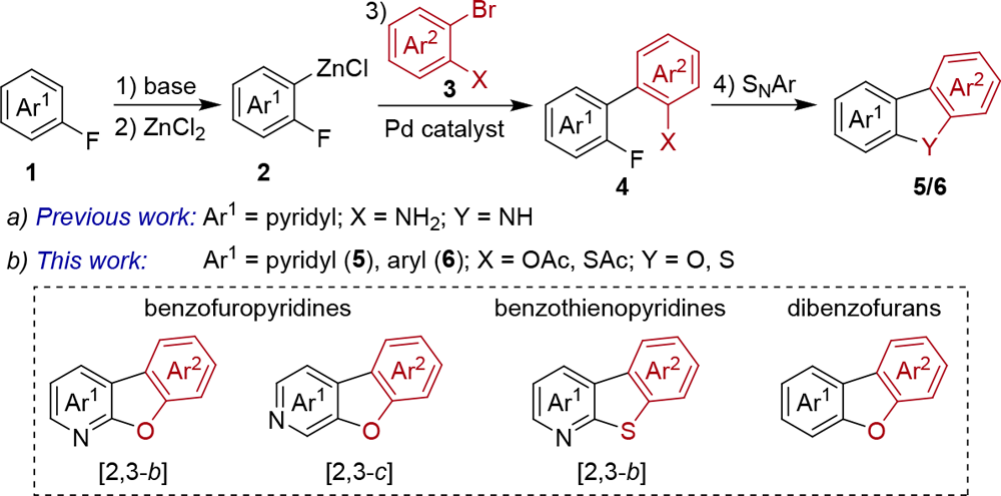
(a) Previously Reported Synthesis of Carbolines by a Lithiation/Zincation/Negishi Cross-Coupling/S_N_Ar Reaction Sequence and (b) Extension of This Methodology to Benzofuro- and Benzothienopyridines as Well as Dibenzofurans

We initiated our studies by optimizing the reaction conditions for the one-pot Negishi cross-coupling/intramolecular S_N_Ar reaction sequence (Table 1). Using our previously reported conditions for the formation of organozinc species **2**,^13^ 2-fluoropyridine (**1a**), and 2-bromophenyl acetate (**3a**) were converted to benzofuropyridine **5a** in 96% yield for the four-step sequence using 2 mol % of an XPhos-based palladium precatalyst^16^ with additional 2 mol % XPhos ligand^17^ and 2.0 equiv of KO*t*Bu after heating at 70 °C overnight (entry 1). Reducing either the catalyst loading to 1 mol % or omitting the additional XPhos ligand decreased the yield of **5a** (entry 2–3). Protection of the phenolic oxygen was essential as no product could be detected with 2-bromophenol, even in the presence of excess base (entry 4). We hypothesize that the free phenol is able to protonate **2**, while the corresponding phenolate does not participate in the cross-coupling reaction. This is in stark contrast to 2-bromoanilines, which do not require protection under similar reaction conditions.^13^ Other phenolic protecting groups gave **5a** either in lower yields or afforded the corresponding biaryls **4**.^18^ Employing alternative palladium sources in the presence of 2 mol % XPhos ligand highlights the superior activity of the palladium precatalyst under the applied cross-coupling conditions (entries 5–7). It is worth noting that both the palladium catalyst and the XPhos ligand were essential for the reaction to take place (entries 8–9). In the absence of additional base, the cross-coupling was complete in 20 min as demonstrated by the isolation of biaryl acetate **4a** in 84% yield (entry 10). Reducing either the amount of base or the reaction time led to incomplete conversion of the intermediate biaryl **4a** (entries 11–13). Finally, while screening other bases to facilitate the deprotection of the acyl group and to promote the S_N_Ar reaction, we found NaHMDS as valuable alternative to KO*t*Bu (entry 14), whereas Cs_2_CO_3_ did not fully deprotect the phenolic alcohol under the applied reaction conditions (entry 15).

**Table 1:**
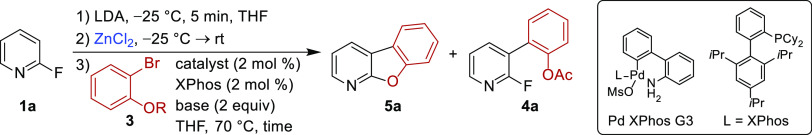
Optimization of the Reaction Conditions^a^

entry	R	catalyst	base	time	yield **5a**^b^(%)	yield **4a**^b^(%)
1	Ac	Pd XPhos G3	KO*t*Bu	o/n	96^c^	−
2^d^	Ac	Pd XPhos G3	KO*t*Bu	o/n	81	−
3^e^	Ac	Pd XPhos G3	KO*t*Bu	o/n	74^c^	−
4^f^	H	Pd XPhos G3	KO*t*Bu	o/n	0	−
5	Ac	Pd(PPh_3_)_4_	KO*t*Bu	o/n	50	−
6	Ac	Pd(OAc)_2_	KO*t*Bu	o/n	70	−
7^d^	Ac	[PdCl(C_3_H_5_)]_2_	KO*t*Bu	o/n	84	−
8	Ac	−	KO*t*Bu	o/n	0	−
9^e^	Ac	Pd(OAc)_2_	KO*t*Bu	o/n	11	−
10	Ac	Pd XPhos G3	−	20 min	−	84^c^
11	Ac	Pd XPhos G3	−	o/n	51	31
12^g^	Ac	Pd XPhos G3	KO*t*Bu	o/n	52	16
13	Ac	Pd XPhos G3	KO*t*Bu	20 min	11	28
14	Ac	Pd XPhos G3	NaHMDS	o/n	86	−
15	Ac	Pd XPhos G3	Cs_2_CO_3_	o/n	22	30

a 0.5 mmol scale; reaction conditions: (1) **1a** (1.2 equiv), LDA (1.3 equiv), THF (0.25 M), 25 °C, 5 min; (2) ZnCl_2_ (1.3 equiv), then −25 °C to rt; (3) 2-bromophenol derivative **3** (1.0 equiv), catalyst (2.0 mol %), and XPhos (2.0 mol %) in THF (0.5 M), base (2.0 equiv), 70 °C.

b Determined by ^1^H NMR spectroscopy using 1,3,5-trimethoxybenzene as internal standard.

c Isolated yield after column chromatography.

d 1 mol % catalyst loading.

e No additional XPhos ligand.

f 2.3 equiv of LDA used.

g 1.0 equiv of base.

The results from the optimization studies reveal the reaction order in the formation of **5a** (Scheme 2). After Negishi cross-coupling between organozinc intermediate **2a** and 2-bromophenyl acetate (**3a**) to form biaryl acetate **4a**, deprotection of the acyl group occurs to provide probably biaryl phenol **7a**, which was not observed in the optimization studies, indicating that it must undergo rapid intramolecular cyclization under the basic conditions to generate the C–O bond and tricyclic **5a** via S_N_Ar.

**Scheme 2:**
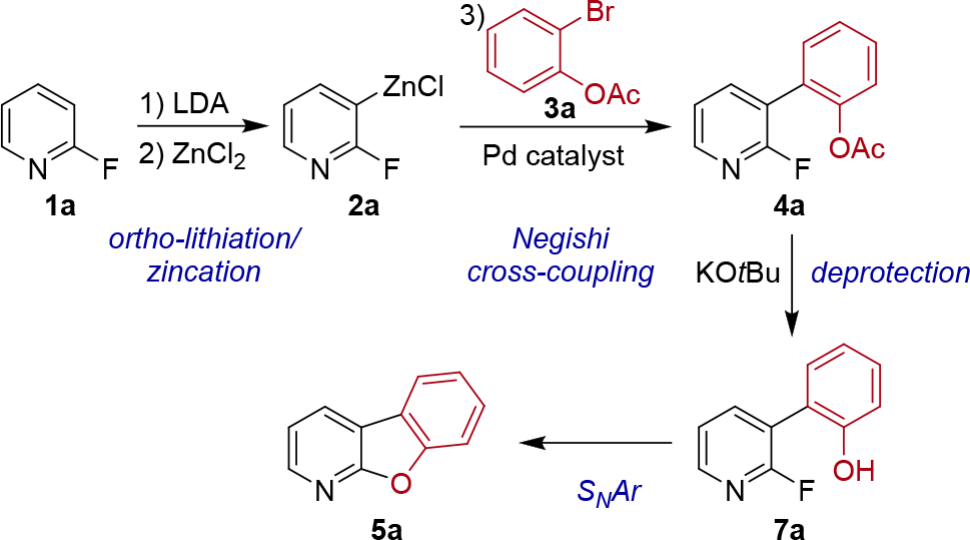
Reaction Sequence and Intermediates in the Formation of Benzofuropyridine **5a**

With optimized reaction conditions in hand, we next examined the generality of the methodology for a variety of fluoropyridines (Table 2). With 2-fluoro-5-methylpyridine, 2,6- and 2,4-difluoropyridine, the corresponding benzofuro[2,3-*b*]pyridines **5b**–**d** were obtained as single regioisomers in 63–88% yield. However, for 3-fluoropyridine, 2,3- and 2,5-difluoropyridine no conversion to the corresponding benzofuro[2,3-*c*]pyridines was observed providing biaryl phenols instead. In other words, nucleophilic substitution of the less activated fluorine in 3-position of the pyridyl ring was not achieved. To promote the intramolecular S_N_Ar reaction, we conducted a solvent exchange from THF to DMF after the cross-coupling step and added additional base (Cs_2_CO_3_).^10b^ After heating to 100 °C for 2 h, **5e**–**g** could be isolated in 39–88% yield. For **5g** we achieved better results conducting the nucleophilic substitution from the purified biaryl phenol.

**Table 2:**
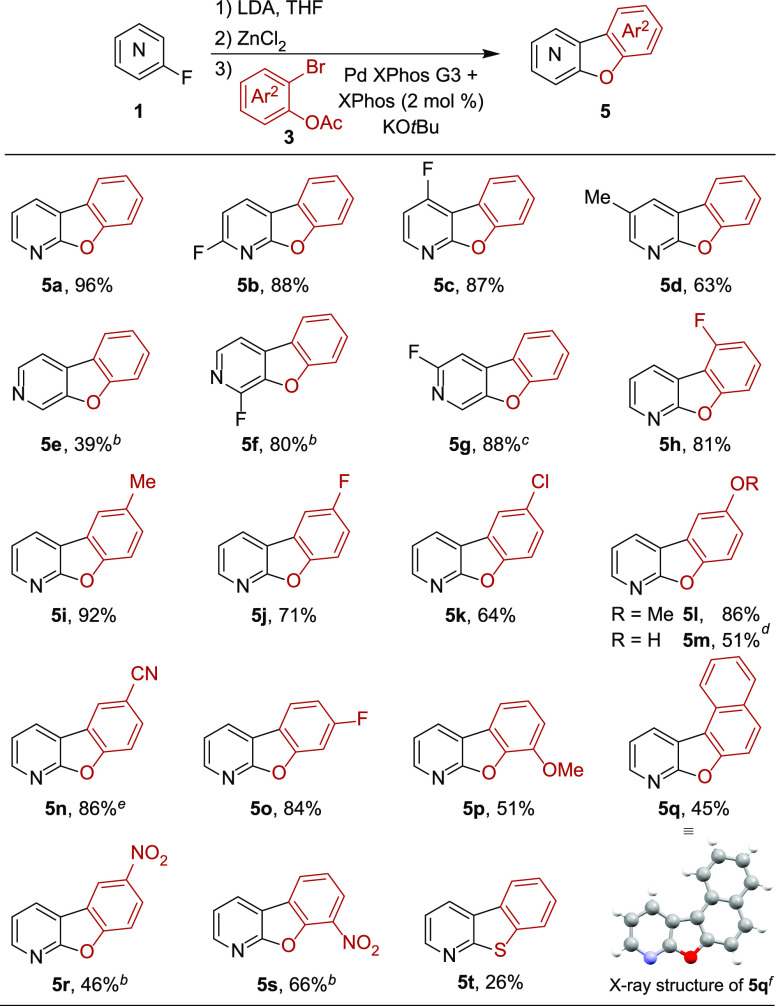
Scope of Benzofuropyridines^a^

a 0.5 mmol scale; reaction conditions: (1) **1** (1.2 equiv), LDA (1.3 equiv), THF (0.25 M), −25 °C, 5 min; (2) ZnCl_2_ (1.3 equiv), then −25 °C to rt; (3) **3** (1.0 equiv), Pd XPhos G3 (2.0 mol %), and XPhos (2.0 mol %) in THF (0.5 M), then KO*t*Bu (2.0 equiv), 70 °C, o/n.

b Reaction conditions as shown for *a*, no KO*t*Bu, 70 °C, 20 min, then solvent exchange to DMF (0.1 M), Cs_2_CO_3_ (5 equiv), 100 °C, 2 h.

c Reaction conditions as shown for *a*, isolation of **7**, then DMF (0.1 M), Cs_2_CO_3_ (5 equiv), 100 °C, 2 h.

d From the diacetate of bromohydroquinone.

e 2.5 mmol scale.

f X-ray structure of **5q** shown with 50% probability ellipsoids.

We next explored the substrate scope of a variety of 2-bromophenyl acetates **3** (Table 2). Substitution in all positions of **3** with a number of electron-withdrawing and -donating groups was tolerated providing dibenzofuro[2,3-*b*]pyridines **5h**–**q** in 45–92% yield. The structure of tetracyclic **5q** was verified by single crystal X-ray diffraction.^19^ Additionally, we demonstrated the scalability of the procedure by the synthesis of **5n**, which could be obtained in excellent yield after simple recrystallization. Though, for substrates containing a strongly electron-withdrawing nitro group intramolecular nucleophilic substitution was not successful. Under standard conditions the intermediate biaryl phenols were isolated. Here, utilizing the reaction conditions for dibenzofuro[2,3-*c*]pyridines (solvent exchange to DMF, then Cs_2_CO_3_), **5r** and **5s** could be obtained in 46% and 66% yield, respectively. The procedure also furnished benzothienopyridine **5t**. It is not clear if the low isolated yield is due to poisoning of the palladium catalyst or too rapid deprotection of the acetyl group, which would make 2-bromothiophenol unreactive as coupling partner as previously shown for the related unprotected phenol.

Next, we extended our methodology to the synthesis of dibenzofurans. In contrast to fluoropyridines, the directed *ortho*-metalation of fluoroarenes requires a stronger base than LDA. Here, the use of the superbasic *n*BuLi/KO*t*Bu system has been well established.^20^ Adapting our standard procedure using superbasic metalation conditions, the corresponding biaryl phenols were obtained, this means that the fluoroarenes were unreactive toward intramolecular S_N_Ar. Again, solvent exchange to DMF and addition of Cs_2_CO_3_ followed by heating (120 °C, overnight) provided a solution converting fluoroarenes **1** and 2-bromophenyl acetates **3** to dibenzofurans **6** in a one-pot four-step procedure.

With fluorobenzene and 3,4-dimethylfluorobenzene, the corresponding dibenzofurans **6a** and **6b** were obtained in 78% and 50%, respectively (Table 3). In the case of fluoroanisoles, fluorine proved to be a stronger directing group under superbasic metalation conditions as demonstrated by the formation of **6c** and **6d**.^20b^ The structure of **6c** was confirmed by single crystal X-ray diffraction.^19^ Furthermore, 1,4- and 1,2-difluorobenzene provided the corresponding fluoro-substituted dibenzofurans **6e** and **6f** in reasonable yield. Finally, the 2-bromophenyl acetates **3** coupling partner was varied delivering **6d′–e′** and **6g**–**i** in 39–65% yield. Here, dibenzofurans **6e/e′** and **6i** have been reported as precursors for the synthesis of host materials for blue phosphorescent OLEDs.^21^ With dibenzofurans **6d** and **6d′** as well as **6e** and **6e′** being pairs of identical compounds, our methodology allows flexibility in terms of the choice of starting materials **1** and **3** (Scheme 3). Hereby, slightly higher yields were achieved utilizing fluorobenzene (**1b**) and substituted 2-bromophenyl acetates **3** via path b. Unfortunately, the synthesis of dibenzothiophenes was not successful under these reaction conditions generating 2-bromothiophenol as main product from the corresponding acetate **3**.^18^

**Table 3:**
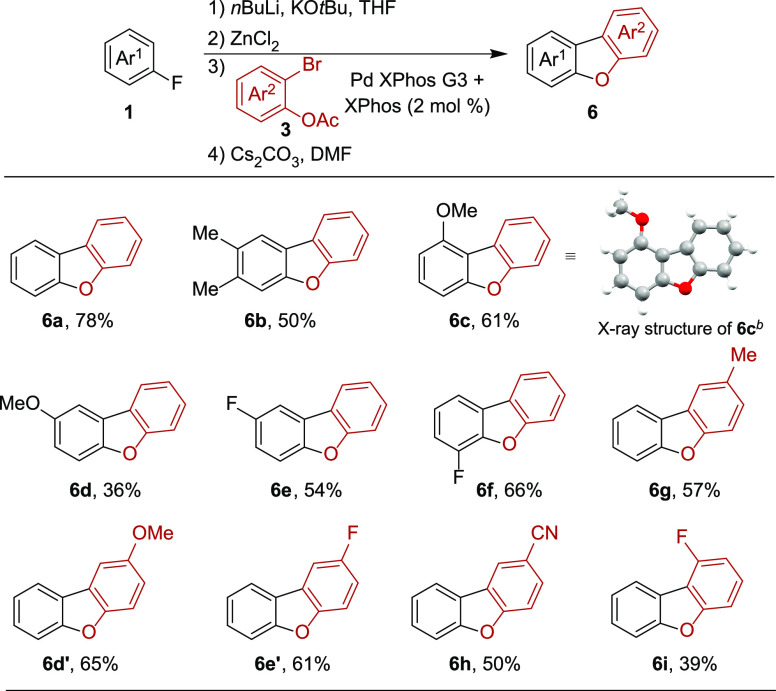
Scope of Dibenzofurans^a^

a 0.5 mmol scale; reaction conditions: (1) **1** (1.2 equiv), KO*t*Bu (1.3 equiv), LDA (1.3 equiv), THF (0.25 M), −78 °C, 5 min; (2) ZnCl_2_ (1.3 equiv), then −78 °C to rt; (3) **3** (1.0 equiv), Pd XPhos G3 (2.0 mol %), and XPhos (2.0 mol %) in THF (0.5 M), then, 70 °C, 20 min; and (4) solvent exchange to DMF (0.1 M), Cs_2_CO_3_ (5 equiv), 120 °C, o/n.

b X-ray structure of **6c** shown with 50% probability ellipsoids.

**Scheme 3:**
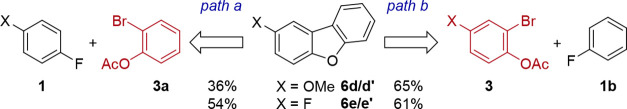
Convergent Synthesis of Dibenzofurans **6d/d′** and **6e/e′**

In conclusion, we have developed an efficient one-pot procedure for the facile assembly of fused benzofuro heterocycles. Fluoropyridines or fluoroarenes were subjected to a directed *ortho*-lithiation followed by zincation and Negishi cross-coupling with 2-bromophenyl acetates. In situ deprotection of the acyl group and subsequent intramolecular S_N_Ar facilitates the formation of benzofuropyridines and dibenzofurans. This methodology takes advantage of readily available starting materials, mild reaction conditions and low catalyst loading to provide a diverse set of benzofuropyridines and dibenzofurans.

## Experimental Section

### Typical Procedure for the Synthesis of 2-Bromophenyl Acetates **3**

#### 2-Bromophenyl Acetate (**3a**)

Following a procedure by Kónya,^15^ to a solution of 2-bromophenol (4.46 g, 40.0 mmol, 1.0 equiv) in anhydrous CH_2_Cl_2_ (40 mL) were added acetyl chloride (1.56 mL, 22.0 mmol, 1.1 equiv) and Et_3_N (3.04 mL, 22.0 mmol, 1.1 equiv). The reaction mixture was stirred for 1 h at room temperature. Then, the organic layer was washed with saturated aqueous NaHCO_3_ solution (20 mL) and the aqueous layer was extracted with CH_2_Cl_2_ (2 × 20 mL). The combined organic layers were dried over anhydrous MgSO_4_, filtered, and the solvent was removed under reduced pressure to give **3a** as a pale-yellow oil (4.26 g, 19.8 mmol, 99% yield), which was used without further purification. ^1^H NMR (400 MHz, CDCl_3_) δ_H_ 7.61 (dd, *J* = 8.3, 0.9 Hz, 1H), 7.33 (t, *J* = 7.7 Hz, 1H), 7.16–7.09 (m, 2H), 2.36 (s, 3H); ^13^C{^1^H} NMR (101 MHz, CDCl_3_) δ_C_ 168.7, 148.4, 133.5, 128.6, 127.5, 123.9, 116.4, 20.9. The analytical data match those reported in the literature.^23^

### Typical Procedure for the Synthesis of Benzofuro[2,3-*b*]pyridines **5**

#### Benzofuro[2,3-*b*]pyridine (**5a**)

To a solution of 2-fluoropyridine (52 μL, 0.60 mmol, 1.2 equiv) in anhydrous THF (2.4 mL), was added a solution of LDA (2.0 M, 325 μL, 0.65 mmol, 1.3 equiv) dropwise at −25 °C. The reaction mixture was stirred at −25 °C for 5 min, followed by the addition of ZnCl_2_ solution in THF (0.7 M, 930 μL, 0.65 mmol, 1.3 equiv). The cooling bath was removed, and the reaction mixture was allowed to warm to room temperature, after which a solution of **3a** (108 mg, 0.50 mmol, 1.0 equiv), precatalyst Pd XPhos G3 (8.5 mg, 10 μmol, 2.0 mol %), and XPhos (4.8 mg, 10 μmol, 2.0 mol %) in THF (1.0 mL) followed by a solution of KO*t*Bu in THF (1.6 M, 625 μL, 1.00 mmol, 2.0 equiv) was added. The reaction mixture was stirred at 70 °C in a heated oil bath overnight. Saturated NH_4_Cl solution (20 mL) was added, and the mixture was extracted with EtOAc (3 × 20 mL). The combined organic layers were dried over anhydrous MgSO_4_, filtered, and concentrated in vacuo. The crude product was purified by column chromatography on silica gel via 9:1 to 4:1 petroleum ether/EtOAc elution gradient to yield **5a** as an off-white solid (81 mg, 0.48 mmol, 96% yield). TLC(petroleum ether/EtOAc 9:1) *R*_f_ = 0.15; mp= 65–66 °C. Lit. 68–69 °C;^8^^1^H NMR (500 MHz, CDCl_3_) δ_H_ 8.44 (dd, *J* = 4.8, 1.3 Hz, 1H), 8.28 (dd, *J* = 7.5, 1.0 Hz, 1H), 7.95 (dd, *J* = 7.7, 0.6 Hz, 1H), 7.65 (d, *J* = 8.3 Hz, 1H), 7.53 (ddd, *J* = 8.4, 7.5, 1.3 Hz, 1H), 7.39 (td, *J* = 7.7, 0.8 Hz, 1H), 7.36 (dd, *J* = 6.8, 4.4 Hz, 1H); ^13^C{^1^H} NMR (126 MHz, CDCl_3_) δ_C_ 163.2, 154.7, 146.3, 129.9, 128.6, 123.5, 122.6, 121.5, 119.4, 117.3, 112.3. The analytical data match those reported in the literature.^8^

### Typical Procedure for the Synthesis of Benzofuro[2,3-*c*]pyridines **5** and Benzofuro[2,3-*b*]pyridines with Strongly Electron-Withdrawing Substituents

#### Benzofuro[2,3-*c*]pyridine (**5e**)

To a solution of 3-fluoropyridine (52 μL, 0.60 mmol, 1.2 equiv) in anhydrous THF (2.4 mL), was added a solution of LDA (2.0 M, 325 μL, 0.65 mmol, 1.3 equiv) dropwise at −25 °C. The reaction mixture was stirred at −25 °C for 5 min, followed by the addition of ZnCl_2_ solution in THF (0.7 M, 930 μL, 0.65 mmol, 1.3 equiv). The cooling bath was removed, and the reaction mixture was allowed to warm to room temperature, after which a solution of **3a** (108 mg, 0.50 mmol, 1.0 equiv), precatalyst Pd XPhos G3 (8.5 mg, 10 μmol, 2.0 mol %) and XPhos (4.8 mg, 10 μmol, 2.0 mol %) in THF (1.0 mL) was added. The reaction mixture was stirred at 70 °C in a heated oil bath for 20 min. The solvent was removed under reduced pressure, and the residue was dissolved in anhydrous DMF (5.0 mL). Cs_2_CO_3_ (815 mg, 2.50 mmol, 5.0 equiv) was added, and the reaction mixture was stirred at 100 °C in a heated oil bath for 2 h. After cooling to room temperature, saturated NH_4_Cl solution (20 mL) was added, and the mixture was extracted with EtOAc (3 × 20 mL). The combined organic layers were washed with water (4 × 30 mL) and brine (30 mL), dried over anhydrous MgSO_4_, filtered, and concentrated in vacuo. The crude product was purified by column chromatography on silica gel via 4:1 to 2:1 petroleum ether/EtOAc elution gradient to give **5e** as an off-white solid (33 mg, 0.20 mmol, 39% yield). TLC(petroleum ether/EtOAc 4:1) *R*_f_ = 0.14; mp = 90.5–91.5 °C. Lit. 93–95 °C;^8^^1^H NMR (500 MHz, CDCl_3_) δ_H_ 8.99 (s, 1H), 8.59 (d, *J* = 5.1 Hz, 1H), 8.03 (d, *J* = 7.8 Hz, 1H), 7.87 (d, *J* = 5.0 Hz, 1H), 7.65 (d, *J* = 8.2 Hz, 1H), 7.61 (t, *J* = 7.8 Hz, 1H), 7.42 (t, *J* = 7.4 Hz, 1H); ^13^C{^1^H} NMR (126 MHz, CDCl_3_) δ_C_ 156.9, 152.9, 143.1, 134.6, 131.1, 130.1, 123.6, 122.3, 122.2, 115.3, 112.6. The analytical data match those reported in the literature.^8^

### Typical Procedure for the Synthesis of Dibenzofurans **6**

#### Dibenzo[b,d]furan (**6a**)

To a solution of fluorobenzene (56 μL, 0.60 mmol, 1.2 equiv) and KO*t*Bu (1.6 M, 403 μL, 0.65 mmol, 1.3 equiv) in anhydrous THF (2.4 mL), was added a solution of *n*BuLi (1.58 M, 411 μL, 0.65 mmol, 1.3 equiv) dropwise at −78 °C. The reaction mixture was stirred at −78 °C for 5 min, followed by the addition of ZnCl_2_ solution in THF (0.7 M, 930 μL, 0.65 mmol, 1.3 equiv). The cooling bath was removed, and the reaction mixture was allowed to warm to room temperature, after which a solution of **3a** (108 mg, 0.50 mmol, 1.0 equiv), precatalyst Pd XPhos G3 (8.5 mg, 10 μmol, 2.0 mol %), and XPhos (4.8 mg, 10 μmol, 2.0 mol %) in THF (1.0 mL) was added. The reaction mixture was stirred at 70 °C in a heated oil bath for 20 min. The solvent was removed under reduced pressure, and the residue was dissolved in anhydrous DMF (5.0 mL). Cs_2_CO_3_ (815 mg, 2.50 mmol, 5.0 equiv) was added, and the reaction mixture was stirred at 120 °C in a heated oil bath overnight. After cooling to room temperature, saturated NH_4_Cl solution (20 mL) was added, and the mixture was extracted with EtOAc (3 × 20 mL). The combined organic layers were washed with water (4 × 30 mL) and brine (30 mL), dried over anhydrous MgSO_4_, filtered, and concentrated in vacuo. The crude product was purified by column chromatography on silica gel using 95:5 petroleum ether/EtOAc to give **6a** as a waxy white solid (66 mg, 0.39 mmol, 78% yield). TLC(petroleum ether/EtOAc 9:1) *R*_f_ = 0.59; mp = 78–80 °C. Lit. 83–84 °C^24^; ^1^H NMR (400 MHz, CDCl_3_) δ_H_ 7.97 (d, *J* = 7.7 Hz, 2H), 7.60 (d, *J* = 8.2 Hz, 2H), 7.48 (t, *J* = 7.7 Hz, 2H), 7.36 (t, *J* = 7.5 Hz, 2H); ^13^C{^1^H} NMR (126 MHz, CDCl_3_) δ_C_ 156.3, 127.3, 124.4, 122.8, 120.8, 111.8. The analytical data match those reported in the literature.^24^

## Supporting Information Available

Experimental procedures and characterization data for all new compounds, copies of ^1^H, ^13^C, and ^19^F NMR spectra, as well as XRD data for **5q** and **6c** (PDF)
