# Supplementary material for: Synthesis of Benzofuropyridines and Dibenzofurans by a Metalation/Negishi Cross-Coupling/SNAr Reaction Sequence
Source: J Org Chem. 2022 Dec 9;88(1):684–9. doi: 10.1021/acs.joc.2c02111 (PMC9830636; doi:10.1021/acs.joc.2c02111)

Supporting Information

Synthesis of Benzofuropyridines and Dibenzofurans by a Metalation/Negishi Cross-Coupling/S_NAr Reaction Sequence

Guy J. Clarkson, Stefan Roesner*

Department of Chemistry, University of Warwick,

Gibbet Hill Road, Coventry, CV4 7AL, UK

E-mail: s.roesner@warwick.ac.uk

Table of Contents

1. General information S2

2. Detailed procedures and analytical data S3

2.1 General procedures S3

2.2 Preparation of 2-bromophenyl acetates S5

2.3 Preparation of 2-fluorobiaryls S9

2.4 Preparation of benzofuropyridines S10

2.5 Preparation of dibenzofurans S19

2.6 Extended optimization table S23

2.7 Low-yielding and unsuccessful substrates S24

3. References S25

4. ^{1}H, ^{13}C and ^{19}F NMR spectra S26

5. Single crystal X-ray structures S77

1. General information

Reaction mixtures were stirred magnetically. All reactions were performed under nitrogen atmosphere using oven-dried glassware. Cooling baths were prepared from H_2O/MeOH/dry ice to reach a temperature range of –25 to –28 ºC (H_2O/MeOH 7:3). Overnight reaction times refer to 16–20 h.

All chemicals were purchased from Acros Organics, Alfa Aesar, Apollo Scientific, Fluorochem, or Sigma-Aldrich and used as received unless other noted. Palladium precatalyst Pd XPhos G3 was prepared according to the literature procedure.[1] Lithium diisopropylamide (LDA) in THF/*n*-heptane/ethylbenzene was purchased from Acros Organics as 2.0 M solution. *n*-Butyllithium was obtained from Sigma-Aldrich as 2.5 M solution in hexanes. The molarity of lithium reagents was regularly determined by titration using *N*-benzyl benzamide as an indicator.[2] Zinc chloride ($ZnCl_2$) was received as 0.7 M solution in THF from Acros Organics. Potassium *tert*-butoxide (KO*t*Bu) was purchased from Acros Organics as 1.6 M solution in THF. Anhydrous THF was purchased from Acros Organics in Sure-Seal™ bottles. Petroleum ether refers to the fraction that boils in the range of 40–60 °C. All mixed solvent eluents are reported as v/v solutions. Flash column chromatography was performed on silica gel (Sigma-Aldrich, Silica Gel 60, 40–63 µm).

All new compounds were characterized by NMR spectroscopy, IR spectroscopy, high-resolution mass spectrometry (HRMS), and melting point (mp). ^{1}H NMR spectra were recorded in $CDCl_3$ or DMSO-*d*6 on a Bruker HD400 (400 MHz), AV500 (500 MHz) or AV600 (600 MHz) Fourier transform spectrometer. Chemical shifts (δ_H) are recorded in parts per million (ppm) and referred to the residual protio-solvent signals of $CHCl_3$ (7.26 ppm) or DMSO (2.50 ppm). ^{1}H NMR coupling constants are reported in hertz (Hz) and refer to apparent multiplicities. ^{13}C NMR spectra were recorded at 101, 126 or 151 MHz and were obtained with ^{1}H decoupling. Chemical shifts (δ_C) are recorded in ppm referenced to $CDCl_3$ (77.16 ppm) or DMSO-*d*6 (39.52 ppm) and coupling constants are reported in hertz. ^{19}F NMR spectra were recorded at 376 MHz and were measured with ^{1}H decoupling. The infrared spectra were recorded of the neat compounds using a Perkin Elmer Spectrum 100 FT-IR spectrometer, irradiating between 4000 cm^{-1} and 600 cm^{-1}. High resolution mass spectra were recorded using a Bruker MaXis Impact Q-TOF. Melting points were measured with a Gallenkamp MPD350 melting point apparatus. Analytical TLC was performed on pre-coated aluminium-backed plates (Merck Silicagel 60 F254), visualized by UV 254 nm, then stained with potassium permanganate ($KMnO_4$) or phosphomolybdic acid (PMA) dip and heated. All reported yields are isolated yields after purification.

2. Detailed procedures and analytical data

2.1 General procedures

General Procedure 1 – Synthesis of 2-bromophenyl acetates 3

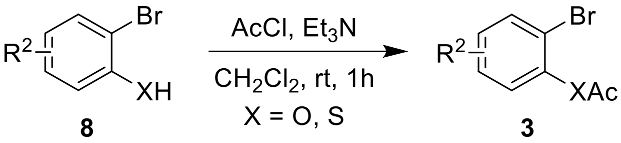

Following a procedure by Kónya,[3] to a solution of a 2-bromophenol or 2-bromothiophenol (**8**, 20.0 mmol, 1.0 equiv) in anhydrous CH_2Cl_2 (40 mL, 0.5 M) in a round-bottom flask were added acetyl chloride (22.0 mmol, 1.1 equiv) and Et_3N (22.0 mmol, 1.1 equiv). The reaction mixture was stirred for 1 h at room temperature. Then, the organic layer was washed with saturated aqueous $NaHCO_3$ solution (20 mL) and the aqueous layer was extracted with CH_2Cl_2 (2 × 20 mL). The combined organic layers were dried over anhydrous $MgSO_4$, filtered, and the solvent was removed under reduced pressure to give the pure acetates **3**, which were used without further purification.

General Procedure 2 – Synthesis of benzofuro[2,3-*b*]pyridines 5

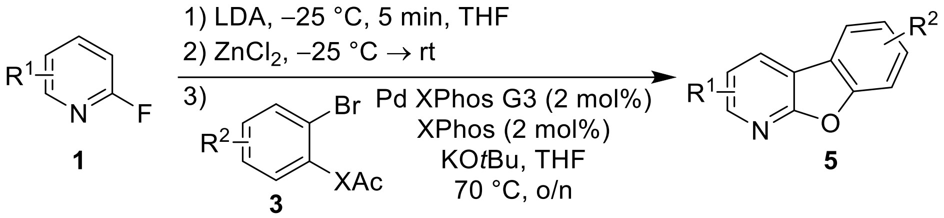

To a solution of fluoro-substituted pyridine **1** (0.60 mmol, 1.2 equiv) in anhydrous THF (2.4 mL, 0.25 M), was added a solution of LDA (0.65 mmol, 1.3 equiv) dropwise at –25 ºC. The reaction mixture was stirred at –25 ºC for 5 min, followed by the addition of $ZnCl_2$ solution in THF (0.65 mmol, 1.3 equiv). The cooling bath was removed, and the reaction mixture was allowed to warm to room temperature (ca. 3 min), after which a solution of 2-bromophenyl acetate **3** (0.50 mmol, 1.0 equiv), precatalyst Pd XPhos G3 (10 µmol, 2.0 mol%) and XPhos (10 µmol, 2.0 mol%) in THF (1.0 mL, 0.5 M) followed by a solution of KO*t*Bu in THF (1.00 mmol, 2.0 equiv) was added. The reaction mixture was stirred at 70 ºC in a heated oil bath overnight. Saturated NH_4Cl solution (20 mL) was added, and the mixture was extracted with EtOAc (3 × 20 mL). The combined organic layers were dried over anhydrous $MgSO_4$, filtered, and concentrated *in vacuo*. The crude product was purified by column chromatography on silica gel to yield the pure benzofuro[2,3-*b*]pyridines **5**.

General Procedure 3a – Synthesis of benzofuro[2,3-*c*]pyridines and benzofuro[2,3-*b*] pyridines with strongly electron-withdrawing substituents

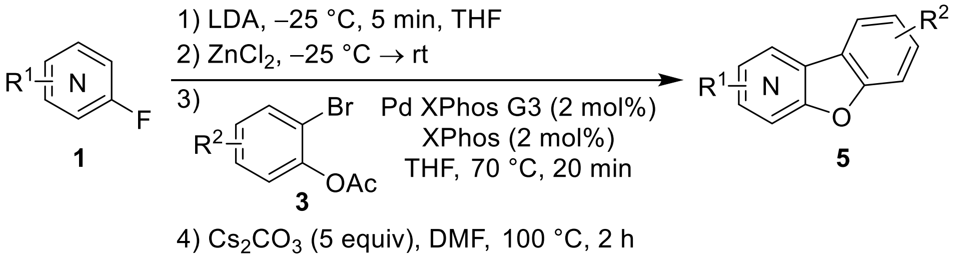

Following general procedure 2, no KO*t*Bu solution was added for the Negishi cross-coupling. The reaction mixture was stirred at 70 ºC in a heated oil bath for 20 min. The solvent was removed under reduced pressure and the residue was dissolved in anhydrous DMF (5.0 mL, 0.1 M). Cs_2CO_3 (2.50 mmol, 5.0 equiv) was added, and the reaction mixture was stirred at 100 ºC in a heated oil bath for 2 h. After cooling to room temperature, saturated NH_4Cl solution (20 mL) was added, and the mixture was extracted with EtOAc (3 × 20 mL). The combined organic layers were washed with water (4 × 30 mL) and brine (30 mL), dried over anhydrous $MgSO_4$, filtered, and concentrated *in vacuo*. The crude product was purified by column chromatography on silica gel to yield the pure benzofuropyridines **5**.

General Procedure 3b

Following general procedure 3a, the reaction was conducted as two-step procedure. After Negishi cross-coupling, the 2-fluorobiaryl was isolated. Then, the purified biaryl was subjected to the S_NAr reaction conditions.

General Procedure 4 – Synthesis of dibenzofurans 6

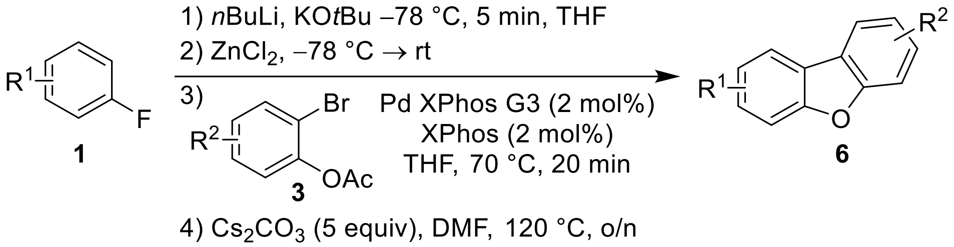

To a solution of fluoroarene **1** (0.60 mmol, 1.2 equiv) and KO*t*Bu (0.65 mmol, 1.3 equiv) in anhydrous THF (2.4 mL, 0.25 M), was added a solution of *n*BuLi (0.65 mmol, 1.3 equiv) dropwise at –78 ºC. The reaction mixture was stirred at –78 ºC for 5 min, followed by the addition of $ZnCl_2$ solution in THF (0.65 mmol, 1.3 equiv). The cooling bath was removed, and the reaction mixture was allowed to warm to room temperature, after which a solution of 2-bromophenyl acetate **3** (0.50 mmol, 1.0 equiv), precatalyst Pd XPhos G3 (10 µmol, 2.0 mol%) and XPhos (10 µmol, 2.0 mol%) in THF (1.0 mL, 0.5 M) was added. The reaction mixture was stirred at 70 ºC in a heated oil bath for 20 min. The solvent was removed under reduced pressure and the residue was dissolved in anhydrous DMF (5.0 mL, 0.1 M). Cs_2CO_3 (2.50 mmol, 5.0 equiv) was added, and the reaction mixture was stirred at 120 ºC in a heated oil bath overnight. After cooling to room temperature, saturated NH_4Cl solution (20 mL) was added, and the mixture was extracted with EtOAc (3 × 20 mL). The combined organic layers were washed with water (4 × 30 mL) and brine (30 mL), dried over anhydrous $MgSO_4$, filtered, and concentrated *in vacuo*. The crude product was purified by column chromatography on silica gel to yield the pure dibenzofurans **6**.

2.2 Preparation of 2-bromophenyl acetates

2-Bromophenyl acetate (3a)

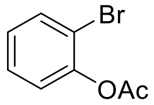

General procedure 1 was followed with 2-bromophenol (3.46 g, 20.0 mmol, 1.0 equiv), acetyl chloride (1.56 mL, 22.0 mmol, 1.1 equiv) and triethylamine (3.04 mL, 22.0 mmol, 1.1 equiv) in CH_2Cl_2 (40 mL) to give **3a** as a pale-yellow oil (4.26 g, 19.8 mmol, 99% yield). The crude product was used without further purification.

^{1}H NMR (400 MHz, $CDCl_3$) δ_H 7.61 (dd, *J* = 8.3, 0.9 Hz, 1H), 7.33 (t, *J* = 7.7 Hz, 1H), 7.16–7.09 (m, 2H), 2.36 (s, 3H);

^{13}C{^{1}H} NMR (101 MHz, $CDCl_3$) δ_C 168.7, 148.4, 133.5, 128.6, 127.5, 123.9, 116.40, 20.9.

The analytical data match those reported in the literature.[4]

2-Bromo-3-fluorophenyl acetate (3b)

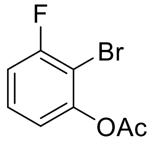

General procedure 1 was followed with 2-bromo-3-fluorophenol (1.06 g, 5.55 mmol, 1.0 equiv), acetyl chloride (434 μL, 6.11 mmol, 1.1 equiv) and triethylamine (847 μL, 6.11 mmol, 1.1 equiv) in CH_2Cl_2 (20 mL) to give **3b** as a pale-yellow oil (1.29 g, 5.55 mmol, quant. yield). The crude product was used without further purification.

^{1}H NMR (500 MHz, $CDCl_3$) δ_H 7.31 (td, *J* = 8.3, 6.0 Hz, 1H), 7.04 (td, *J* = 8.3, 1.1 Hz, 1H), 6.96 (d, *J* = 8.2 Hz, 1H), 2.37 (s, 3H);

^{13}C{^{1}H} NMR (126 MHz, $CDCl_3$) δ_C 168.4, 160.2 (d, *J* = 249 Hz), 149.9 (d, *J* = 2.7 Hz), 128.6 (d, *J* = 9.0 Hz), 119.3 (d, *J* = 3.4 Hz), 114.0 (d, *J* = 22.3 Hz), 104.9 (d, *J* = 22.6 Hz), 20.9;

^{19}F{^{1}H} NMR (376 MHz, $CDCl_3$) δ_F –104.6;

ν_{max} (neat) 1771, 1463, 1180, 1018, 864, 793, 706 cm^{-1};

HRMS (ESI^+) calculated for $C_8H_6BrFNaO_2$ $[M+Na]^+$ 254.9427, found 254.9427.

2-Bromo-4-methylphenyl acetate (3c)

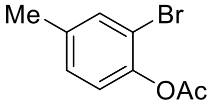

General procedure 1 was followed with 2-bromo-4-methylphenol (1.21 mL, 10.0 mmol, 1.0 equiv), acetyl chloride (782 μL, 11.0 mmol, 1.1 equiv) and triethylamine (1.52 mL, 11.0 mmol, 1.1 equiv) in CH_2Cl_2 (20 mL) to give **3c** as a pale-yellow oil (2.28 g, 9.99 mmol, >99% yield). The crude product was used without further purification.

^{1}H NMR (500 MHz, $CDCl_3$) δ_H 7.42 (d, *J* = 1.2 Hz, 1H), 7.12 (dd, *J* = 8.2, 1.2 Hz, 1H), 7.00 (d, *J* = 8.2 Hz, 1H), 2.34 (s, 3H), 2.33 (s, 3H);

^{13}C{^{1}H} NMR (126 MHz, $CDCl_3$) δ_C 168.9, 146.1, 137.7, 133.8, 129.3, 123.4, 115.9, 20.9, 20.7;

ν_{max} (neat) 1764, 1486, 1367, 1180, 1046, 1008, 908, 863, 819 cm^{-1};

HRMS (ESI^+) calculated for $C_9H_{10}BrO_2$ $[M+H]^+$ 228.9859, found 228.9865.

2-Bromo-4-fluorophenyl acetate (3d)

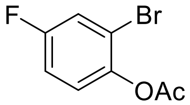

General procedure 1 was followed with 2-bromo-4-fluorophenol (1.90 g, 10.0 mmol, 1.0 equiv), acetyl chloride (782 μL, 11.0 mmol, 1.1 equiv) and triethylamine (1.52 mL, 11.0 mmol, 1.1 equiv) in CH_2Cl_2 (20 mL) to give **3d** as a pale-yellow oil (2.29 g, 9.93 mmol, >99% yield). The crude product was used without further purification.

^{1}H NMR (500 MHz, $CDCl_3$) δ_H 7.35 (dd, *J* = 7.8, 2.8 Hz, 1H), 7.10 (dd, *J* = 8.9, 5.1 Hz, 1H), 7.05 (ddd, *J* = 8.9, 7.7, 2.8 Hz, 1H), 2.35 (s, 3H);

^{13}C{^{1}H} NMR (126 MHz, $CDCl_3$) δ_C 168.7, 160.1 (d, *J* = 249 Hz), 144.7 (d, *J* = 3.5 Hz), 124.5 (d, *J* = 8.9 Hz), 120.5 (d, *J* = 26.0 Hz), 116.7 (d, *J* = 10.1 Hz), 115.5 (d, *J* = 23.1 Hz), 20.8;

^{19}F{^{1}H} NMR (376 MHz, $CDCl_3$) δ_F –114.5;

ν_{max} (neat) 1767, 1597, 1482, 1369, 1201, 1172, 1037, 1010, 899 cm^{-1};

HRMS (ESI^+) calculated for $C_8H_7BrFO_2$ $[M+H]^+$ 232.9608, found 232.9620.

2-Bromo-4-chlorophenyl acetate (3e)

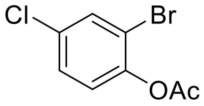

General procedure 1 was followed with 2-bromo-4-chlorophenol (2.07 g, 10.0 mmol, 1.0 equiv), acetyl chloride (782 μL, 11.0 mmol, 1.1 equiv) and triethylamine (1.52 mL, 11.0 mmol, 1.1 equiv) in CH_2Cl_2 (20 mL) to give **3e** as a pale-yellow oil (2.48 g, 9.94 mmol, >99% yield). The crude product was used without further purification.

^{1}H NMR (600 MHz, $CDCl_3$) δ_H 7.61 (d, *J* = 2.4 Hz, 1H), 7.31 (dd, *J* = 8.6, 2.4 Hz, 1H), 7.07 (d, *J* = 8.6 Hz, 1H), 2.35 (s, 3H);

^{13}C{^{1}H} NMR (151 MHz, $CDCl_3$) δ_C 168.4, 147.2, 133.1, 132.3, 128.8, 124.7, 117.0, 20.9;

ν_{max} (neat) 1768, 1468, 1369, 1184, 1094, 1045, 1009, 905, 746 cm^{-1};

HRMS (ESI^+) calculated for $C_8H_6BrClNaO_2$ $[M+Na]^+$ 270.9132, found 270.9142.

2-Bromo-4-methoxyphenyl acetate (3f)

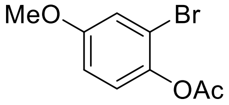

General procedure 1 was followed with 2-bromo-4-methoxyphenol (1.17 g, 5.76 mmol, 1.0 equiv), acetyl chloride (451 μL, 6.34 mmol, 1.1 equiv) and triethylamine (879 μL, 6.34 mmol, 1.1 equiv) in CH_2Cl_2 (20 mL) to give **3f** as a pale-yellow oil (1.40 g, 5.70 mmol, 99% yield). The crude product was used without further purification.

^{1}H NMR (600 MHz, $CDCl_3$) δ_H 7.13 (d, *J* = 2.9 Hz, 1H), 7.03 (d, *J* = 8.9 Hz, 1H), 6.85 (dd, *J* = 8.9, 2.9 Hz, 1H), 3.79 (s, 3H), 2.33 (s, 3H);

^{13}C{^{1}H} NMR (151 MHz, $CDCl_3$) δ_C 169.2, 158.0, 142.0, 124.0, 118.3, 116.5, 114.3, 56.0, 20.9.

The analytical data match those reported in the literature.[5]

2-Bromo-1,4-phenylene diacetate (3g)

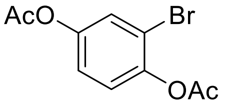

General procedure 1 was followed with bromohydroquinone (1.13 g, 5.98 mmol, 1.0 equiv), acetyl chloride (935 μL, 13.1 mmol, 2.2 equiv) and triethylamine (1.82 mL, 13.1 mmol, 2.2 equiv) in CH_2Cl_2 (40 mL) to give **3g** as an off-white solid (1.63 g, 5.98 mmol, >99% yield). The crude product was used without further purification.

mp = 77–79 °C. Lit. 71–73 ºC;[a]

^{1}H NMR (500 MHz, $CDCl_3$) δ_H 7.39 (d, *J* = 2.6 Hz, 1H), 7.13 (d, *J* = 8.8 Hz, 1H), 7.08 (dd, *J* = 8.8, 2.6 Hz, 1H), 2.35 (s, 3H), 2.29 (s, 3H);

^{13}C{^{1}H} NMR (126 MHz, $CDCl_3$) δ_C 169.0, 168.6, 148.6, 146.0, 126.6, 124.0, 121.8, 116.3, 21.1, 20.9.

The analytical data match those reported in the literature.[6]

2-Bromo-4-cyanophenyl acetate (3h)

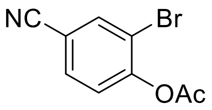

General procedure 1 was followed with 3-bromo-4-hydroxybenzonitrile (1.98 g, 10.0 mmol, 1.0 equiv), acetyl chloride (782 μL, 11.0 mmol, 1.1 equiv) and triethylamine (1.52 mL, 11.0 mmol, 1.1 equiv) in CH_2Cl_2 (20 mL) to give **3h** as an off-white solid (2.39 g, 9.96 mmol, >99% yield). The crude product was used without further purification.

mp = 101–102 °C. Lit. 88–90 ºC;[7]

^{1}H NMR (600 MHz, $CDCl_3$) δ_H 7.91 (d, *J* = 1.9 Hz, 1H), 7.64 (dd, *J* = 8.4, 1.9 Hz, 1H), 7.26 (d, *J* = 8.4 Hz, 1H), 2.38 (s, 3H);

^{13}C{^{1}H} NMR (151 MHz, $CDCl_3$) δ_C 167.8, 152.1, 137.1, 132.5, 124.9, 117.5, 116.9, 111.6, 20.9.

The analytical data match those reported in the literature.[5]

2-Bromo-4-nitrophenyl acetate (3i)

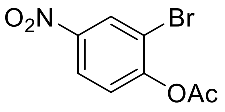

General procedure 1 was followed with 2-bromo-4-nitrophenol (315 mg, 1.44 mmol, 1.0 equiv), acetyl chloride (120 μL, 1.59 mmol, 1.1 equiv) and triethylamine (220 μL, 1.59 mmol, 1.1 equiv) in CH_2Cl_2 (5.0 mL) to give **3i** as an off-white solid (372 mg, 1.43 mmol, >99% yield). The crude product was used without further purification.

mp = 66–68 °C;

^{1}H NMR (500 MHz, $CDCl_3$) δ_H 8.51 (d, *J* = 2.6 Hz, 1H), 8.22 (dd, *J* = 8.9, 2.6 Hz, 1H), 7.33 (d, *J* = 8.9 Hz, 1H), 2.40 (s, 3H);

^{13}C{^{1}H} NMR (126 MHz, $CDCl_3$) δ_C 167.7, 153.4, 145.9, 129.0, 124.4, 123.9, 117.2, 20.9;

ν_{max} (neat) 3100, 1768, 1523, 1348, 1177, 1043, 910, 746, 725 cm^{-1};

HRMS (ESI^+) calculated for $C_8H_6BrNNaO_4$ $[M+Na]^+$ 281.9372, found 281.9378.

2-Bromo-5-fluorophenyl acetate (3j)

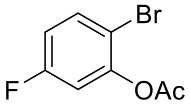

General procedure 1 was followed with 2-bromo-5-fluorophenol (1.91 g, 10.0 mmol, 1.0 equiv), acetyl chloride (782 μL, 11.0 mmol, 1.1 equiv) and triethylamine (1.52 mL, 11.0 mmol, 1.1 equiv) in CH_2Cl_2 (20 mL) to give **3j** as a pale-yellow oil (2.29 g, 9.91 mmol, >99% yield). The crude product was used without further purification.

^{1}H NMR (500 MHz, $CDCl_3$) δ_H 7.56 (dd, J = 8.8, 5.8 Hz, 1H), 6.94–6.87 (m, 2H), 2.36 (s, 3H);

^{13}C{^{1}H} NMR (126 MHz, $CDCl_3$) δ_C 168.2, 162.1 (d, J = 249 Hz), 149.1 (d, J = 11.1 Hz), 133.9 (d, J = 9.0 Hz), 114.8 (d, J = 22.3 Hz), 112.1 (d, J = 25.2 Hz), 111.0 (d, J = 4.1 Hz), 20.9;

^{19}F{^{1}H} NMR (376 MHz, $CDCl_3$) δ_F –112.0.

The analytical data match those reported in the literature.[7]

2-Bromo-6-methoxyphenyl acetate (3k)

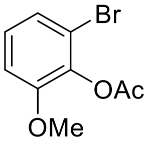

General procedure 1 was followed with 2-bromo-6-methoxyphenol (2.03 g, 10.0 mmol, 1.0 equiv), acetyl chloride (782 μL, 11.0 mmol, 1.1 equiv) and triethylamine (1.52 mL, 11.0 mmol, 1.1 equiv) in CH_2Cl_2 (20 mL) to give **3k** as a pale-yellow oil (2.45 g, 10.0 mmol, >99% yield). The crude product was used without further purification.

^{1}H NMR (500 MHz, $CDCl_3$) δ_H 7.18 (dd, J = 8.2, 1.4 Hz, 1 H), 7.07 (t, J = 8.2 Hz, 1 H), 6.90 (dd, J = 8.2, 1.4 Hz, 1 H), 3.83 (s, 3 H), 2.36 (s, 3 H);

^{13}C{^{1}H} NMR (126 MHz, $CDCl_3$) δ_C 168.1, 152.8, 138.1, 127.4, 124.6, 117.4, 111.5, 56.4, 20.6.

The analytical data match those reported in the literature.[8]

2-Bromo-6-nitrophenyl acetate (3l)

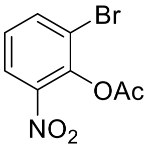

General procedure 1 was followed with 2-bromo-6-nitrophenol (2.18 g, 10.0 mmol, 1.0 equiv), acetyl chloride (782 μL, 11.0 mmol, 1.1 equiv) and triethylamine (1.52 mL, 11.0 mmol, 1.1 equiv) in CH_2Cl_2 (20 mL) to give **3l** as an off-white solid (2.60 g, 9.99 mmol, >99% yield). The crude product was used without further purification.

mp = 41–44 °C;

^{1}H NMR (500 MHz, $CDCl_3$) δ_H 8.01 (dd, J = 8.3, 1.4 Hz, 1H), 7.87 (dd, J = 8.1, 1.4 Hz, 1H), 7.28 (t, J = 8.2 Hz, 1H), 2.41 (s, 3H);

^{13}C{^{1}H} NMR (126 MHz, $CDCl_3$) δ_C 167.4, 143.2, 142.4, 138.3, 127.3, 125.0, 119.9, 20.6;

ν_{max} (neat) 3100, 1774, 1525, 1345, 1171, 899 cm^{-1};

HRMS (ESI$^+$) calculated for $C_8H_6BrNNaO_4$ [M+Na]$^+$ 281.9372, found 281.9373.

1-Bromonaphthalen-2-yl acetate (3m)

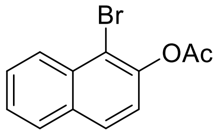

General procedure 1 was followed with 1-bromo-2-naphthol (2.23 g, 10.0 mmol, 1.0 equiv), acetyl chloride (782 μL, 11.0 mmol, 1.1 equiv) and triethylamine (1.52 mL, 11.0 mmol, 1.1 equiv) in CH_2Cl_2 (20 mL) to give **3m** as a brown oil (2.65 g, 10.0 mmol, >99% yield). The crude product was used without further purification.

^{1}H NMR (600 MHz, $CDCl_3$) δ_H 8.27 (d, *J* = 8.5 Hz, 1H), 7.86 (d, *J* = 8.0 Hz, 1H), 7.84 (d, *J* = 8.7 Hz, 1H), 7.62 (ddd, *J* = 8.3, 6.9, 1.2 Hz, 1H), 7.53 (ddd, *J* = 8.0, 6.9, 1.1 Hz, 1H), 7.26 (d, *J* = 8.8 Hz, 1H), 2.43 (s, 3H);

^{13}C{^{1}H} NMR (151 MHz, $CDCl_3$) δ_C 168.9, 146.5, 132.8, 132.6, 129.0, 128.3, 128.0, 127.2, 126.5, 122.0, 115.3, 21.1.

The analytical data match those reported in the literature.[9]

***S*-(2-Bromophenyl) thioacetate (3n)**

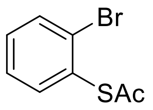

General procedure 1 was followed with 2-bromothiophenol (1.20 mL, 10.0 mmol, 1.0 equiv), acetyl chloride (782 μL, 11.0 mmol, 1.1 equiv) and triethylamine (1.52 mL, 11.0 mmol, 1.1 equiv) in CH_2Cl_2 (20 mL) to give **3n** as a pale-yellow oil (2.31 g, 10.0 mmol, >99% yield). The crude product was used without further purification.

^{1}H NMR (600 MHz, $CDCl_3$) δ_H 7.70 (dd, *J* = 7.6, 1.2 Hz, 1H), 7.54 (dd, *J* = 7.6, 1.6 Hz, 1H), 7.36 (td, *J* = 7.6, 1.2 Hz, 1H), 7.27 (td, *J* = 7.6, 1.6 Hz, 1H), 2.45 (s, 3H);

^{13}C{^{1}H} NMR (126 MHz, $CDCl_3$) δ_C 192.2, 137.1, 133.6, 131.2, 129.7, 129.3, 128.0, 30.3;

ν_{max} (neat) 1706, 1448, 1429, 1351, 1110, 1021, 948, 753 cm^{-1};

HRMS (ESI^+) calculated for C_8H_8BrOS $[M+H]^+$ 230.9474, found 230.9483.

2.3 Preparation of 2-fluorobiaryls

2-(2-Fluoropyridin-3-yl)phenyl acetate (4a)

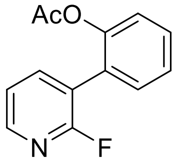

General procedure 2 was followed with 2-fluoropyridine (52 μL, 0.60 mmol, 1.2 equiv) and 2-bromophenyl acetate (**3a**) (108 mg, 0.50 mmol, 1.0 equiv) without the use of KO*t*Bu. The reaction mixture was stirred at 70 ºC in an oil bath for 20 min to facilitate the Negishi cross-coupling. The crude product was purified by column chromatography via 4:1 to 2:1 petroleum ether/EtOAc elution gradient to give **4a** as an off-white solid (97 mg, 0.42 mmol, 84% yield).

R_f (petroleum ether/EtOAc 2:1) 0.35;

mp = 81–82 °C;

^{1}H NMR (500 MHz, $CDCl_3$) δ_H 8.38 (d, *J* = 4.5 Hz, 1H), 7.89 (ddd, *J* = 9.3, 7.4, 1.9 Hz, 1H), 7.60 (td, *J* = 8.2, 1.8 Hz, 1H), 7.52 (dd, *J* = 7.5, 1.5 Hz, 1H), 7.47 (td, *J* = 7.6, 0.9 Hz, 1H), 7.42–7.39 (m, 1H), 7.36 (dd, *J* = 8.1, 0.5 Hz, 1H), 2.23 (s, 3H);

$^{13}C\{^1H\}$ **NMR** (126 MHz, $CDCl_3$) δ_C 169.1, 160.6 (d, J = 240 Hz), 148.3, 147.3 (d, J = 14.4 Hz), 142.0 (d, J = 4.3 Hz), 131.2, 130.1, 127.2 (d, J = 4.4 Hz), 126.4, 123.1, 121.5 (d, J = 4.4 Hz), 120.2 (d, J = 31.2 Hz), 20.9;

$^{19}F\{^1H\}$ **NMR** (376 MHz, $CDCl_3$) δ_F –68.5;

ν_{max} (neat) 2944, 1744, 1417, 1179, 1001, 913, 851, 807, 776, 761 cm^{-1};

HRMS (ESI^+) calculated for $C_{13}H_{10}FNNaO_2$ $[M+Na]^+$ 254.0588, found 254.0589.

2.4 Preparation of benzofuropyridines

Benzofuro[2,3-*b*]pyridine (5a)

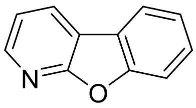

General procedure 2 was followed with 2-fluoropyridine (52 µL, 0.60 mmol, 1.2 equiv) and 2-bromophenyl acetate (**3a**) (108 mg, 0.50 mmol, 1.0 equiv). The crude product was purified by column chromatography via 9:1 to 4:1 petroleum ether/EtOAc elution gradient to give **5a** as an off-white solid (81 mg, 0.48 mmol, 96% yield).

R_f (petroleum ether/EtOAc 9:1) 0.15;

mp = 65–66 °C. Lit. 68–69 ºC;[10]

^1H **NMR** (500 MHz, $CDCl_3$) δ_H 8.44 (dd, J = 4.8, 1.3 Hz, 1H), 8.28 (dd, J = 7.5, 1.0 Hz, 1H), 7.95 (dd, J = 7.7, 0.6 Hz, 1H), 7.65 (d, J = 8.3 Hz, 1H), 7.53 (ddd, J = 8.4, 7.5, 1.3 Hz, 1H), 7.39 (td, J = 7.7, 0.8 Hz, 1H), 7.36 (dd, J = 6.8, 4.4 Hz, 1H);

$^{13}C\{^1H\}$ **NMR** (126 MHz, $CDCl_3$) δ_C 163.2, 154.7, 146.3, 129.9, 128.6, 123.5, 122.6, 121.5, 119.4, 117.3, 112.3.

The analytical data match those reported in the literature.[10]

2-Fluorobenzofuro[2,3-*b*]pyridine (5b)

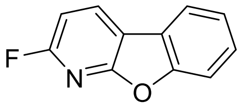

General procedure 2 was followed with 2,6-difluoropyridine (55 µL, 0.60 mmol, 1.2 equiv) and 2-bromophenyl acetate (**3a**) (108 mg, 0.50 mmol, 1.0 equiv). The crude product was purified by column chromatography via 9:1 to 4:1 petroleum ether/EtOAc elution gradient to give **5b** as a white solid (82 mg, 0.44 mmol, 88% yield).

R_f (petroleum ether/EtOAc 9:1) 0.22;

mp = 101–102 °C;

^1H **NMR** (500 MHz, $CDCl_3$) δ_H 8.32 (dd, J = 8.0, 7.6 Hz, 1H), 7.91 (dd, J = 7.7, 0.5 Hz, 1H), 7.64 (d, J = 8.3 Hz, 1H), 7.51 (ddd, J = 8.4, 7.5, 1.3 Hz, 1H), 7.40 (td, J = 7.6, 0.9 Hz, 1H), 6.99 (dd, J = 8.2, 1.3 Hz, 1H);

$^{13}C\{^1H\}$ **NMR** (126 MHz, $CDCl_3$) δ_C 161.9 (d, J = 244 Hz), 161.0, 154.9 (d, J = 2.6 Hz), 133.8 (d, J = 9.2 Hz), 128.0, 123.9, 122.2, 120.9, 114.3 (d, J = 4.3 Hz), 112.4, 104.5 (d, J = 36.8 Hz);

$^{19}F\{^1H\}$ NMR (376 MHz, $CDCl_3$) δ_F –69.7;

ν_{max} (neat) 1590, 1451, 1399, 1354, 1179, 987, 819, 774, 743, 728 cm^{-1};

HRMS (ESI^+) calculated for $C_{11}H_7FNO$ $[M+H]^+$ 188.0506, found 188.0515.

4-Fluorobenzofuro[2,3-*b*]pyridine (5c)

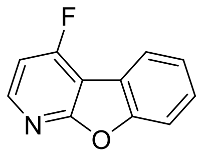

General procedure 2 was followed with 2,4-difluoropyridine (55 μL, 0.60 mmol, 1.2 equiv) and 2-bromophenyl acetate (**3a**) (108 mg, 0.50 mmol, 1.0 equiv). The crude product was purified by column chromatography using 9:1 petroleum ether/EtOAc to give **5c** as a white solid (83 mg, 0.44 mmol, 87% yield).

R_f (petroleum ether/EtOAc 9:1) 0.12;

mp = 85–86 °C;

^1H NMR (500 MHz, $CDCl_3$) δ_H 8.41 (dd, *J* = 7.8, 5.8 Hz, 1H), 8.04 (d, *J* = 7.7 Hz, 1H), 7.65 (d, *J* = 8.3 Hz, 1H), 7.56 (td, *J* = 7.9, 0.9 Hz, 1H), 7.43 (t, *J* = 7.5 Hz, 1H), 7.09 (dd, *J* = 8.6, 5.7 Hz, 1H);

$^{13}C\{^1H\}$ NMR (126 MHz, $CDCl_3$) δ_C 165.7 (d, *J* = 10.6 Hz), 164.1 (d, *J* = 267 Hz), 154.1, 148.2 (d, *J* = 7.0 Hz), 128.7, 124.1, 123.4 (d, *J* = 2.2 Hz), 120.1 (d, *J* = 2.0 Hz), 112.2, 107.4 (d, *J* = 15.6 Hz), 106.0 (d, *J* = 17.7 Hz);

$^{19}F\{^1H\}$ NMR (376 MHz, $CDCl_3$) δ_F –106.2;

ν_{max} (neat) 2919, 1582, 1447, 1370, 1264, 1182, 1040, 807, 743 cm^{-1};

HRMS (ESI^+) calculated for $C_{11}H_6FNNaO$ $[M+Na]^+$ 210.0336, found 210.0330.

3-Methylbenzofuro[2,3-*b*]pyridine (5d)

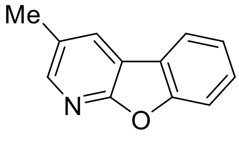

General procedure 2 was followed with 2-fluoro-5-methylpyridine (62 μL, 0.60 mmol, 1.2 equiv) and 2-bromophenyl acetate (**3a**) (108 mg, 0.50 mmol, 1.0 equiv). The crude product was purified by column chromatography via 9:1 to 4:1 petroleum ether/EtOAc elution gradient to give **5d** as a white solid (58 mg, 0.32 mmol, 63% yield).

R_f (petroleum ether/EtOAc 9:1) 0.22;

mp = 115–116 °C;

^1H NMR (500 MHz, $CDCl_3$) δ_H 8.26 (s, 1H), 8.06 (d, *J* = 1.3 Hz, 1H), 7.91 (d, *J* = 7.7 Hz, 1H), 7.61 (d, *J* = 8.3 Hz, 1H), 7.50 (td, *J* = 8.0, 1.0 Hz, 1H), 7.36 (t, *J* = 7.5 Hz, 1H), 2.50 (s, 3H);

$^{13}C\{^1H\}$ NMR (126 MHz, $CDCl_3$) δ_C 162.0, 155.0, 146.8, 130.1, 128.6, 128.3, 123.3, 122.6, 121.3, 116.6, 112.3, 18.5;

ν_{max} (neat) 2917, 1585, 1438, 1380, 1183, 880, 779, 730 cm^{-1};

HRMS (ESI^+) calculated for $C_{12}H_{10}NO$ $[M+H]^+$ 184.0757, found 184.0752.

Benzofuro[2,3-*c*]pyridine (5e)

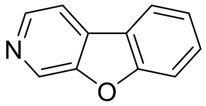

General procedure 3a was followed with 3-fluoropyridine (52 μL, 0.60 mmol, 1.2 equiv) and 2-bromophenyl acetate (**3a**) (108 mg, 0.50 mmol, 1.0 equiv). The crude product was purified by column chromatography via 4:1 to 2:1 petroleum ether/EtOAc elution gradient to give **5e** as an off-white solid (33 mg, 0.20 mmol, 39% yield).

R$_f$ (petroleum ether/EtOAc 4:1) 0.14;

mp = 90.5–91.5 °C. Lit. 93–95 °C;[10]

^{1}H NMR (500 MHz, $CDCl_3$) δ_H 8.99 (s, 1H), 8.59 (d, *J* = 5.1 Hz, 1H), 8.03 (d, *J* = 7.8 Hz, 1H), 7.87 (d, *J* = 5.0 Hz, 1H), 7.65 (d, *J* = 8.2 Hz, 1H), 7.61 (t, *J* = 7.8 Hz, 1H), 7.42 (t, *J* = 7.4 Hz, 1H);

^{13}C{^{1}H} NMR (126 MHz, $CDCl_3$) δ_C 156.9, 152.9, 143.1, 134.6, 131.1, 130.1, 123.6, 122.3, 122.2, 115.3, 112.6.

The analytical data match those reported in the literature.[10]

1-Fluorobenzofuro[2,3-*c*]pyridine (5f)

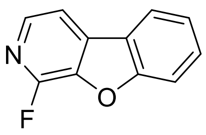

General procedure 3a was followed with 2,3-difluoropyridine (55 μL, 0.60 mmol, 1.2 equiv) and 2-bromophenyl acetate (**3a**) (108 mg, 0.50 mmol, 1.0 equiv). The crude product was purified by column chromatography using 9:1 petroleum ether/EtOAc to give **5f** as a white solid (75 mg, 0.40 mmol, 80% yield).

R$_f$ (petroleum ether/EtOAc 4:1) 0.35;

mp = 93–95 °C;

^{1}H NMR (400 MHz, $CDCl_3$) δ_H 8.11 (dd, *J* = 5.2, 1.5 Hz, 1H), 8.01 (d, *J* = 7.8 Hz, 1H), 7.75 (dd, *J* = 5.2, 2.2 Hz, 1H), 7.69 (d, *J* = 8.3 Hz, 1H), 7.65 (ddd, *J* = 8.2, 7.5, 0.9 Hz, 1H), 7.45 (t, *J* = 7.5 Hz, 1H);

^{13}C{^{1}H} NMR (126 MHz, $CDCl_3$) δ_C 157.2, 149.3 (d, *J* = 238 Hz), 139.6 (d, *J* = 13.1 Hz), 138.1 (d, *J* = 28.3 Hz), 136.3 (d, *J* = 5.7 Hz), 130.6, 124.1, 122.3 (d, *J* = 3.2 Hz), 122.2, 114.1 (d, *J* = 5.4 Hz), 113.0;

^{19}F{^{1}H} NMR (376 MHz, $CDCl_3$) δ_F –84.0;

ν_{max} (neat) 2917, 1632, 1570, 1425, 1316, 1254, 1193, 1085, 1073, 929, 832, 753 cm^{-1};

HRMS (ESI^+) calculated for $C_{11}H_6FNNaO$ $[M+Na]^+$ 210.0326, found 210.0327.

3-Fluorobenzofuro[2,3-*c*]pyridine (5g)

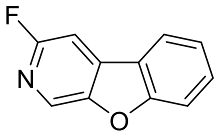

General procedure 3b (two-step procedure) was followed with 2,5-difluoropyridine (54 μL, 0.60 mmol, 1.2 equiv) and 2-bromophenyl acetate (**3a**) (108 mg, 0.50 mmol, 1.0 equiv). The crude benzofuropyridine was purified by column chromatography using 9:1 petroleum ether/EtOAc to give **5g** as a white solid (76 mg, 0.41 mmol, 88% yield over 2 steps).

R_f (petroleum ether/EtOAc 4:1) 0.38;

mp = 109.5–111 °C;

^{1}H NMR (500 MHz, $CDCl_3$) δ_H 8.49 (d, *J* = 0.6 Hz, 1H), 7.99 (d, *J* = 7.8 Hz, 1H), 7.66–7.60 (m, 2H), 7.43–7.39 (m, 2H);

^{13}C{^{1}H} NMR (126 MHz, $CDCl_3$) δ_C 159.1 (d, *J* = 231 Hz), 158.6, 151.0 (d, *J* = 3.2 Hz), 136.4 (d, *J* = 9.7 Hz), 131.0, 130.2 (d, *J* = 17.6 Hz), 123.7, 122.5, 122.1 (d, *J* = 4.5 Hz), 112.8, 100.2 (d, *J* = 43.3 Hz);

^{19}F{^{1}H} NMR (376 MHz, $CDCl_3$) δ_F –76.1;

ν_{max} (neat) 1626, 1584, 1443, 1404, 1165, 1150, 856, 758 cm^{-1};

HRMS (ESI^+) calculated for $C_{11}H_7FNO$ $[M+H]^+$ 188.0506, found 188.0505.

5-Fluorobenzofuro[2,3-*b*]pyridine (5h)

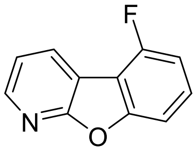

General procedure 2 was followed with 2-fluoropyridine (52 μL, 0.60 mmol, 1.2 equiv) and 2-bromo-3-fluorophenyl acetate (**3b**) (117 mg, 0.50 mmol, 1.0 equiv). The crude product was purified by column chromatography via 9:1 to 4:1 petroleum ether/EtOAc elution gradient to give **5h** as a white solid (76 mg, 0.41 mmol, 81% yield).

R_f (petroleum ether/EtOAc 9:1) 0.21;

mp = 92–93 °C;

^{1}H NMR (500 MHz, $CDCl_3$) δ_H 8.47 (dd, *J* = 4.9, 1.4 Hz, 1H), 8.36 (dd, *J* = 7.6, 1.7 Hz, 1H), 7.50–7.43 (m, 2H), 7.37 (dd, *J* = 7.6, 5.0 Hz, 1H), 7.08 (ddd, *J* = 9.0, 7.8, 0.9 Hz, 1H);

^{13}C{^{1}H} NMR (151 MHz, $CDCl_3$) δ_C 162.8, 158.0 (d, *J* = 252 Hz), 155.9 (d, *J* = 8.8 Hz), 146.8, 131.8 (d, *J* = 2.4 Hz), 129.1 (d, *J* = 8.2 Hz), 119.8, 114.6 (d, *J* = 1.9 Hz), 111.8 (d, *J* = 21.2 Hz), 109.8 (d, *J* = 18.6 Hz), 108.3 (d, *J* = 4.2 Hz);

^{19}F{^{1}H} NMR (376 MHz, $CDCl_3$) δ_F –117.2;

ν_{max} (neat) 2919, 1594, 1391, 1225, 1015, 796, 767, 722 cm^{-1};

HRMS (ESI^+) calculated for $C_{11}H_6FNNaO$ $[M+Na]^+$ 210.0326, found 210.0327.

6-Methylbenzofuro[2,3-*b*]pyridine (5i)

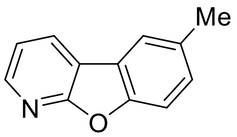

General procedure 2 was followed with 2-fluoropyridine (52 μL, 0.60 mmol, 1.2 equiv) and 2-bromo-4-methylphenyl acetate (**3c**) (115 mg, 0.50 mmol, 1.0 equiv). The crude product was purified by column chromatography via 9:1 to 4:1 petroleum ether/EtOAc elution gradient to give **5i** as a white solid (84 mg, 0.46 mmol, 92% yield).

R_f (petroleum ether/EtOAc 9:1) 0.16;

mp = 91–92 °C;

^{1}H NMR (500 MHz, $CDCl_3$) δ_H 8.42 (dd, *J* = 4.9, 1.3 Hz, 1H), 8.21 (dd, *J* = 7.5, 1.6 Hz, 1H), 7.73 (s, 1H), 7.51 (d, *J* = 8.4 Hz, 1H), 7.33–7.29 (m, 2H), 2.51 (s, 3H);

^{13}C{^{1}H} NMR (126 MHz, $CDCl_3$) δ_C 163.6, 153.0, 146.3, 133.1, 129.6, 129.5, 122.5, 121.3, 119.1, 117.1, 111.8, 21.5;

ν_{max} (neat) 2916, 1586, 1477, 1392, 1183, 796, 773 cm^{-1};

HRMS (ESI$^+$) calculated for $C_{12}H_9NNaO$ [M+Na]$^+$ 206.0576, found 206.0578.

6-Fluorobenzofuro[2,3-*b*]pyridine (5j)

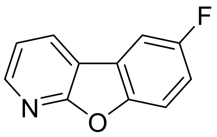

General procedure 2 was followed with 2-fluoropyridine (52 μL, 0.60 mmol, 1.2 equiv) and 2-bromo-4-fluorophenyl acetate (**3d**) (116 mg, 0.50 mmol, 1.0 equiv). The crude product was purified by column chromatography via 9:1 to 4:1 petroleum ether/EtOAc elution gradient to give **5j** as a white solid (66 mg, 0.35 mmol, 71% yield).

R_f (petroleum ether/EtOAc 9:1) 0.19;

mp = 110–112 °C;

^{1}H NMR (400 MHz, $CDCl_3$) δ_H 8.48 (dd, *J* = 4.9, 1.6 Hz, 1H), 8.25 (dd, *J* = 7.6, 1.6 Hz, 1H), 7.61 (dd, *J* = 7.9, 2.6 Hz, 1H), 7.59 (dd, *J* = 9.0, 4.0 Hz, 1H), 7.36 (dd, *J* = 7.5, 4.9 Hz, 1H), 7.25 (dd, *J* = 9.0, 2.6 Hz, 1H);

^{13}C{^{1}H} NMR (126 MHz, $CDCl_3$) δ_C 163.9, 159.4 (d, *J* = 241 Hz), 150.7, 147.1, 130.3, 123.4 (d, *J* = 10.2 Hz), 119.4, 117.0, 116.0 (d, *J* = 25.7 Hz), 113.3 (d, *J* = 9.1 Hz), 107.6 (d, *J* = 25.3 Hz);

^{19}F{^{1}H} NMR (376 MHz, $CDCl_3$) δ_F –118.9;

ν_{max} (neat) 1588, 1469, 1442, 1395, 1257, 1159, 858, 800, 772 cm^{-1};

HRMS (ESI$^+$) calculated for $C_{11}H_7FNO$ [M+H]$^+$ 188.0506, found 188.0505.

6-Chlorobenzofuro[2,3-*b*]pyridine (5k)

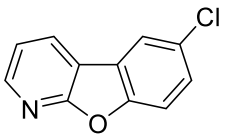

General procedure 2 was followed with 2-fluoropyridine (52 μL, 0.60 mmol, 1.2 equiv) and 2-bromo-4-chlorophenyl acetate (**3e**) (125 mg, 0.50 mmol, 1.0 equiv). The crude product was purified by column chromatography via 9:1 to 4:1 petroleum ether/EtOAc elution gradient to give **5k** as a white solid (65 mg, 0.32 mmol, 64% yield).

R$_f$ (petroleum ether/EtOAc 9:1) 0.15;

mp = 159–161 °C. Lit. 150–151 °C;[11]

^{1}H NMR (500 MHz, $CDCl_3$) δ_H 8.48 (d, *J* = 2.2 Hz, 1H), 8.23 (dd, *J* = 7.6, 1.4 Hz, 1H), 7.90 (d, *J* = 2.2 Hz, 1H), 7.55 (d, *J* = 8.7 Hz, 1H), 7.47 (dd, *J* = 8.7, 2.2 Hz, 1H), 7.35 (dd, *J* = 7.5, 4.9 Hz, 1H);

^{13}C{^{1}H} NMR (126 MHz, $CDCl_3$) δ_C 163.6, 152.9, 147.3, 130.2, 129.1, 128.6, 123.9, 121.3, 119.6, 116.3, 113.4.

The analytical data match those reported in the literature.[12]

6-Methoxybenzofuro[2,3-*b*]pyridine (5l)

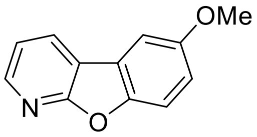

General procedure 2 was followed with 2-fluoropyridine (52 μL, 0.60 mmol, 1.2 equiv) and 2-bromo-4-methoxyphenyl acetate (**3f**) (123 mg, 0.50 mmol, 1.0 equiv). The crude product was purified by column chromatography via 9:1 to 4:1 petroleum ether/EtOAc elution gradient to give **5l** as a white solid (86 mg, 0.43 mmol, 86% yield).

R$_f$ (petroleum ether/EtOAc 9:1) 0.20;

mp = 77–79 °C;

^{1}H NMR (500 MHz, $CDCl_3$) δ_H 8.44 (br. s, 1H), 8.23 (dd, *J* = 7.6, 1.4 Hz, 1H), 7.53 (d, *J* = 9.0 Hz, 1H), 7.39 (d, *J* = 2.6 Hz, 1H), 7.31 (dd, *J* = 7.5, 5.0 Hz, 1H), 7.11 (dd, *J* = 9.0, 2.6 Hz, 1H), 3.91 (s, 3H);

^{13}C{^{1}H} NMR (126 MHz, $CDCl_3$) δ_C 163.8, 156.4, 149.3, 146.4, 129.8, 123.0, 119.0, 117.4, 116.5, 112.9, 104.5, 56.2;

ν$_{max}$ (neat) 2918, 1477, 1436, 1393, 1231, 1185, 1166, 1026, 794, 774 cm^{-1};

HRMS (ESI^+) calculated for $C_{12}H_9NNaO_2$ $[M+Na]^+$ 222.0525, found 222.0523.

Benzofuro[2,3-*b*]pyridin-6-ol (5m)

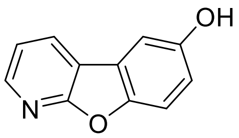

General procedure 2 was followed with 2-fluoropyridine (52 μL, 0.60 mmol, 1.2 equiv) and 2-bromo-1,4-phenylene diacetate (**3g**) (137 mg, 0.50 mmol, 1.0 equiv). The crude product was purified by column chromatography via 2:1 to 1:1 petroleum ether/EtOAc elution gradient to give **5m** as a white solid (47 mg, 0.25 mmol, 51% yield).

R_f (petroleum ether/EtOAc 1:1) 0.36;

mp = 233–234 °C. Lit. 238–240 ºC;[13]

^{1}H NMR (400 MHz, DMSO-*d6*) δ_H 9.57 (br. s, 1H), 8.54 (dd, *J* = 7.6, 1.7 Hz, 1H), 8.41 (dd, *J* = 4.9, 1.7 Hz, 1H), 7.55 (d, *J* = 8.8 Hz, 1H), 7.48 (d, *J* = 2.6 Hz, 1H), 7.44 (dd, *J* = 7.6, 4.9 Hz, 1H), 7.01 (dd, *J* = 8.8, 2.6 Hz, 1H);

^{13}C{^{1}H} NMR (126 MHz, DMSO-*d6*) δ_C 163.0, 153.9, 147.4, 146.2, 130.7, 122.6, 119.3, 116.8, 116.5, 112.3, 106.9.

The analytical data match those reported in the literature.[13]

Benzofuro[2,3-*b*]pyridine-6-carbonitrile (5n)

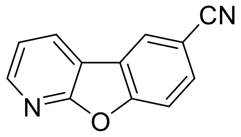

General procedure 2 was followed with 2-fluoropyridine (258 µL, 3.00 mmol, 1.2 equiv) and 2-bromo-4-cyanophenyl acetate (**3h**) (600 mg, 2.50 mmol, 1.0 equiv). The crude product was recrystallized from hot toluene to give **5n** as off-white needles (417 mg, 2.15 mmol, 86% yield).

mp = 247–248 °C;

^{1}H NMR (500 MHz, $CDCl_3$) δ_H 8.55 (dd, *J* = 4.9, 1.6 Hz, 1H), 8.34 (dd, *J* = 7.6, 1.6 Hz, 1H), 8.29 (d, *J* = 1.6 Hz, 1H), 7.82 (dd, *J* = 8.6, 1.6 Hz, 1H), 7.75 (d, *J* = 8.6 Hz, 1H), 7.44 (dd, *J* = 7.6, 4.9 Hz, 1H);

^{13}C{^{1}H} NMR (126 MHz, $CDCl_3$) δ_C 163.6, 156.5, 148.5, 132.1, 130.7, 126.2, 123.6, 120.3, 118.9, 115.5, 113.6, 107.6;

ν_{max} (neat) 3028, 2224, 1587, 1461, 1397, 1192, 1110, 919, 801, 794, 774, 742 cm^{-1};

HRMS (ESI^+) calculated for $C_{12}H_6N_2NaO$ $[M+Na]^+$ 217.0372, found 217.0373.

7-Fluorobenzofuro[2,3-*b*]pyridine (5o)

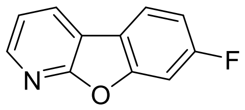

General procedure 2 was followed with 2-fluoropyridine (52 µL, 0.60 mmol, 1.2 equiv) and 2-bromo-4-fluorophenyl acetate (**3j**) (116 mg, 0.50 mmol, 1.0 equiv). The crude product was purified by column chromatography using 9:1 petroleum ether/EtOAc to give **5o** as a white solid (79 mg, 0.42 mmol, 84% yield).

R_f (petroleum ether/EtOAc 9:1) 0.22;

mp = 76–77 °C;

^{1}H NMR (500 MHz, $CDCl_3$) δ_H 8.42 (d, *J* = 4.2 Hz, 1H), 8.22 (dd, *J* = 7.6, 1.2 Hz, 1H), 7.89 (dd, *J* = 8.5, 5.4 Hz, 1H), 7.38–7.32 (m, 2H), 7.13 (td, *J* = 8.9, 2.1 Hz, 1H);

^{13}C{^{1}H} NMR (126 MHz, $CDCl_3$) δ_C 163.8 (d, *J* = 1.8 Hz), 163.0 (d, *J* = 247 Hz), 155.1 (d, *J* = 13.6 Hz), 146.1, 129.5, 122.2 (d, *J* = 10.2 Hz), 119.6, 118.9 (d, *J* = 2.3 Hz), 116.6, 111.7 (d, *J* = 23.9 Hz), 100.4 (d, *J* = 27.0 Hz);

^{19}**F{^{1}H} NMR** (376 MHz, $CDCl_3$) δ_F –110.2;

ν_{max} (neat) 2920, 1585, 1489, 1431, 1395, 1354, 1263, 1203, 1095, 948, 789, 772 cm^{-1};

HRMS (ESI$^+$) calculated for $C_{11}H_6FNNaO$ [M+Na]$^+$ 210.0326, found 210.0327.

8-Methoxybenzofuro[2,3-*b*]pyridine (5p)

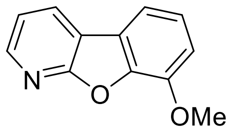

General procedure 2 was followed with 2-fluoropyridine (104 μL, 1.20 mmol, 1.2 equiv) and 2-bromo-6-methoxyphenyl acetate (**3k**) (245 mg, 1.00 mmol, 1.0 equiv). The crude product was purified by column chromatography using 4:1 petroleum ether/EtOAc to give **5p** as a white solid (102 mg, 0.51 mmol, 51% yield).

$\mathbf{R_f}$ (petroleum ether/EtOAc 4:1) 0.19;

mp = 93–94 °C;

^{1}**H NMR** (500 MHz, $CDCl_3$) δ_H 8.46 (dd, *J* = 4.9, 1.7 Hz, 1H), 8.25 (dd, *J* = 7.6, 1.7 Hz, 1H), 7.53 (dd, *J* = 7.8, 1.1 Hz, 1H), 7.35–7.28 (m, 2H), 7.06 (dd, *J* = 8.1, 1.0 Hz, 1H), 4.09 (s, 3H);

^{13}**C{^{1}H} NMR** (126 MHz, $CDCl_3$) δ_C 163.2, 146.7, 145.9, 143.9, 130.0, 124.14, 124.12, 119.3, 117.1, 113.4, 111.3, 56.7;

ν_{max} (neat) 1580, 1493, 1429, 1395, 1324, 1253, 1170, 1095, 1020, 845, 783, 732 cm^{-1};

EA Anal. calcd. for $C_{12}H_9NO_2$: C, 72.35; H, 4.55. Found: C, 72.20; H, 4.61.

Naphtho[1',2':4,5]furo[2,3-*b*]pyridine (5q)

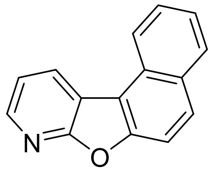

General procedure 2 was followed with 2-fluoropyridine (52 μL, 0.60 mmol, 1.2 equiv) and 1-bromonaphthalen-2-yl acetate (**3m**) (133 mg, 0.50 mmol, 1.0 equiv). The crude product was purified by column chromatography via 9:1 to 4:1 petroleum ether/EtOAc elution gradient to give **5q** as an off-white solid (49 mg, 0.22 mmol, 45% yield).

$\mathbf{R_f}$ (petroleum ether/EtOAc 9:1) 0.17;

mp = 120–122 °C;

^{1}**H NMR** (500 MHz, $CDCl_3$) δ_H 8.65 (dd, *J* = 7.7, 1.3 Hz, 1H), 8.51–8.45 (m, 2H), 8.03 (d, *J* = 8.2 Hz, 1H), 7.98 (d, *J* = 8.9 Hz, 1H), 7.81 (d, *J* = 8.9 Hz, 1H), 7.73 (t, *J* = 7.5 Hz, 1H), 7.57 (t, *J* = 7.5 Hz, 1H), 7.45 (dd, *J* = 7.6, 4.9 Hz, 1H);

^{13}**C{^{1}H} NMR** (126 MHz, $CDCl_3$) δ_C 162.8, 153.1, 145.3, 130.71, 130.66, 123.0, 129.7, 129.1, 127.8, 125.1, 123.4, 119.6, 117.7, 116.1, 112.9;

ν_{max} (neat) 2917, 1584, 1392, 1210, 994, 957, 801, 742 cm^{-1};

HRMS (ESI$^+$) calculated for $C_{15}H_9NNaO$ [M+Na]$^+$ 242.0576, found 242.0572.

6-Nitrobenzofuro[2,3-*b*]pyridine (5r)

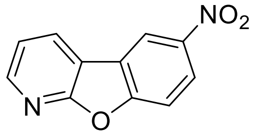

General procedure 3a was followed with 2-fluoropyridine (52 μL, 0.60 mmol, 1.2 equiv) and 2-bromo-4-nitrophenyl acetate (**3i**) (130 mg, 0.50 mmol, 1.0 equiv). The crude product was purified by recrystallization from hot toluene to give **5r** as a white solid (49 mg, 0.23 mmol, 46% yield).

mp = 274–276 °C. Lit. 276–278 ºC;[11]

^{1}H NMR (500 MHz, DMSO-*d6*) δ_H δ 9.28 (d, *J* = 2.4 Hz, 1H), 8.88 (dd, *J* = 7.6, 1.5 Hz, 1H), 8.58 (dd, *J* = 4.9, 1.5 Hz, 1H), 8.49 (dd, *J* = 9.1, 2.5 Hz, 1H), 8.02 (d, *J* = 9.1 Hz, 1H), 7.62 (dd, *J* = 7.6, 4.9 Hz, 1H);

^{13}C{^{1}H} NMR (126 MHz, DMSO-*d6*) δ_C 163.5, 156.9, 148.2, 143.9, 132.5, 124.2, 123.0, 120.8, 118.9, 115.6, 113.0;

ν_{max} (neat) 3096, 1595, 1518, 1340, 1193, 1116, 1079, 828, 772, 749 cm^{-1};

HRMS (ESI$^+$) calculated for $C_{11}H_6N_2NaO_3$ [M+Na]$^+$ 237.0271, found 237.0271.

8-Nitrobenzofuro[2,3-*b*]pyridine (5s)

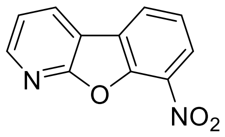

General procedure 3a was followed with 2-fluoropyridine (52 μL, 0.60 mmol, 1.2 equiv) and 2-bromo-6-nitrophenyl acetate (**3l**) (130 mg, 0.50 mmol, 1.0 equiv). The crude product was purified by recrystallization from hot toluene to give **5s** as an off-white solid (71 mg, 0.33 mmol, 66% yield).

mp = 222–223 °C;

^{1}H NMR (500 MHz, DMSO-*d6*) δ_H 8.77 (dd, *J* = 7.6, 1.6 Hz, 1H), 8.67 (dd, *J* = 7.7, 0.8 Hz, 1H), 8.59 (dd, *J* = 4.9, 1.6 Hz, 1H), 8.40 (dd, *J* = 8.2, 0.8 Hz, 1H), 7.68 (t, *J* = 8.0 Hz, 1H), 7.63 (dd, *J* = 7.6, 4.9 Hz, 1H);

^{13}C{^{1}H} NMR (126 MHz, DMSO-*d6)* δ_C 163.0, 148.1, 146.3, 133.6, 132.0, 129.2, 125.9, 124.2, 124.0, 120.9, 114.7;

ν_{max} (neat) 1593, 1523, 1396, 1341, 1189, 911, 843, 802, 774, 736 cm^{-1};

HRMS (ESI$^+$) calculated for $C_{11}H_6N_2NaO_3$ [M+Na]$^+$ 237.0271, found 237.0268.

Benzo[4,5]thieno[2,3-*b*]pyridine (5t)

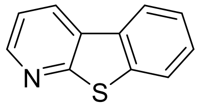

General procedure 2 was followed with 2-fluoropyridine (52 μL, 0.60 mmol, 1.2 equiv) and *S*-(2-bromophenyl) thioacetate (**3n**) (116 mg, 0.50 mmol, 1.0 equiv). The crude product was purified by column chromatography using 9:1 petroleum ether/EtOAc to give **5t** as a white solid (24 mg, 0.13 mmol, 26% yield).

R_f (petroleum ether/EtOAc 9:1) 0.18;

mp = 71–73 °C. Lit. 73–74 ºC;[14]

^{1}H NMR (500 MHz, $CDCl_3$) δ_H 8.64 (dd, *J* = 4.6, 1.5 Hz, 1H), 8.36 (dd, *J* = 7.9, 1.5 Hz, 1H), 8.12 (dd, *J* = 6.9, 1.5 Hz, 1H), 7.88 (dd, *J* = 7.2, 1.2 Hz, 1H), 7.50 (app pd, *J* = 7.1, 1.2 Hz, 2H), 7.38 (dd, *J* = 7.9, 4.7 Hz, 1H);

^{13}C{^{1}H} NMR (126 MHz, $CDCl_3$) δ_C 162.1, 148.5, 138.2, 133.0, 129.6, 129.0, 127.7, 125.0, 123.2, 122.2, 119.6;

ν_{max} (neat) 2917, 1546, 1437, 1373, 1257, 1068, 751, 730 cm^{-1};

HRMS (ESI^+) calculated for $C_{11}H_8NS$ $[M+H]^+$ 186.0372, found 186.0373.

2.5 Preparation of dibenzofurans

Dibenzo[*b*,*d*]furan (6a)

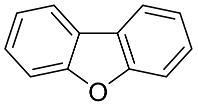

General procedure 4 was followed with fluorobenzene (56 μL, 0.60 mmol, 1.2 equiv) and 2-bromophenyl acetate (**3a**) (108 mg, 0.50 mmol, 1.0 equiv). The crude product was purified by column chromatography using 95:5 petroleum ether/EtOAc to give **6a** as a waxy white solid (66 mg, 0.39 mmol, 78% yield).

R_f (petroleum ether/EtOAc 9:1) 0.59;

mp = 78–80 °C. Lit. 83–84 ºC;[15]

^{1}H NMR (400 MHz, $CDCl_3$) δ_H 7.97 (d, *J* = 7.7 Hz, 2H), 7.60 (d, *J* = 8.2 Hz, 2H), 7.48 (t, *J* = 7.7 Hz, 2H), 7.36 (t, *J* = 7.5 Hz, 2H);

^{13}C{^{1}H} NMR (126 MHz, $CDCl_3$) δ_C 156.3, 127.3, 124.4, 122.8, 120.8, 111.8.

The analytical data match those reported in the literature.[15]

2,3-Dimethyldibenzo[*b*,*d*]furan (6b)

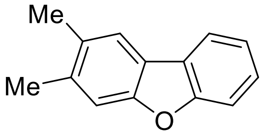

General procedure 4 was followed with 3,4-dimethylfluorobenzene (62 mg, 0.60 mmol, 1.2 equiv) and 2-bromophenyl acetate (**3a**) (108 mg, 0.50 mmol, 1.0 equiv). The crude product was purified by column chromatography using 99:1 petroleum ether/EtOAc to give **6b** as a white solid (49 mg, 0.25 mmol, 50% yield).

R_f (petroleum ether/EtOAc 98:2) 0.37;

mp = 81–83 °C. Lit. 90–91 ºC;[16]

^{1}H NMR (500 MHz, $CDCl_3$) δ_H 7.89 (d, *J* = 7.6 Hz, 1H), 7.70 (s, 1H), 7.54 (d, *J* = 8.2 Hz, 1H), 7.41 (ddd, *J* = 8.5, 2.4, 1.3 Hz, 1H), 7.36 (s, 1H), 7.31 (t, *J* = 7.5 Hz, 1H), 2.42 (s, 3H), 2.41 (s, 3H);

^{13}C{^{1}H} NMR (126 MHz, $CDCl_3$) δ_C 156.3, 155.2, 136.6, 131.3, 126.5, 124.6, 122.6, 122.0, 121.0, 120.4, 112.3, 111.7, 20.9, 20.1.

The analytical data match those reported in the literature.[16]

1-Methoxydibenzo[*b*,*d*]furan (6c)

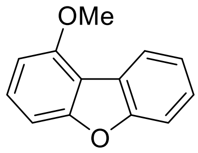

General procedure 4 was followed with 3-fluoroanisole (69 μL, 0.60 mmol, 1.2 equiv) and 2-bromophenyl acetate (**3a**) (108 mg, 0.50 mmol, 1.0 equiv). The crude product was purified by column chromatography using 97.5:2.5 petroleum ether/EtOAc to give **6c** as an off-white solid (60 mg, 0.30 mmol, 61% yield).

R$_f$ (petroleum ether/EtOAc 9:1) 0.43;

mp = 60–62 °C. Lit. 57–58 ºC;[17]

^{1}H NMR (500 MHz, $CDCl_3$) δ_H 8.14 (d, J = 7.6 Hz, 1H), 7.55 (d, J = 8.2 Hz, 1H), 7.44–7.37 (m, 2H), 7.34 (t, J = 7.6 Hz, 1H), 7.20 (d, J = 8.2 Hz, 1H), 6.80 (d, J = 8.2 Hz, 1H), 4.06 (s, 3H);

^{13}C{^{1}H} NMR (126 MHz, $CDCl_3$) δ_C 157.4, 156.0, 155.6, 128.0, 126.3, 123.7, 123.0, 122.9, 113.7, 111.1, 104.5, 103.9, 55.8.

The analytical data match those reported in the literature.[17]

2-Methoxydibenzo[*b*,*d*]furan (6d)

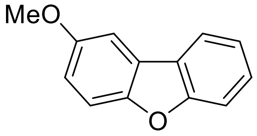

General procedure 4 was followed with 4-fluoroanisole (68 μL, 0.60 mmol, 1.2 equiv) and 2-bromophenyl acetate (**3a**) (108 mg, 0.50 mmol, 1.0 equiv). The crude product was purified by column chromatography using 97.5:2.5 petroleum ether/EtOAc to give **6d** as a pale-yellow viscous oil (36 mg, 0.18 mmol, 36% yield).

R$_f$ (petroleum ether/EtOAc 9:1) 0.42;

^{1}H NMR (500 MHz, $CDCl_3$) δ_H 7.92 (d, J = 7.7 Hz, 1H), 7.55 (d, J = 8.2 Hz, 1H), 7.48–7.42 (m, 3H), 7.33 (ddd, J = 8.0, 7.3, 0.6 Hz, 1H), 7.05 (dd, J = 8.9, 2.6 Hz, 1H), 3.92 (s, 3H);

^{13}C{^{1}H} NMR (126 MHz, $CDCl_3$) δ_C 157.1, 156.0, 151.1, 127.3, 124.8, 124.6, 122.6, 120.7, 115.3, 112.3, 111.9, 103.9, 56.2.

The analytical data match those reported in the literature.[15]

2-Fluorodibenzo[*b*,*d*]furan (6e)

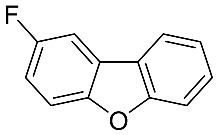

General procedure 4 was followed with 1,4-difluorobenzene (62 μL, 0.60 mmol, 1.2 equiv) and 2-bromophenyl acetate (**3a**) (108 mg, 0.50 mmol, 1.0 equiv). The crude product was purified by column chromatography using 97.5:2.5 petroleum ether/EtOAc to give **6e** as a white solid (50 mg, 0.27 mmol, 54% yield).

R$_f$ (petroleum ether/EtOAc 9:1) 0.53;

mp = 88–89 °C. Lit. 89 ºC;[15]

^{1}H NMR (500 MHz, $CDCl_3$) δ_H 7.91 (d, J = 7.7 Hz, 1H), 7.61 (dd, J = 8.1, 2.6 Hz, 1H), 7.57 (d, J = 8.3 Hz, 1H), 7.53–7.45 (m, 2H), 7.35 (t, J = 7.5 Hz, 1H), 7.17 (td, J = 9.0, 2.7 Hz, 1H);

^{13}C{^{1}H} NMR (126 MHz, $CDCl_3$) δ_C 159.1 (d, J = 239 Hz), 157.3, 152.3, 127.9, 125.3 (d, J = 10.2 Hz), 124.1 (d, J = 3.6 Hz), 122.9, 121.0, 114.6 (d, J = 25.9 Hz), 112.4 (d, J = 9.3 Hz), 112.0, 106.8 (d, J = 25.1 Hz);

^{19}F{^{1}H} NMR (376 MHz, $CDCl_3$) δ_F –120.5.

The analytical data match those reported in the literature.[15]

4-Fluorodibenzo[*b*,*d*]furan (6f)

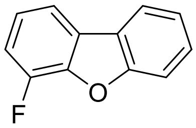

General procedure 4 was followed with 1,2-difluorobenzene (59 μL, 0.60 mmol, 1.2 equiv) and 2-bromophenyl acetate (**3a**) (108 mg, 0.50 mmol, 1.0 equiv). The crude product was purified by column chromatography using petroleum ether to give **6f** as a white solid (61 mg, 0.33 mmol, 66% yield).

R_f (petroleum ether) 0.28;

mp = 50–52 °C. Lit. 50–51 ºC;[18]

^{1}H NMR (500 MHz, $CDCl_3$) δ_H 7.95 (d, J = 7.6 Hz, 1H), 7.71 (d, J = 7.6 Hz, 1H), 7.63 (d, J = 8.3 Hz, 1H), 7.50 (ddd, J = 8.3, 7.4, 1.1 Hz, 1H), 7.38 (t, J = 7.6 Hz, 1H), 7.27 (td, J = 7.9, 3.9 Hz, 1H), 7.21 (ddd, J = 10.0, 8.2, 0.7 Hz, 1H);

^{13}C{^{1}H} NMR (126 MHz, $CDCl_3$) δ_C 156.6, 148.4 (d, J = 249 Hz), 143.2 (d, J = 11.2 Hz), 128.0, 127.9 (d, J = 3.1 Hz), 124.0 (d, J = 2.6 Hz), 123.5 (d, J = 5.8 Hz), 123.4, 121.1, 116.3 (d, J = 3.9 Hz), 113.7 (d, J = 16.3 Hz), 112.2;

^{19}F{^{1}H} NMR (376 MHz, $CDCl_3$) δ_F –136.7.

The analytical data match those reported in the literature.[18]

2-Methyldibenzo[*b*,*d*]furan (6g)

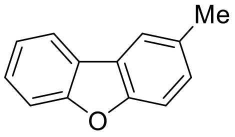

General procedure 4 was followed with fluorobenzene (56 μL, 0.60 mmol, 1.2 equiv) and 2-bromo-4-methylphenyl acetate (**3c**) (115 mg, 0.50 mmol, 1.0 equiv). The crude product was purified by column chromatography using 99:1 petroleum ether/EtOAc to give **6g** as a white solid (52 mg, 0.29 mmol, 57% yield).

R_f (petroleum ether/EtOAc 9:1) 0.62;

mp = 46.5–47.5 °C. Lit. 43–45 ºC;[15]

^{1}H NMR (500 MHz, $CDCl_3$) δ_H 7.93 (d, J = 7.7 Hz, 1H), 7.76 (s, 1H), 7.56 (d, J = 8.2 Hz, 1H), 7.49–7.42 (m, 2H), 7.33 (t, J = 7.5 Hz, 1H), 7.27 (dd, J = 8.5, 1.2 Hz, 1H), 2.52 (s, 3H);

^{13}C{^{1}H} NMR (126 MHz, $CDCl_3$) δ_C 156.6, 154.7, 132.3, 128.4, 127.1, 124.4, 124.3, 122.7, 120.8, 120.7, 111.8, 111.3, 21.5.

The analytical data match those reported in the literature.[15]

2-Methoxydibenzo[*b*,*d*]furan (6d')

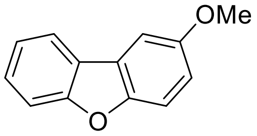

General procedure 4 was followed with fluorobenzene (56 μL, 0.60 mmol, 1.2 equiv) and 2-bromo-4-methoxyphenyl acetate (**3f**) (108 mg, 0.50 mmol, 1.0 equiv). The crude product was purified by column chromatography using 97.5:2.5 petroleum ether/EtOAc to give **6d'** as a pale-yellow oil (64 mg, 0.32 mmol, 65% yield). The analytical data match those reported for **6d**.

2-Fluorodibenzo[*b*,*d*]furan (6e')

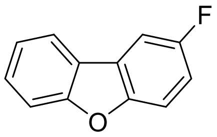

General procedure 4 was followed with fluorobenzene (56 μL, 0.60 mmol, 1.2 equiv) and 2-bromo-4-fluorophenyl acetate (**3d**) (116 mg, 0.50 mmol, 1.0 equiv). The crude product was purified by column chromatography using 97.5:2.5 petroleum ether/EtOAc to give **6e'** as an off-white solid (57 mg, 0.31 mmol, 61% yield). The analytical data match those reported for **6e**.

Dibenzo[*b*,*d*]furan-2-carbonitrile (6h)

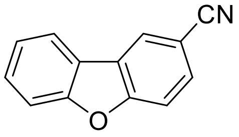

General procedure 4 was followed with fluorobenzene (56 μL, 0.60 mmol, 1.2 equiv) and 2-bromo-4-cyanophenyl acetate (**3h**) (120 mg, 0.50 mmol, 1.0 equiv). The crude product was purified by column chromatography via 95:5 to 9:1 petroleum ether/EtOAc elution gradient to give **6h** as a white solid (50 mg, 0.25 mmol, 50% yield).

R_f (petroleum ether/EtOAc 9:1) 0.31;

mp = 140–141 °C. Lit. 139–141 ºC;[19]

^{1}H NMR (500 MHz, $CDCl_3$) δ_H 8.23 (s, 1H), 7.95 (d, *J* = 7.7 Hz, 1H), 7.72 (d, *J* = 8.5 Hz, 1H), 7.64–7.59 (m, 2H), 7.55 (t, *J* = 7.7 Hz, 1H), 7.41 (t, *J* = 7.5 Hz, 1H);

^{13}C{^{1}H} NMR (126 MHz, $CDCl_3$) δ_C 158.0, 156.8, 130.9, 128.9, 125.5, 125.3, 123.9, 122.6, 121.2, 119.3, 112.9, 112.2, 106.7.

The analytical data match those reported in the literature.[19]

1-Fluorodibenzo[*b*,*d*]furan (6i)

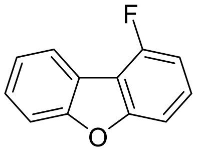

General procedure 4 was followed with fluorobenzene (56 μL, 0.60 mmol, 1.2 equiv) and 2-bromo-3-fluorophenyl acetate (**3b**) (116 mg, 0.50 mmol, 1.0 equiv). The crude product was purified by column chromatography using 99:1 petroleum ether/EtOAc to give **6i** as a white solid (36 mg, 0.19 mmol, 39% yield).

R_f (petroleum ether/EtOAc 9:1) 0.54;

mp = 54–56 °C;

^{1}H NMR (500 MHz, $CDCl_3$) δ_H 8.07 (d, *J* = 7.7 Hz, 1H), 7.58 (d, *J* = 8.3 Hz, 1H), 7.49 (td, *J* = 7.9, 1.0 Hz, 1H), 7.41–7.36 (m, 3H), 7.04 (ddd, *J* = 9.0, 7.6, 1.2 Hz, 1H);

^{13}C{^{1}H} NMR (126 MHz, $CDCl_3$) δ_C 157.9 (d, J = 251 Hz), 157.8 (d, J = 9.5 Hz), 155.8, 127.8 (d, J = 8.1 Hz), 127.5, 123.4, 123.0 (d, J = 2.6 Hz), 121.8 (d, J = 2.3 Hz), 113.3 (d, J = 21.0 Hz), 111.6, 109.1 (d, J = 18.8 Hz), 107.8 (d, J = 4.0 Hz);

^{19}F{^{1}H} NMR (376 MHz, $CDCl_3$) δ_F –118.2;

ν_{max} (neat) 2920, 1601, 1447, 1432, 1260, 1227, 1192, 1025, 844, 781, 746, 713 cm^{-1};

HRMS (ESI^+) calculated for $C_{12}H_8FO$ $[M+H]^+$ 187.0554, found 187.0554.

2.6 Extended optimization table

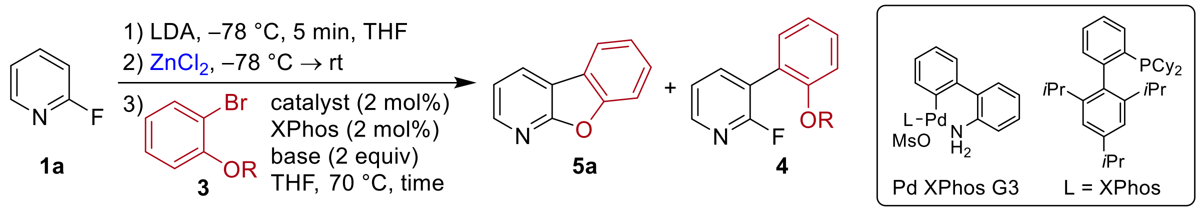

Entry	R	Catalyst	Base	Time	Yield **5a**[b]	Yield **4**[b]
1	Ac	Pd XPhos G3	KO*t*Bu	o/n	96%[c]	–
2[d]	Ac	Pd XPhos G3	KO*t*Bu	o/n	81%	–
3[e]	Ac	Pd XPhos G3	KO*t*Bu	o/n	74%[c]	–
4[f]	H	Pd XPhos G3	KO*t*Bu	o/n	0%	–
5	Ac	$Pd(PPh_3)_4$	KO*t*Bu	o/n	50%	–
6	Ac	$Pd(OAc)_2$	KO*t*Bu	o/n	70%	–
7[d]	Ac	$[PdCl(C_3H_5)]_2$	KO*t*Bu	o/n	84%	–
8	Ac	–	KO*t*Bu	o/n	0%	–
9[e]	Ac	$Pd(OAc)_2$	KO*t*Bu	o/n	11%	–
10	Ac	Pd XPhos G3	–	20 min	–	84%[c]
11	Ac	Pd XPhos G3	–	o/n	51%	31%
12[g]	Ac	Pd XPhos G3	KO*t*Bu	o/n	52%	16%
13	Ac	Pd XPhos G3	KO*t*Bu	20 min	11%	28%
14	Ac	Pd XPhos G3	NaHMDS	o/n	86%	–
15	Ac	Pd XPhos G3	Cs_2CO_3	o/n	22%	30%
16	Me	Pd XPhos G3	KO*t*Bu	o/n	–	94%[c]
17	TMS	Pd XPhos G3	KO*t*Bu	o/n	43%[c]	36%[c]
18	TBS	Pd XPhos G3	KO*t*Bu	o/n	–	53%[c]
19[h]	Ac	$Pd(OAc)_2$	KO*t*Bu	o/n	77%	–
20[d,e]	Ac	$[Pd(\mu\text{-Br})P\textit{t}Bu_3]_2$	KO*t*Bu	o/n	49%	–

[a]0.5 mmol scale; reaction conditions: 1) **1a** (1.2 equiv), LDA (1.3 equiv), THF (0.25 M), –25 °C, 5 min; 2) $ZnCl_2$ (1.3 equiv), then –25 °C to rt; 3) 2-bromophenol derivative **3** (1.0 equiv), catalyst (2.0 mol%) and XPhos (2.0 mol%) in THF (0.5 M), base (2.0 equiv), 70 °C. [b]Determined by ^{1}H NMR spectroscopy using 1,3,5-trimethoxybenzene as internal standard. [c]Isolated yield after column chromatography. [d]1 mol% catalyst loading. [e]No additional XPhos ligand. [f]2.3 equiv of LDA used. [g]1.0 equiv of base. [h]CPhos (2.0 mol%) as ligand.

2.7 Low-yielding and unsuccessful substrates

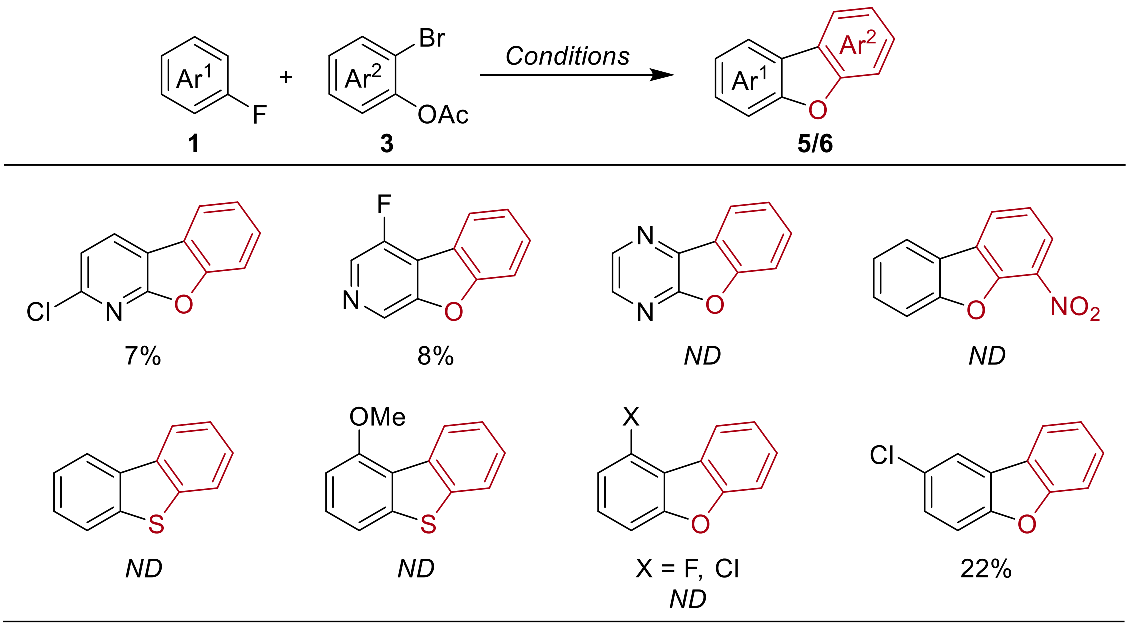

ND - not determined

3. References

(1) Bruno, N. C.; Tudge, M. T.; Buchwald, S. L. *Chem. Sci.* **2013**, *4*, 916–920.

(2) Burchat, A. F.; Chong, J. M.; Nielsen, N. *J. Organomet. Chem.* **1997**, *542*, 281–283.

(3) Pajtás, D.; Patonay, T.; Kónya, K. *Synthesis* **2016**, *48*, 97–102.

(4) Chyan, W.; Kilgore, H. R.; Gold, B.; Raines, R. T. *J. Org. Chem.* **2017**, *82*, 4297–4304.

(5) Swamy, N. K.; Yazici, A.; Pyne, S. G. *J. Org. Chem.* **2010**, *75*, 3412–3419.

(6) Liu, Y.; Zhou, C.-J.; Li, Q.; Wang, H. *Org. Biomol. Chem.* **2016**, *14*, 10362–10365.

(7) Cook, A. K.; Emmert, M. H.; Sanford, M. S. *Org. Lett.* **2013**, *15*, 5428–5431.

(8) Mabic, S.; Vaysse, L.; Benezra, C.; Lepoittevin, J.-P. *Synthesis* **1999**, 1127–1134.

(9) Satkar, Y.; Ramadoss, V.; Nahide, P. D.; García-Medina, E.; Juárez-Ornelas, K. A.; Alonso-Castro, A. J.; Chávez-Rivera, R.; Jiménez-Halla, J. O. C.; Solorio-Alvarado, C. R. *RSC Adv.* **2018**, *8*, 17806–17812.

(10) Yue, W. S.; Li, J. J. *Org. Lett.* **2002**, *4*, 2201–2203.

(11) Abramovitch, R. A.; Inbasekaran, M. N. *Tetrahedron Lett.* **1977**, *18*, 1109–1112.

(12) Liu, J.; Fitzgerald, A. E.; Mani, N. S. *J. Org. Chem.* **2008**, *73*, 2951–2954.

(13) Singh, R.; Horsten, T.; Prakash, R.; Dey, S.; Dehaen, W. *Beilstein J. Org. Chem.* **2021**, *17*, 977–982.

(14) Roques, B. P.; Prange, T.; Oberlin, R. *Org. Magn. Reason.* **1977**, *9*, 185–192.

(15) Nervig, C. S.; Waller, P. J.; Kalyani, D. *Org. Lett.* **2012**, *14*, 4838–4841.

(16) Liu, Z.; Larock, R. C. *Tetrahedron* **2007**, *63*, 347–355.

(17) Wei, Y.; Yoshikai, N. *Org. Lett.* **2011**, *13*, 5504–5507.

(18) Ichiishi, N.; Canty, A. J.; Yates, B. F.; Sanford, M. S. *Org. Lett.* **2013**, *15*, 5134–5137.

(19) Solórzano, P. C.; Brigante, F.; Pierini, A. B.; Jimenez, L. B. *J. Org. Chem.* **2018**, *83*, 7867–7877.

4. ^{1}H, ^{13}C and ^{19}F NMR spectra

^{1}H NMR (400 MHz, $CDCl_3$)

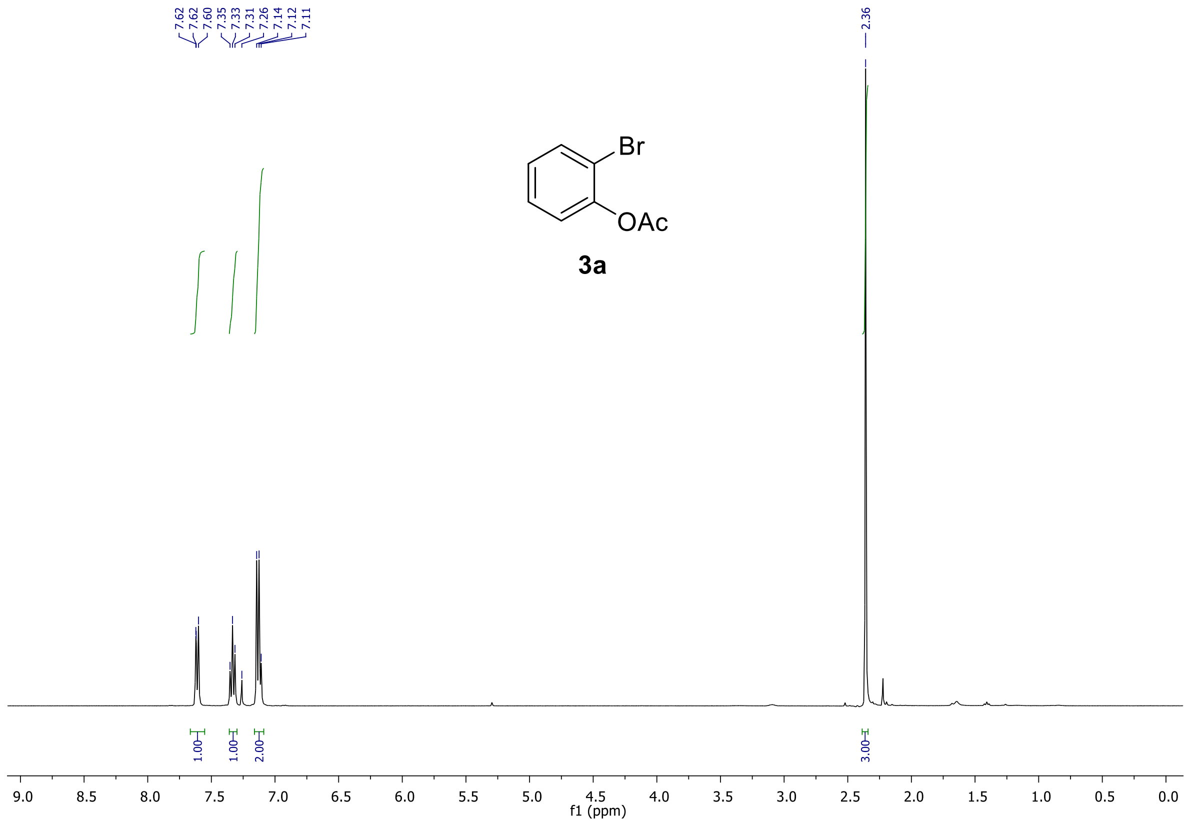

^{13}C{^{1}H} NMR (101 MHz, $CDCl_3$)

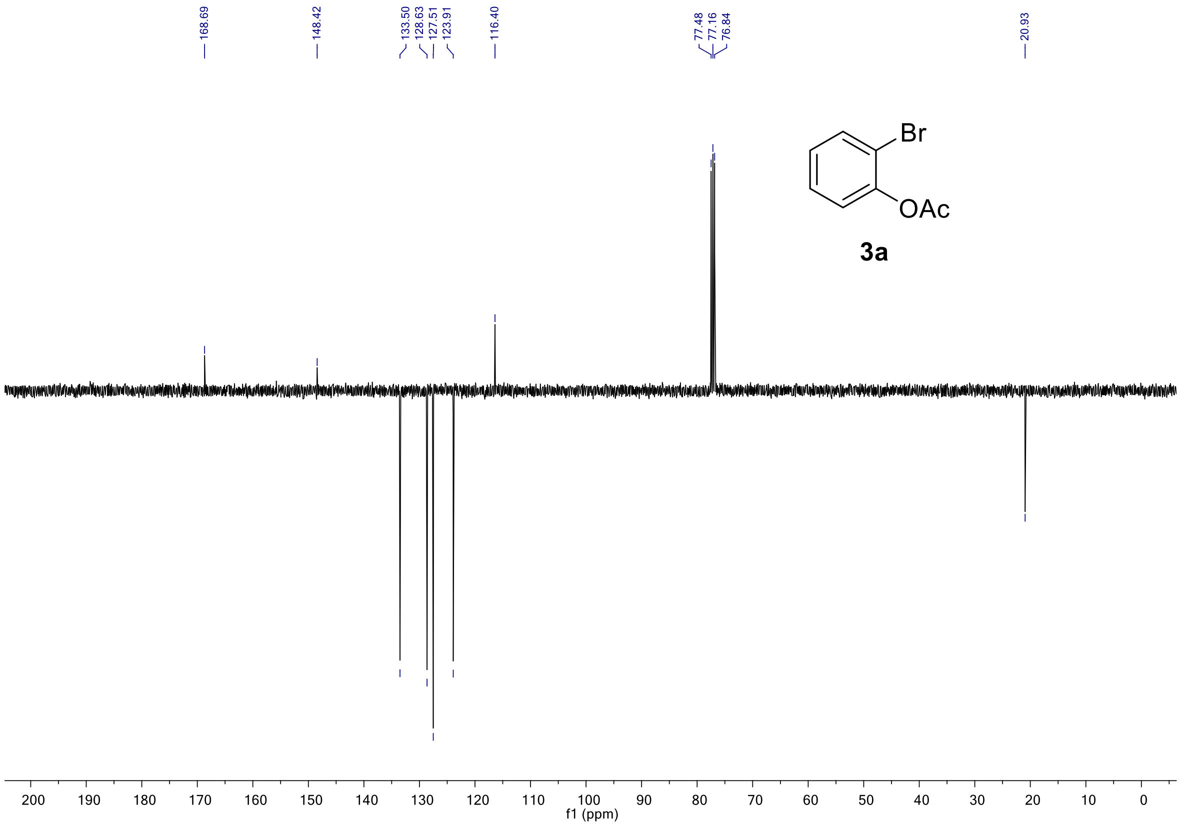

^1H NMR (500 MHz, $CDCl_3$)

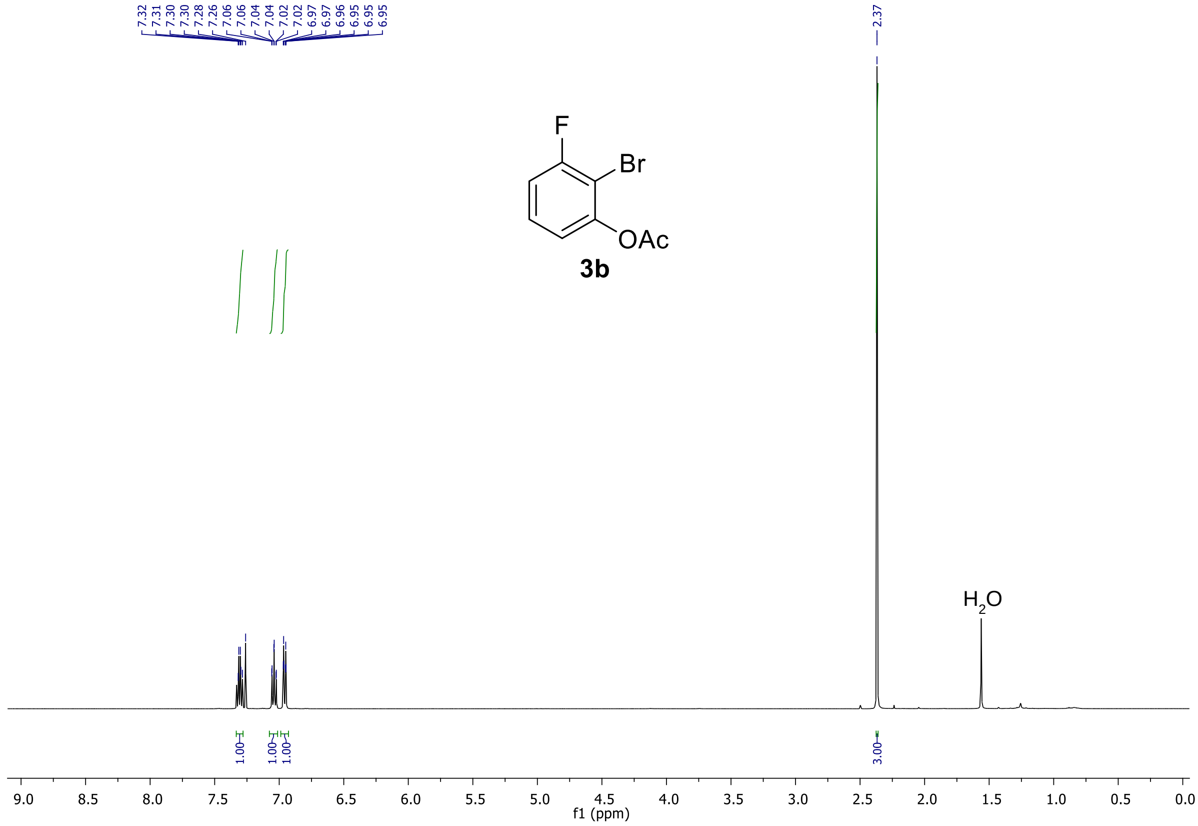

$^{13}C\{^1H\}$ NMR (126 MHz, $CDCl_3$)

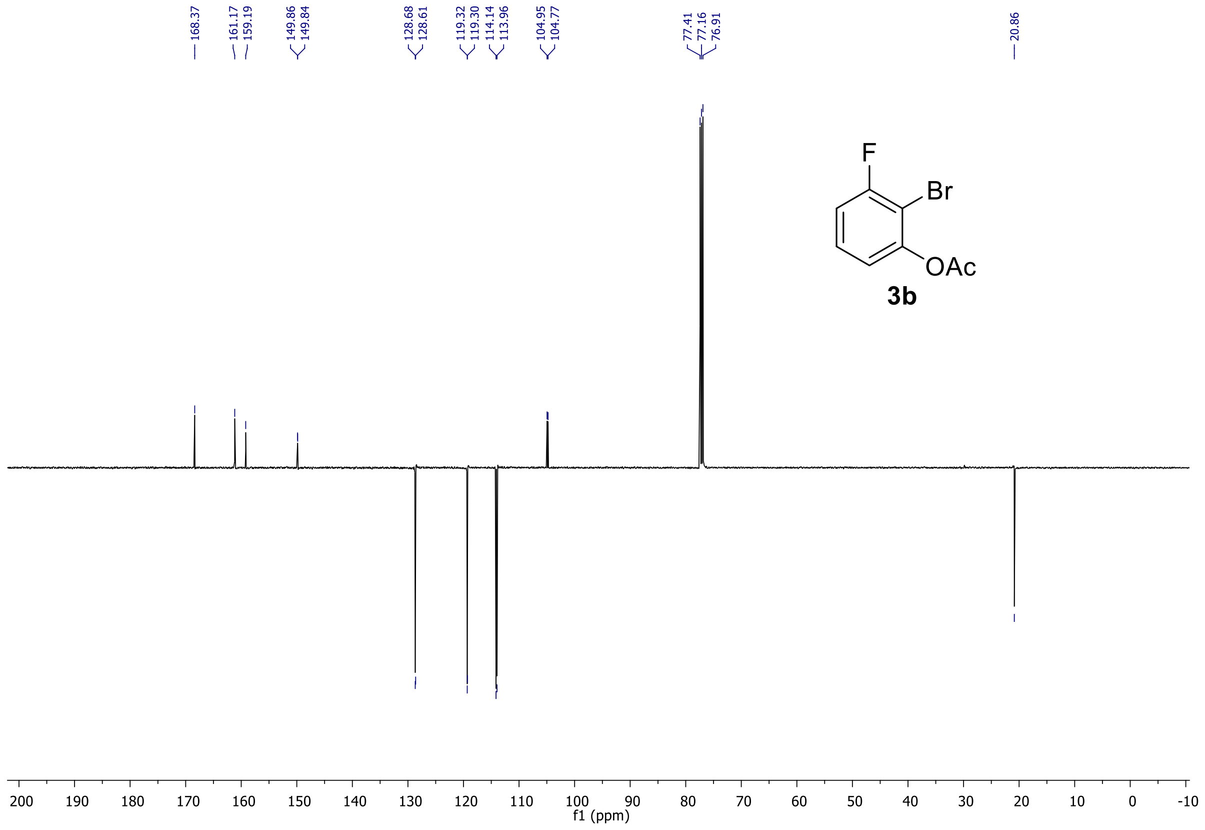

$^{19}F\{^{1}H\}$ NMR (376 MHz, $CDCl_3$)

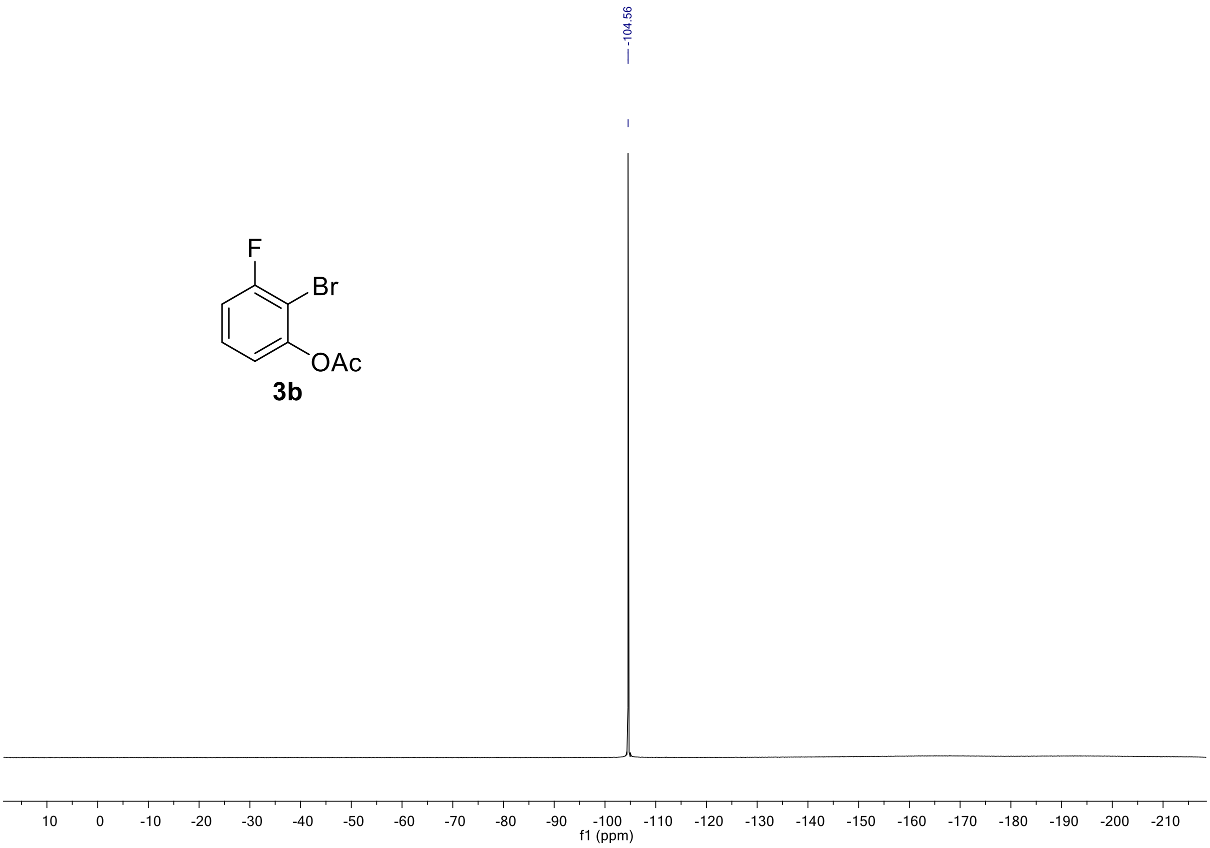

^{1}H NMR (500 MHz, $CDCl_3$)

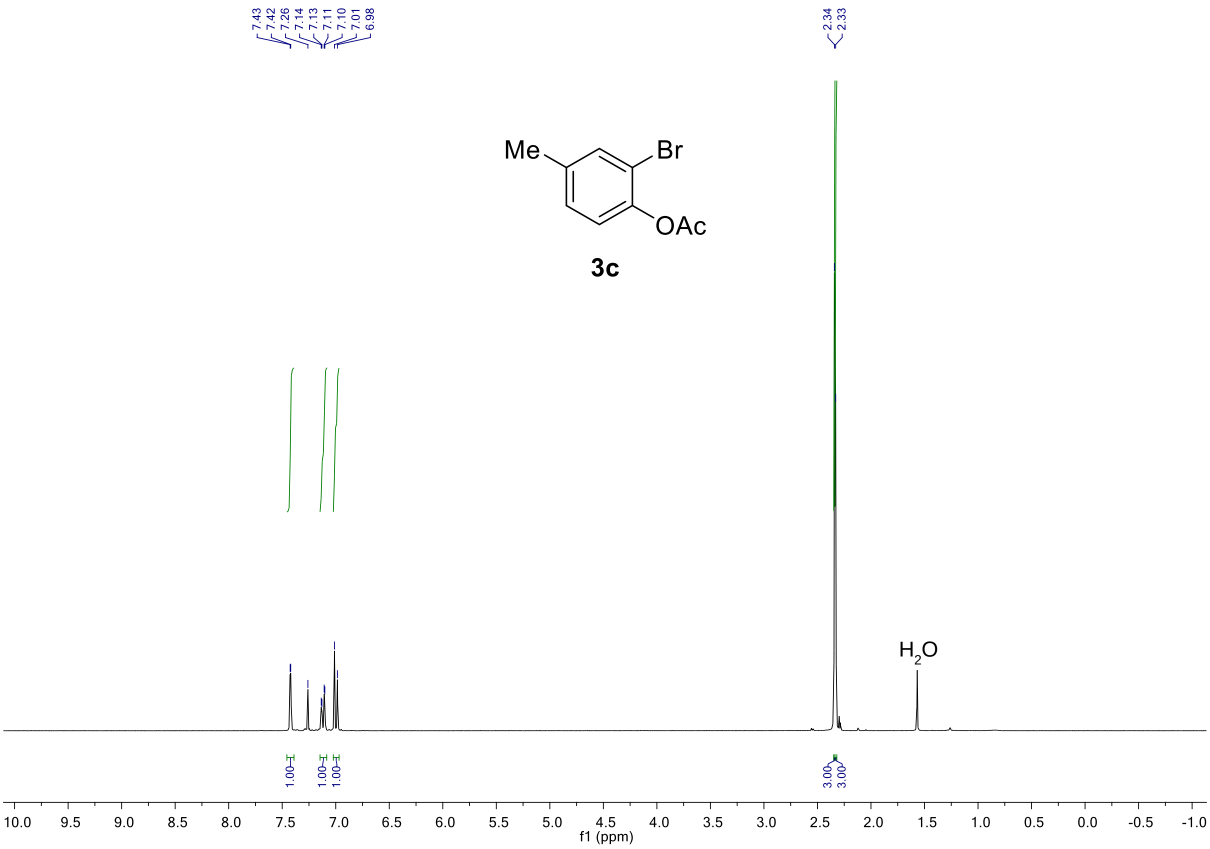

$^{13}C\{^{1}H\}$ NMR (126 MHz, $CDCl_3$)

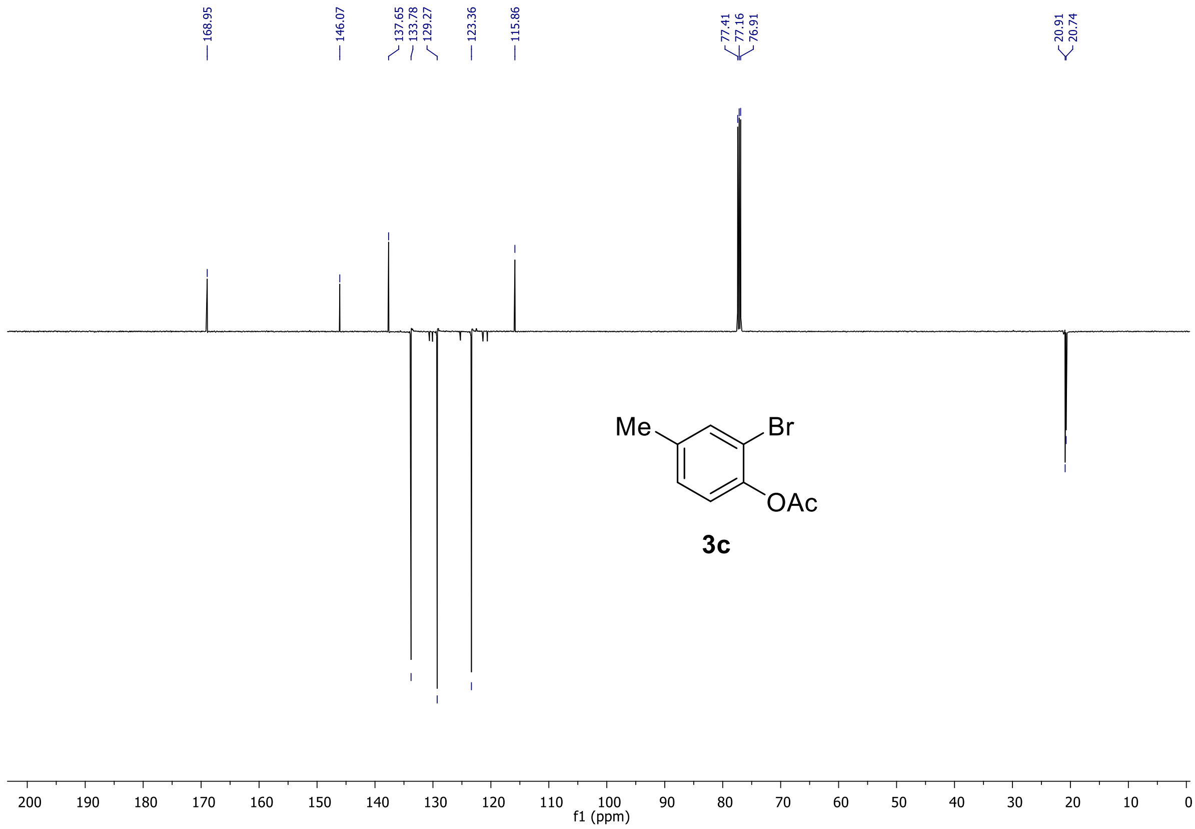

^{1}H NMR (500 MHz, $CDCl_3$)

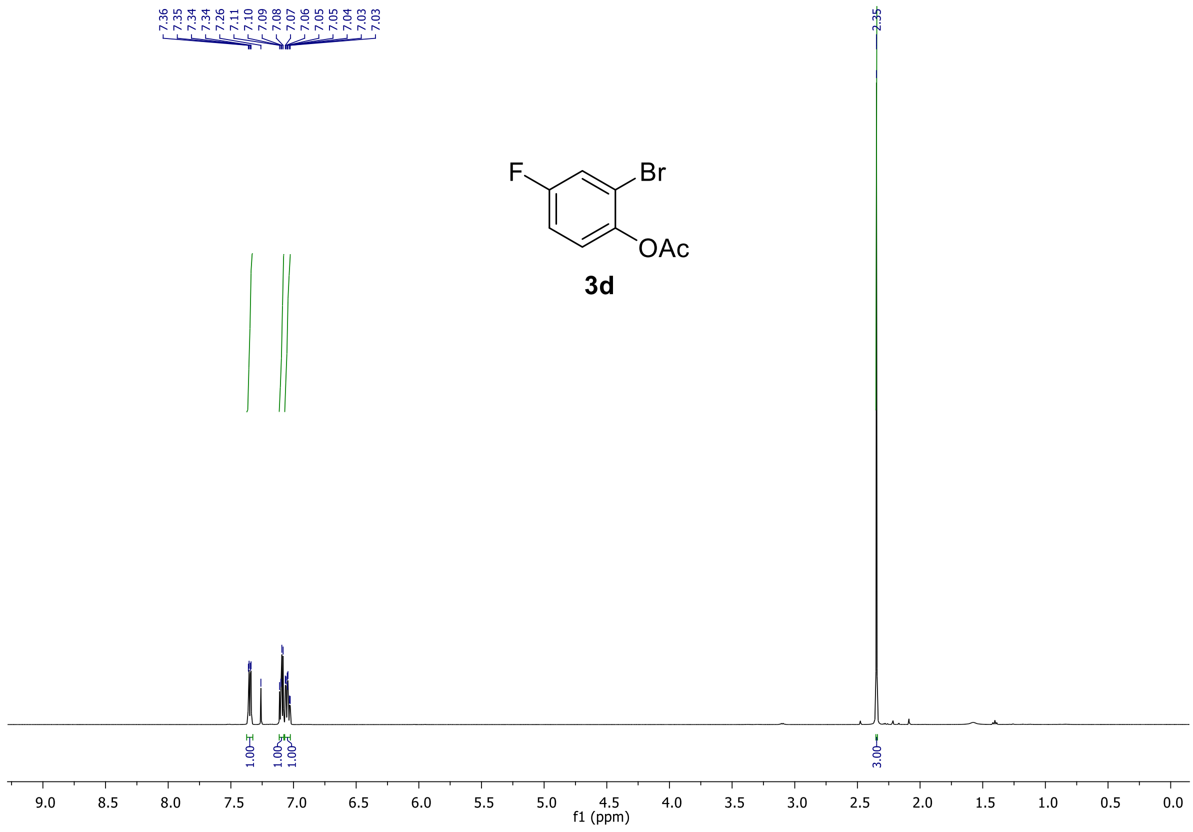

$^{13}C\{^1H\}$ NMR (126 MHz, $CDCl_3$)

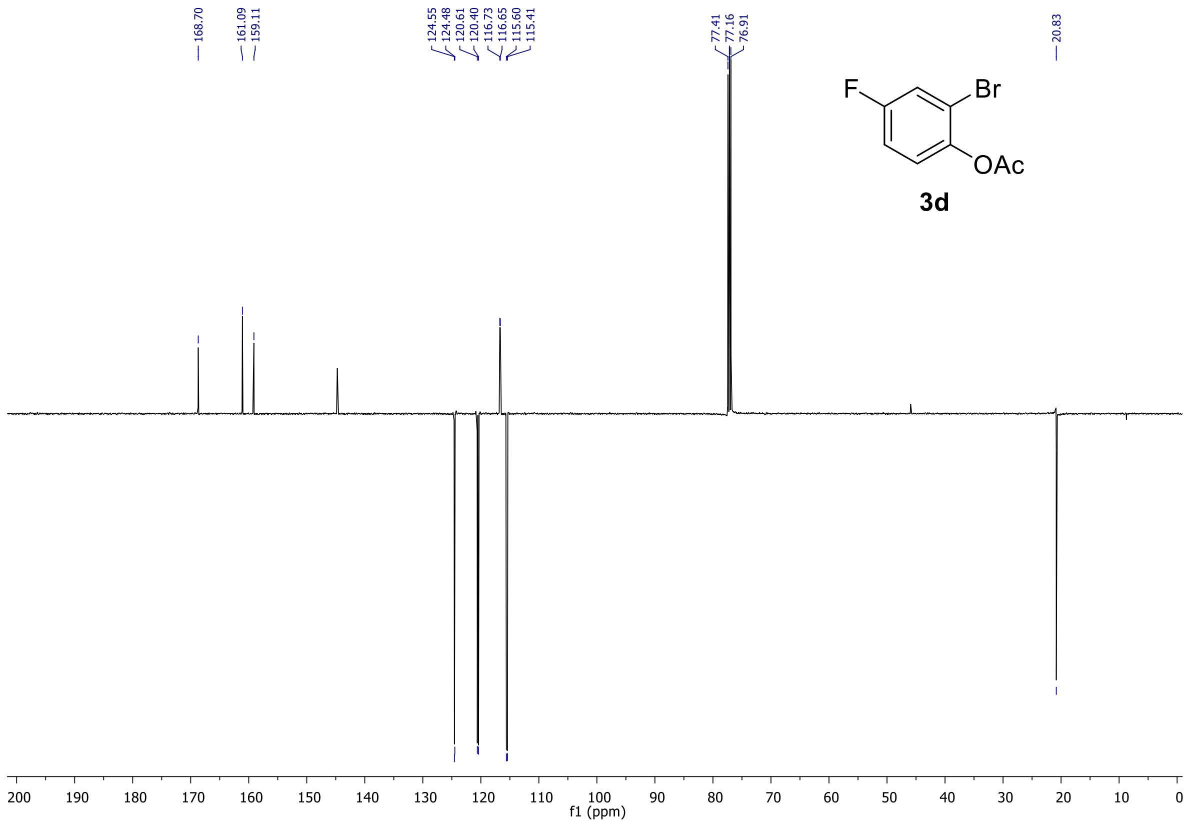

$^{19}F\{^1H\}$ NMR (376 MHz, $CDCl_3$)

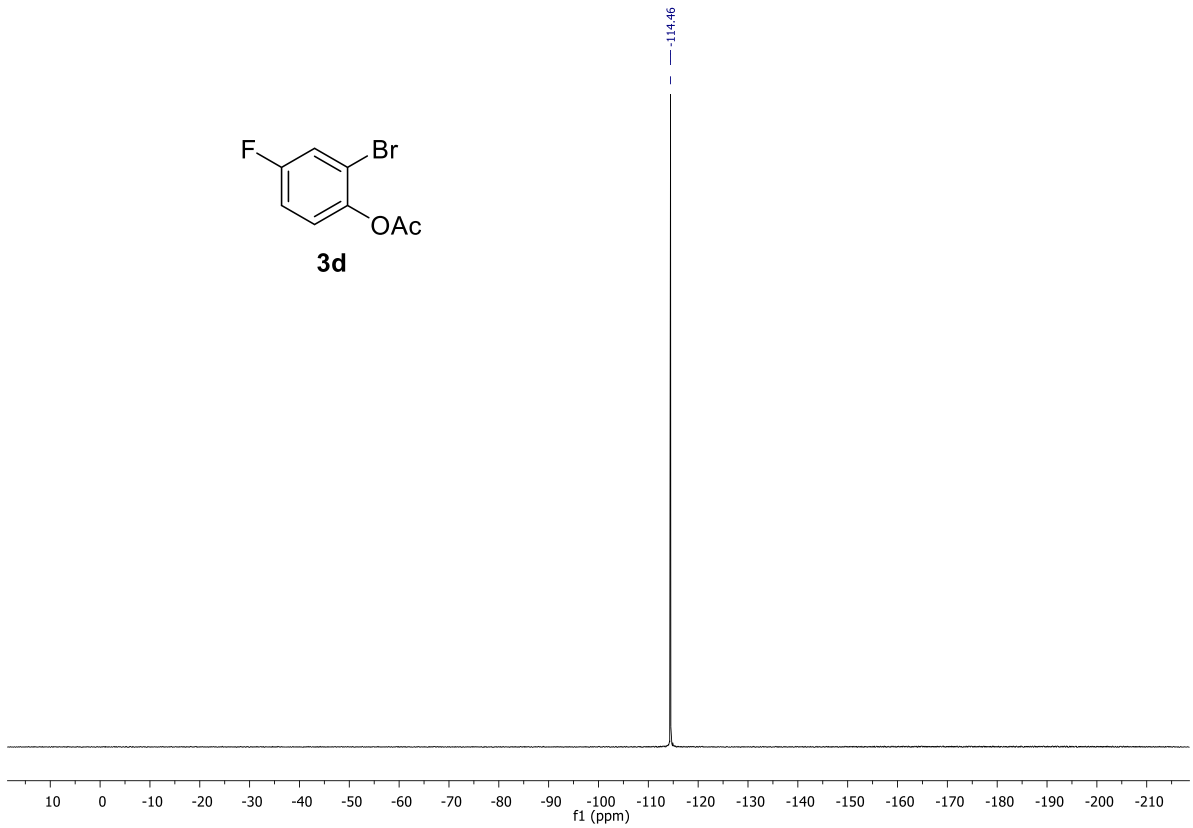

^{1}H NMR (600 MHz, $CDCl_3$)

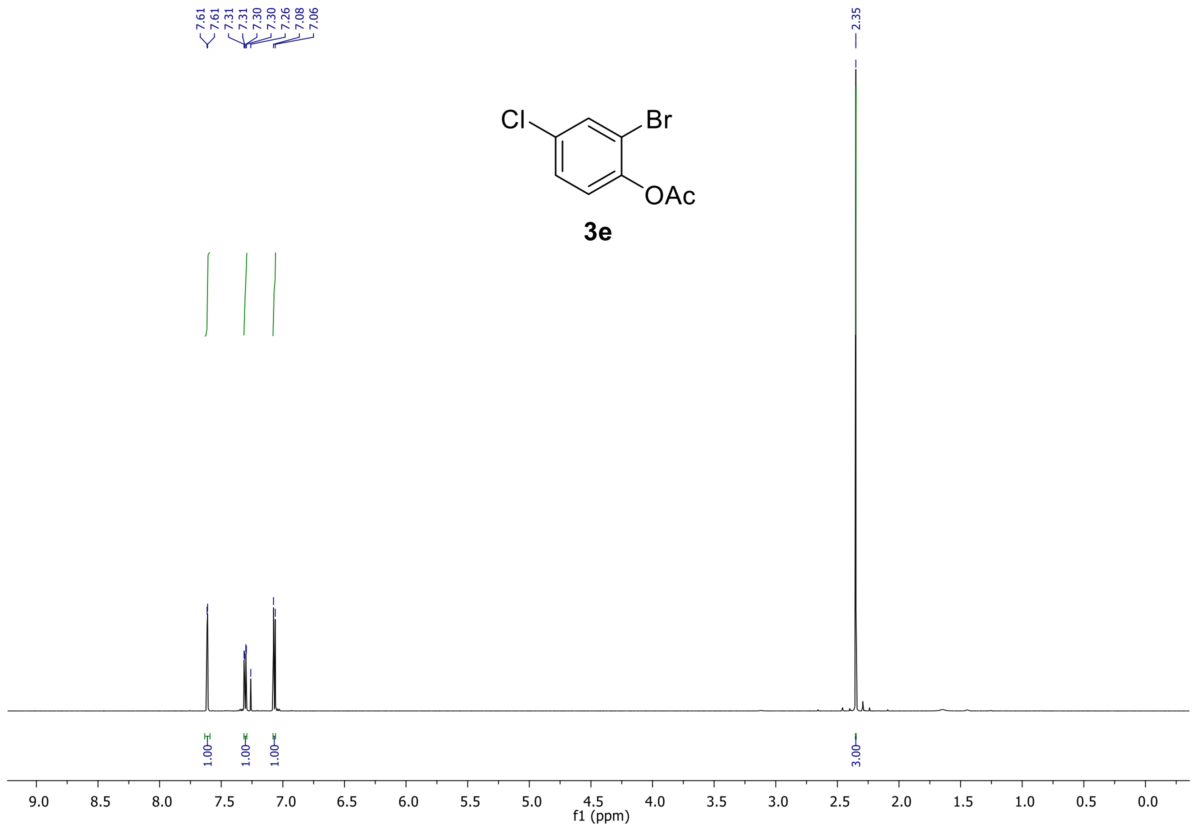

$^{13}C\{^{1}H\}$ NMR (151 MHz, $CDCl_3$)

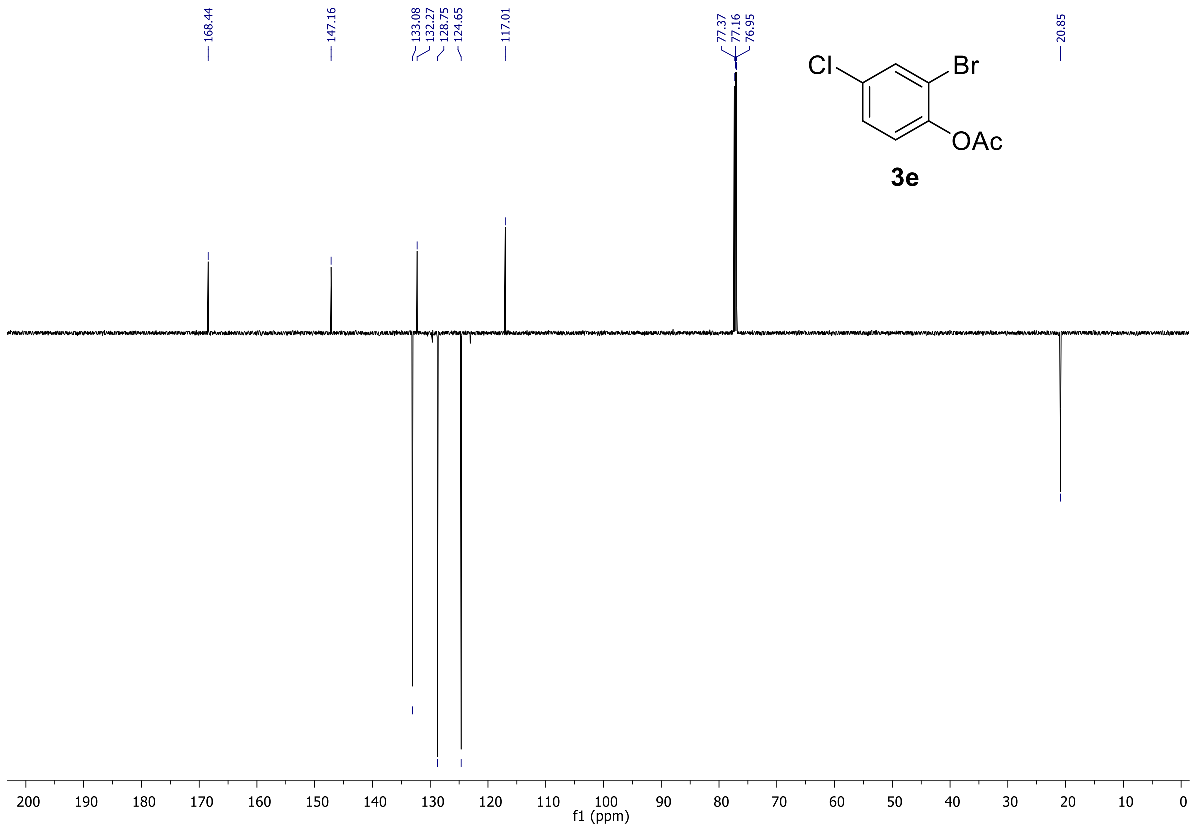

^{1}H NMR (600 MHz, $CDCl_3$)

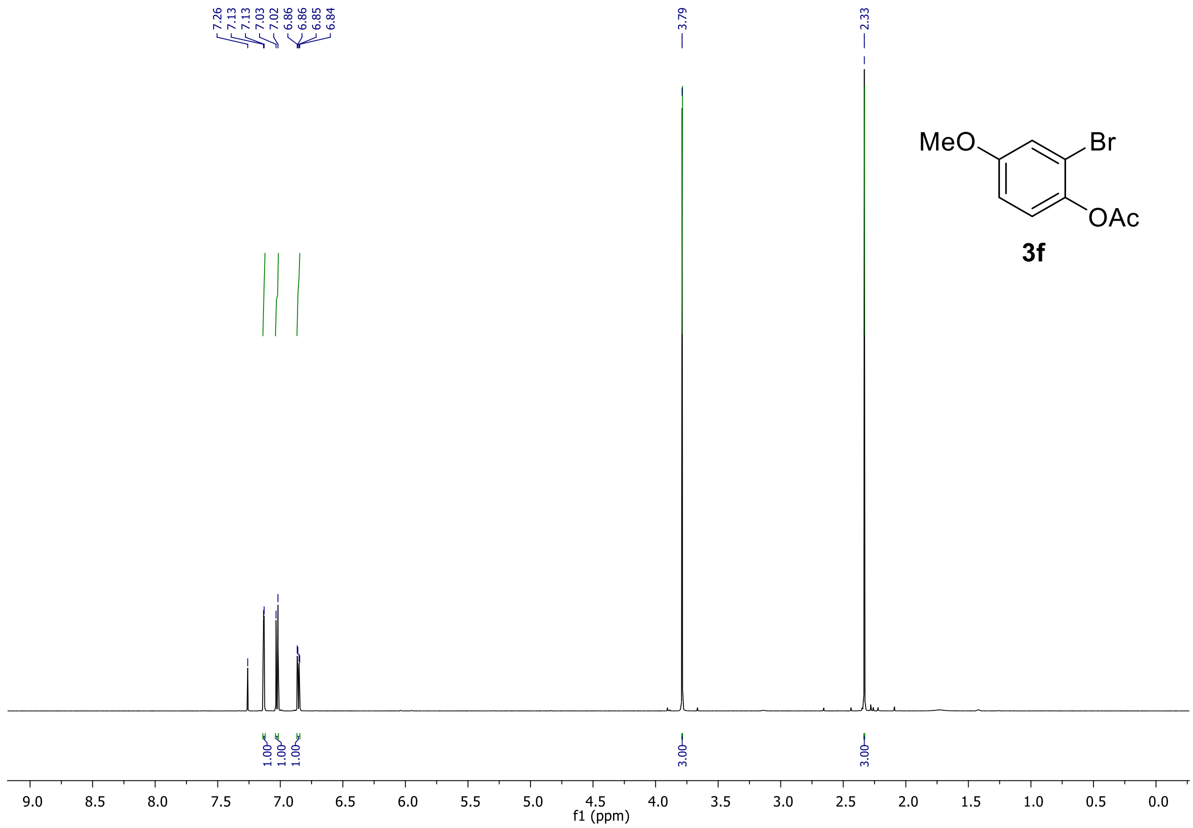

$^{13}C\{^{1}H\}$ NMR (151 MHz, $CDCl_3$)

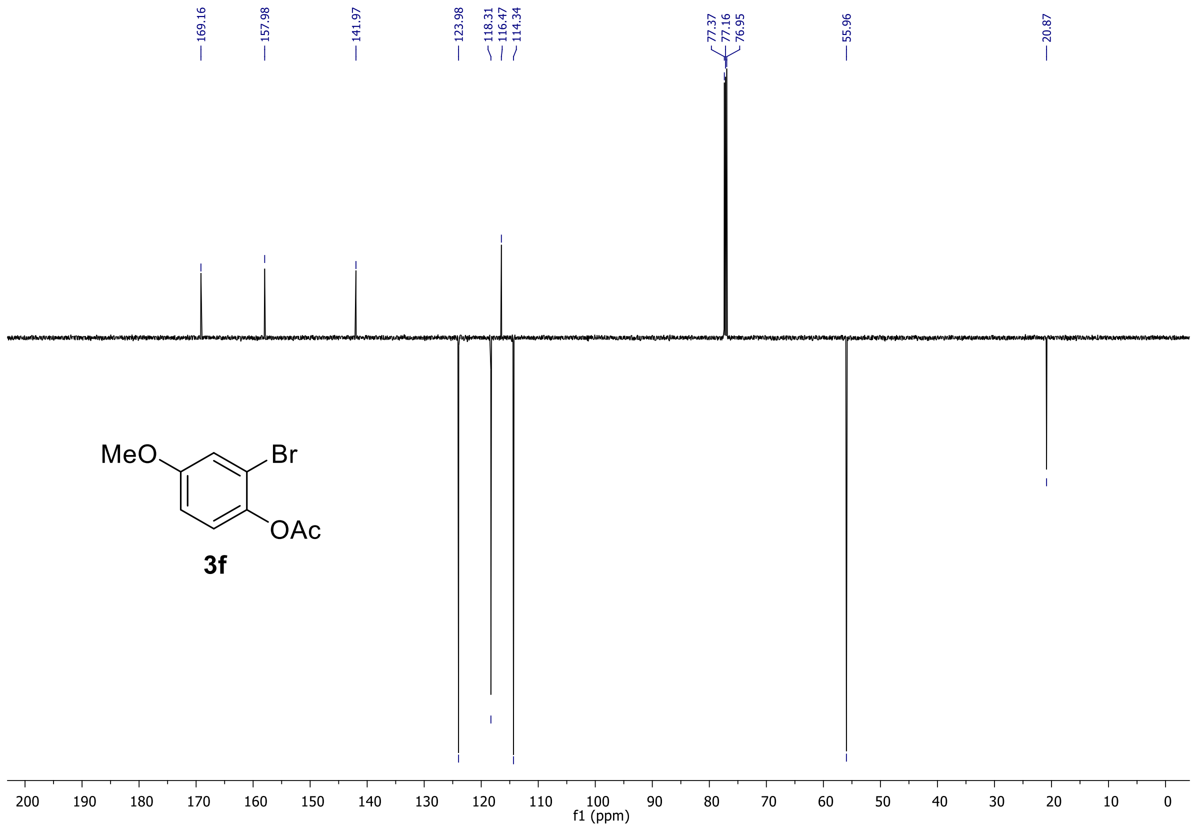

^{1}H NMR (500 MHz, $CDCl_3$)

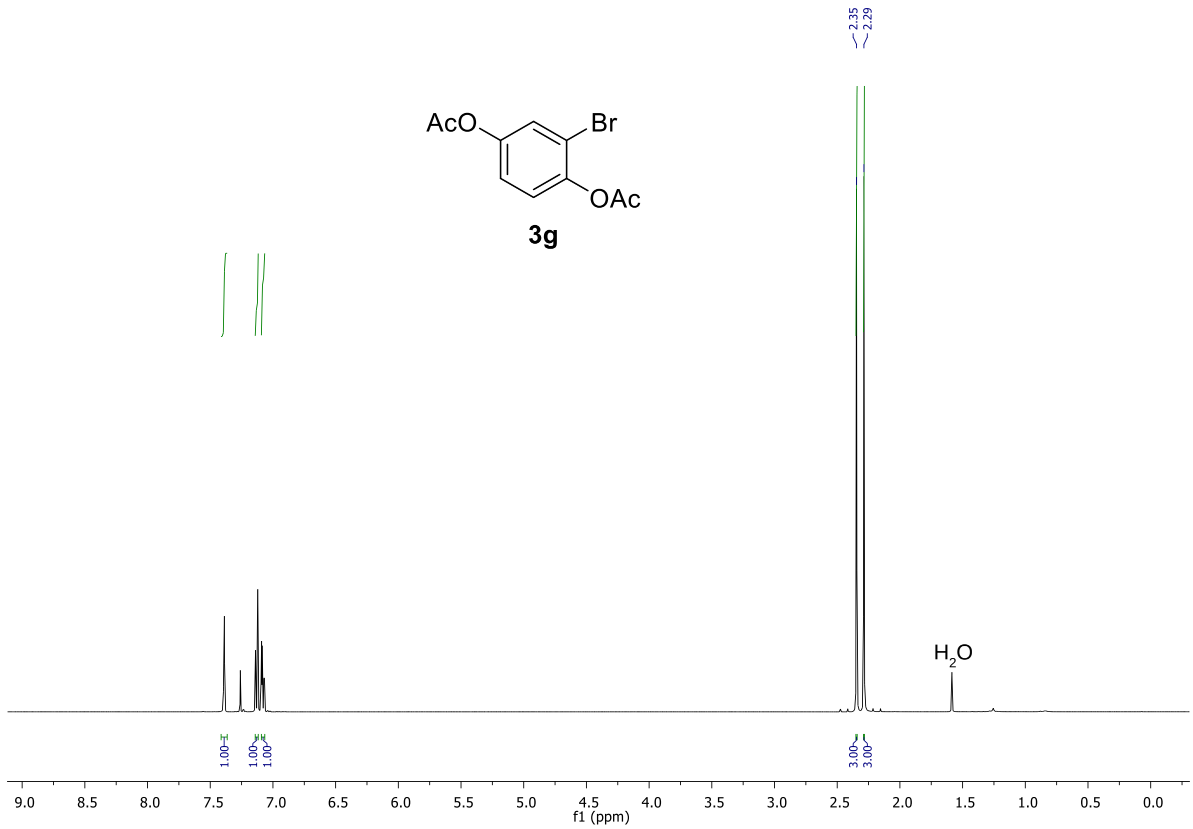

^{13}C{^{1}H} NMR (126 MHz, $CDCl_3$)

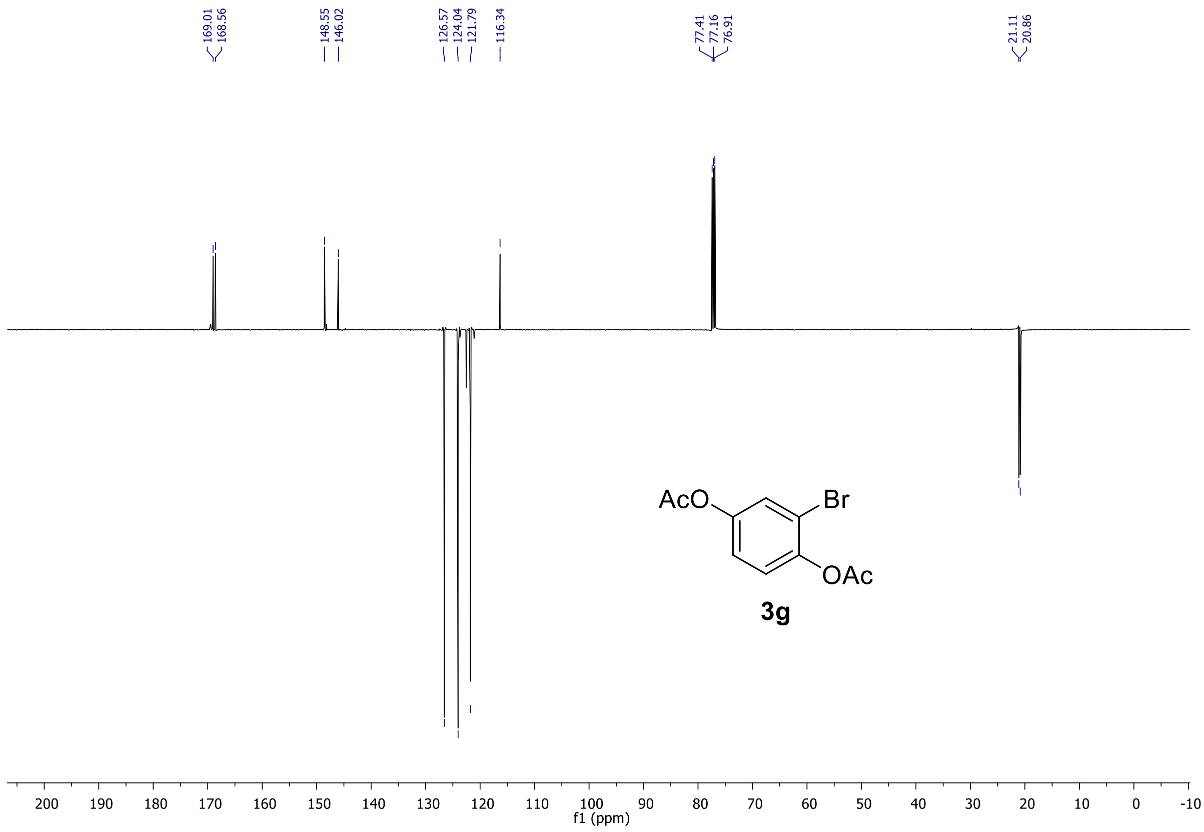

^{1}H NMR (600 MHz, $CDCl_3$)

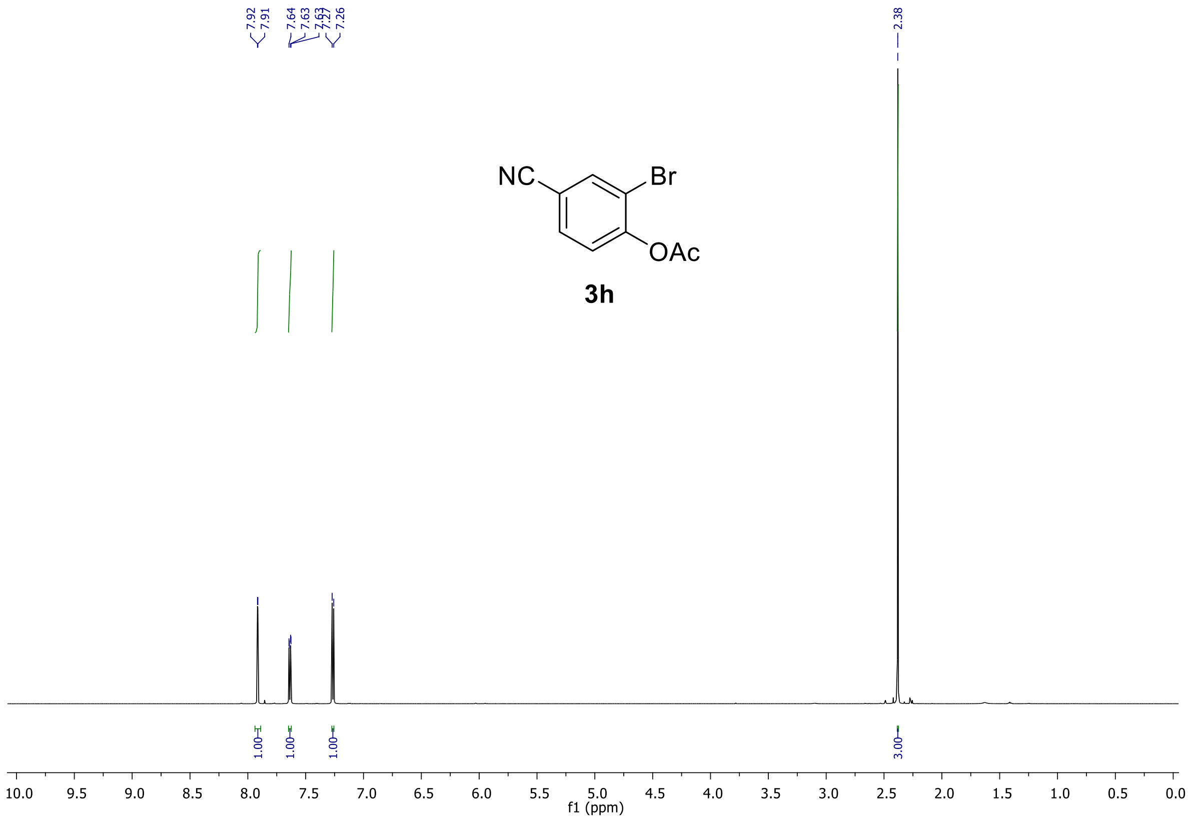

$^{13}C\{^{1}H\}$ NMR (151 MHz, $CDCl_3$)

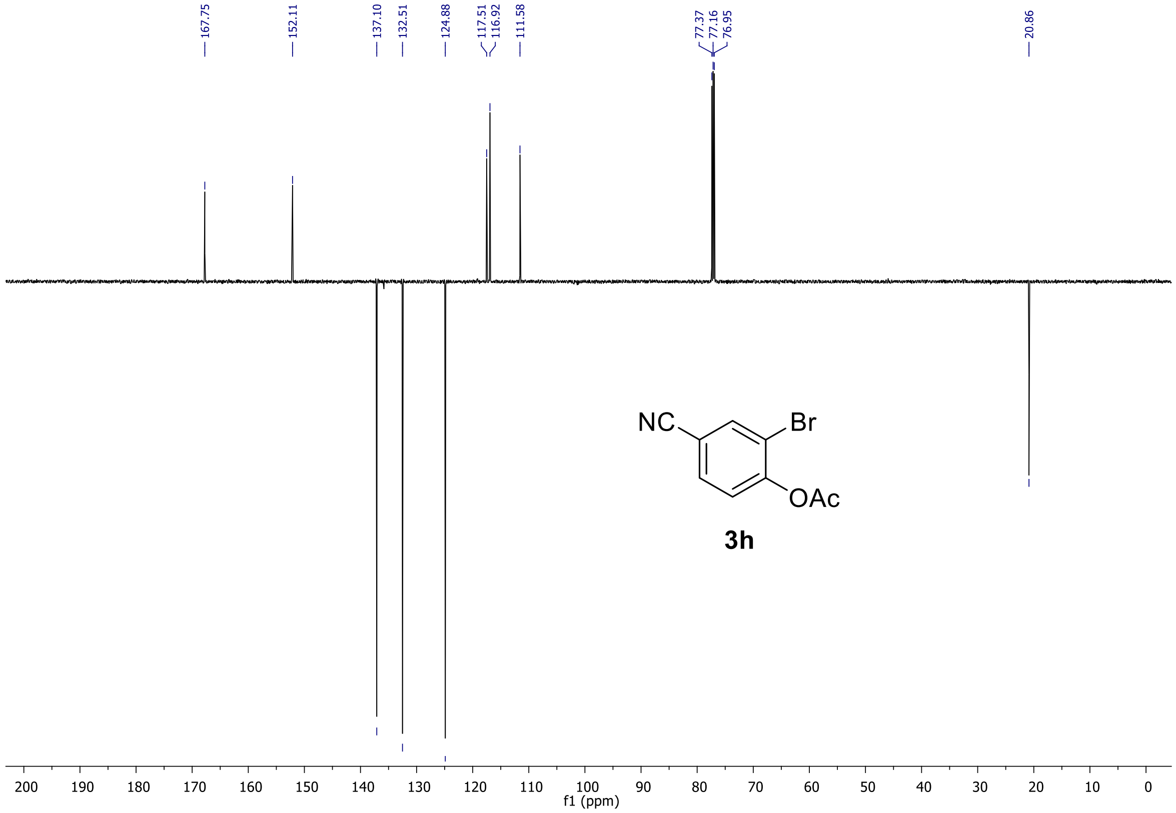

^{1}H NMR (500 MHz, $CDCl_3$)

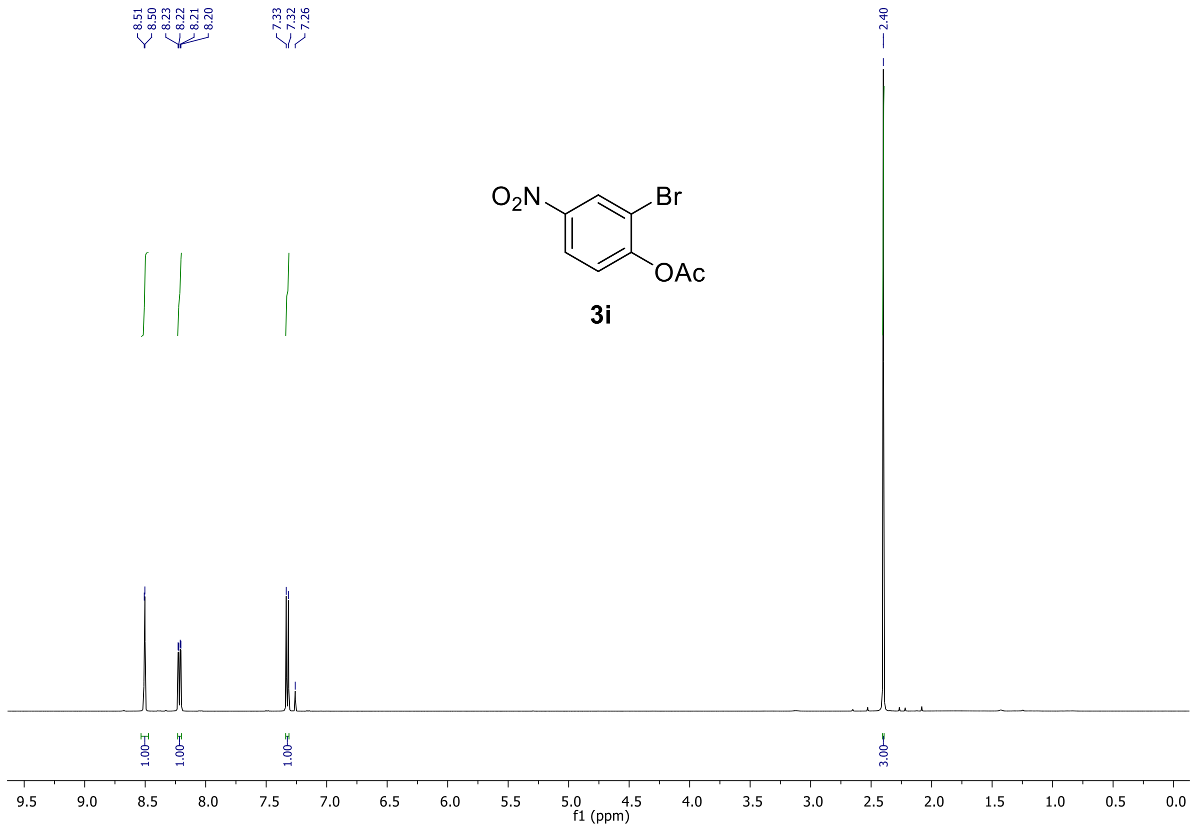

$^{13}C\{^{1}H\}$ NMR (126 MHz, $CDCl_3$)

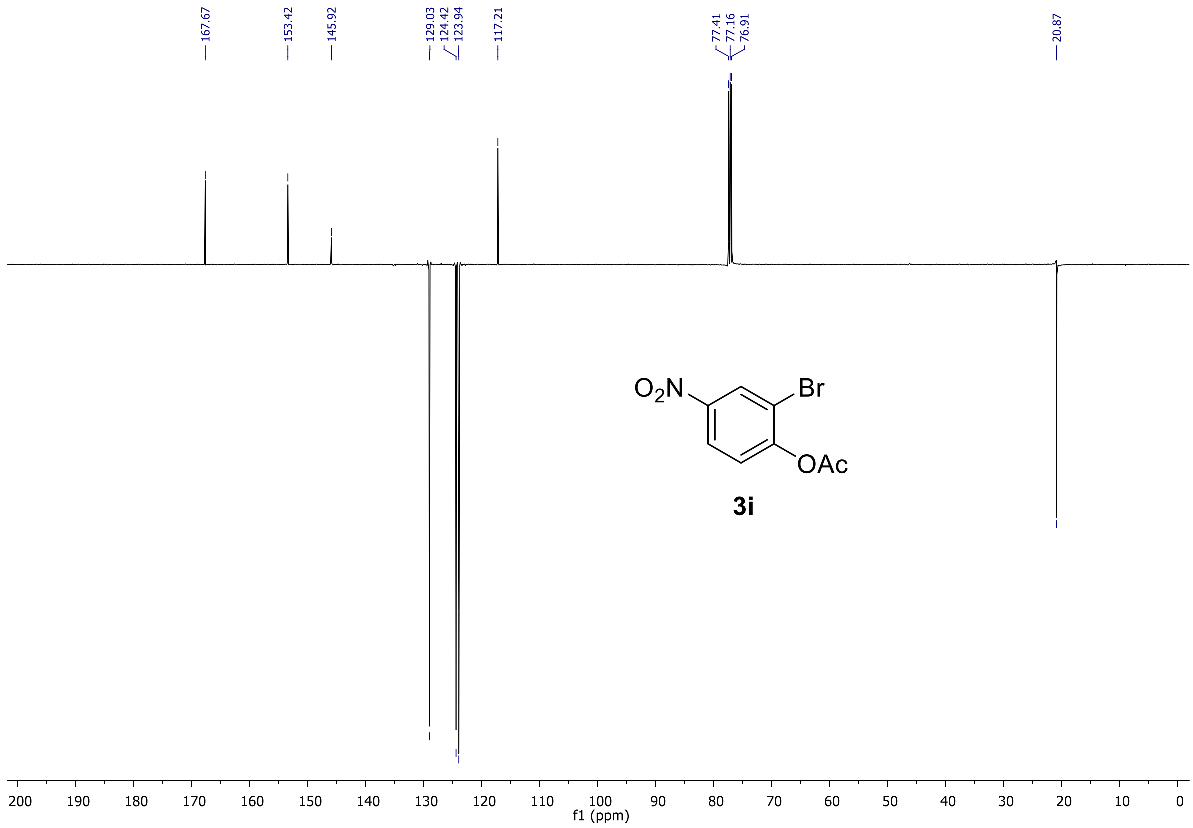

^{1}H NMR (500 MHz, $CDCl_3$)

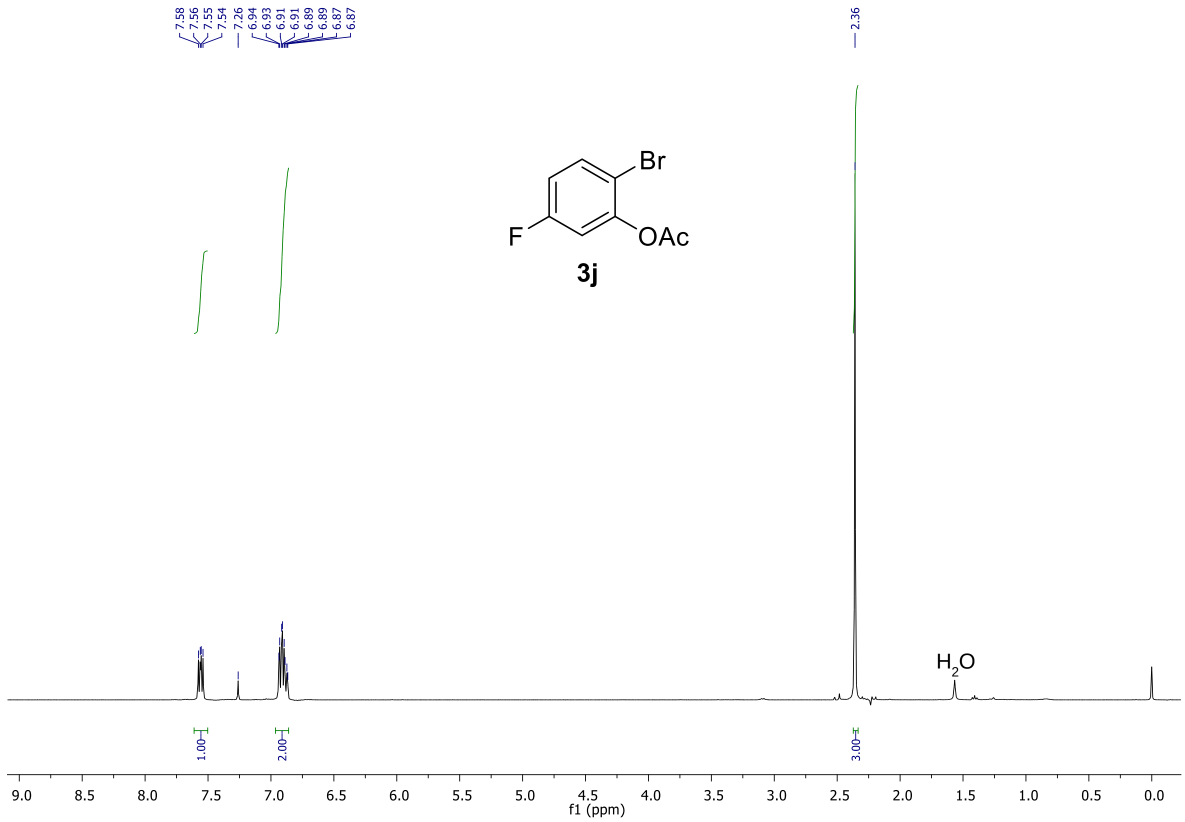

^{13}C{^{1}H} NMR (126 MHz, $CDCl_3$)

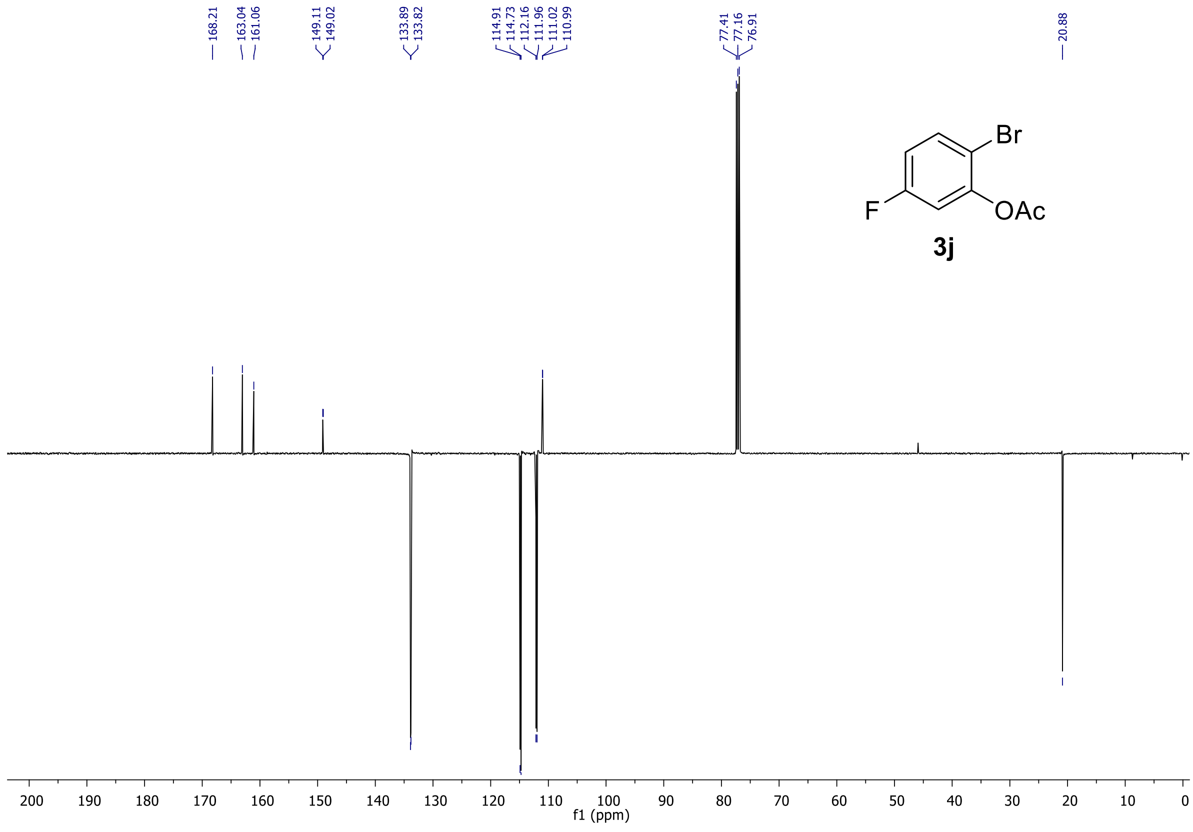

$^{19}F\{^{1}H\}$ NMR (376 MHz, $CDCl_3$)

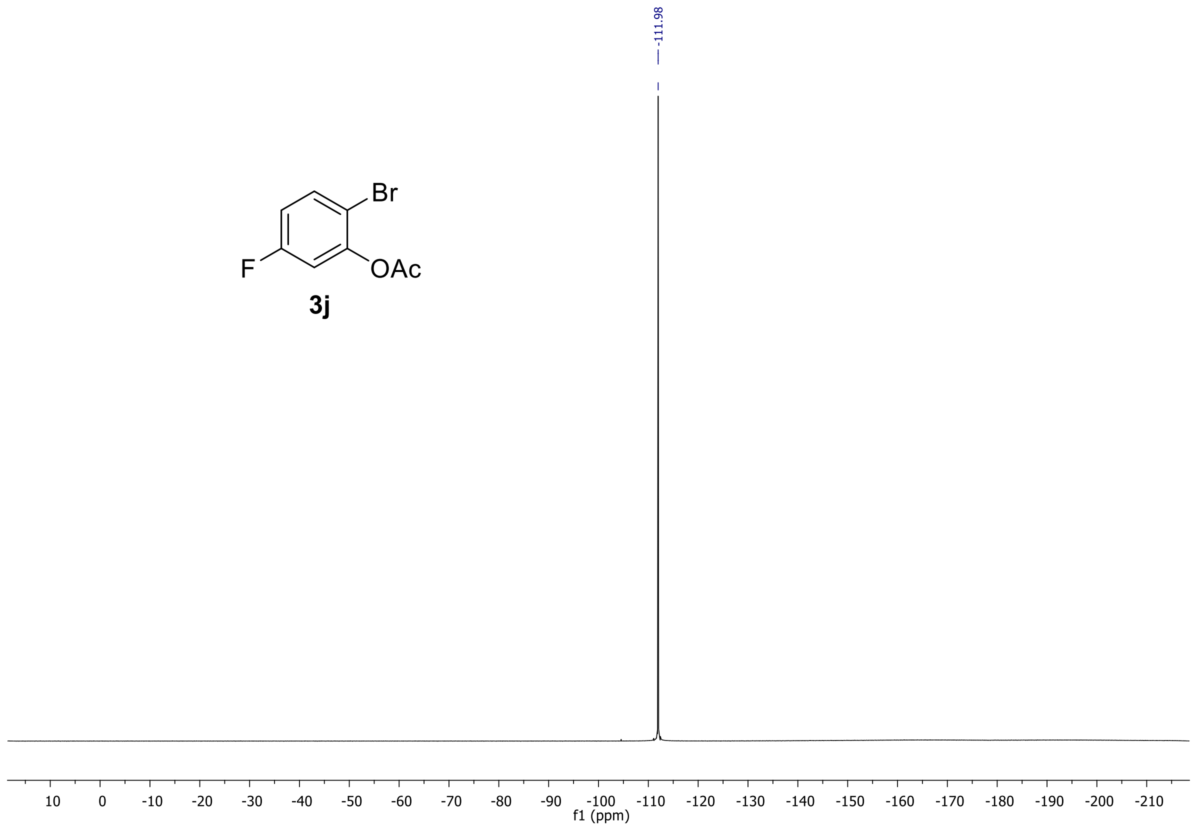

^{1}H NMR (500 MHz, $CDCl_3$)

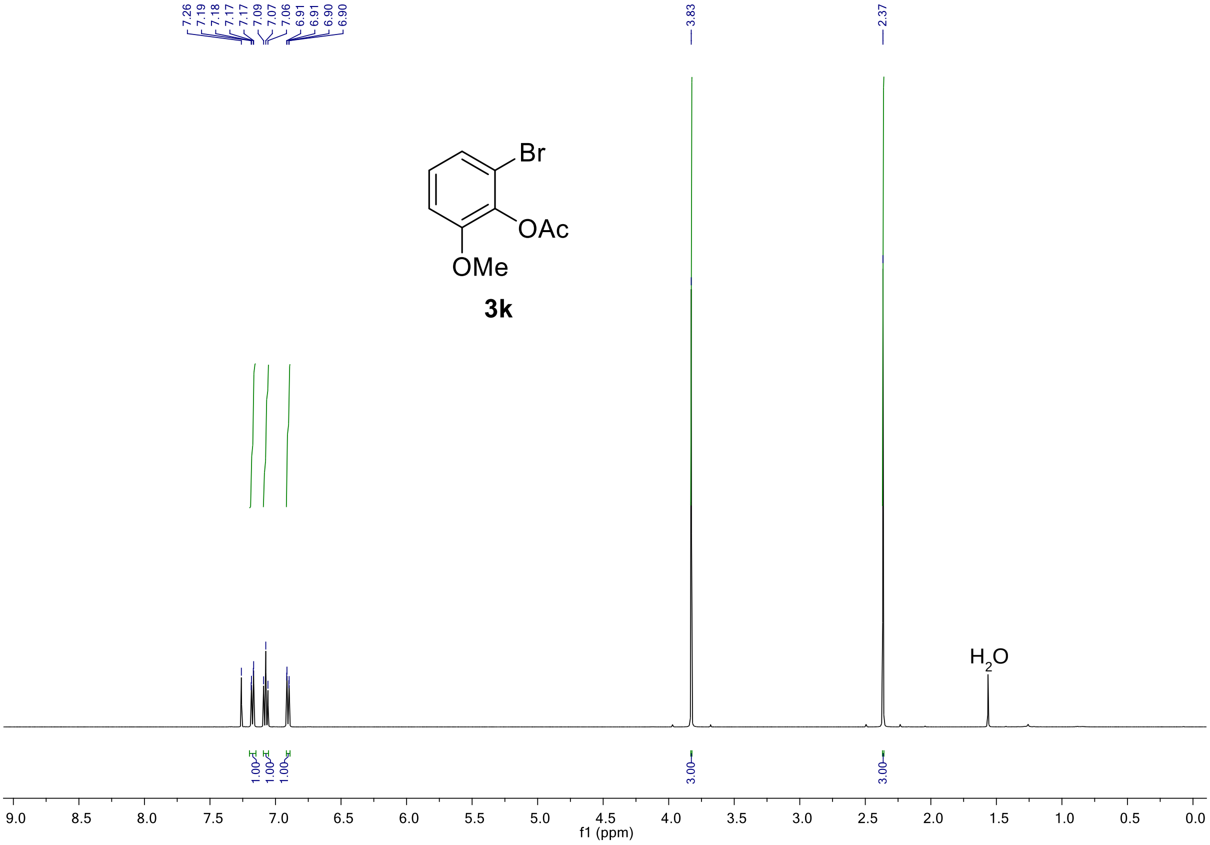

$^{13}C\{^{1}H\}$ NMR (126 MHz, $CDCl_3$)

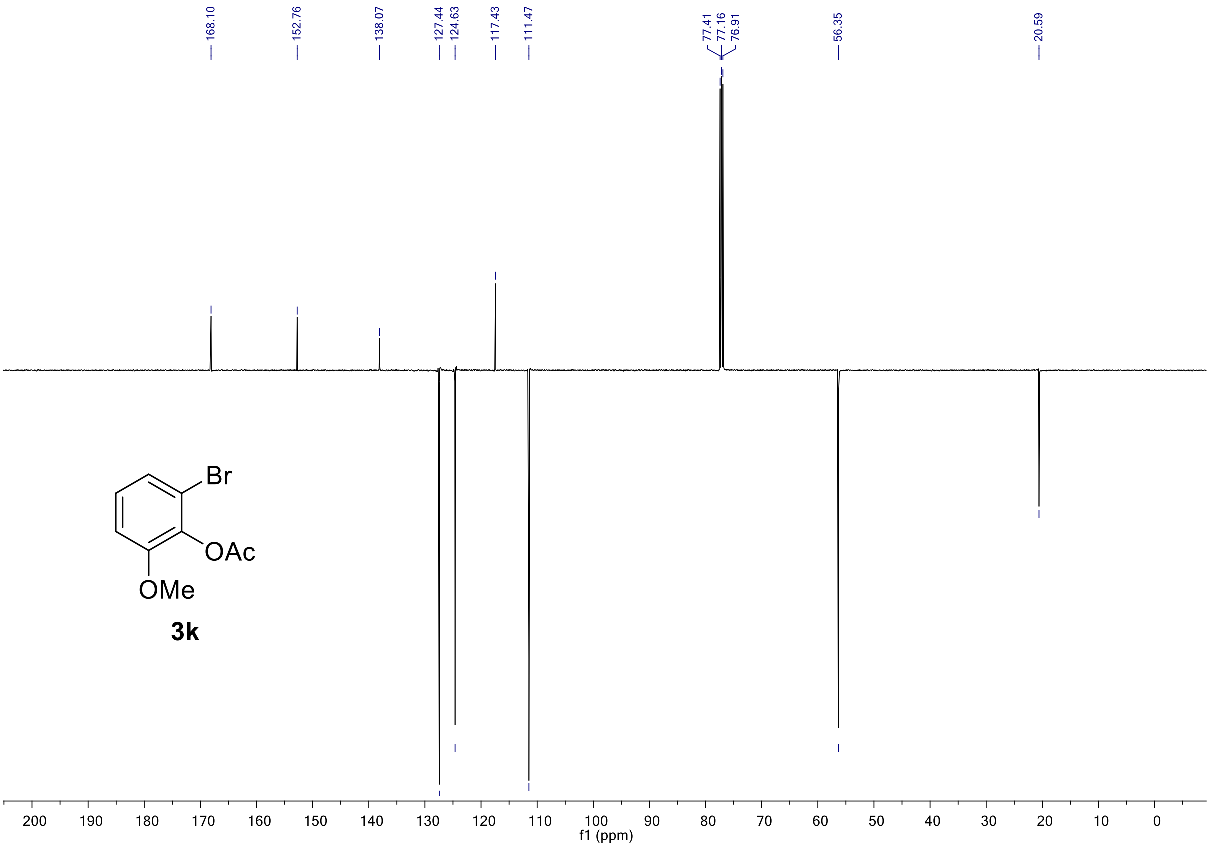

^{1}H NMR (500 MHz, $CDCl_3$)

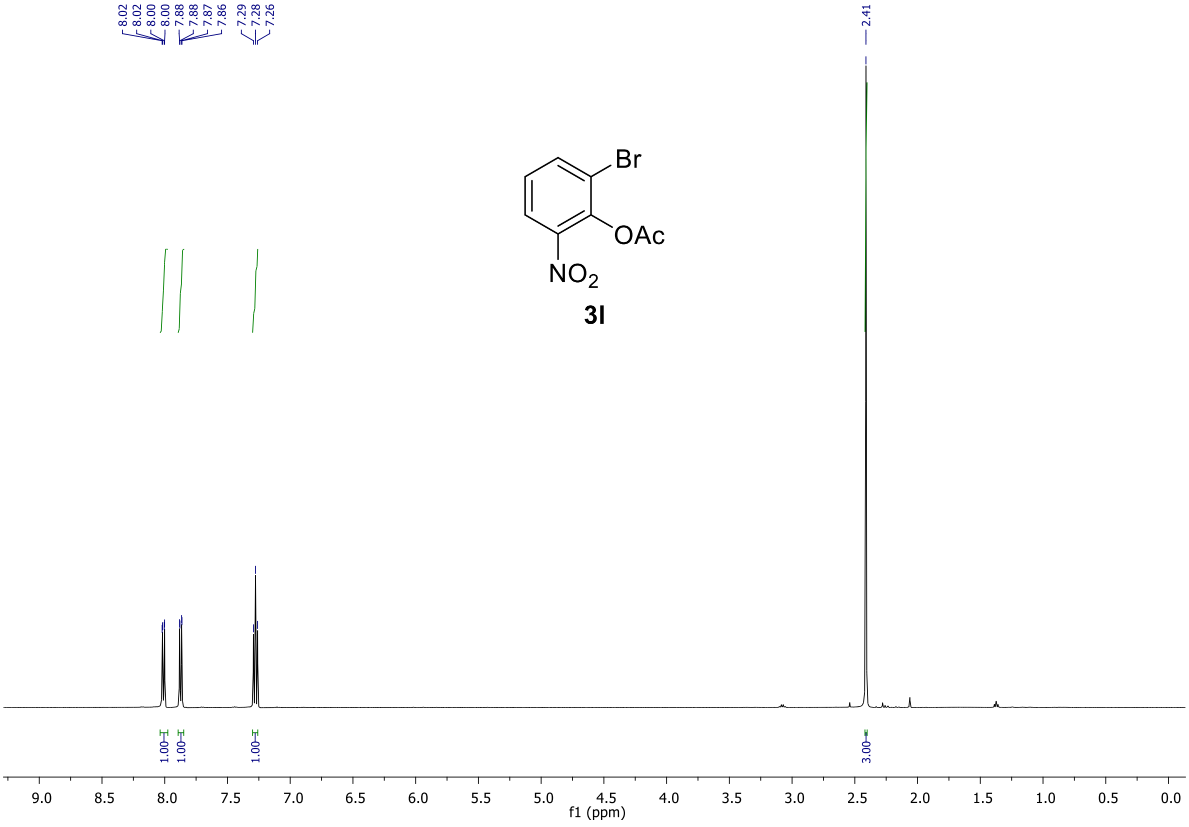

$^{13}C\{^{1}H\}$ NMR (126 MHz, $CDCl_3$)

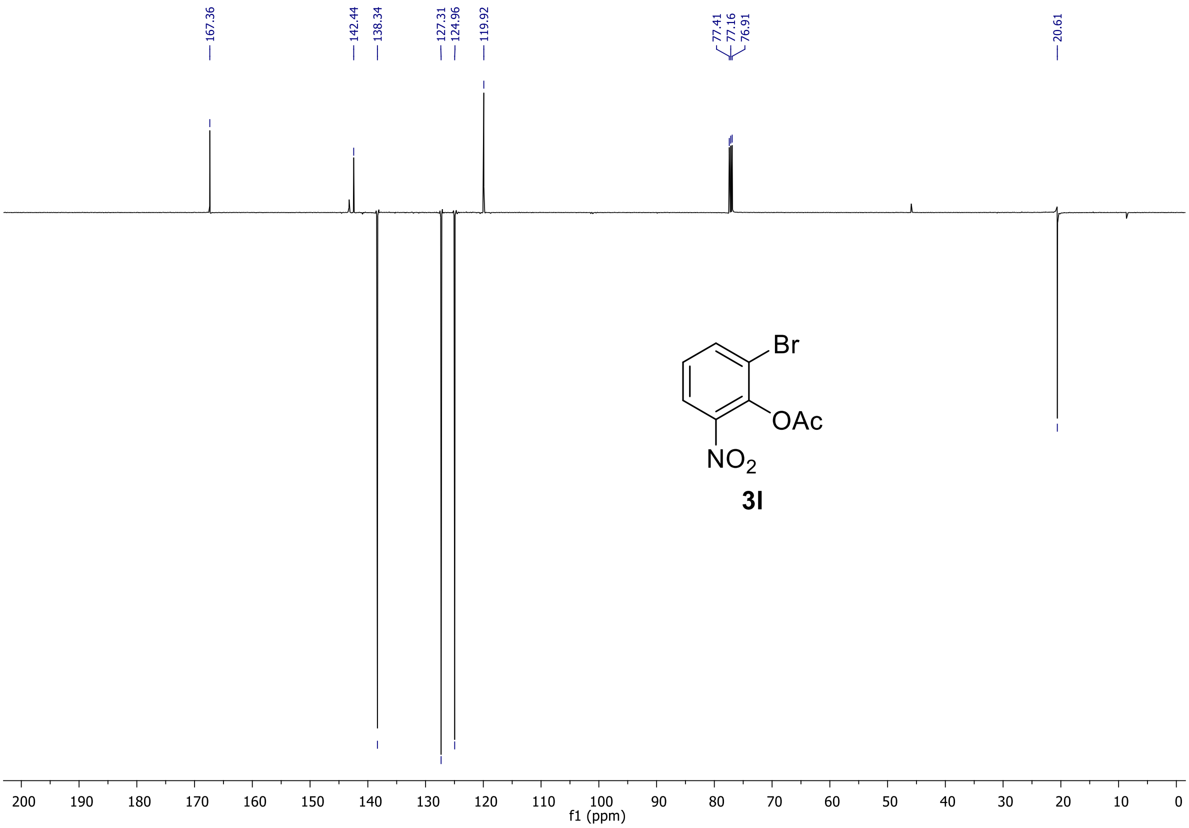

^{1}H NMR (600 MHz, $CDCl_3$)

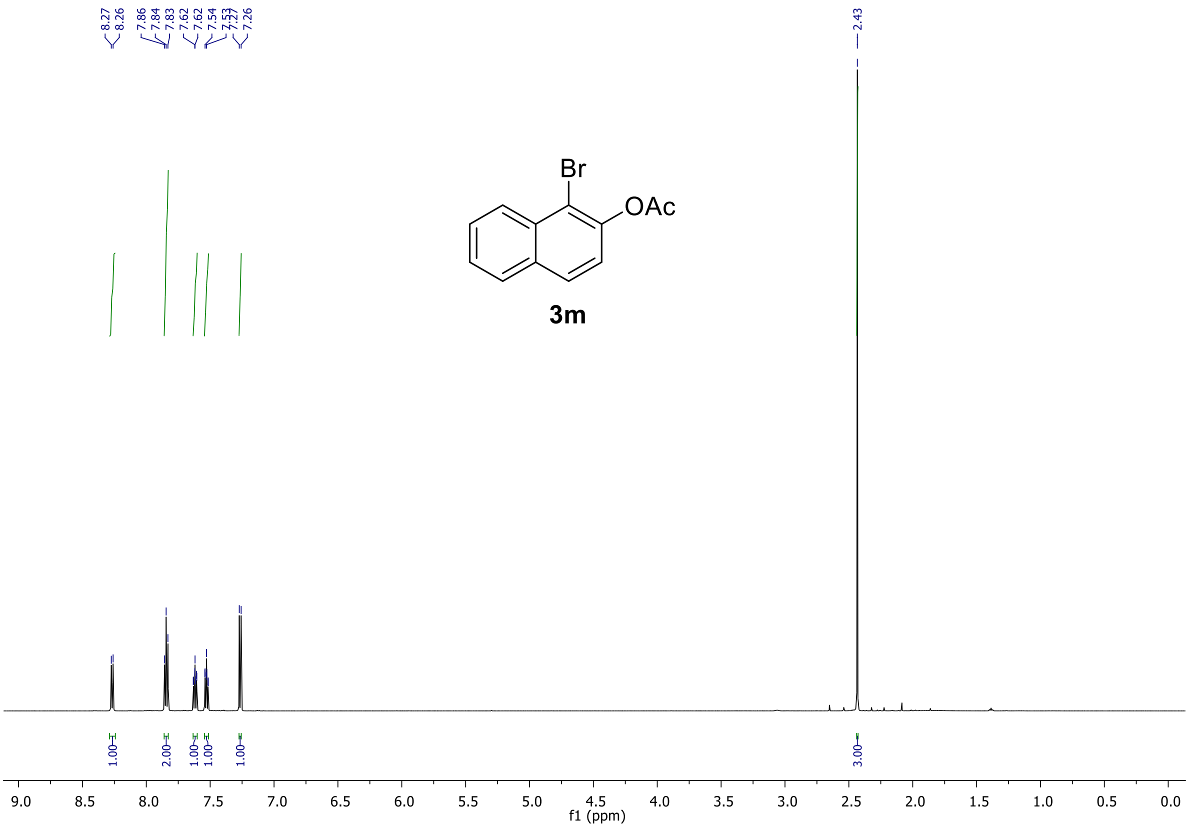

$^{13}C\{^{1}H\}$ NMR (151 MHz, $CDCl_3$)

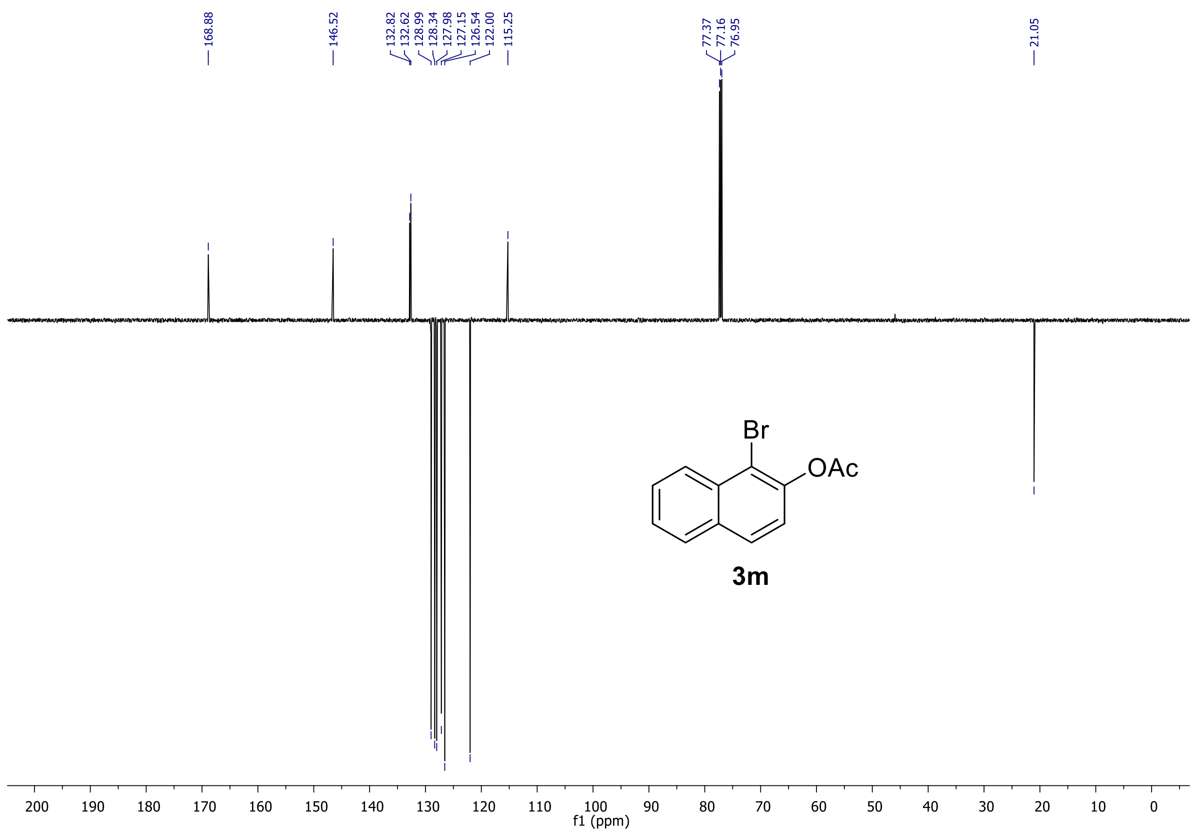

^{1}H NMR (500 MHz, $CDCl_3$)

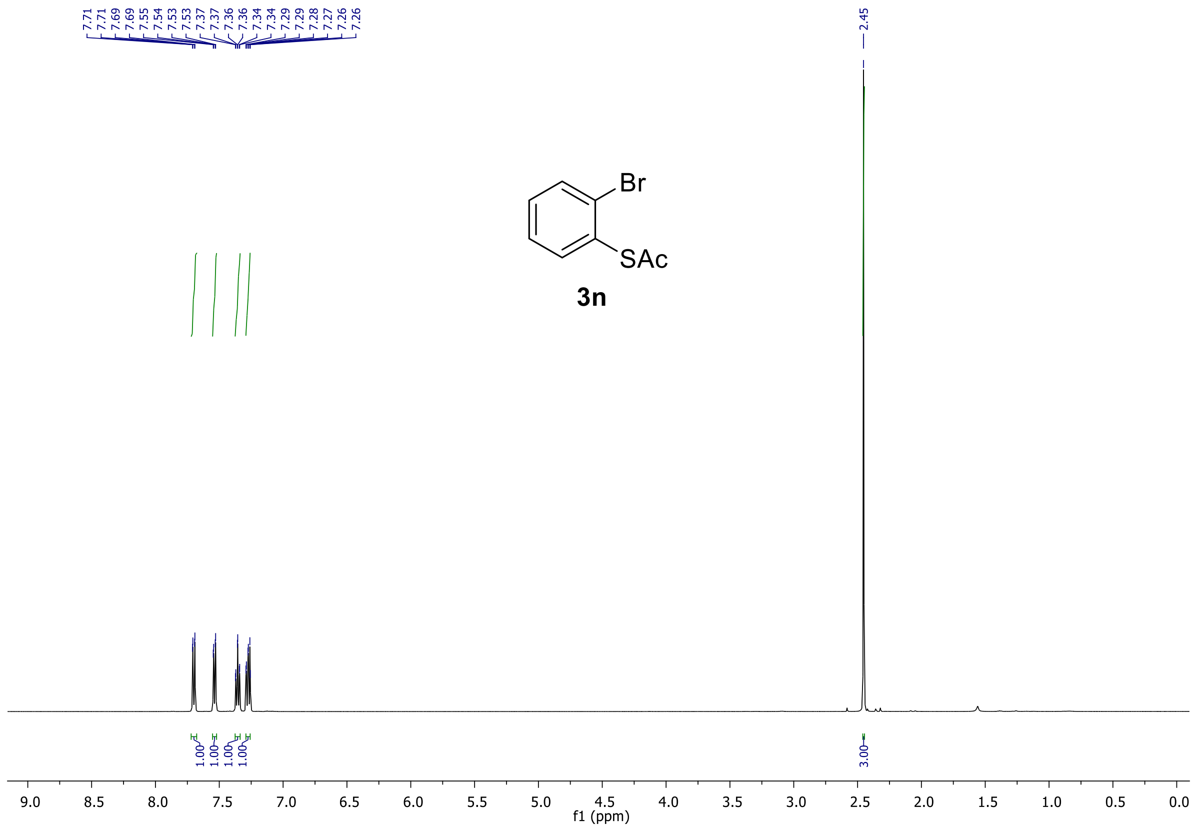

$^{13}C\{^1H\}$ NMR (126 MHz, $CDCl_3$)

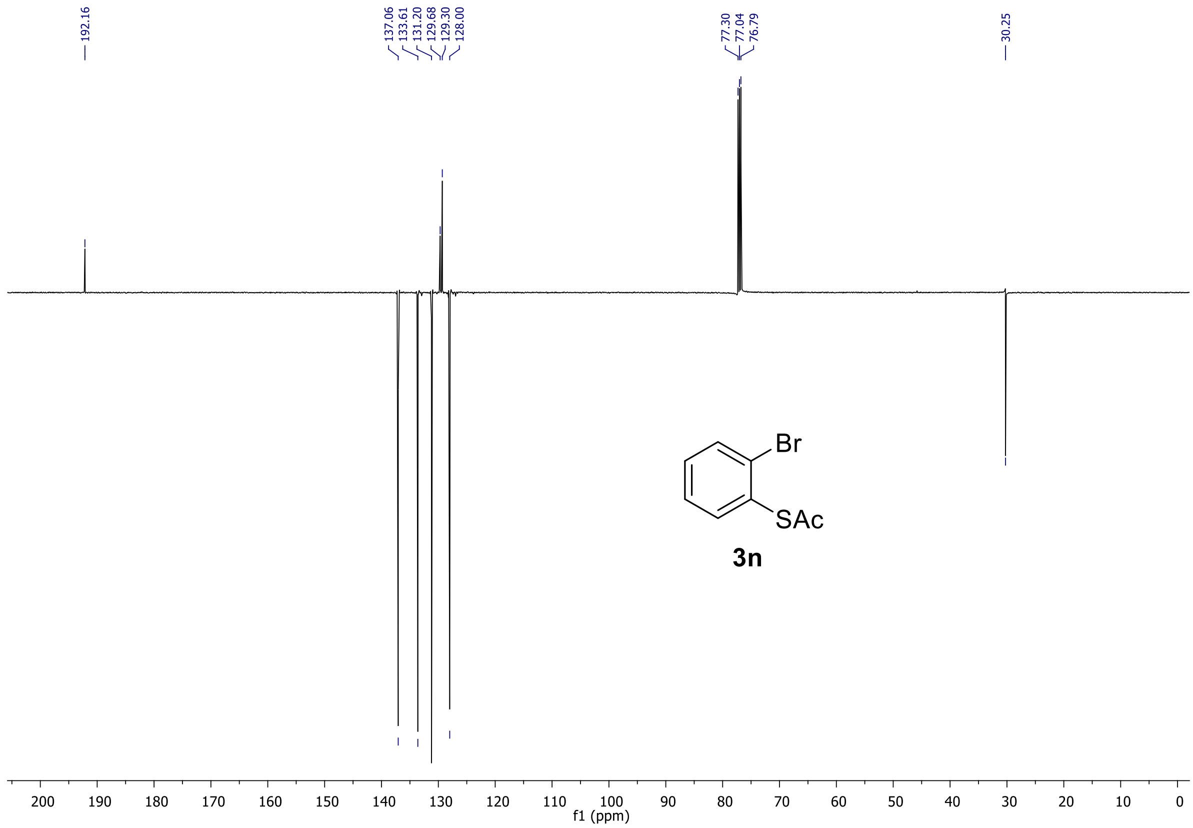

^1H NMR (500 MHz, $CDCl_3$)

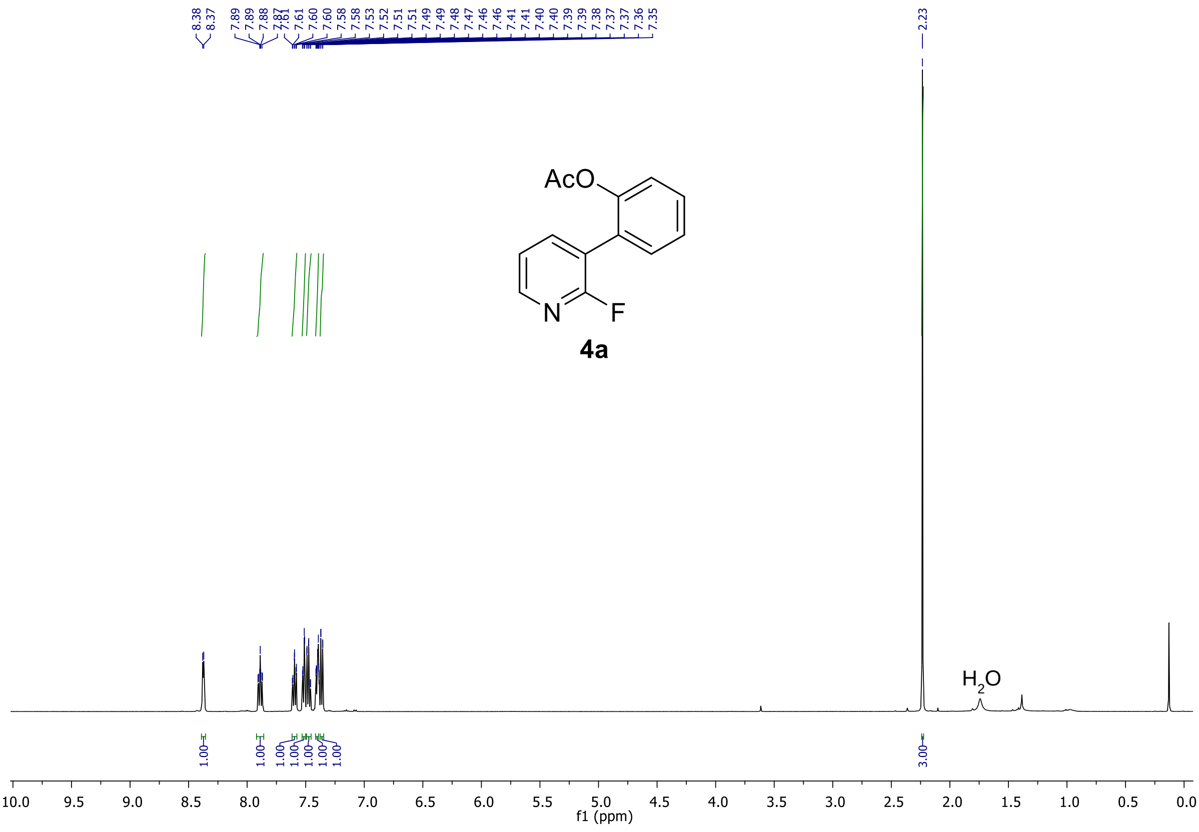

$^{13}C\{^{1}H\}$ NMR (126 MHz, $CDCl_3$)

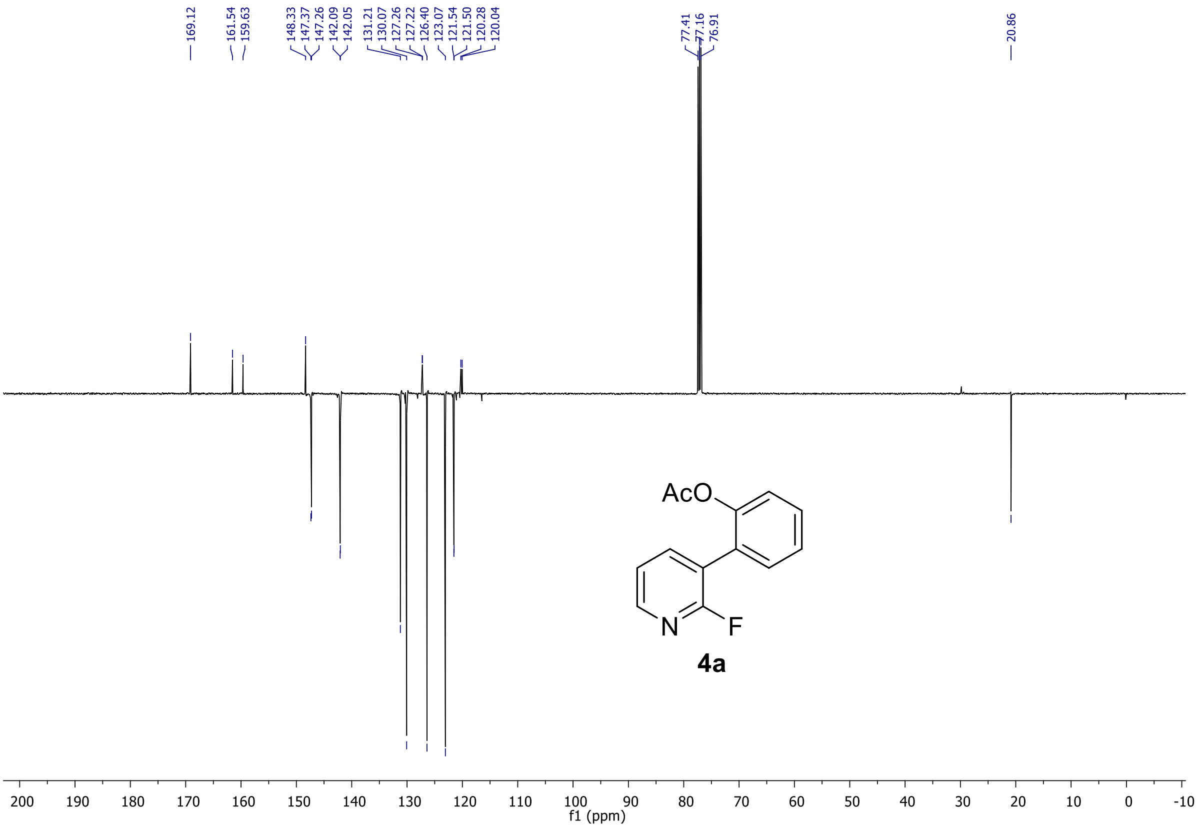

$^{19}F\{^{1}H\}$ NMR (376 MHz, $CDCl_3$)

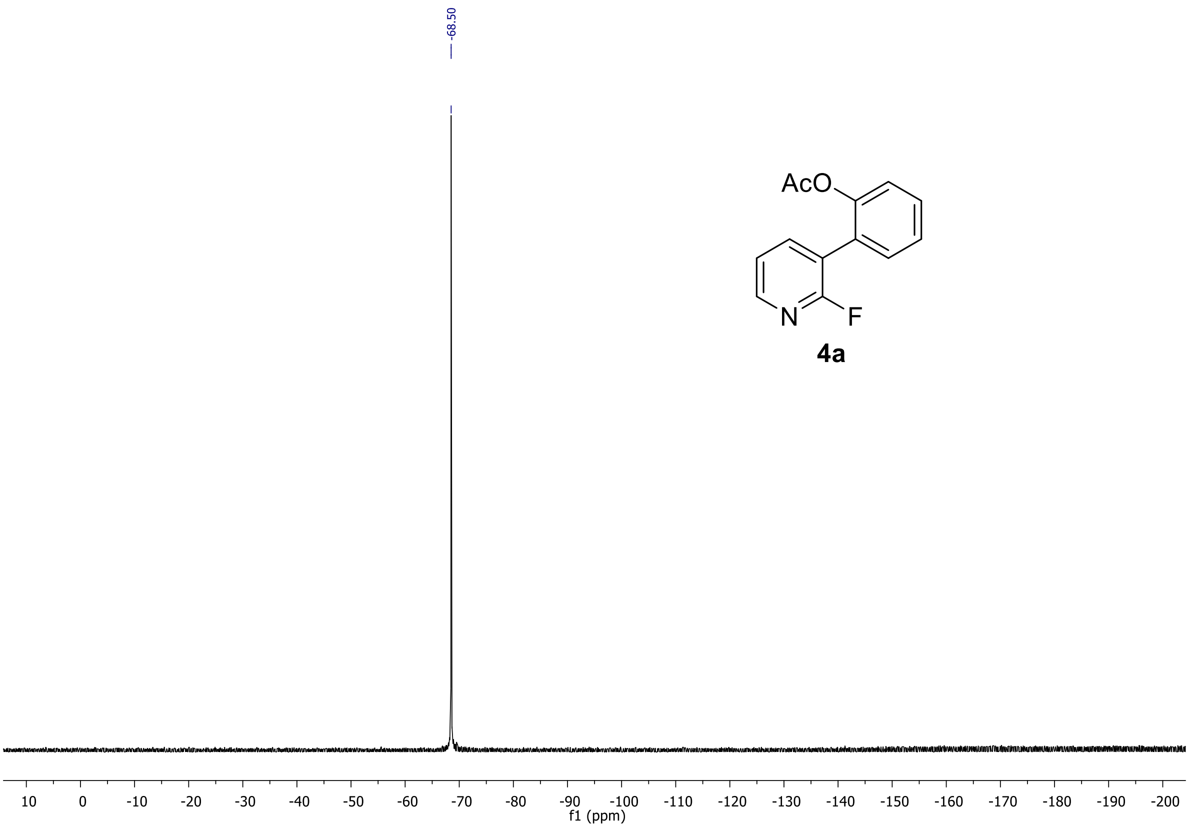

^1H NMR (500 MHz, $CDCl_3$)

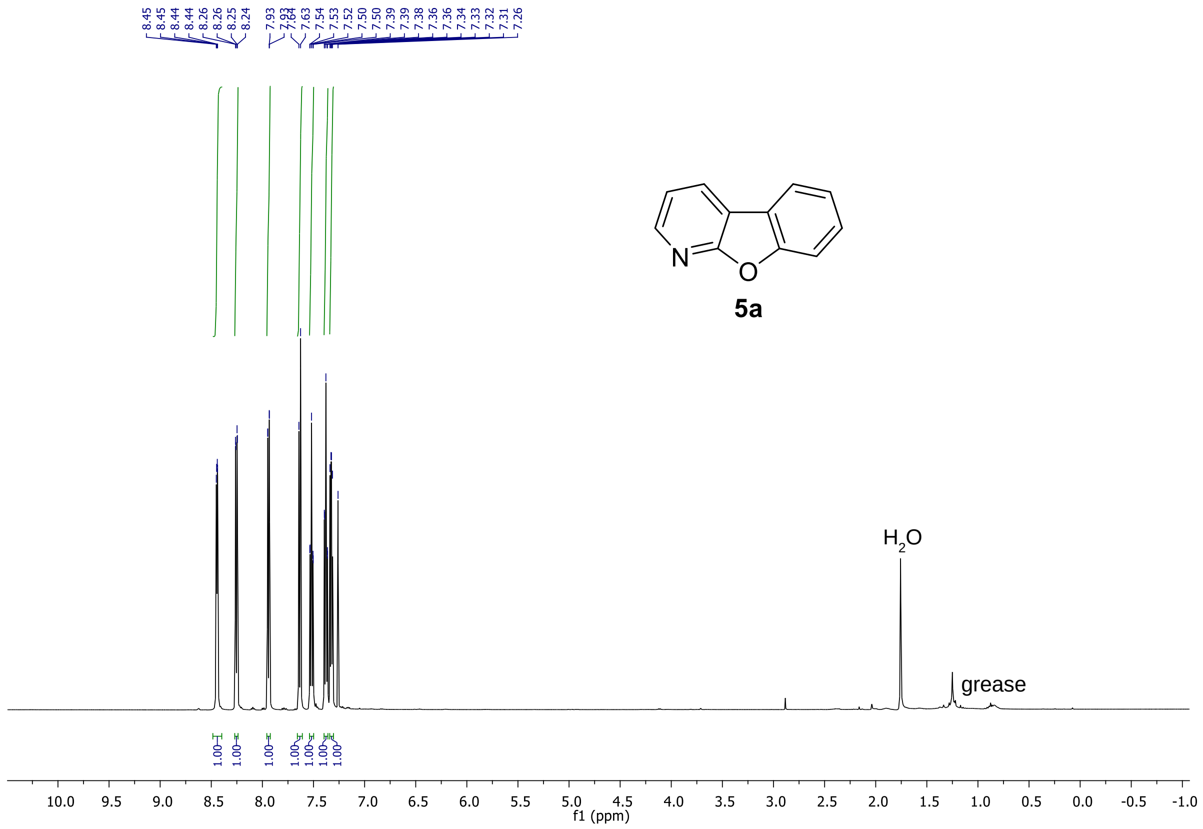

$^{13}C\{^1H\}$ NMR (126 MHz, $CDCl_3$)

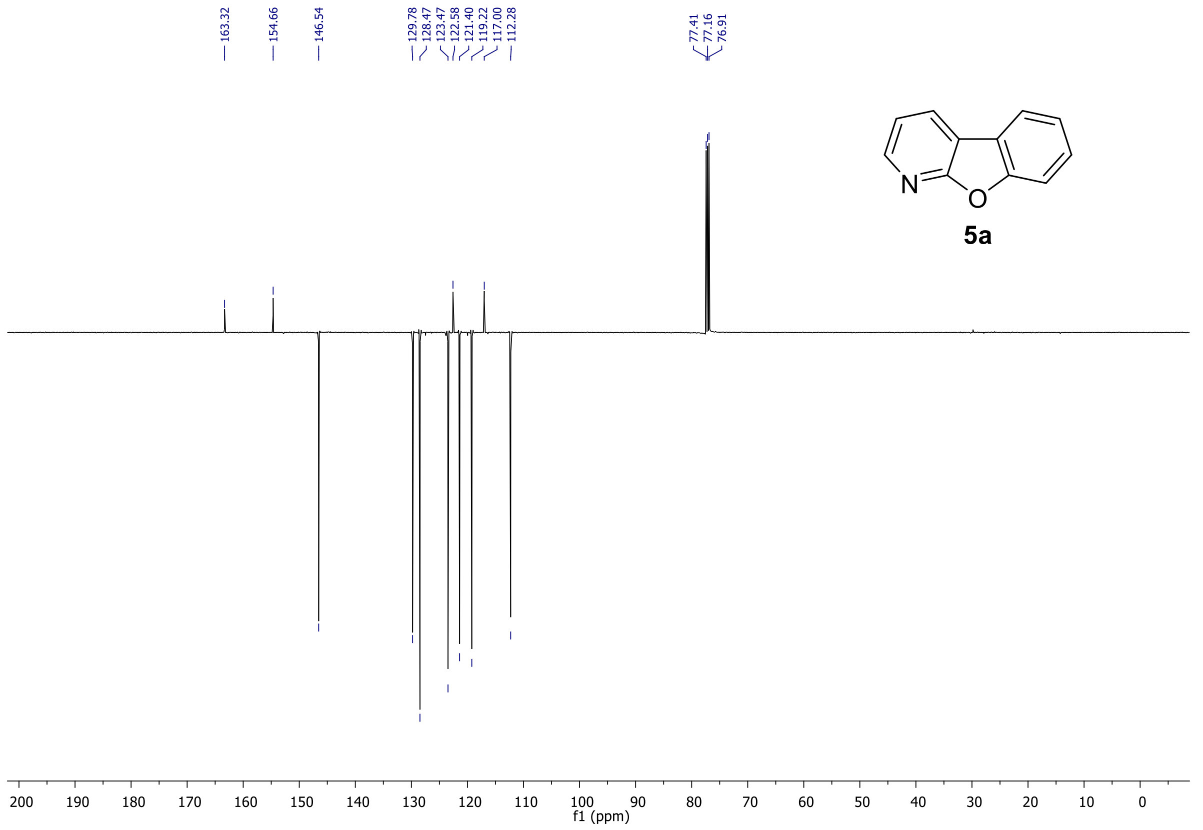

^{1}H NMR (500 MHz, $CDCl_3$)

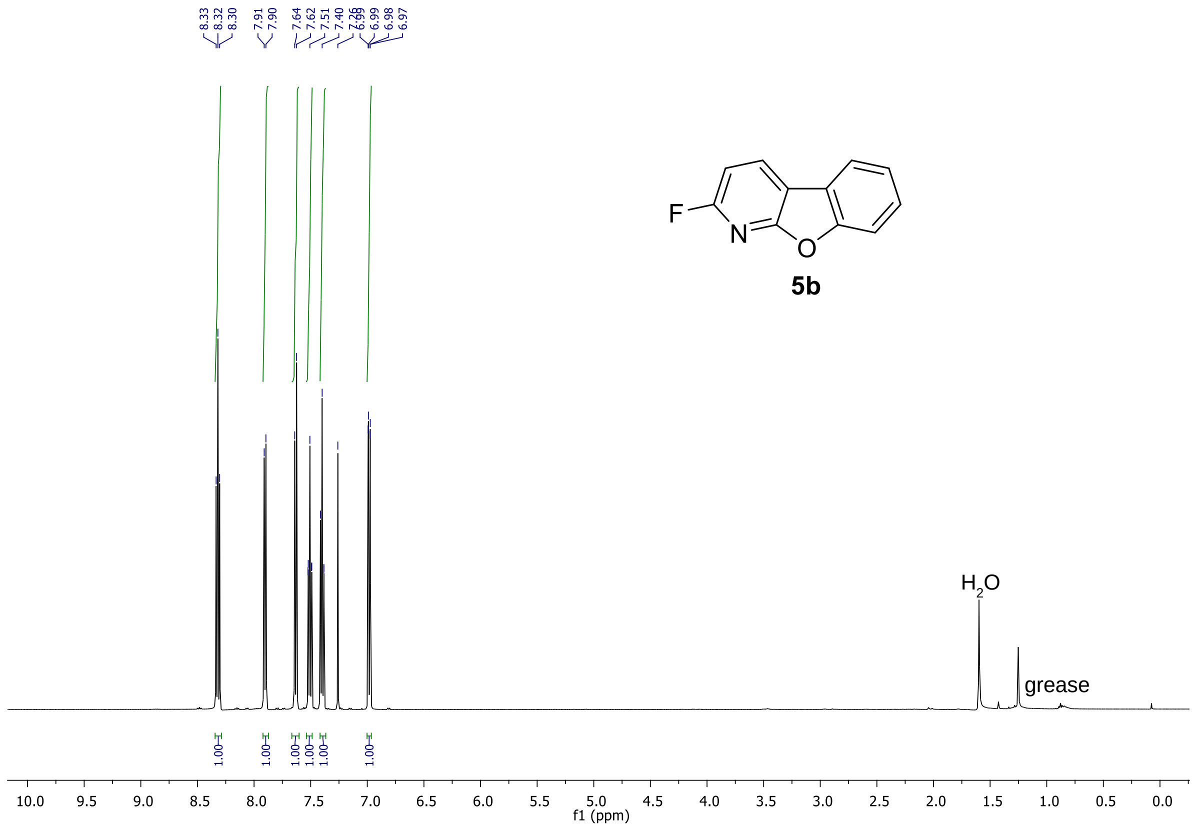

^{13}C{^{1}H} NMR (126 MHz, $CDCl_3$)

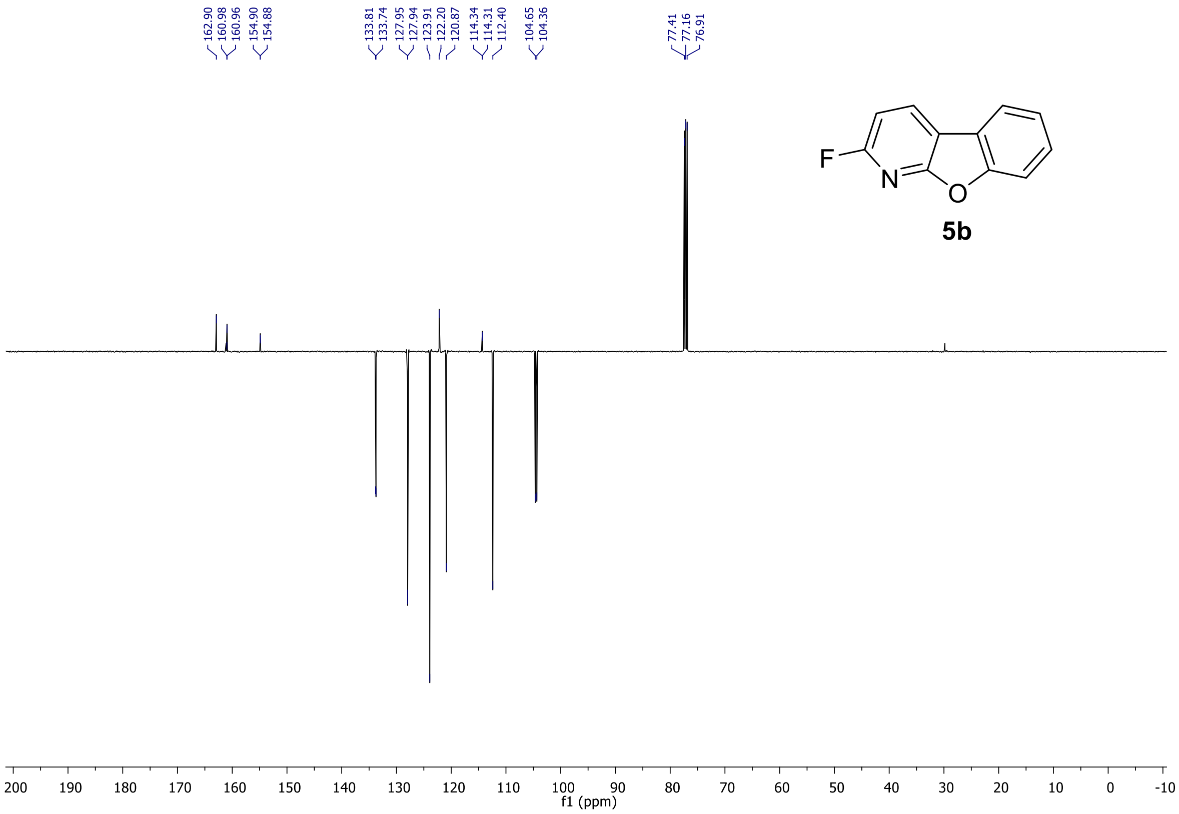

$^{19}F\{^{1}H\}$ NMR (376 MHz, $CDCl_3$)

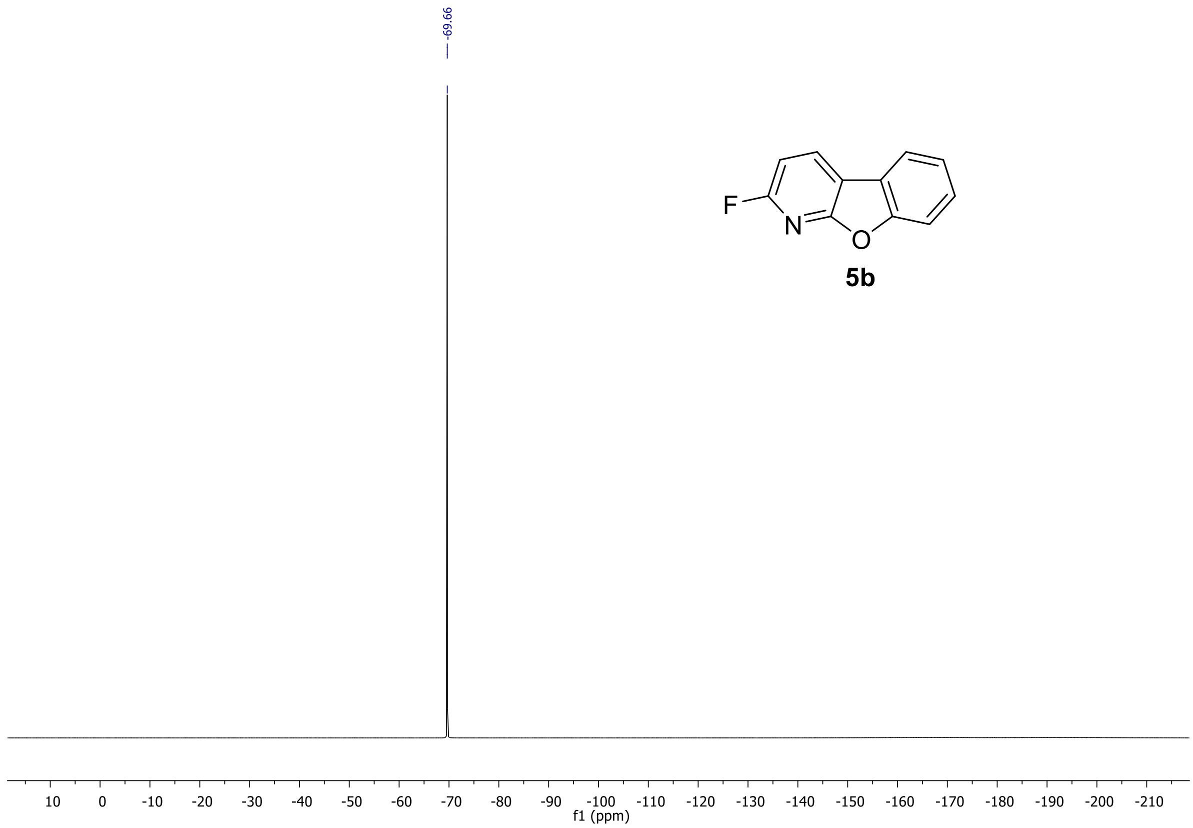

^{1}H NMR (500 MHz, $CDCl_3$)

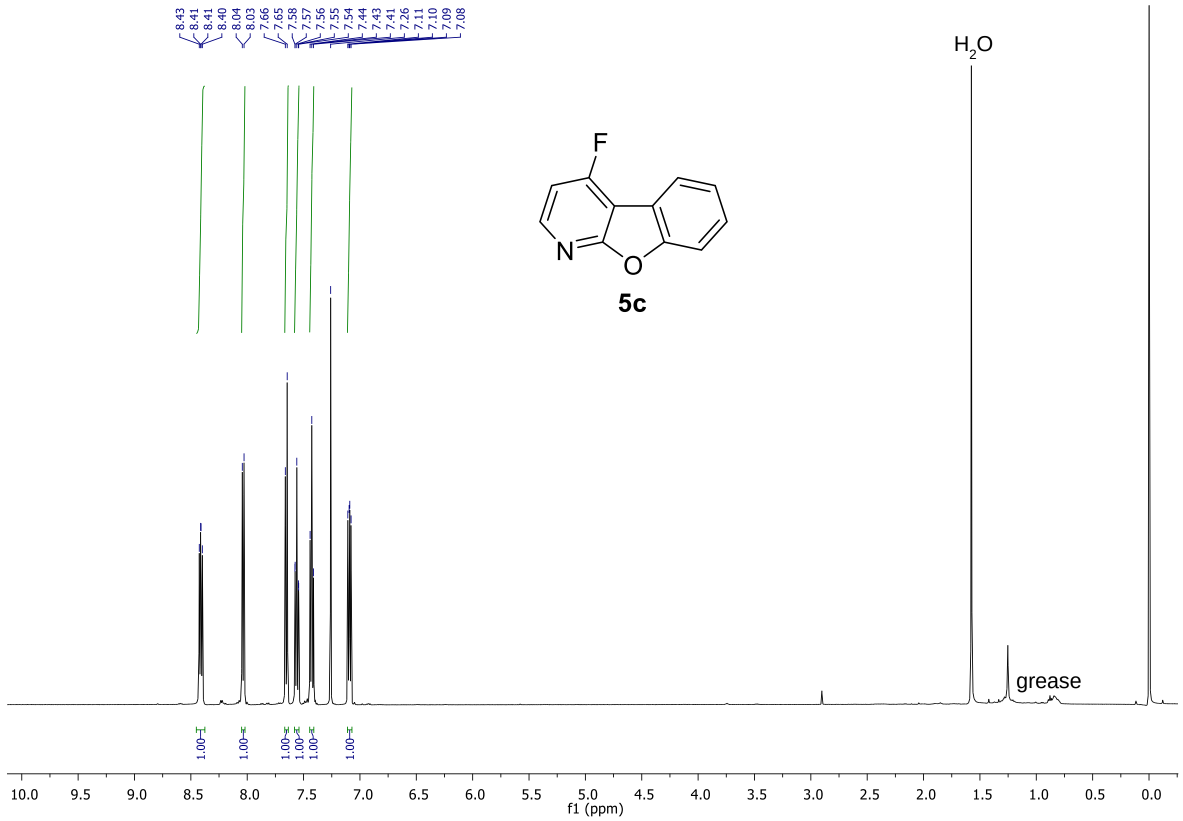

$^{13}C\{^{1}H\}$ NMR (126 MHz, $CDCl_3$)

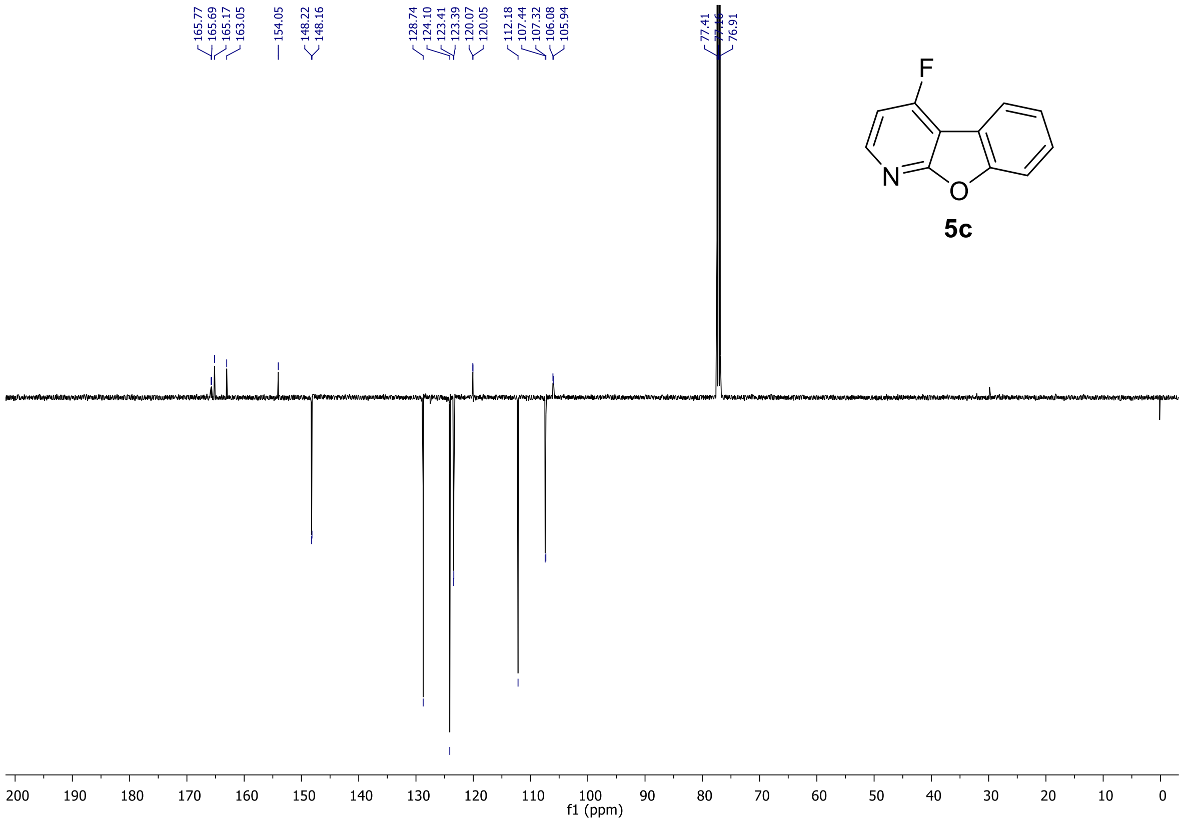

$^{19}F\{^{1}H\}$ NMR (376 MHz, $CDCl_3$)

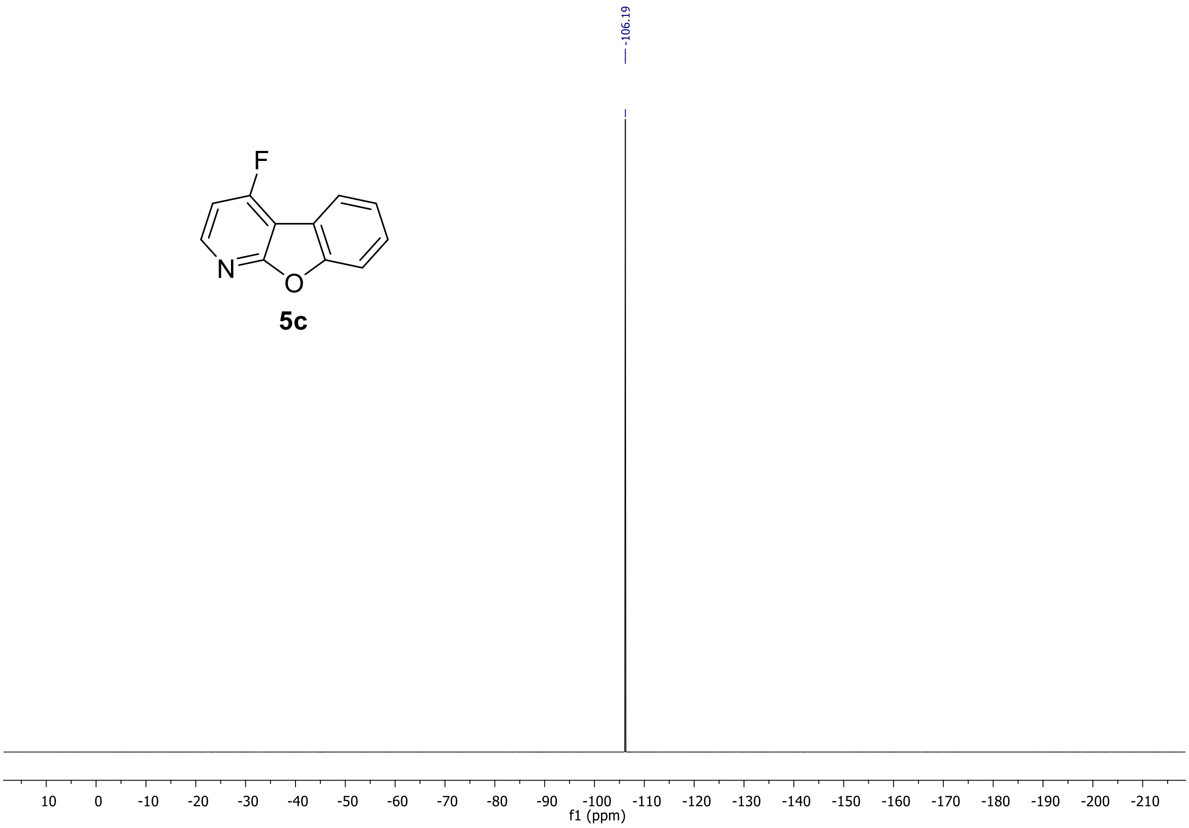

^{1}H NMR (500 MHz, $CDCl_3$)

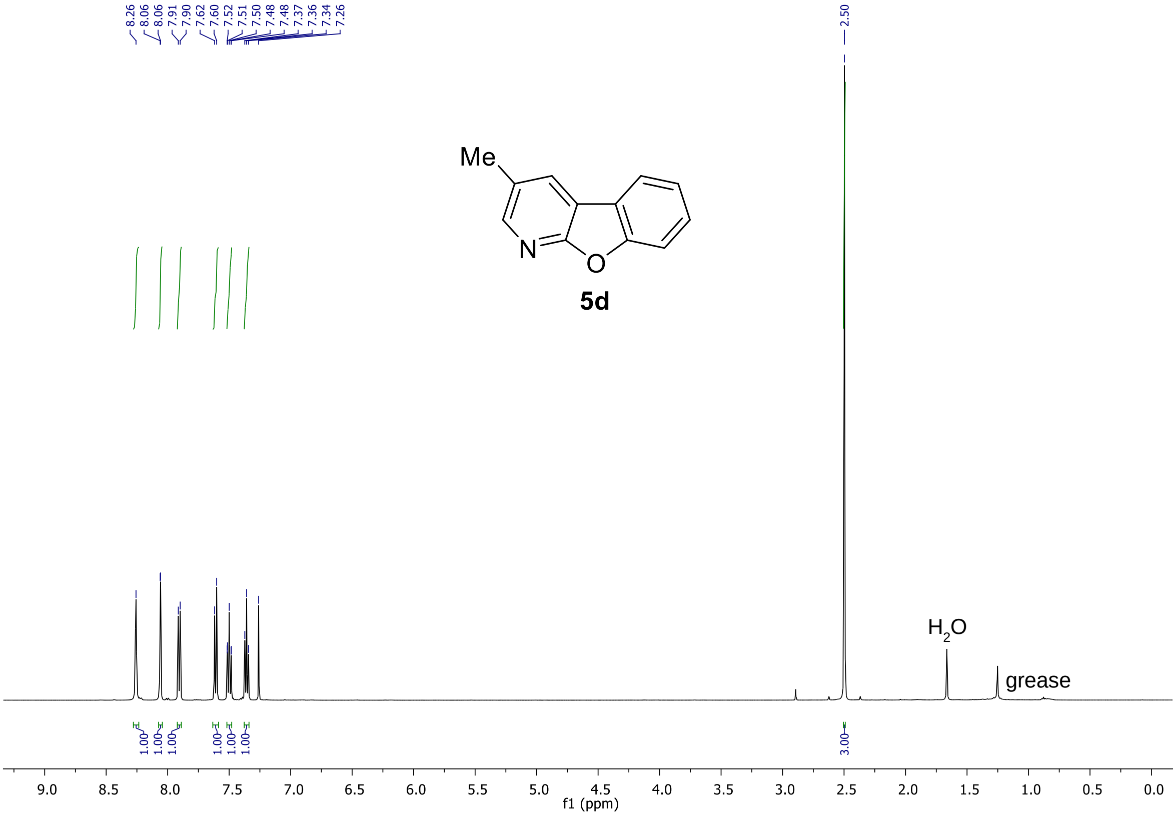

$^{13}C\{^{1}H\}$ NMR (126 MHz, $CDCl_3$)

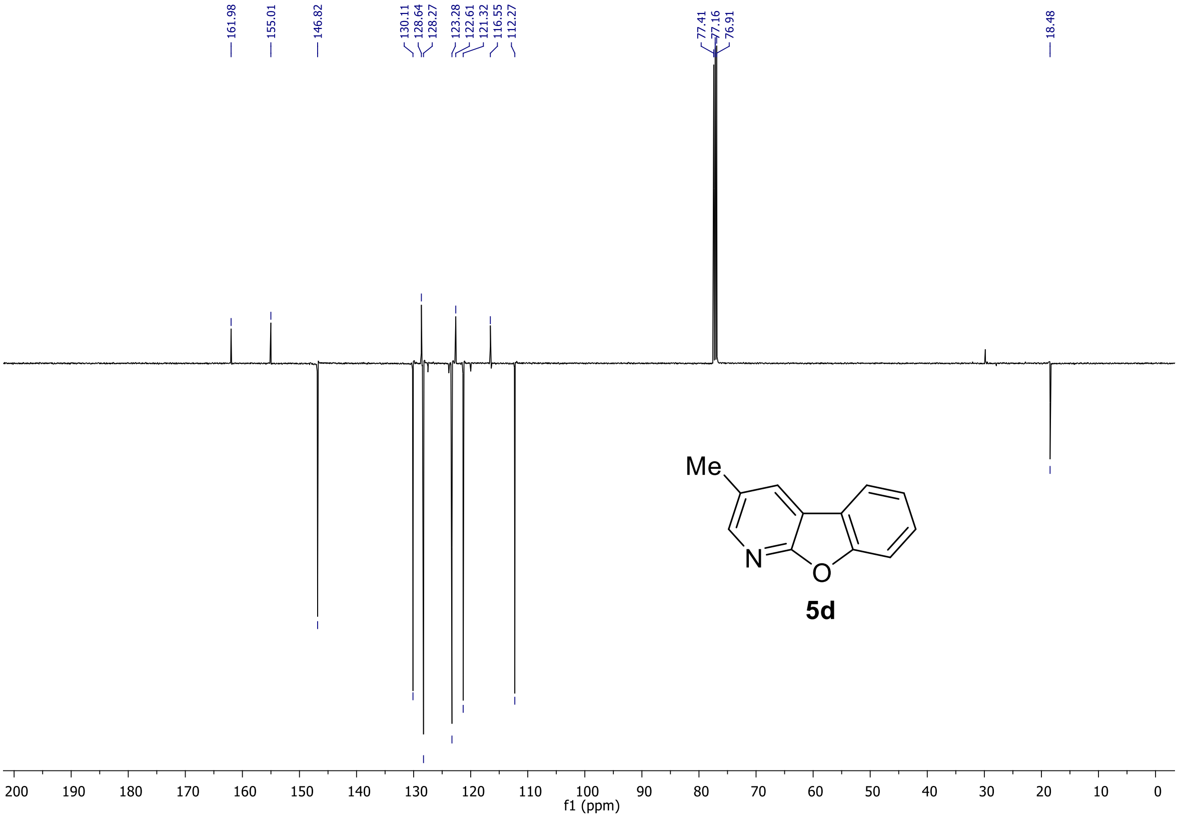

^{1}H NMR (500 MHz, $CDCl_3$)

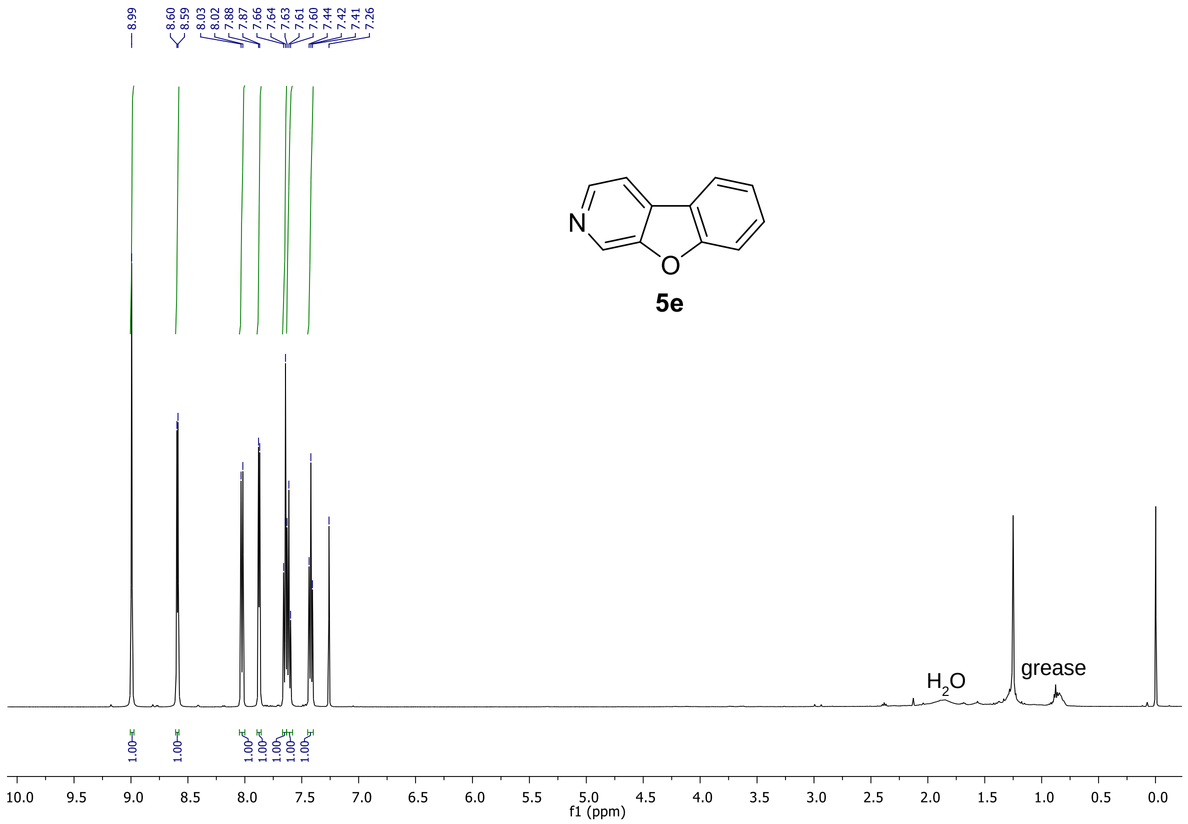

$^{13}C\{^{1}H\}$ NMR (126 MHz, $CDCl_3$)

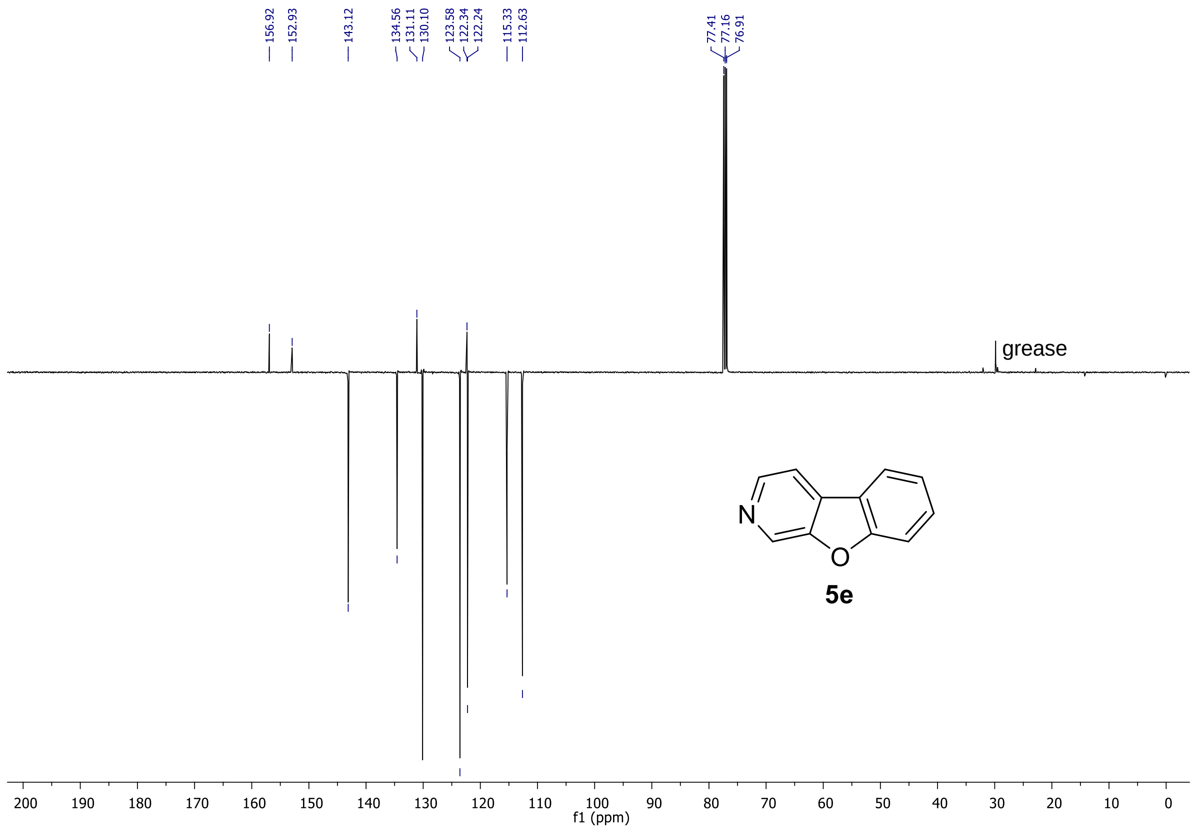

^{1}H NMR (400 MHz, $CDCl_3$)

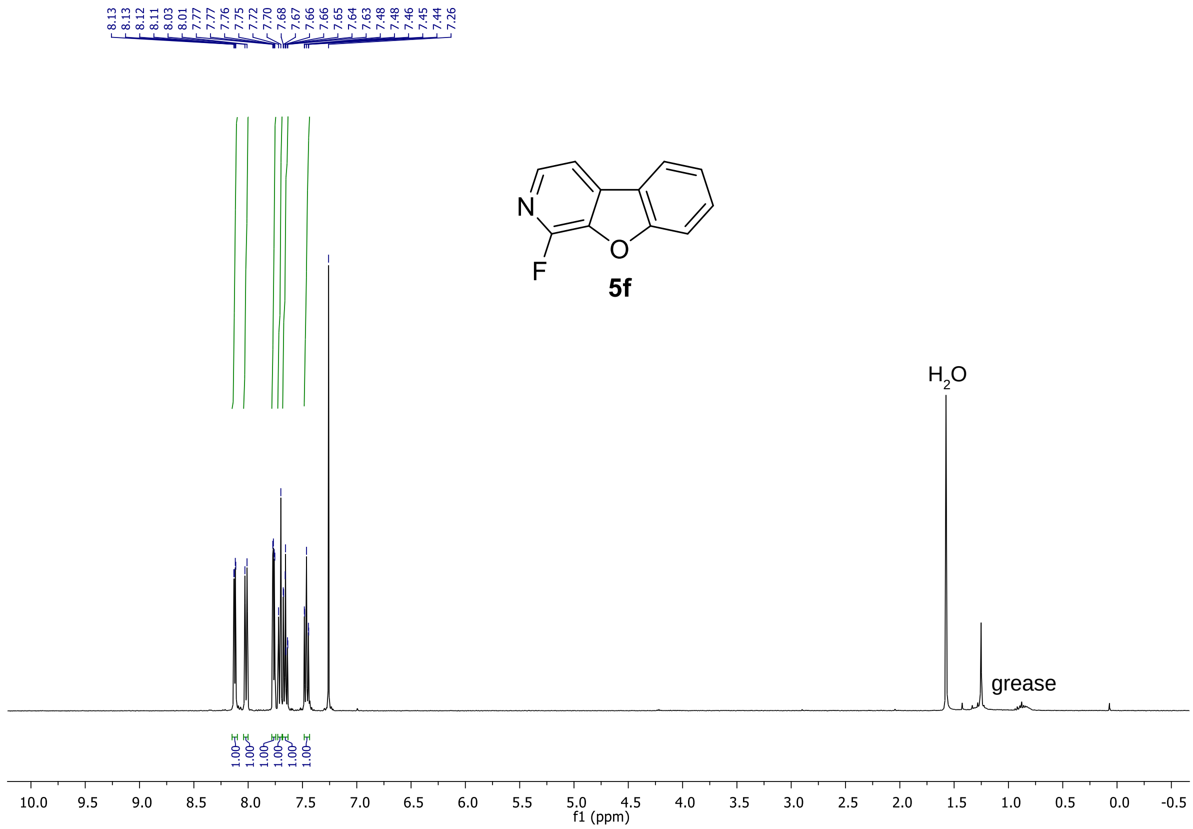

$^{13}C\{^{1}H\}$ NMR (126 MHz, $CDCl_3$)

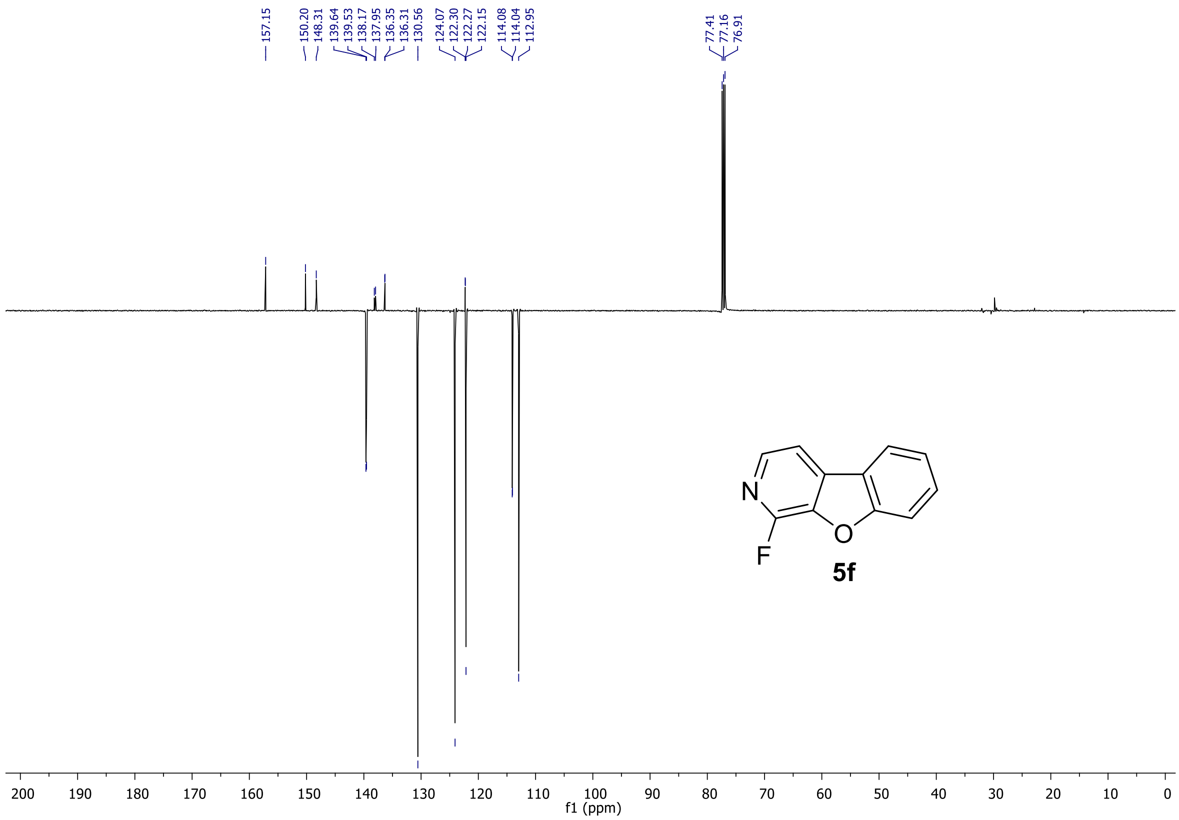

$^{19}F\{^{1}H\}$ NMR (376 MHz, $CDCl_3$)

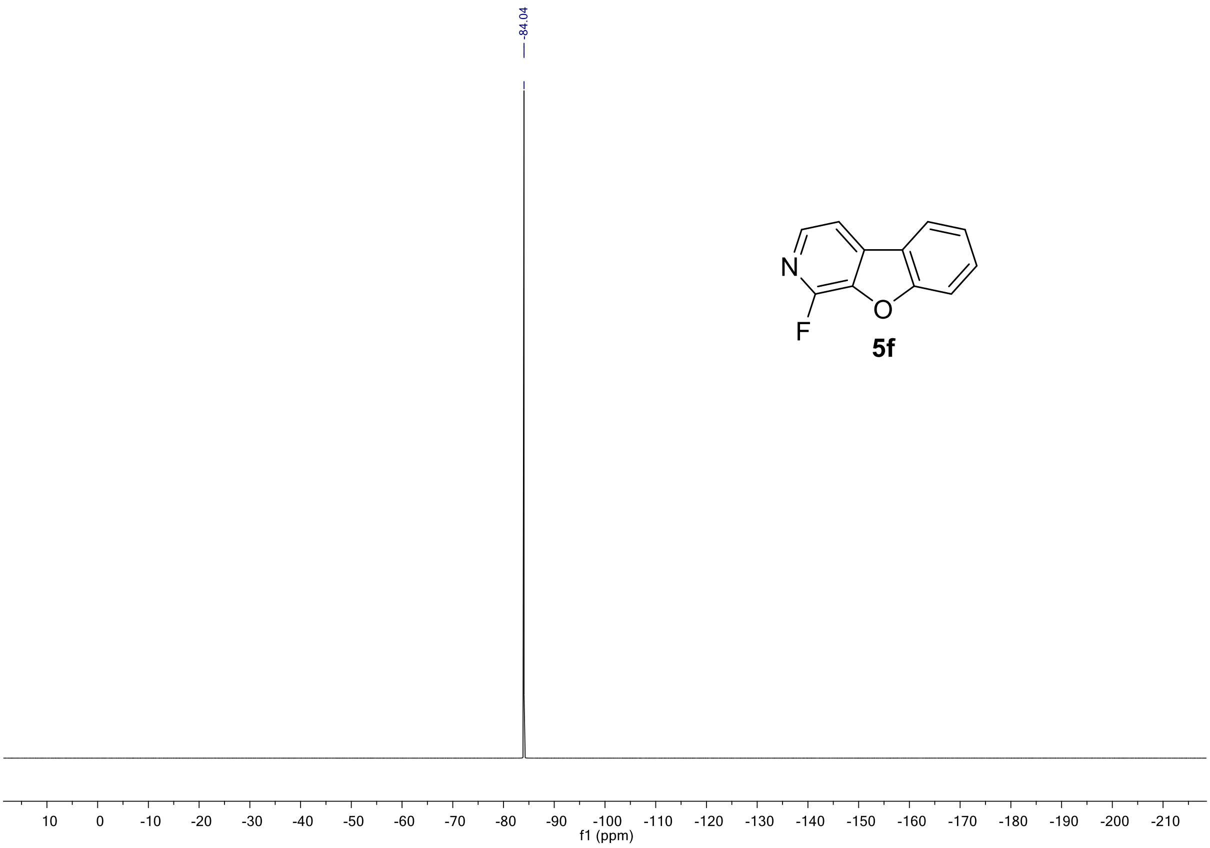

^{1}H NMR (500 MHz, $CDCl_3$)

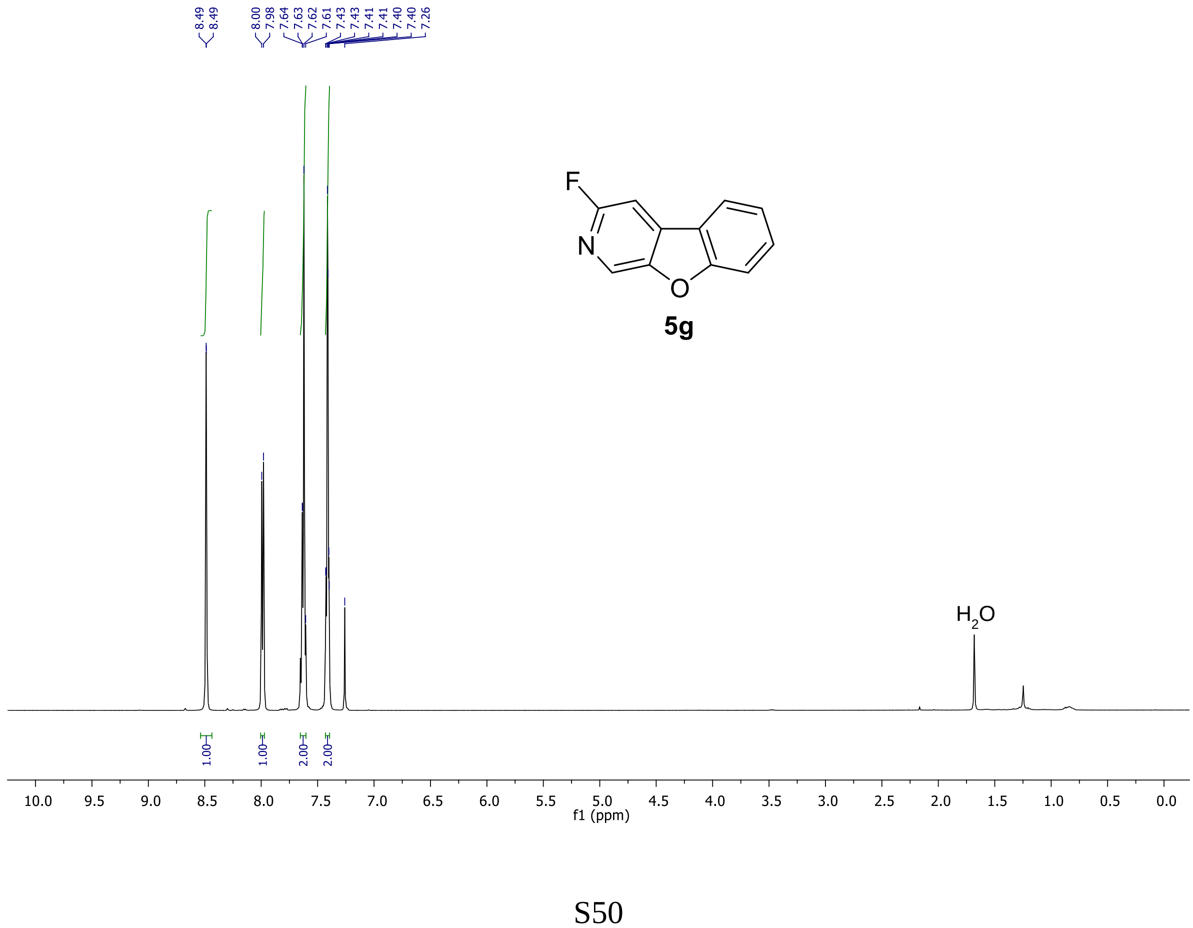

$^{13}C\{^{1}H\}$ NMR (126 MHz, $CDCl_3$)

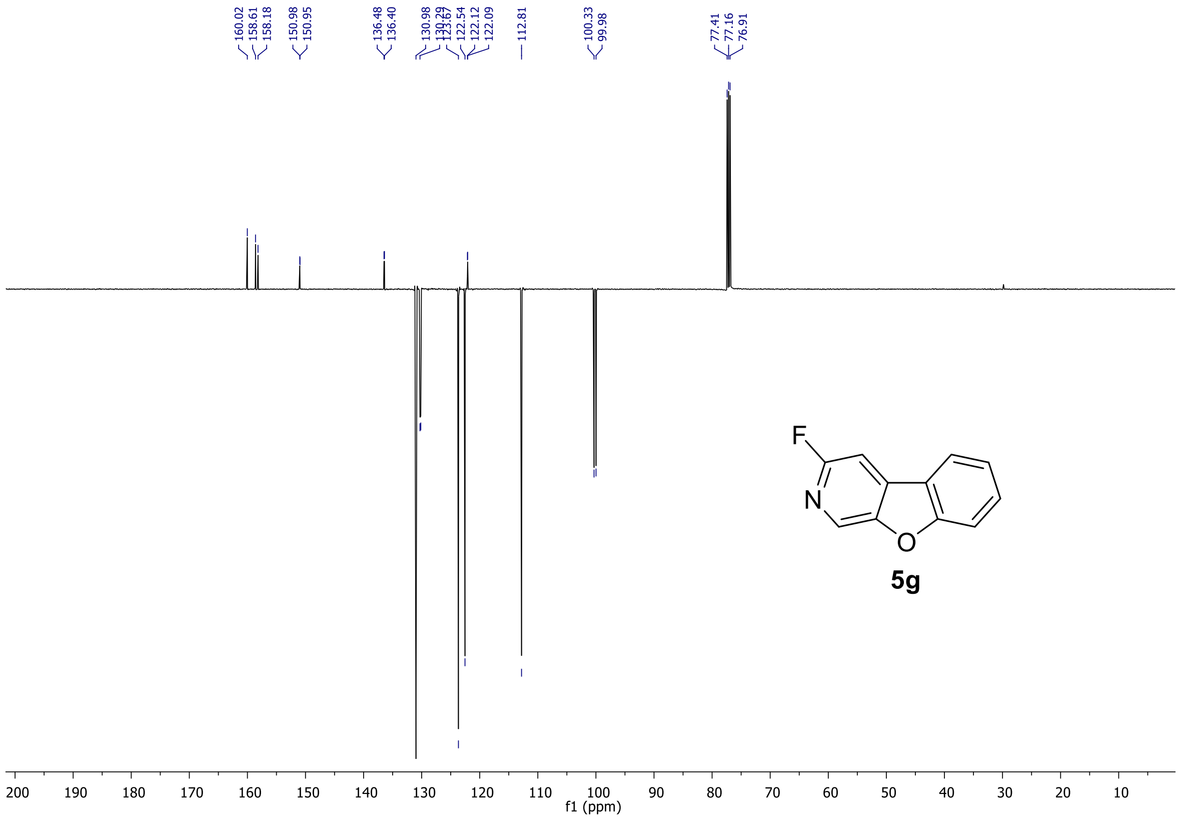

$^{19}F\{^{1}H\}$ NMR (376 MHz, $CDCl_3$)

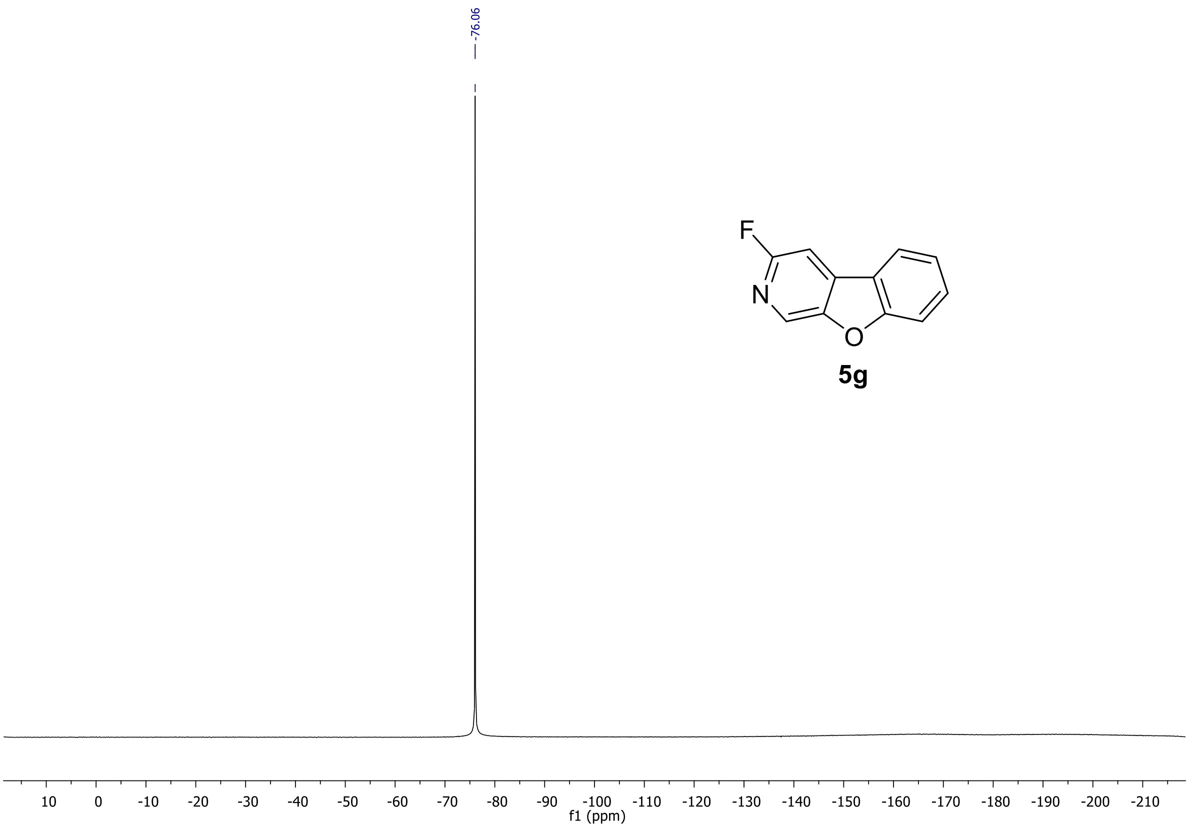

^{1}H NMR (500 MHz, $CDCl_3$)

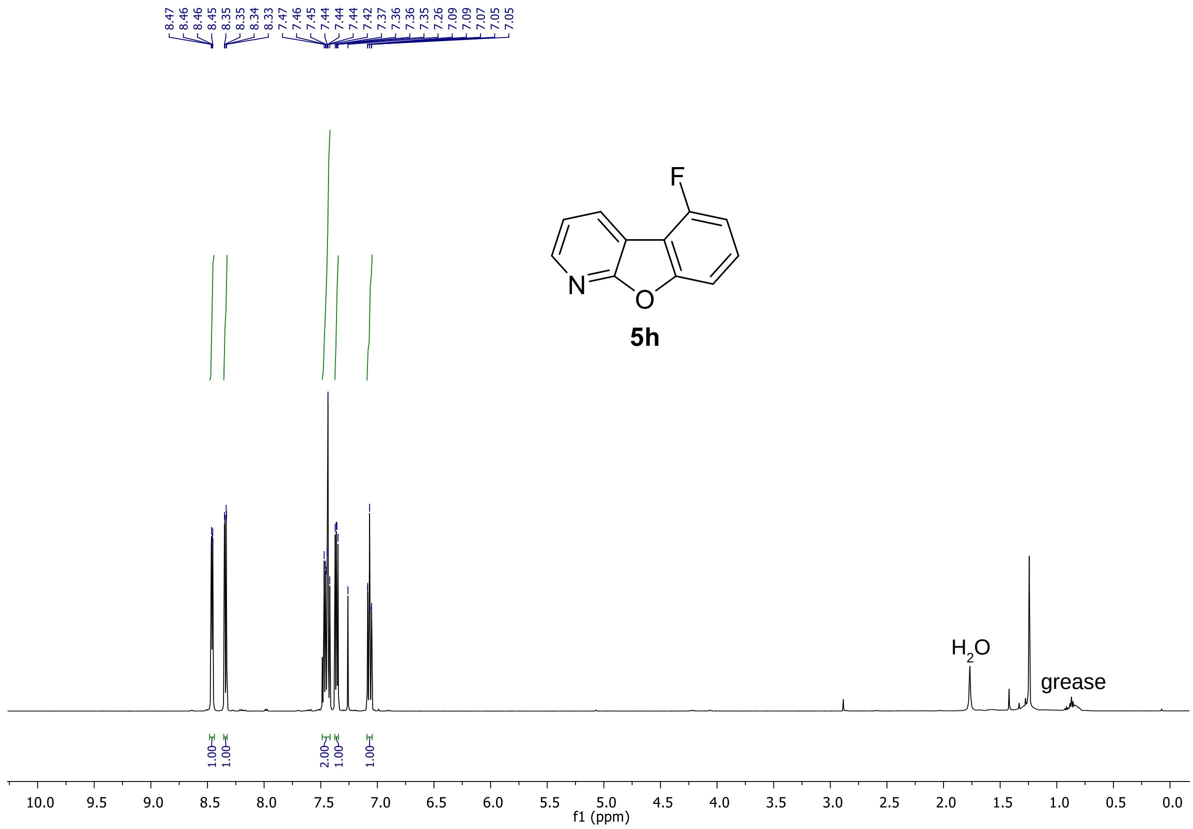

$^{13}C\{^{1}H\}$ NMR (126 MHz, $CDCl_3$)

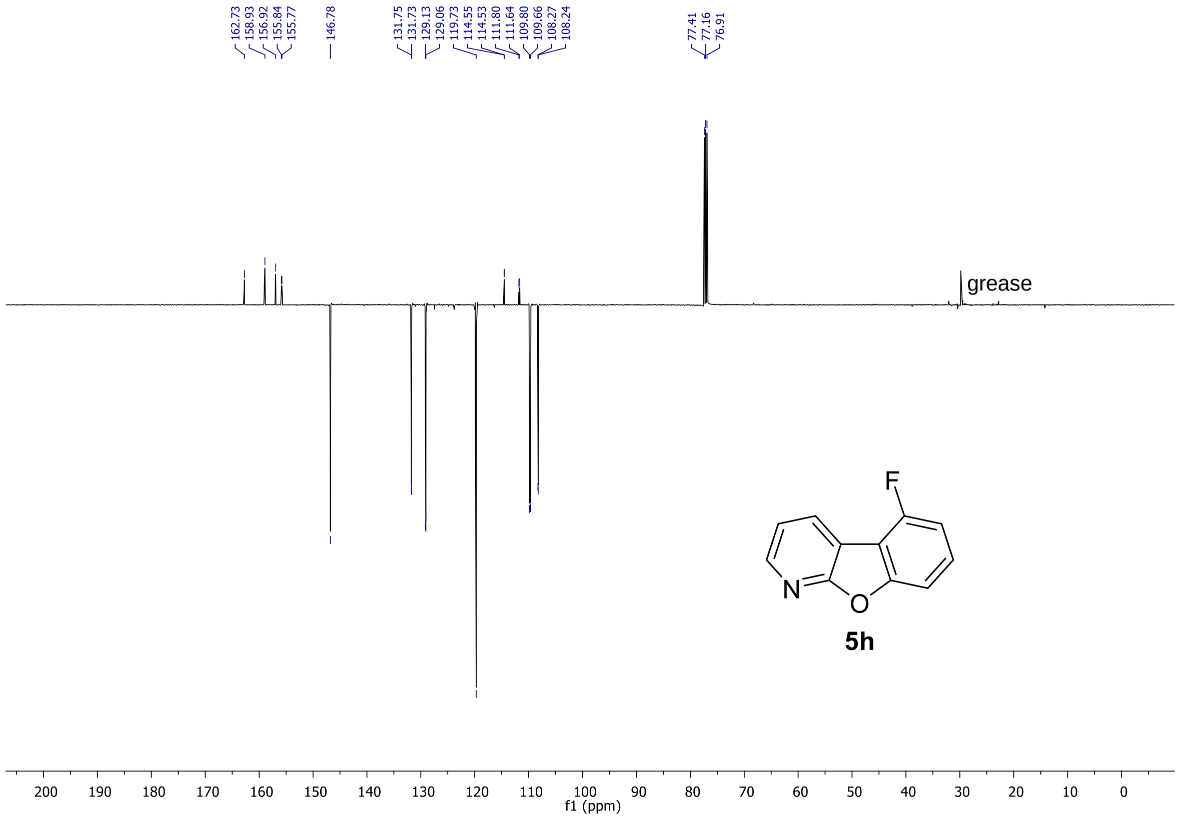

$^{19}F\{^{1}H\}$ NMR (376 MHz, $CDCl_3$)

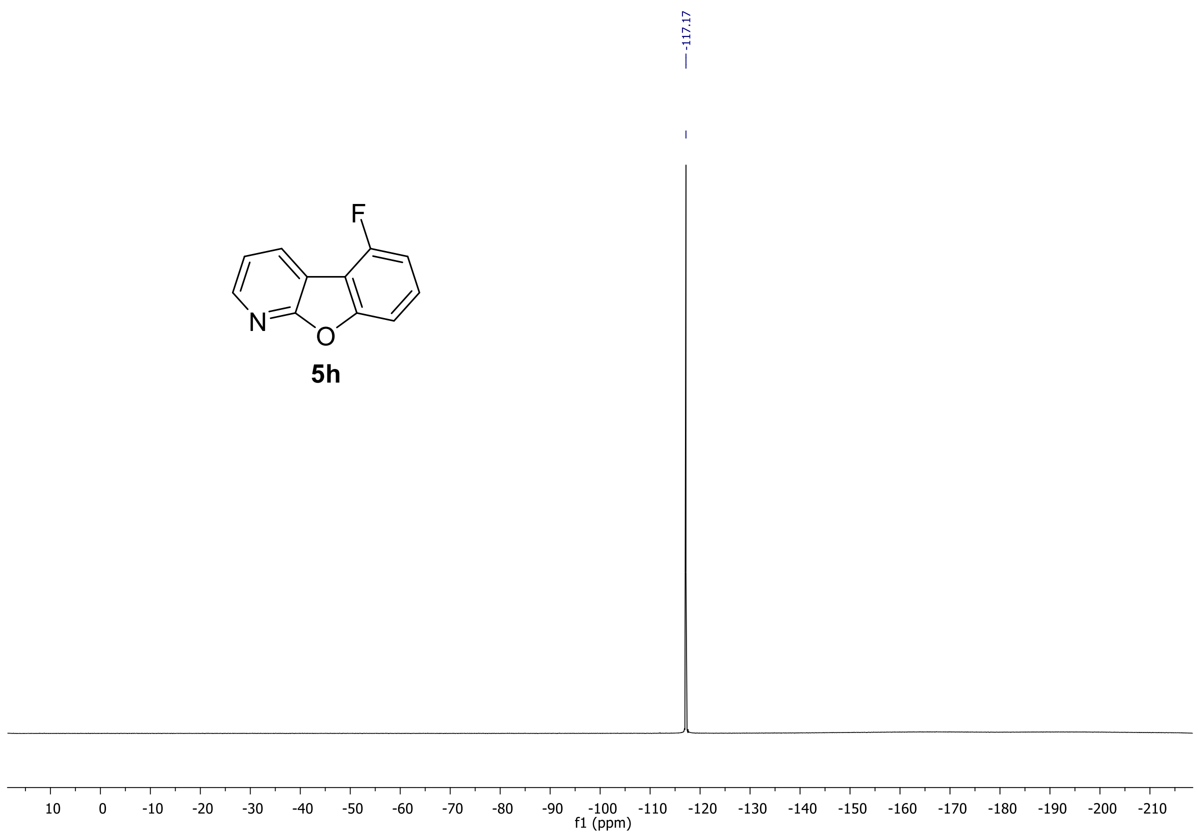

^{1}H NMR (500 MHz, $CDCl_3$)

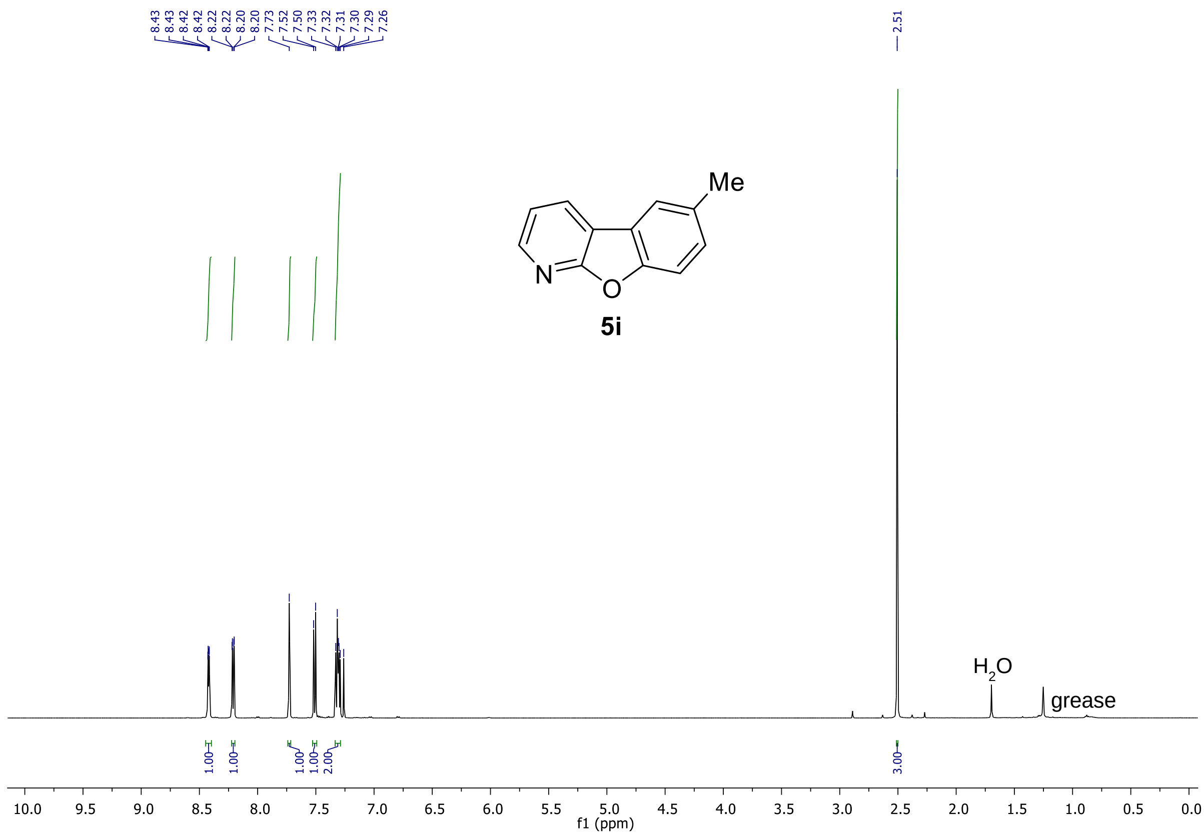

$^{13}C\{^1H\}$ NMR (126 MHz, $CDCl_3$)

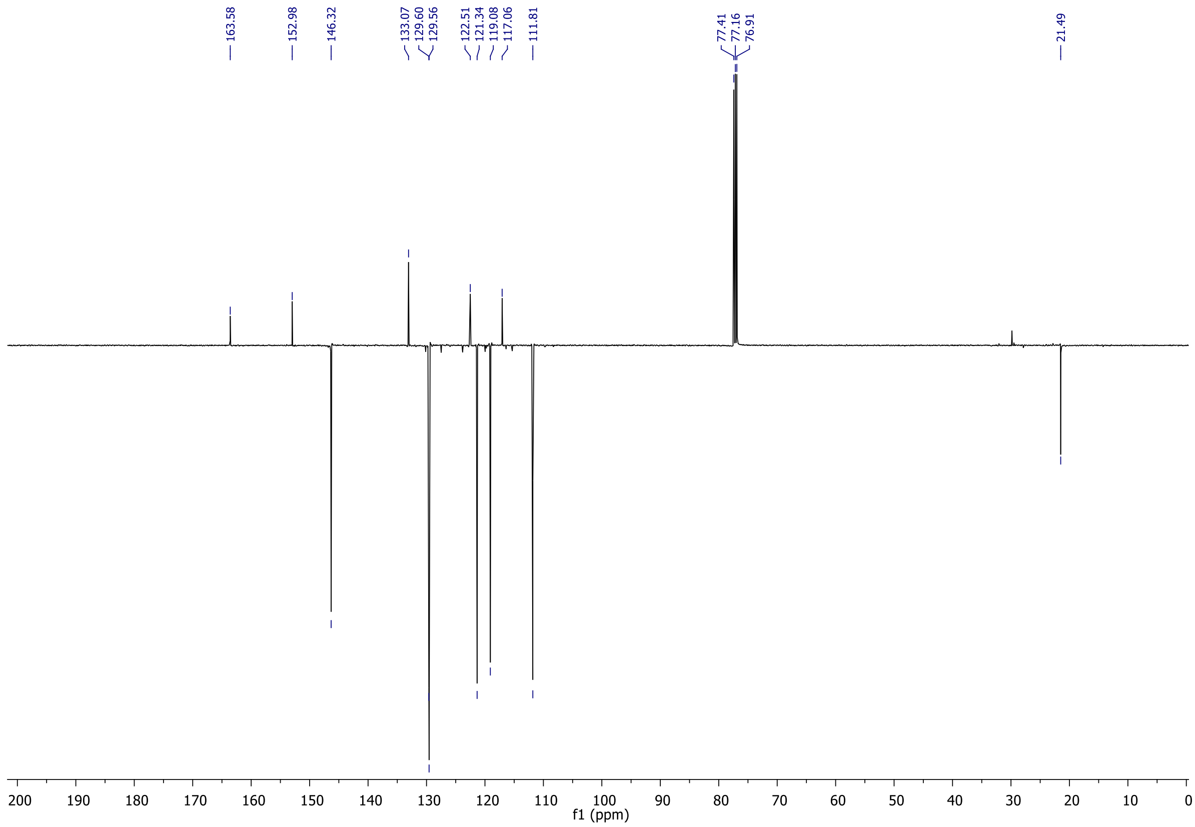

^1H NMR (400 MHz, $CDCl_3$)

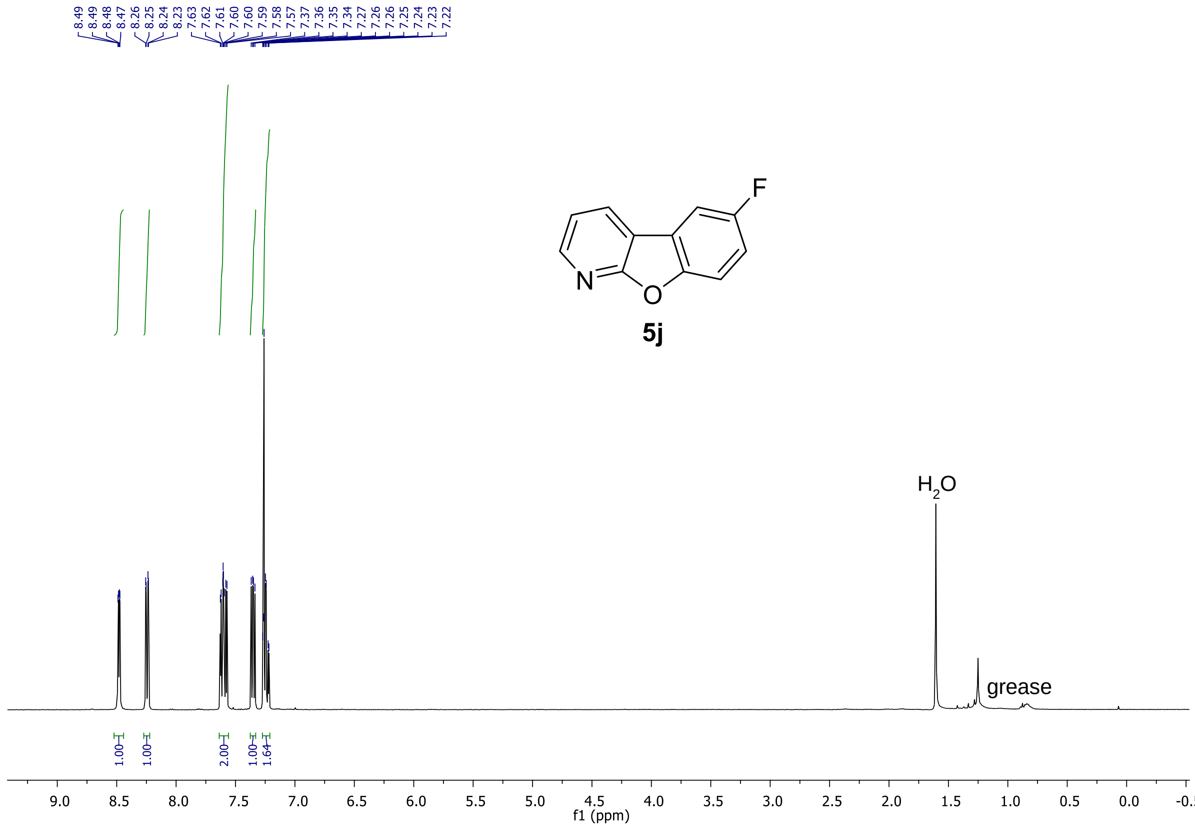

$^{13}C\{^{1}H\}$ NMR (126 MHz, $CDCl_3$)

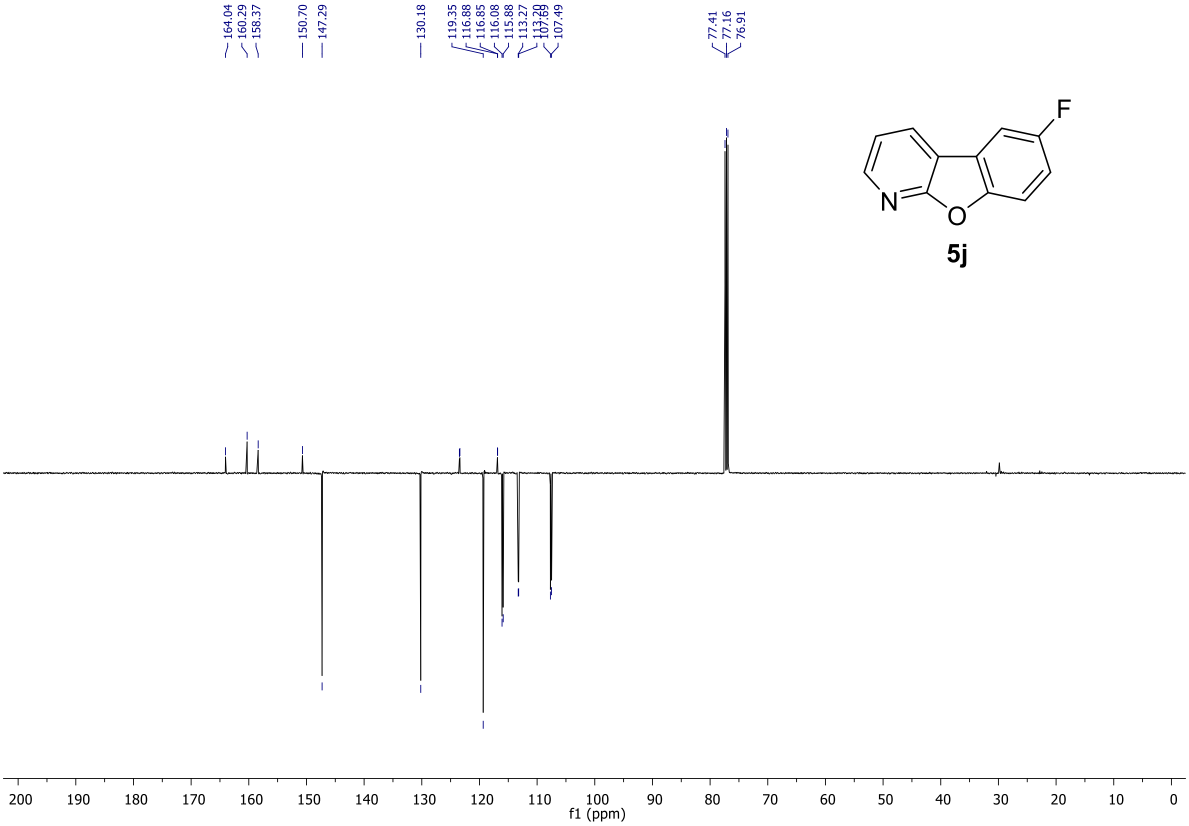

$^{19}F\{^{1}H\}$ NMR (376 MHz, $CDCl_3$)

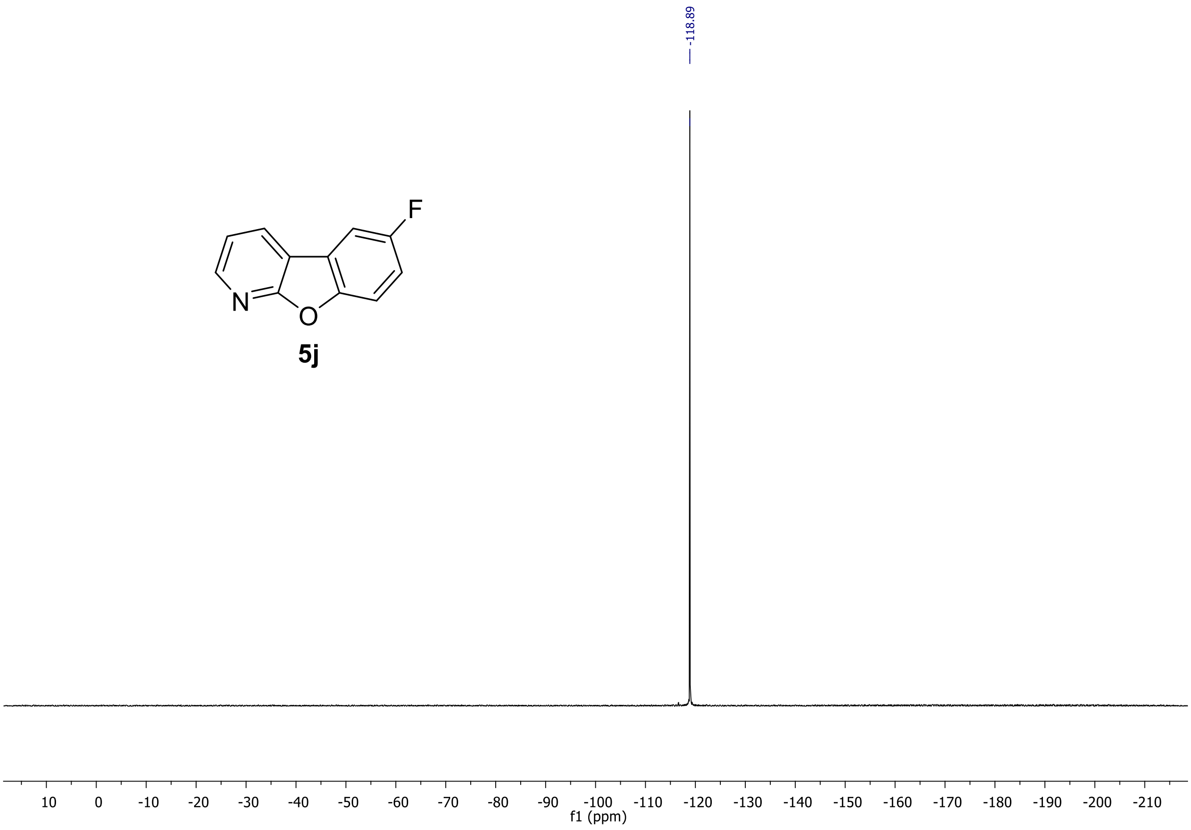

^{1}H NMR (500 MHz, $CDCl_3$)

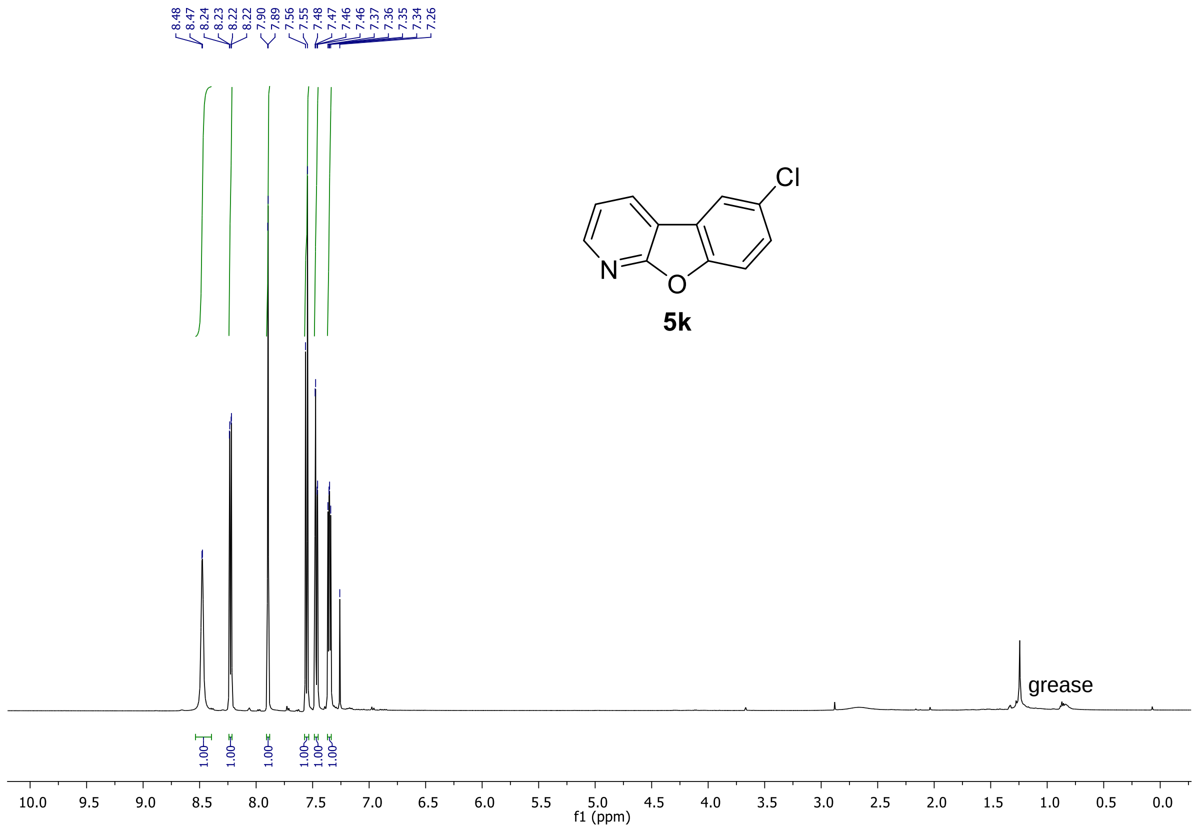

^{13}C{^{1}H} NMR (126 MHz, $CDCl_3$)

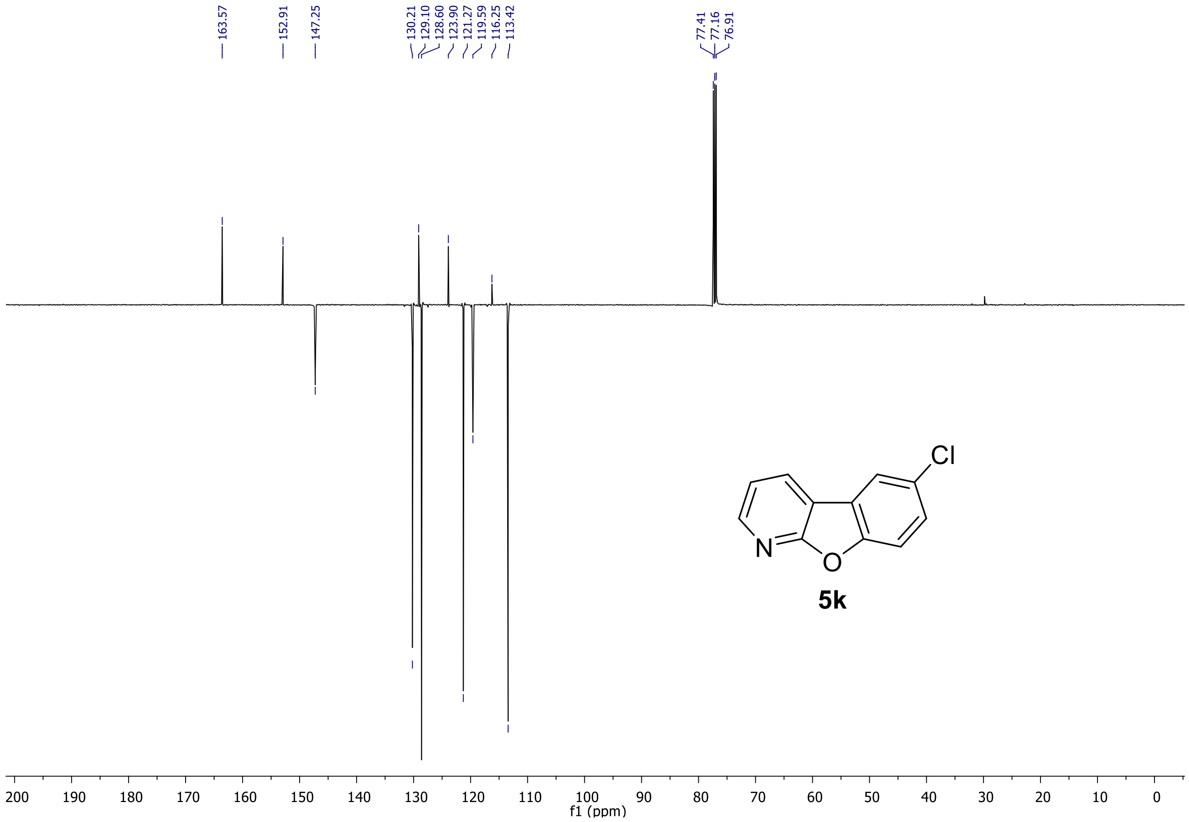

^{1}H NMR (500 MHz, $CDCl_3$)

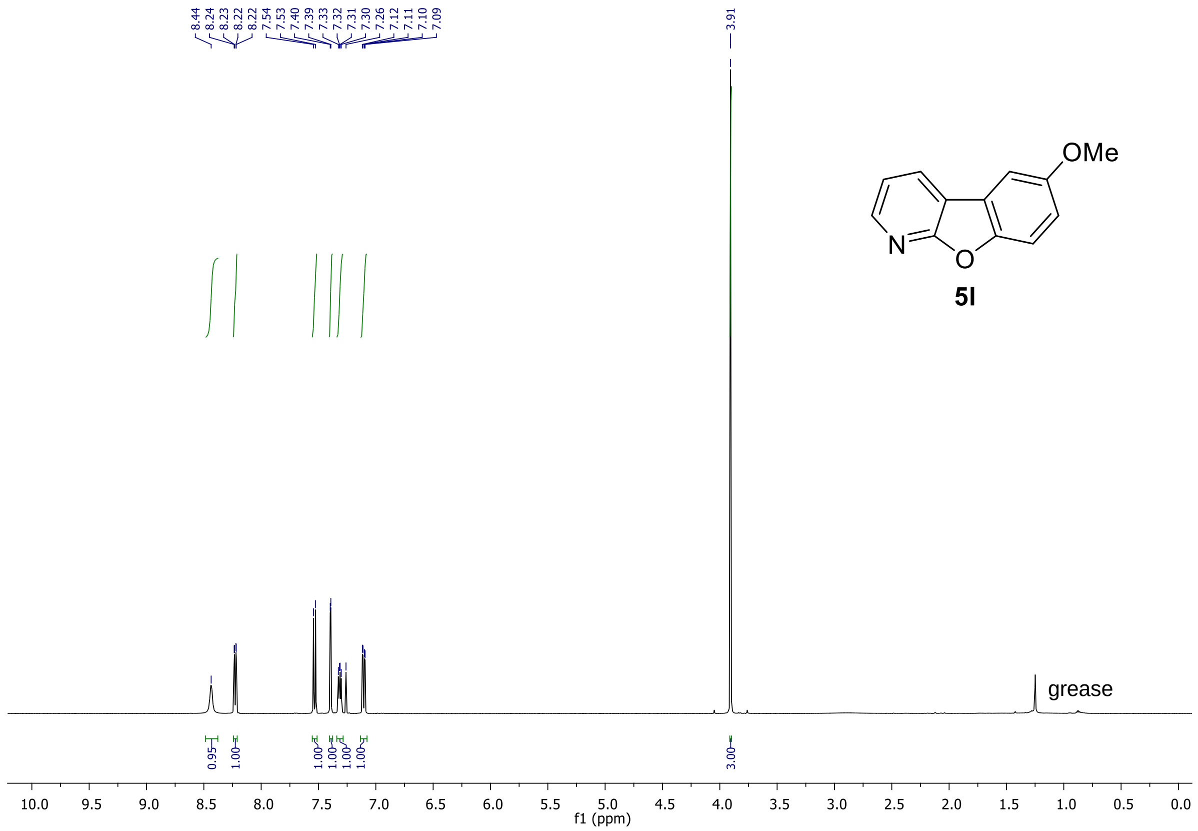

$^{13}C\{^{1}H\}$ NMR (126 MHz, $CDCl_3$)

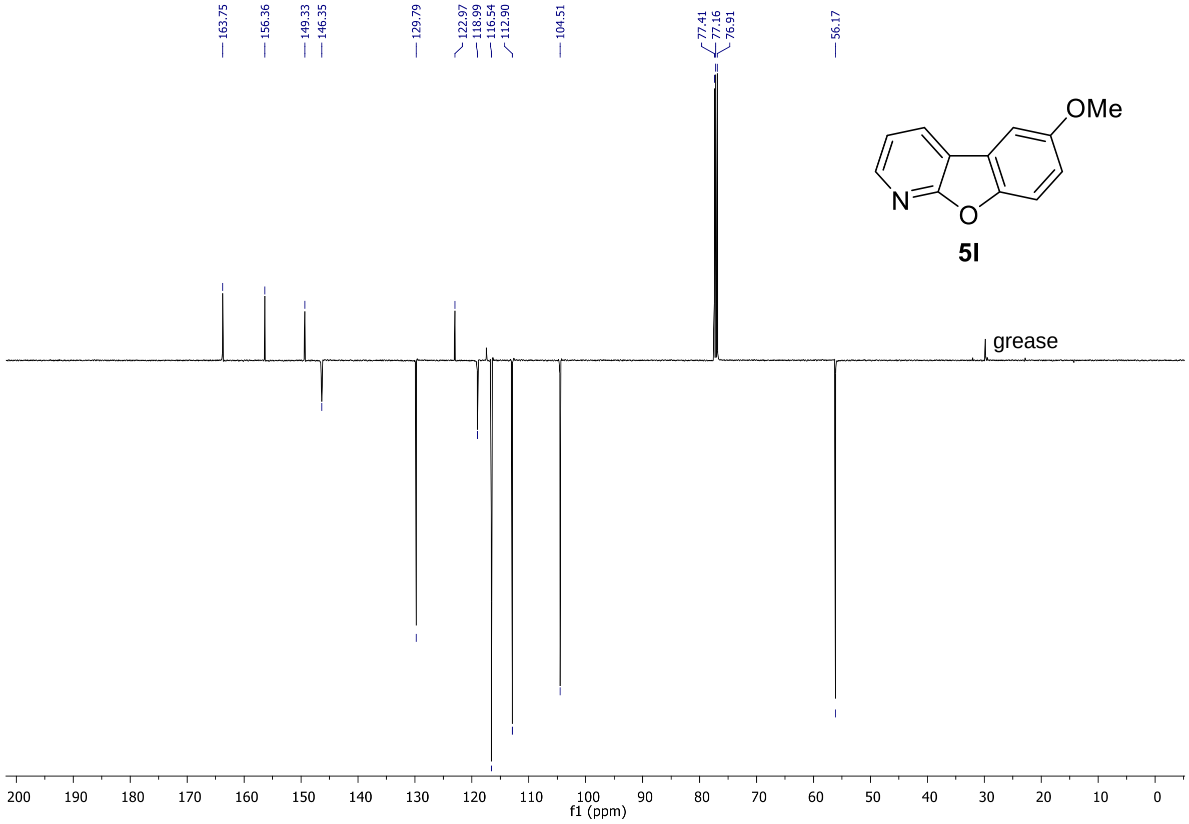

^{1}H NMR (400 MHz, DMSO-*d6*)

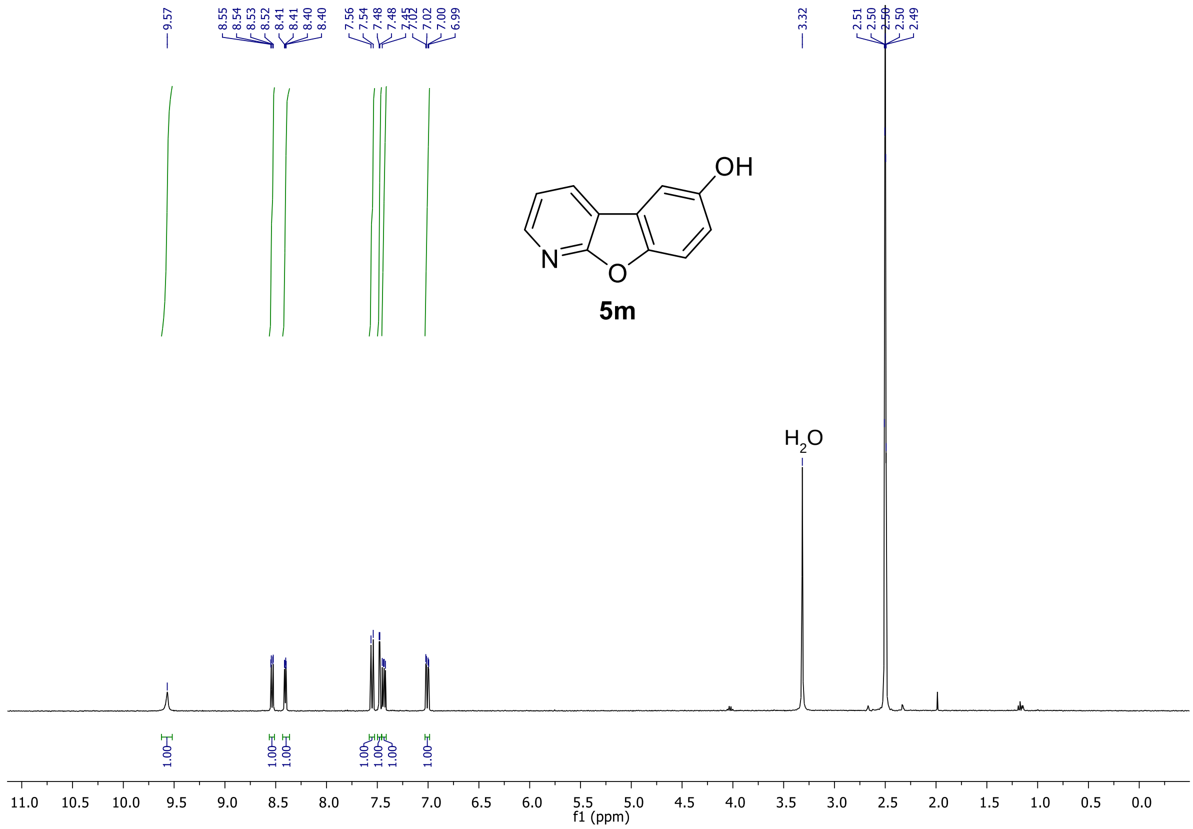

$^{13}C\{^{1}H\}$ NMR (126 MHz, DMSO-*d6*)

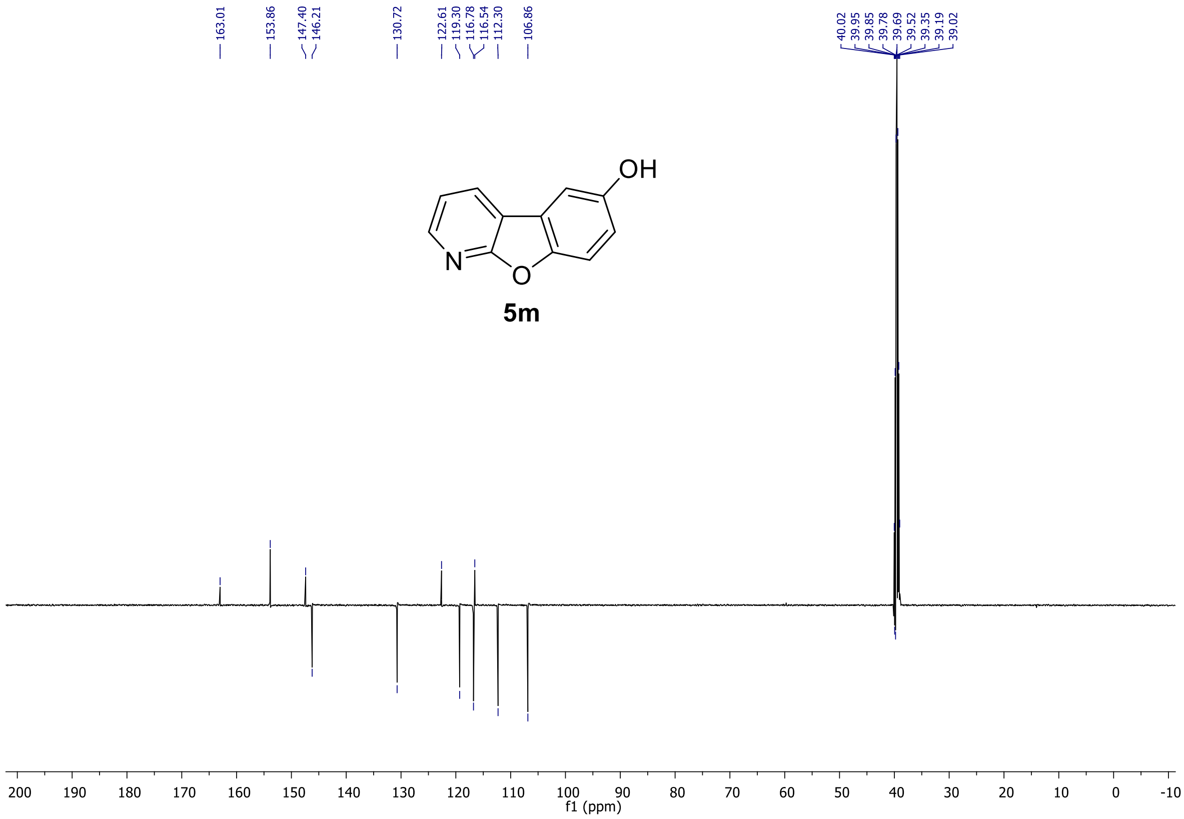

^{1}H NMR (500 MHz, $CDCl_3$)

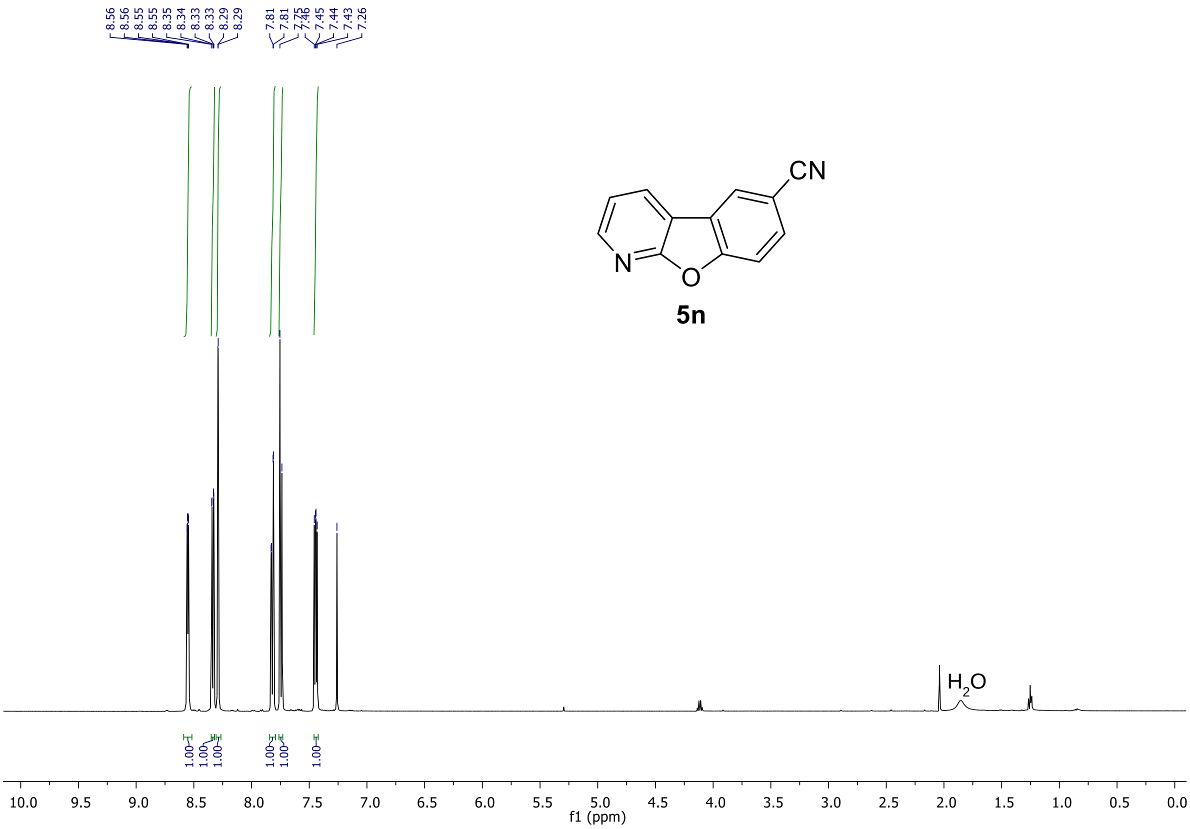

$^{13}C\{^{1}H\}$ NMR (126 MHz, $CDCl_3$)

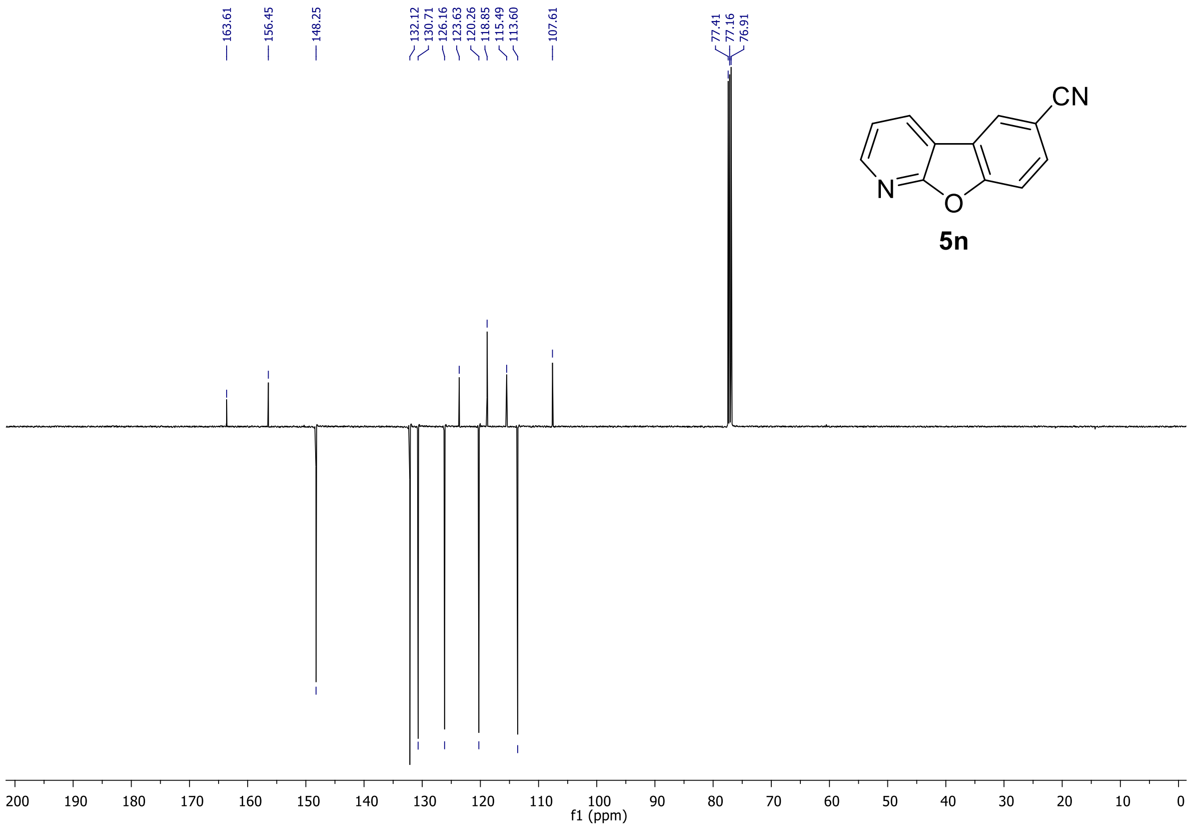

^{1}H NMR (500 MHz, $CDCl_3$)

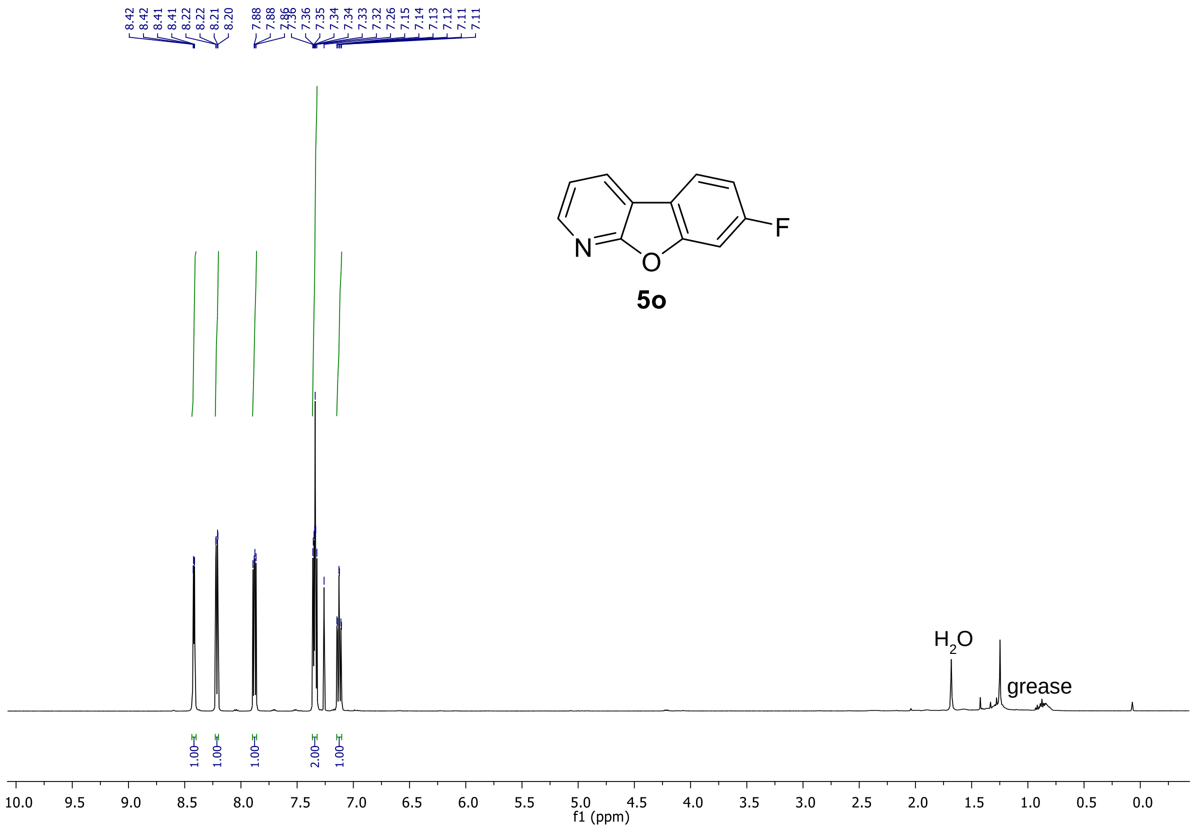

$^{13}C\{^{1}H\}$ NMR (126 MHz, $CDCl_3$)

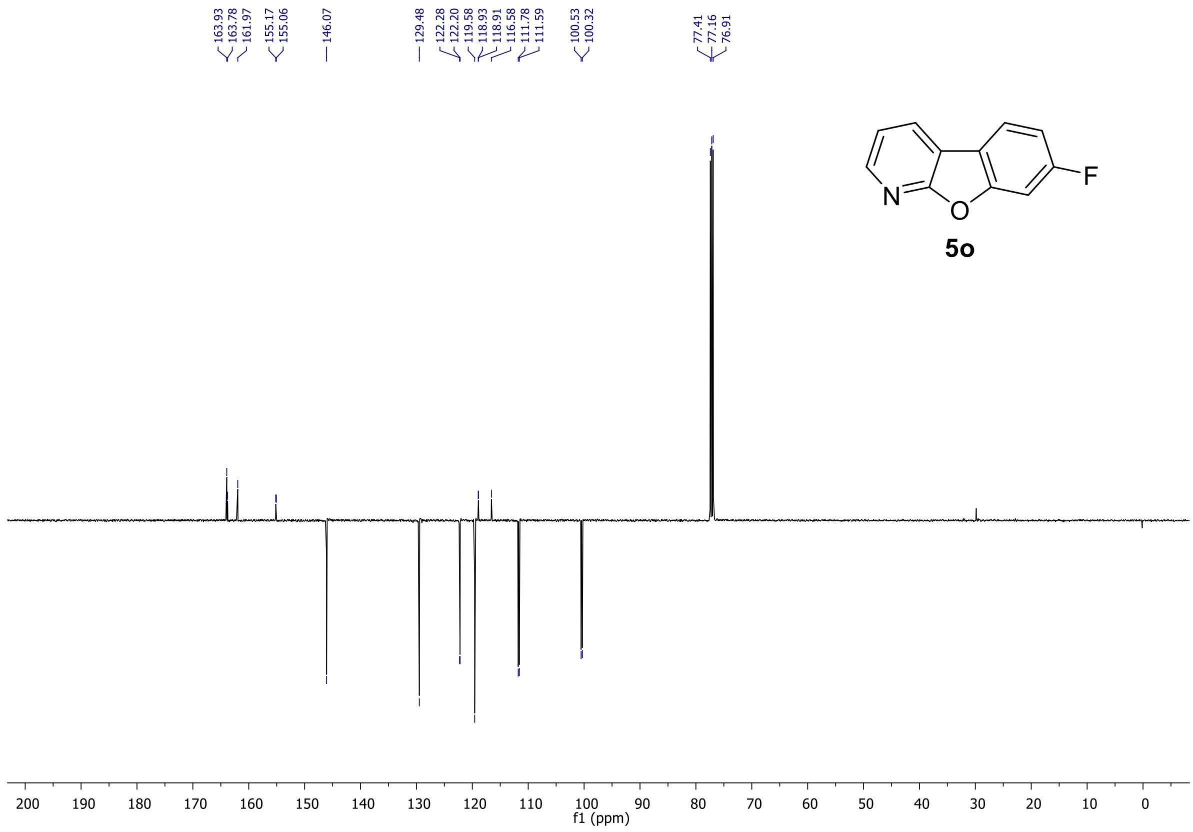

$^{19}F\{^{1}H\}$ NMR (376 MHz, $CDCl_3$)

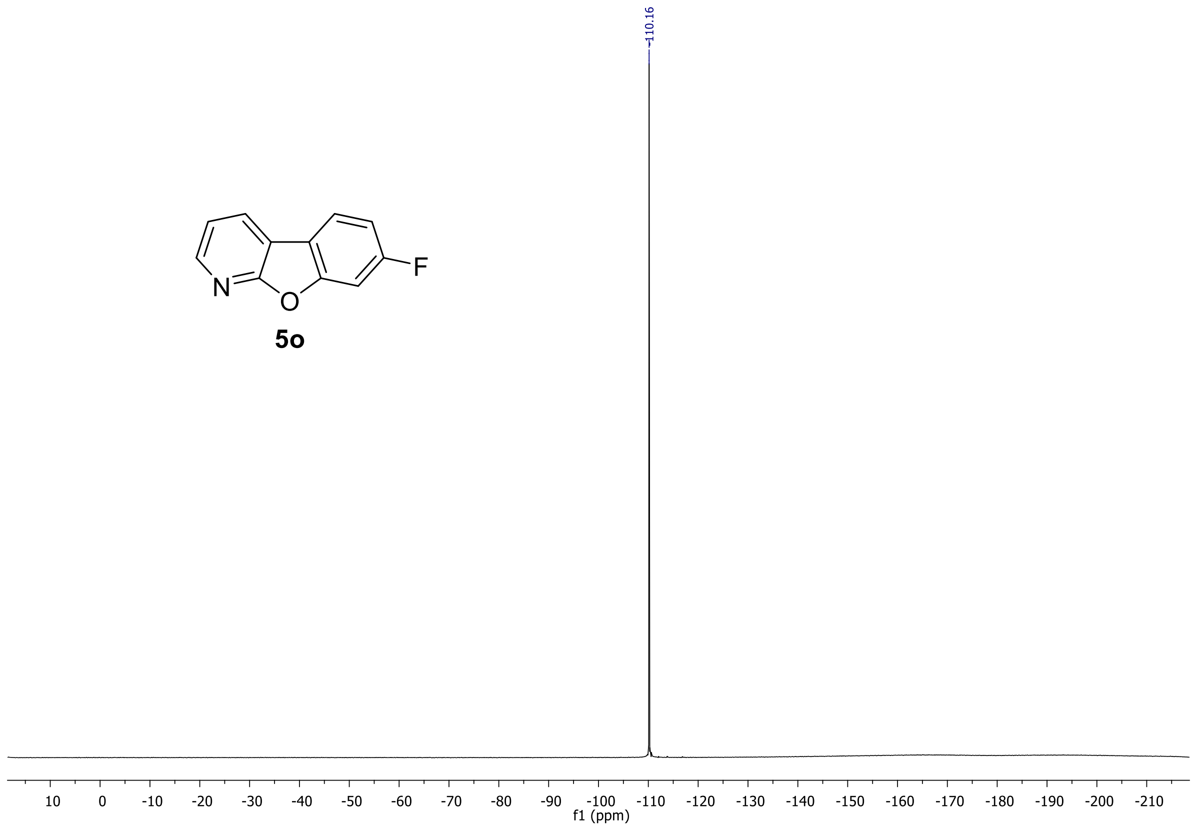

^{1}H NMR (500 MHz, $CDCl_3$)

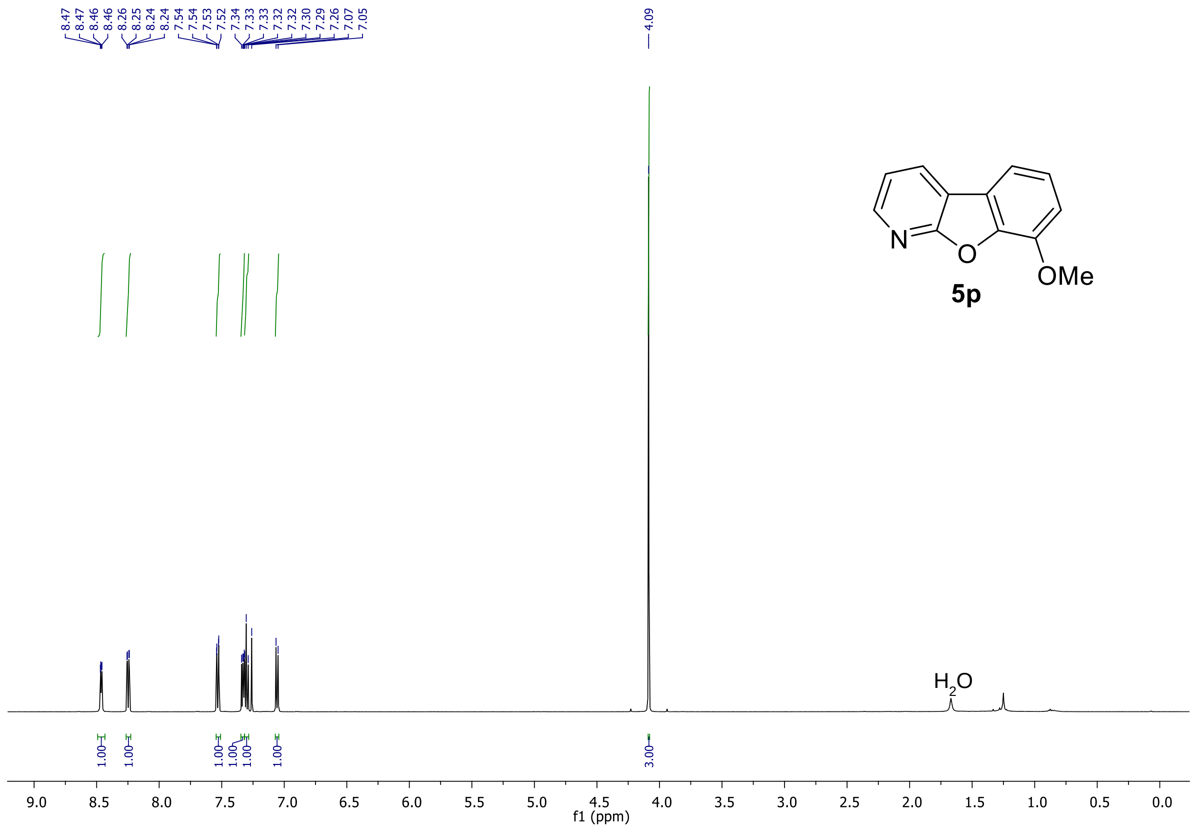

$^{13}C\{^{1}H\}$ NMR (126 MHz, $CDCl_3$)

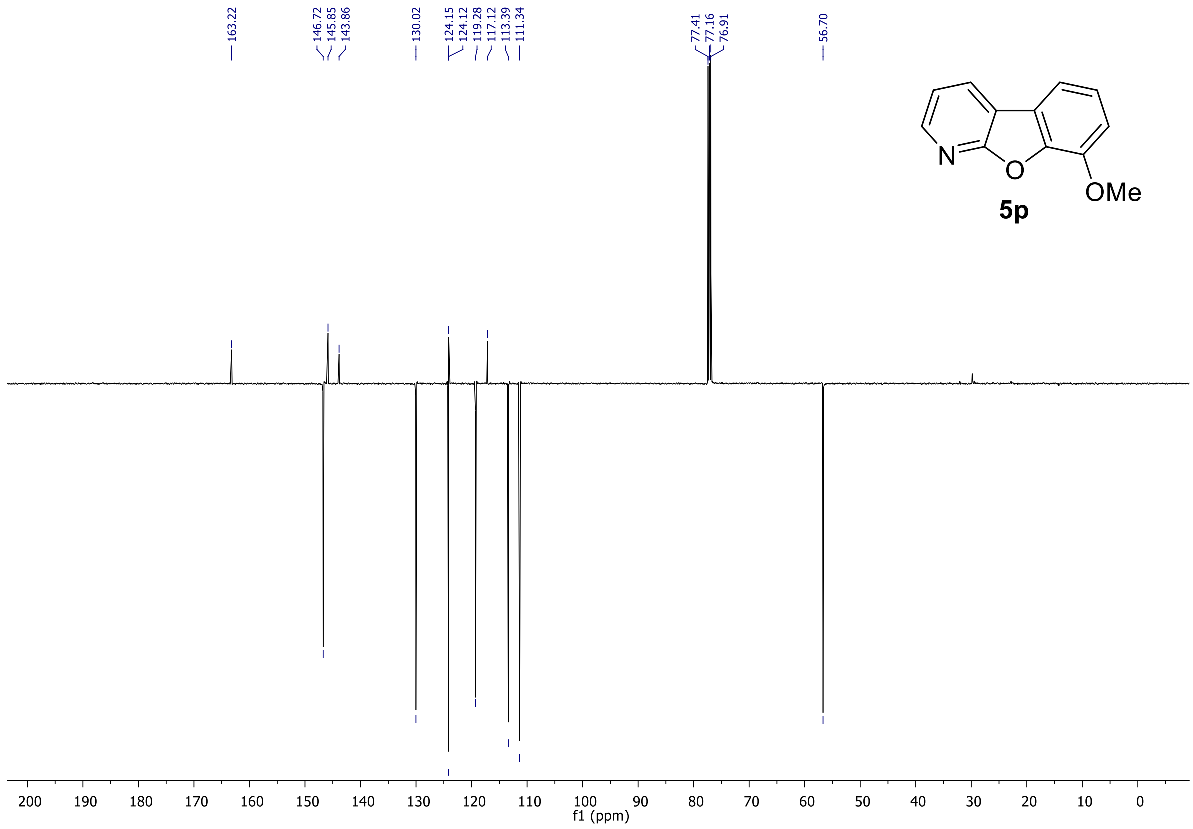

^{1}H NMR (500 MHz, $CDCl_3$)

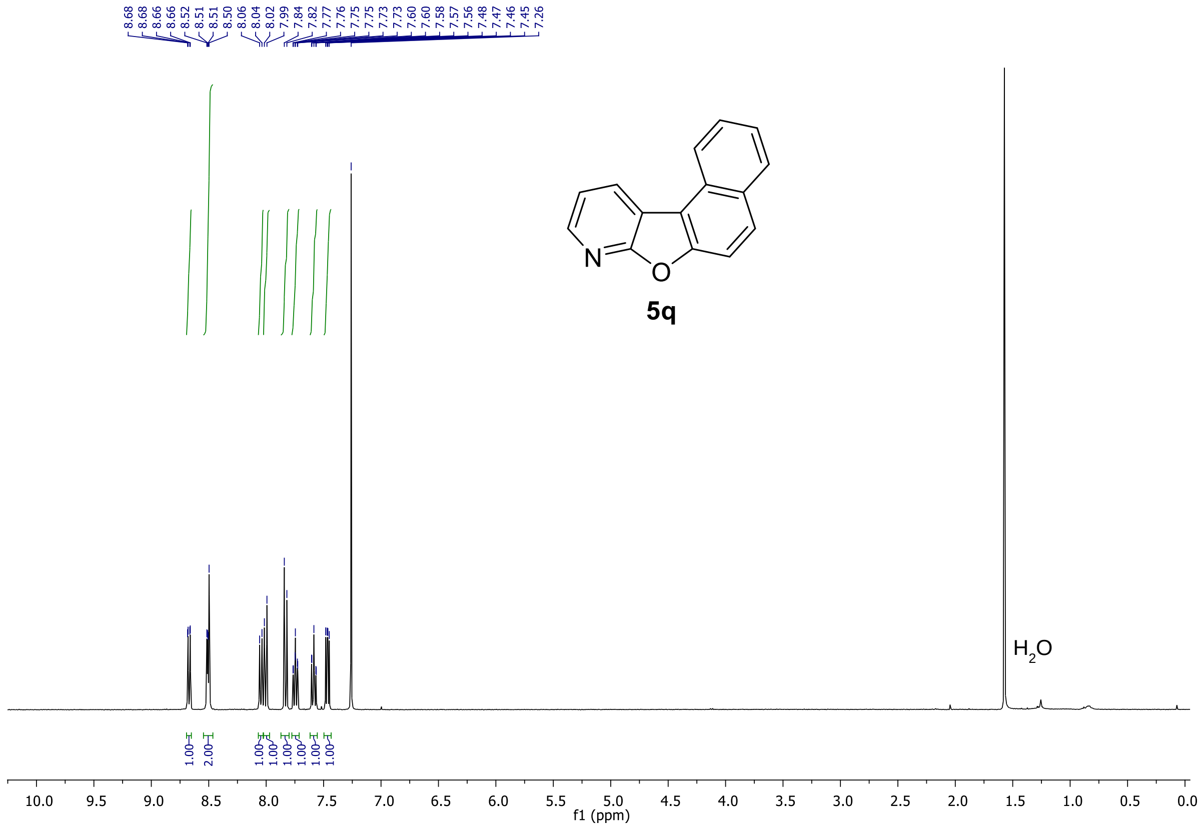

$^{13}C\{^{1}H\}$ NMR (126 MHz, $CDCl_3$)

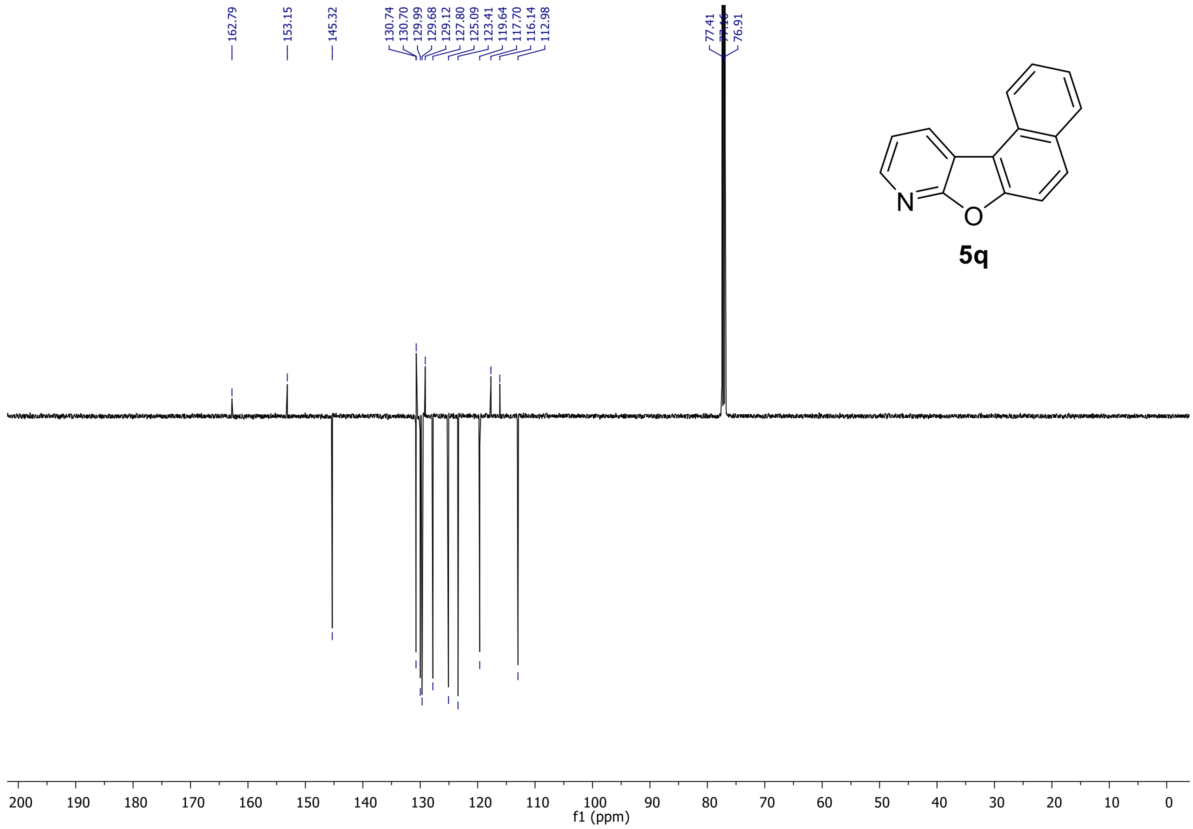

^{1}H NMR (500 MHz, DMSO-*d6*)

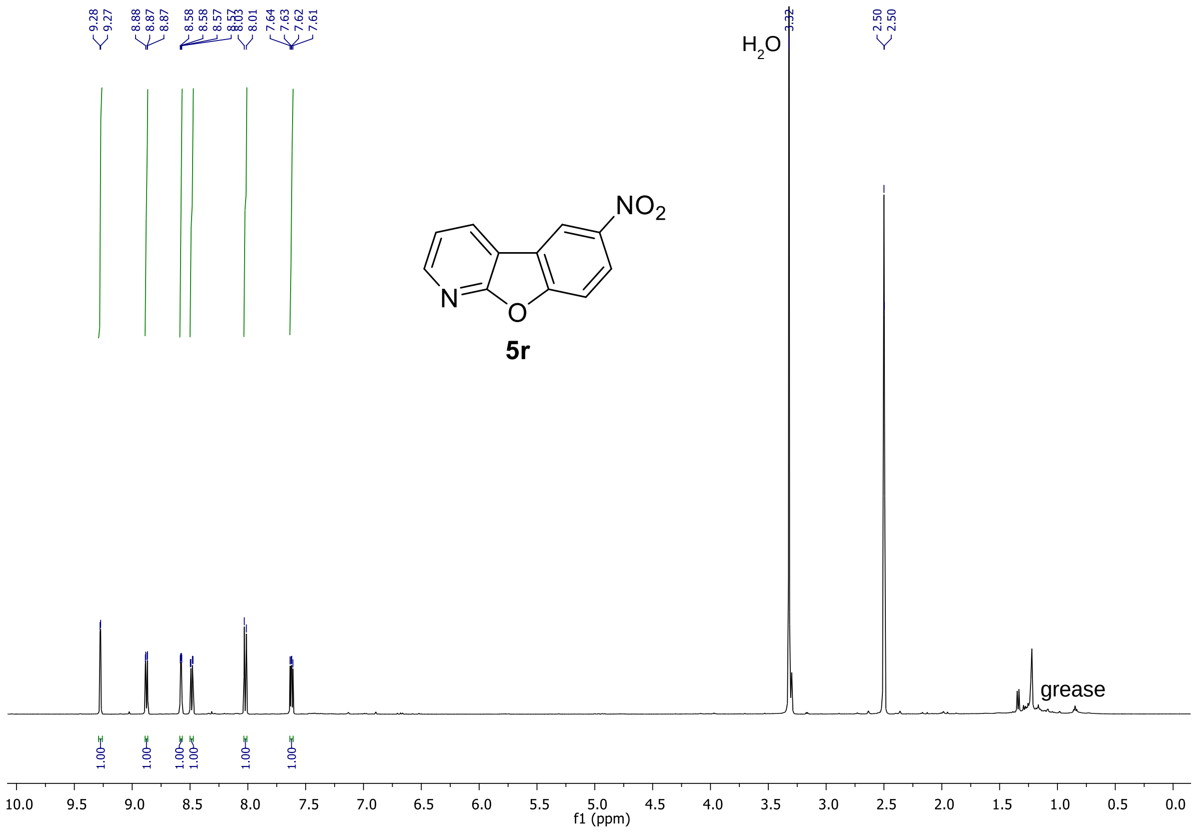

$^{13}C\{^{1}H\}$ NMR (126 MHz, DMSO-*d6*)

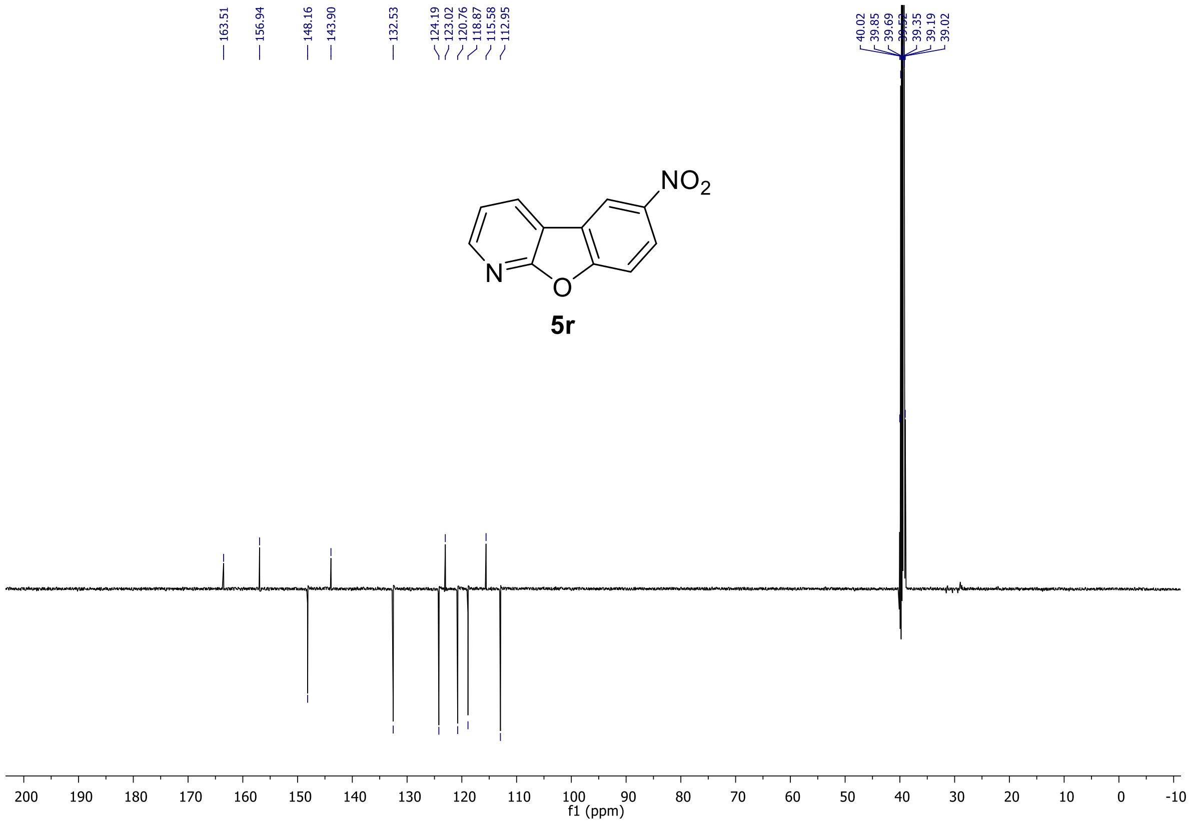

^{1}H NMR (500 MHz, DMSO-*d6*)

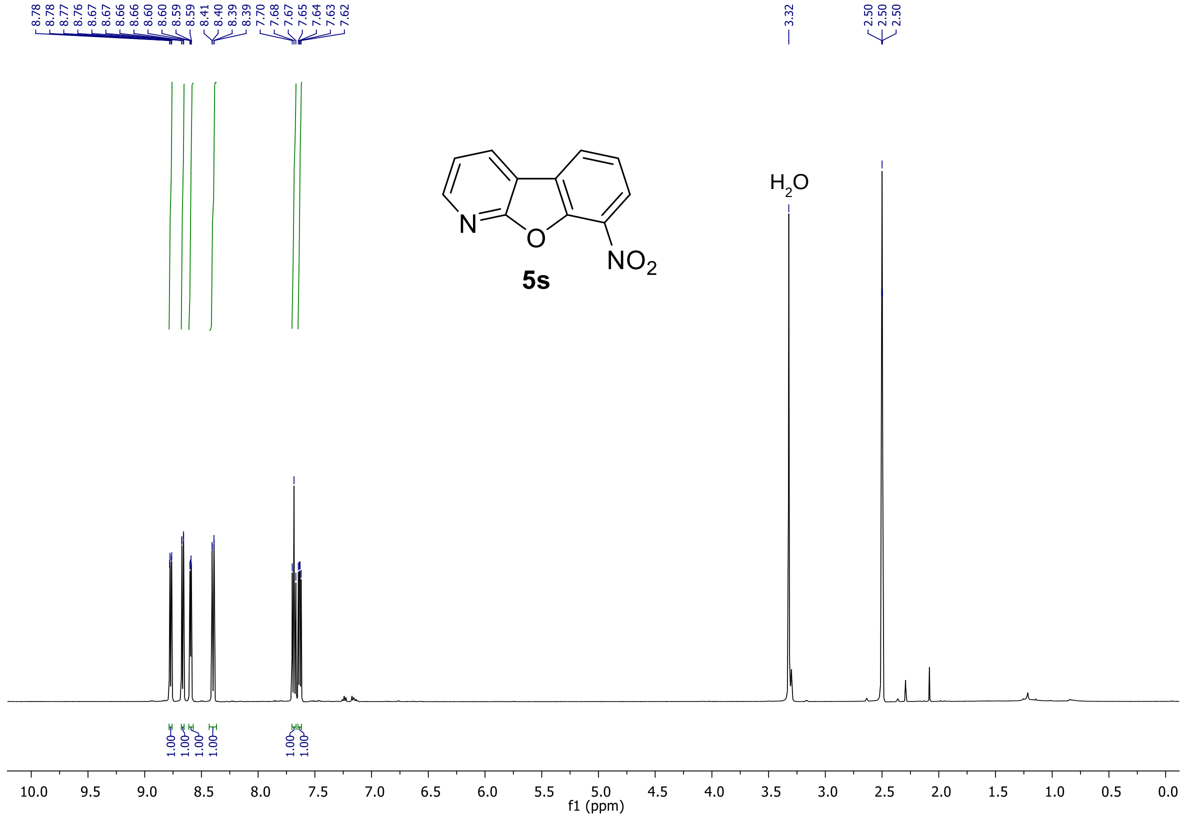

$^{13}C\{^1H\}$ NMR (126 MHz, DMSO-*d6*)

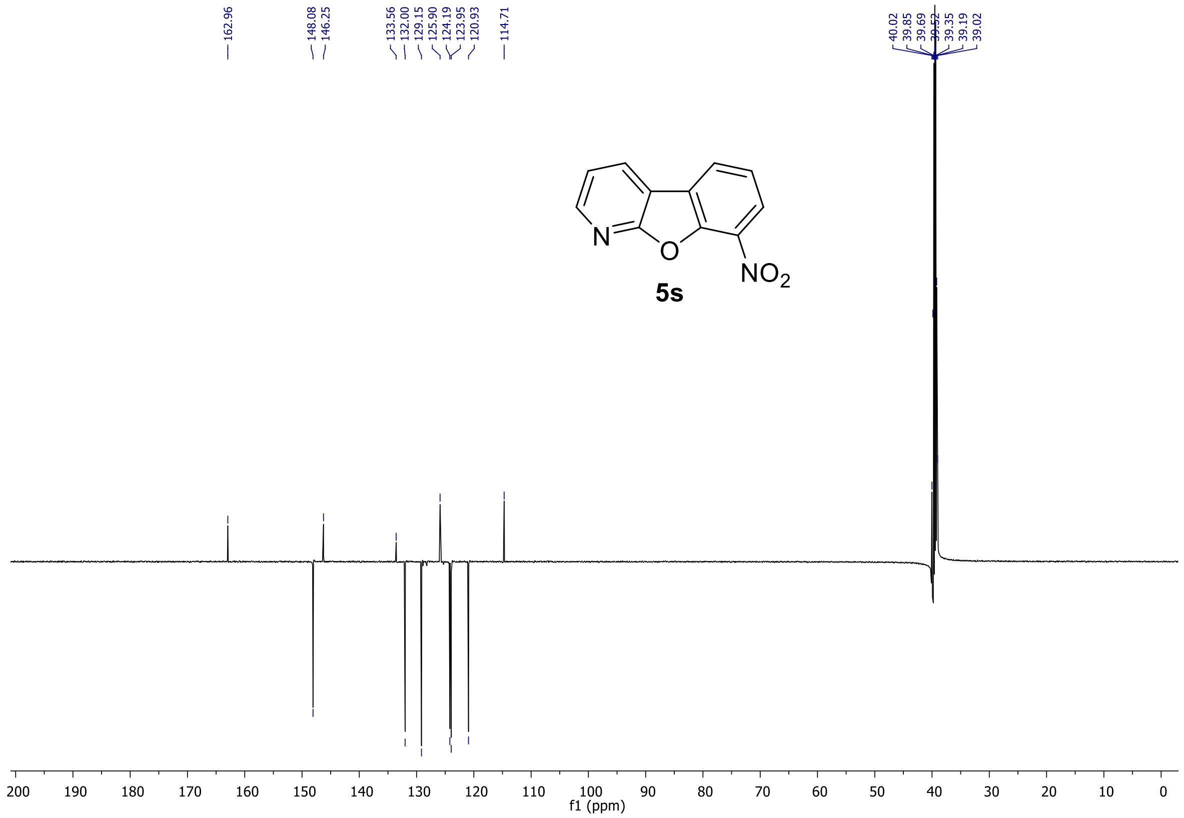

^1H NMR (500 MHz, $CDCl_3$)

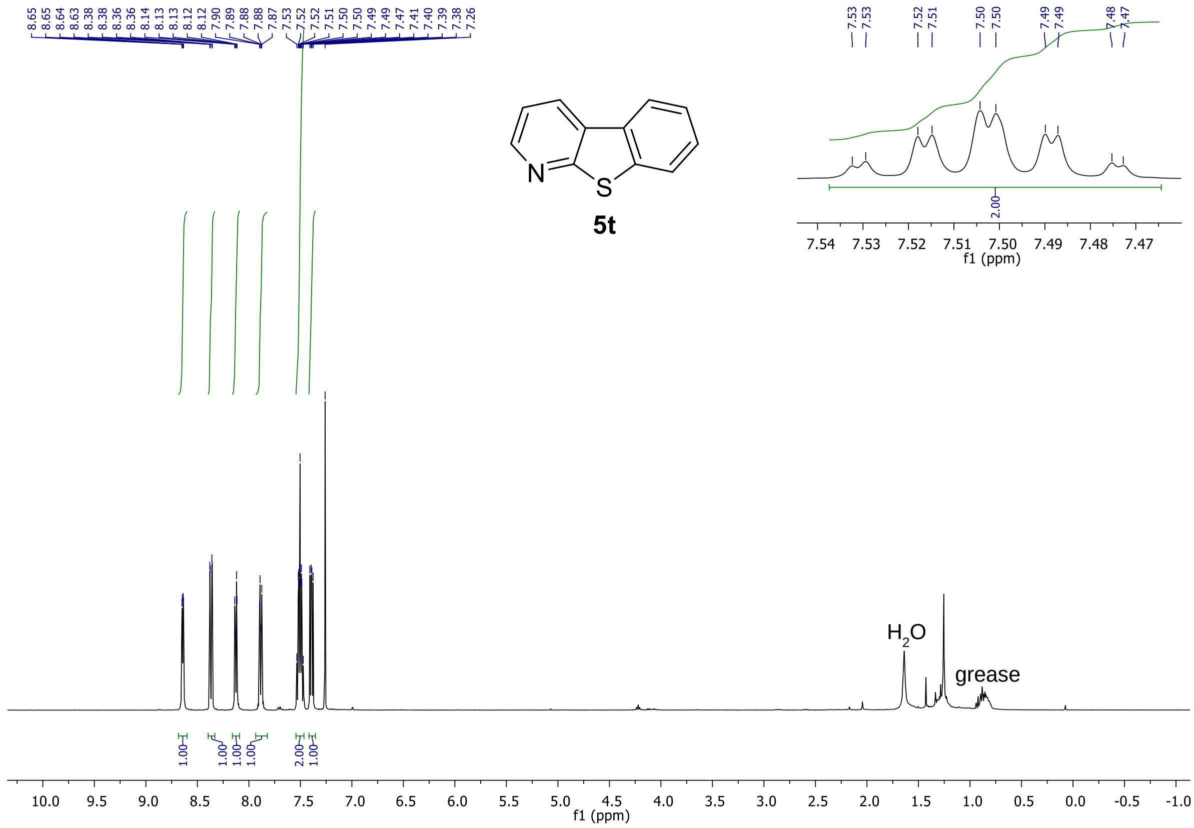

$^{13}C\{^{1}H\}$ NMR (126 MHz, $CDCl_3$)

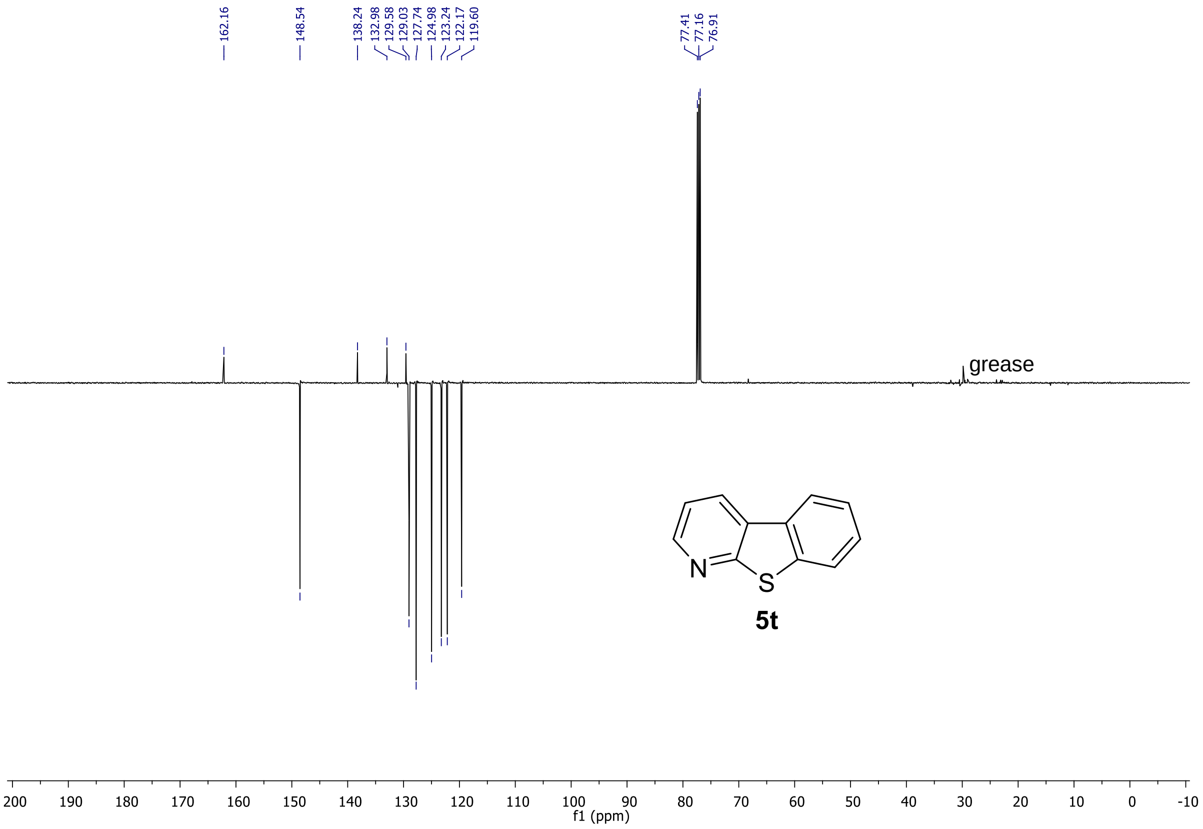

^{1}H NMR (400 MHz, $CDCl_3$)

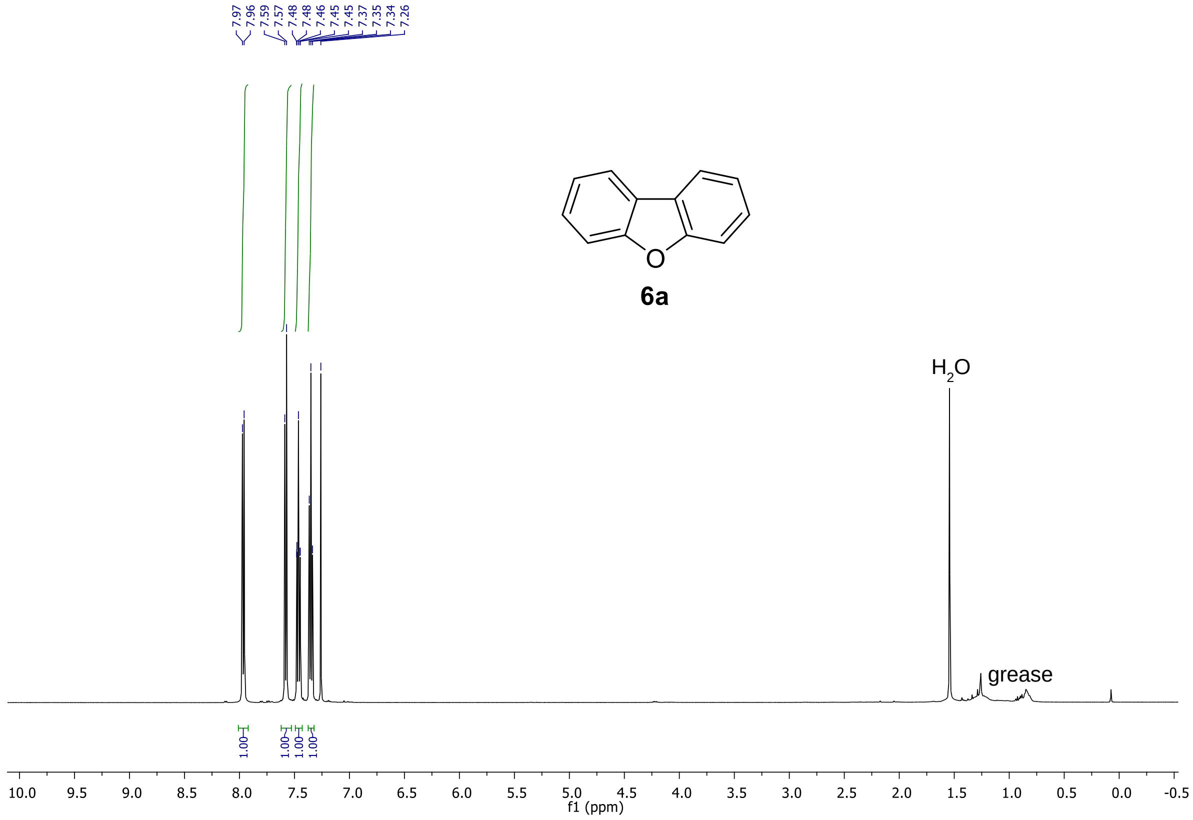

$^{13}C\{^{1}H\}$ NMR (126 MHz, $CDCl_3$)

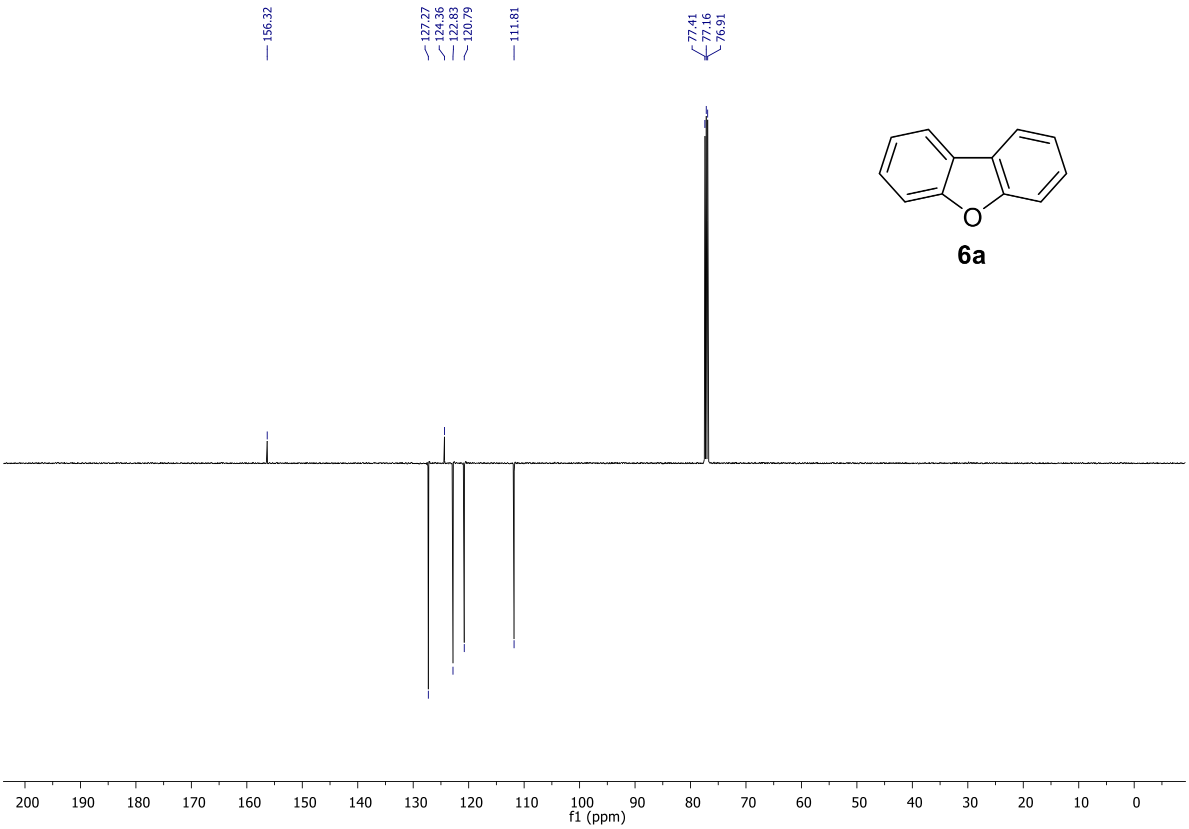

^{1}H NMR (500 MHz, $CDCl_3$)

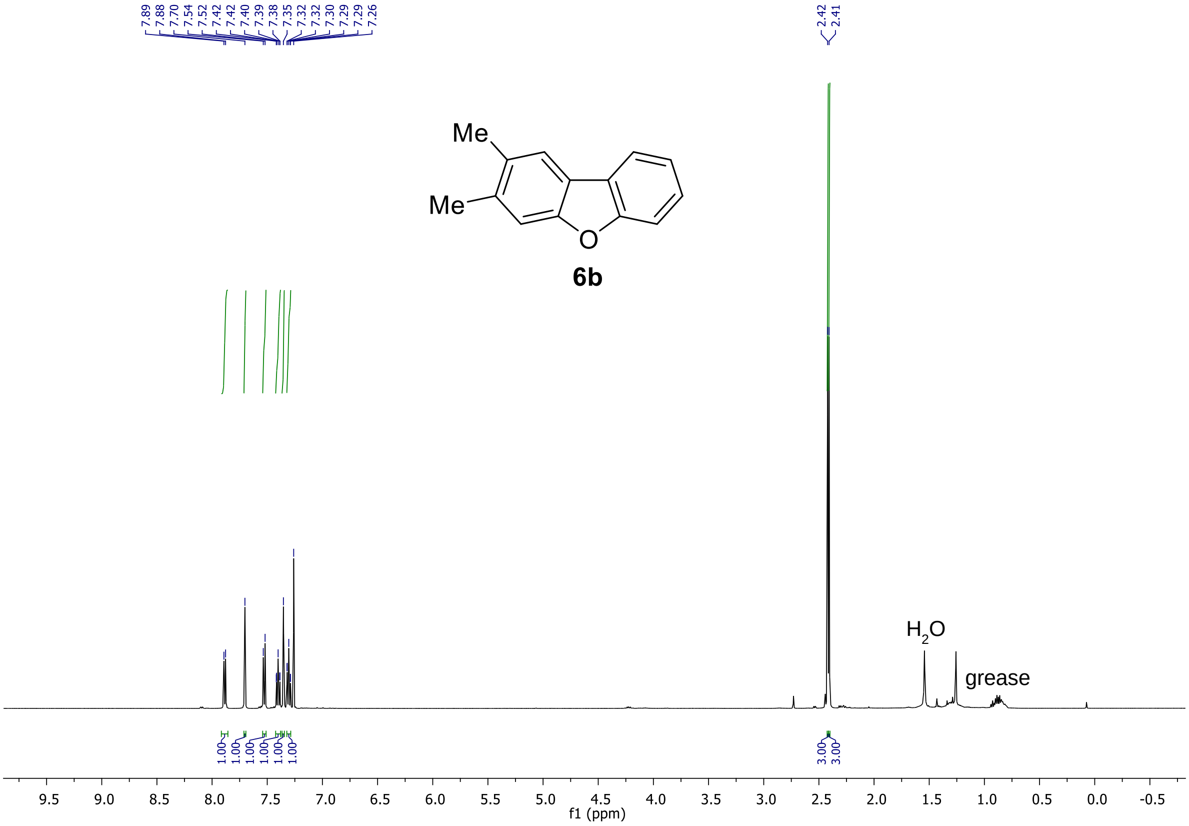

$^{13}C\{^{1}H\}$ NMR (126 MHz, $CDCl_3$)

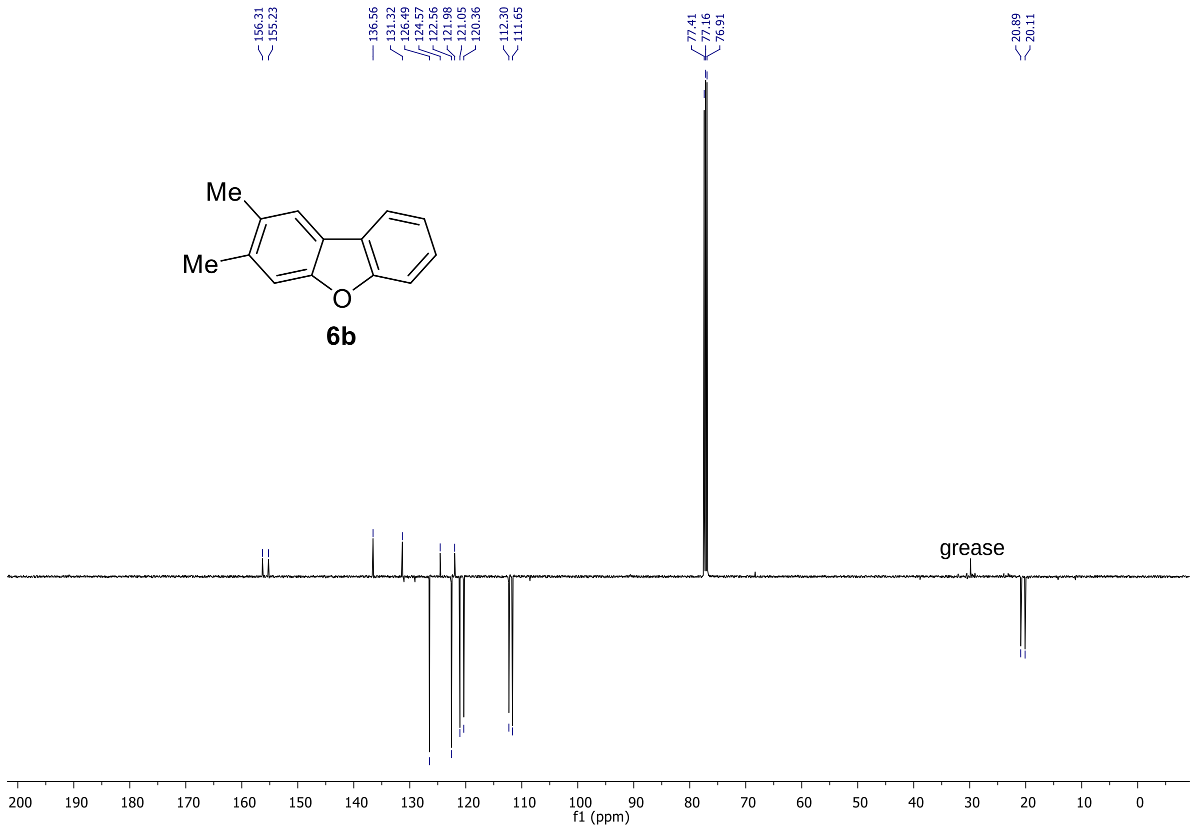

^{1}H NMR (500 MHz, $CDCl_3$)

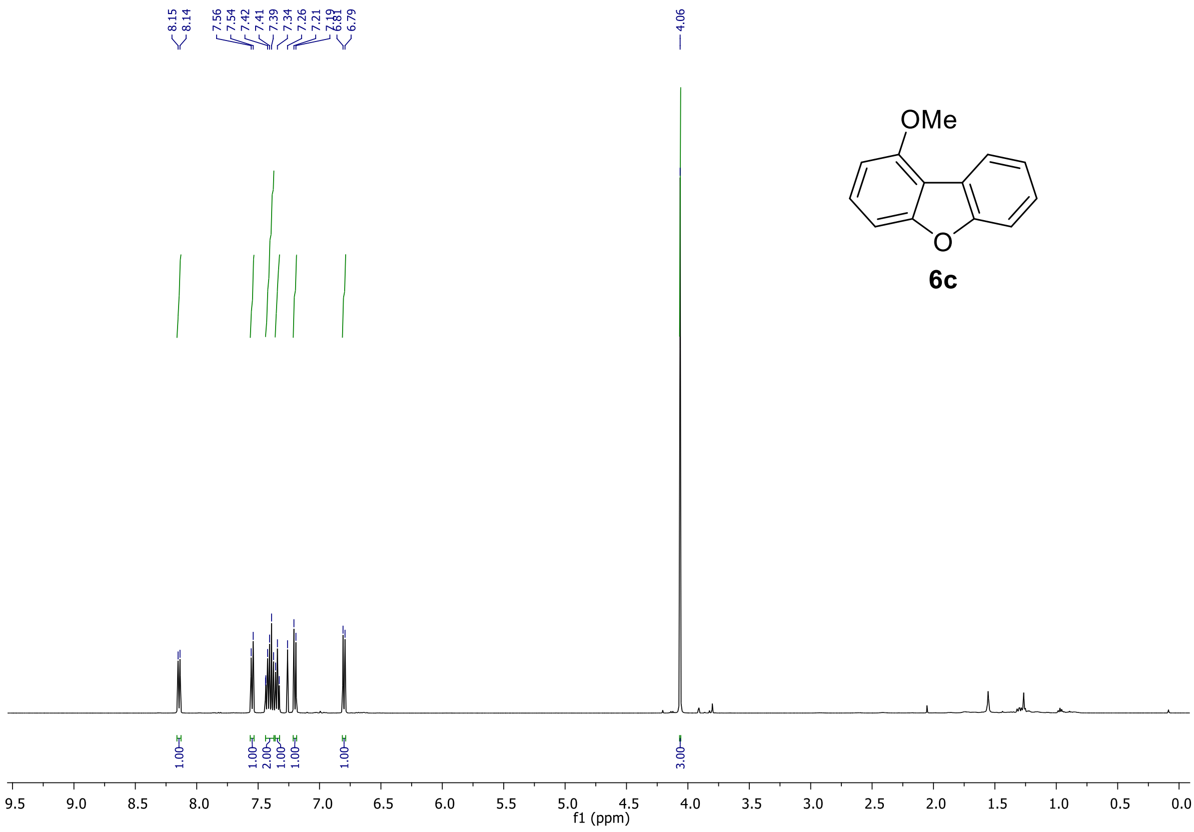

$^{13}C\{^{1}H\}$ NMR (126 MHz, $CDCl_3$)

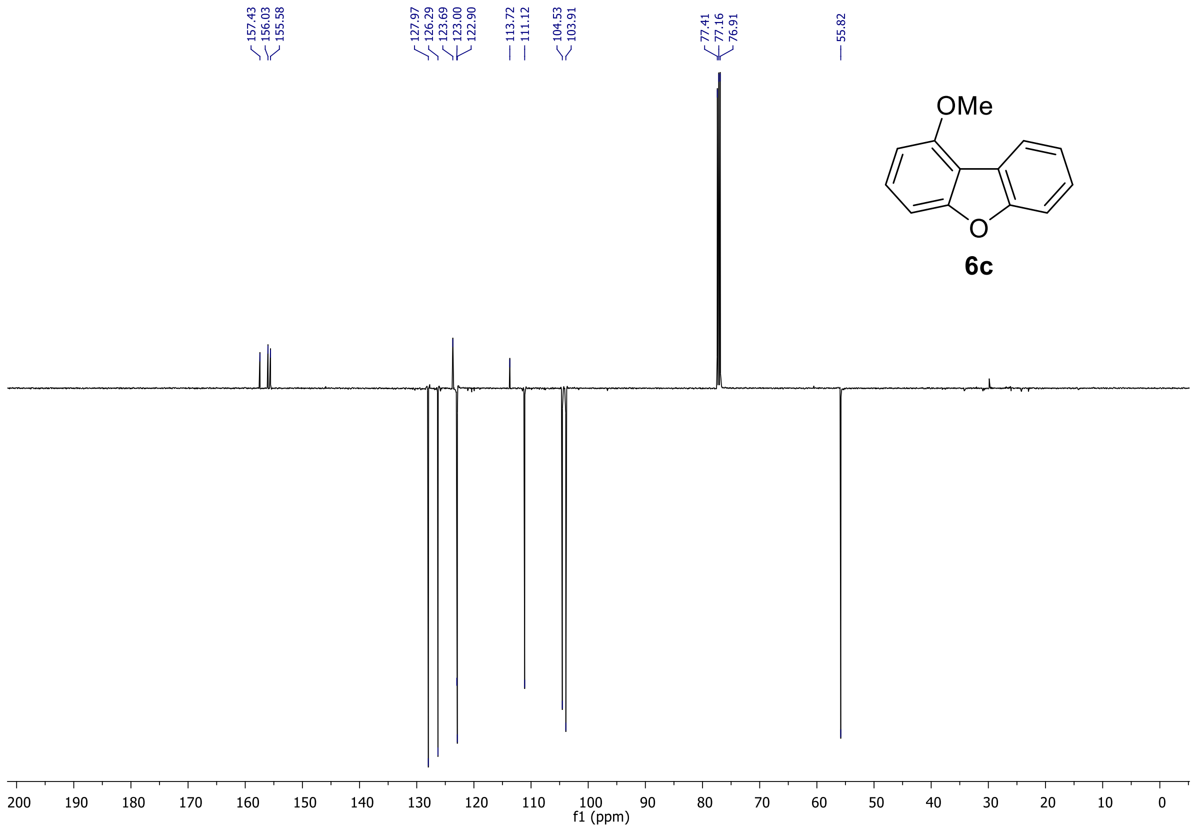

^{1}H NMR (500 MHz, $CDCl_3$)

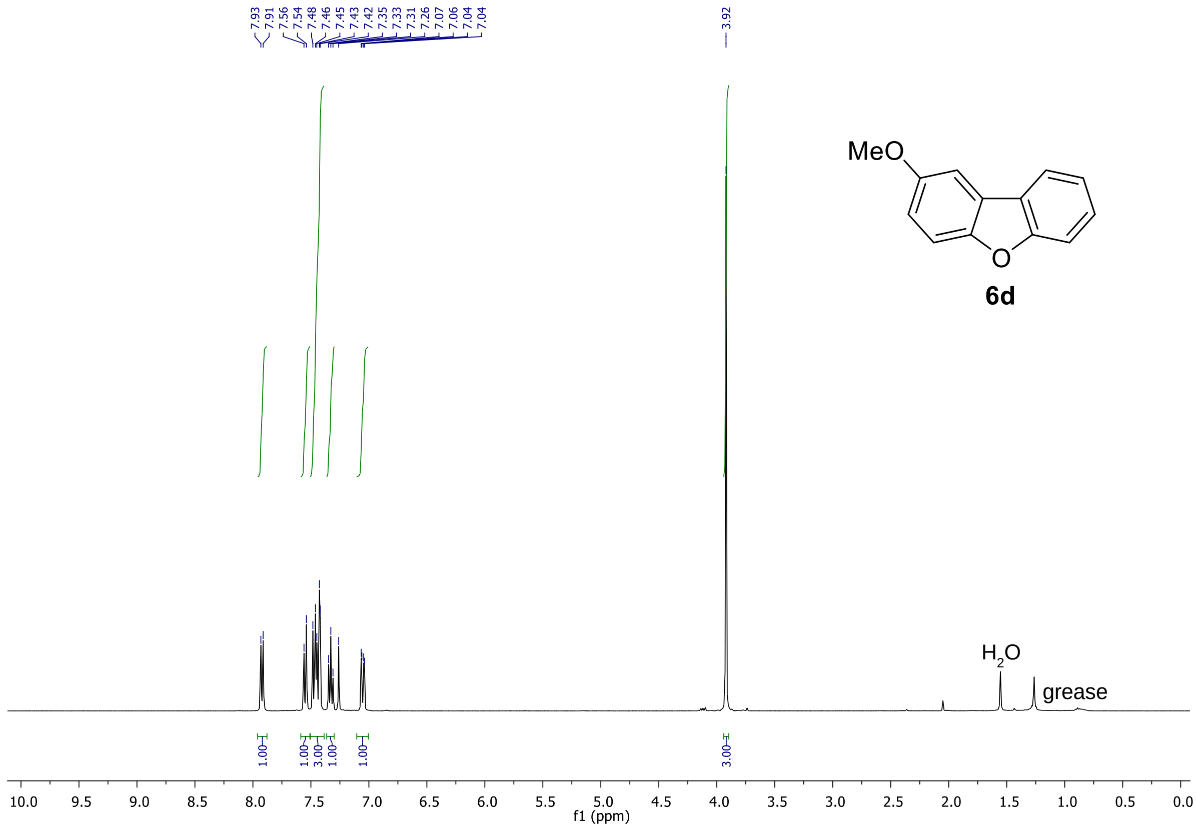

$^{13}C\{^1H\}$ NMR (126 MHz, $CDCl_3$)

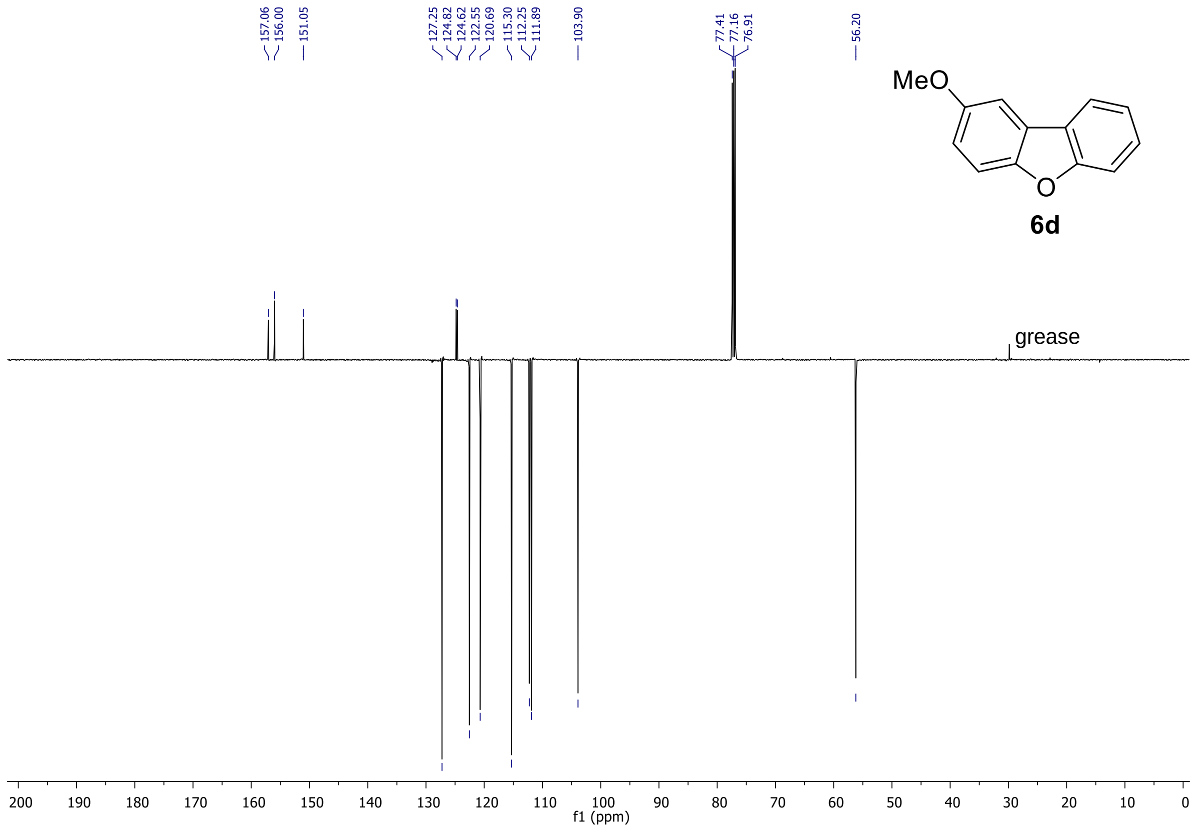

^1H NMR (500 MHz, $CDCl_3$)

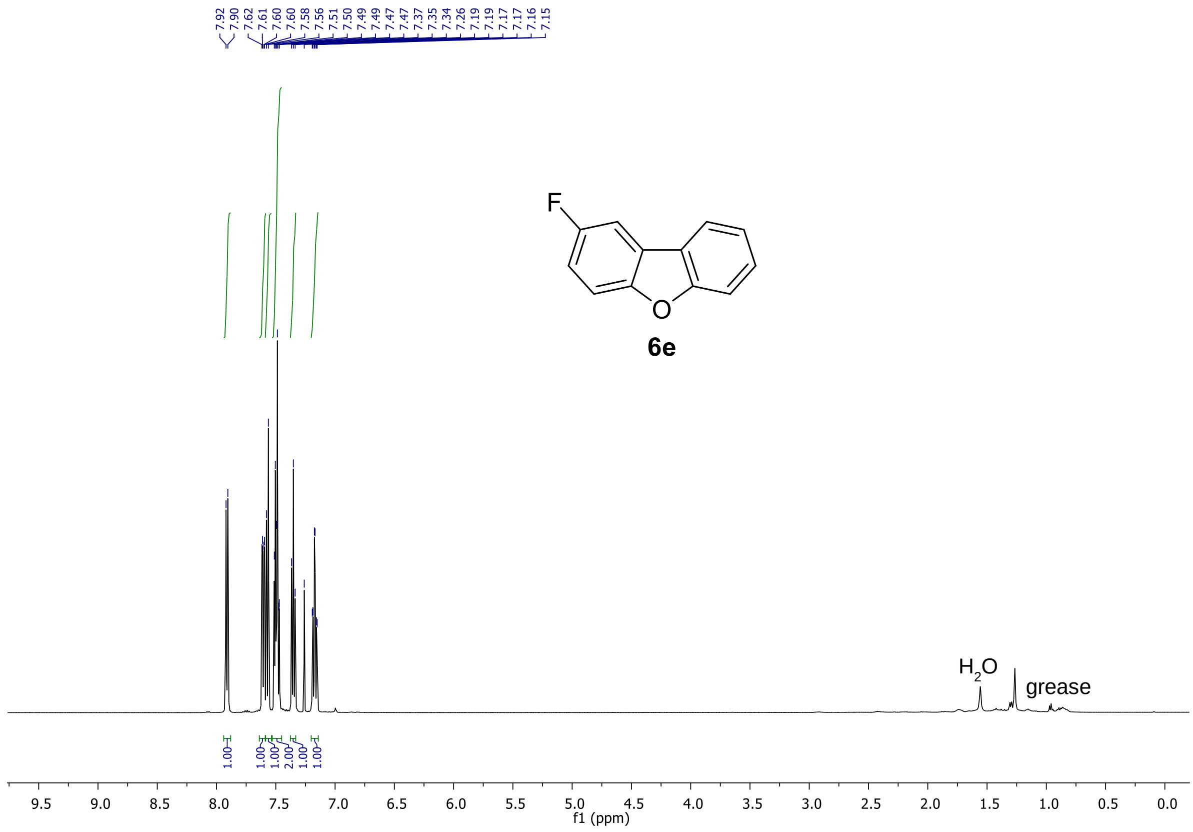

$^{13}C\{^{1}H\}$ NMR (126 MHz, $CDCl_3$)

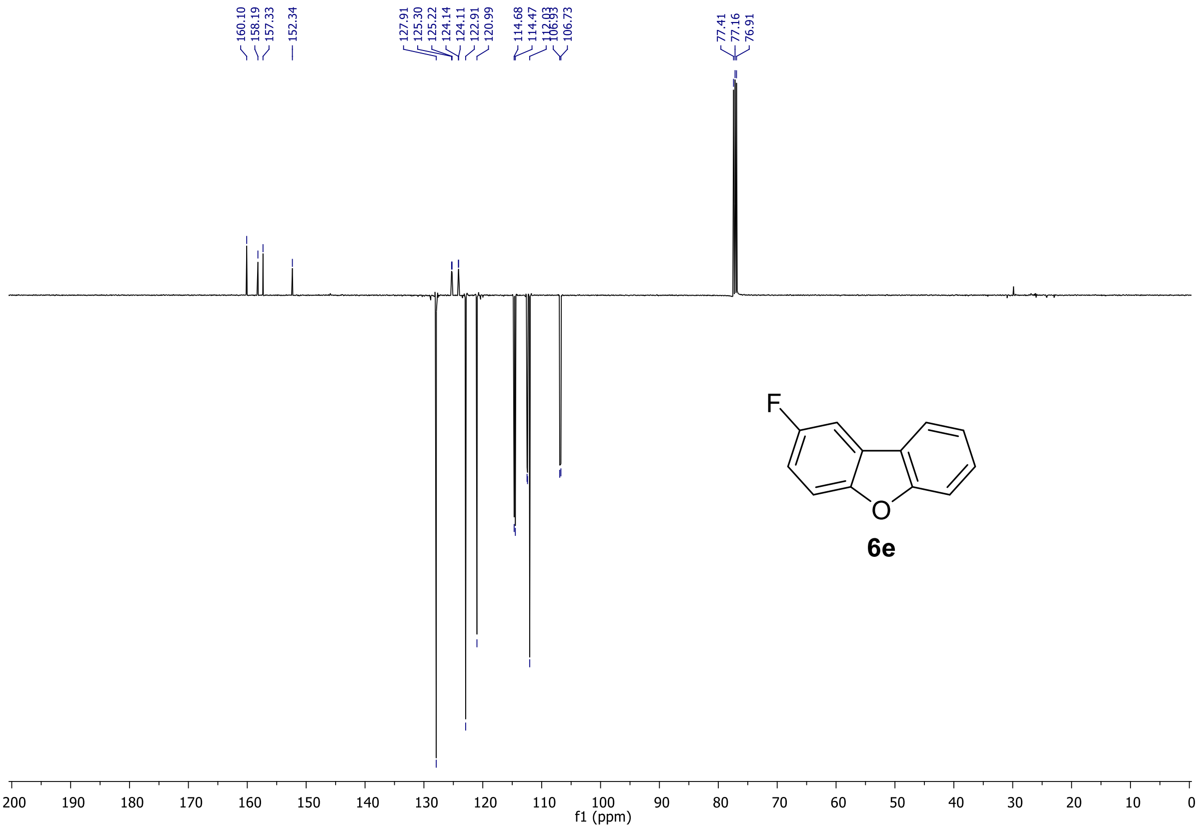

$^{19}F\{^{1}H\}$ NMR (376 MHz, $CDCl_3$)

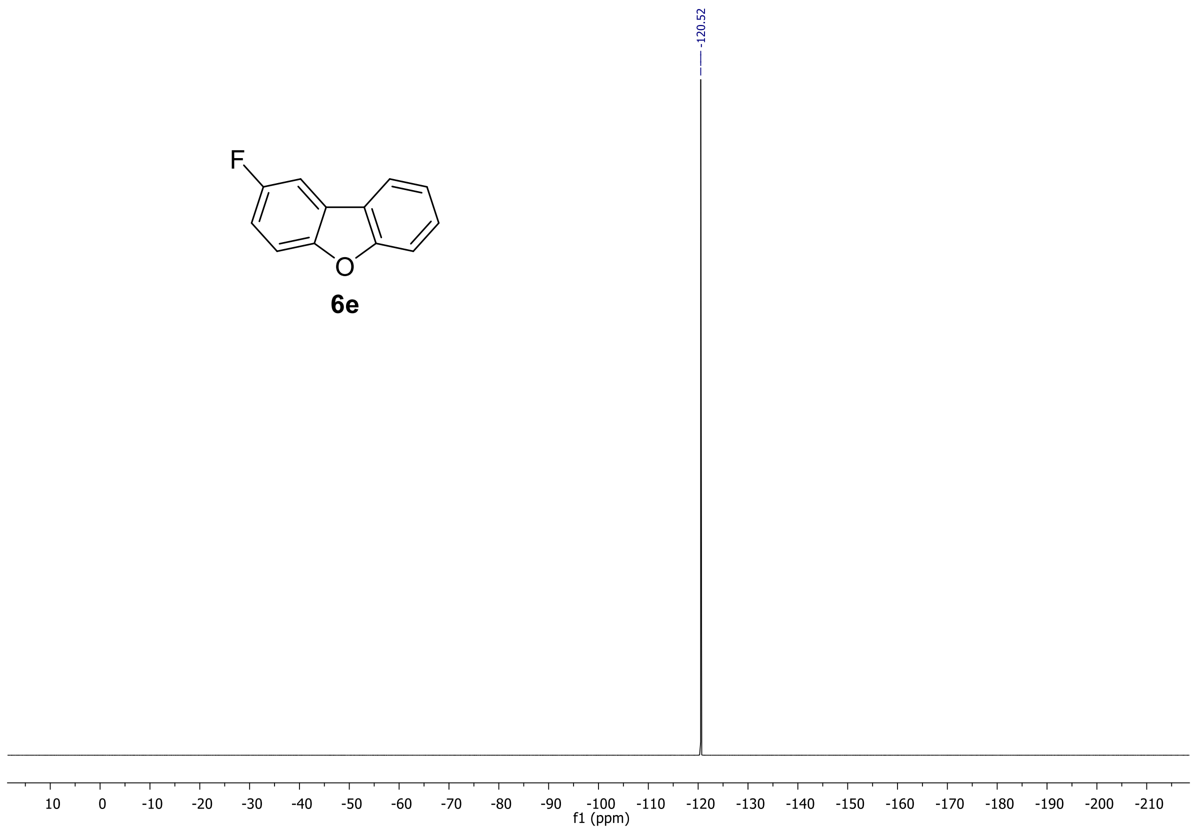

^{1}H NMR (500 MHz, $CDCl_3$)

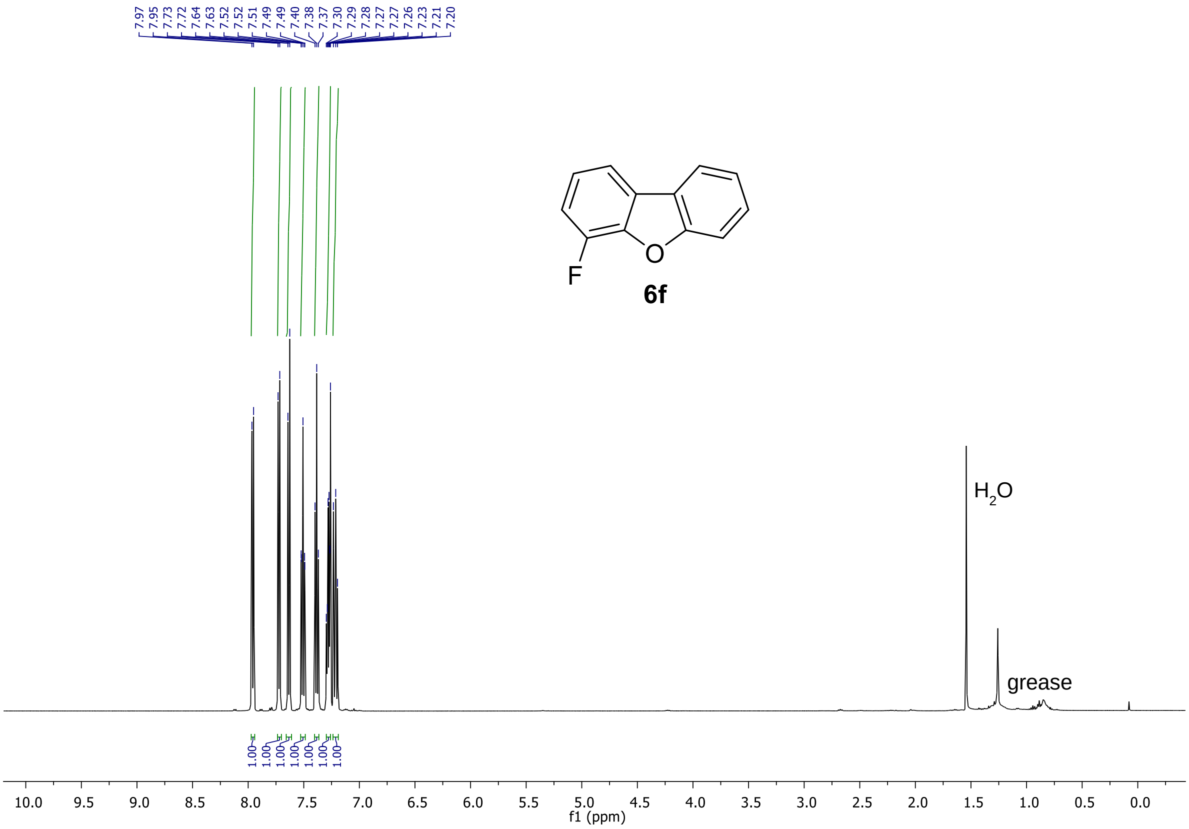

$^{13}C\{^{1}H\}$ NMR (126 MHz, $CDCl_3$)

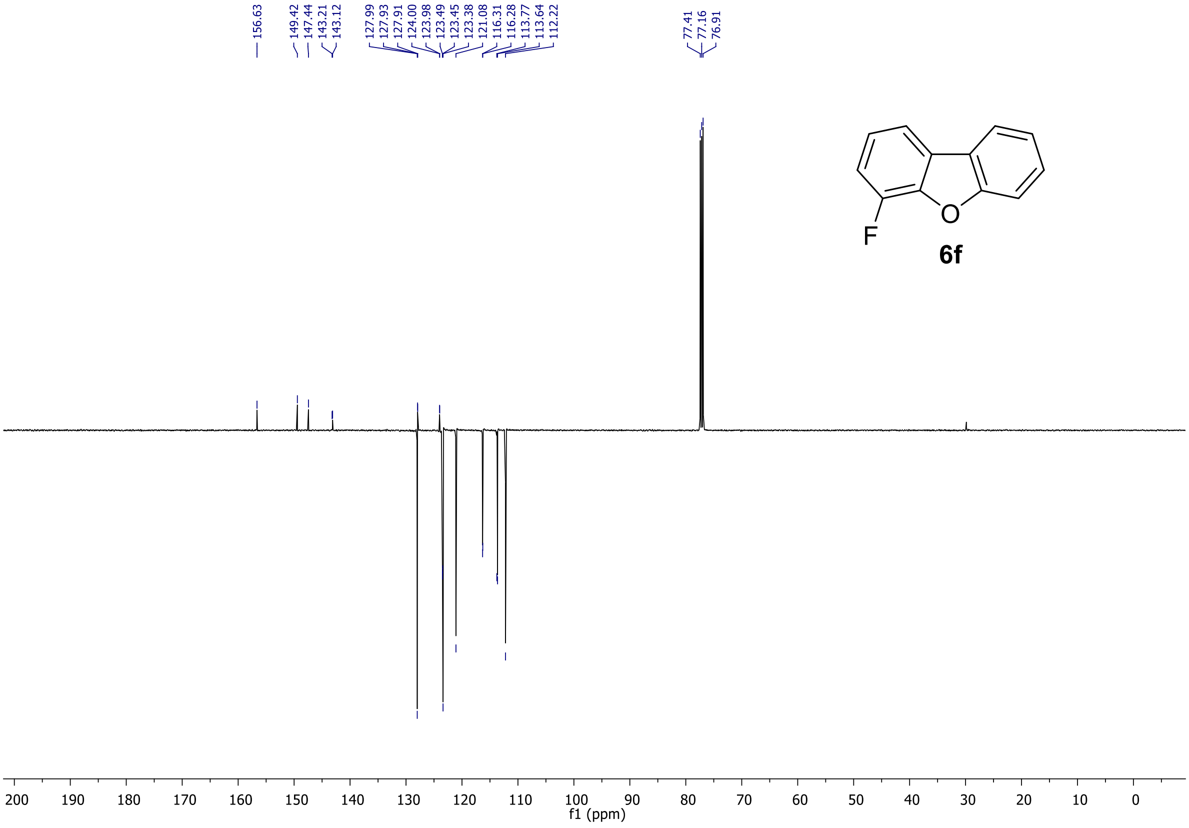

$^{19}F\{^1H\}$ NMR (376 MHz, $CDCl_3$)

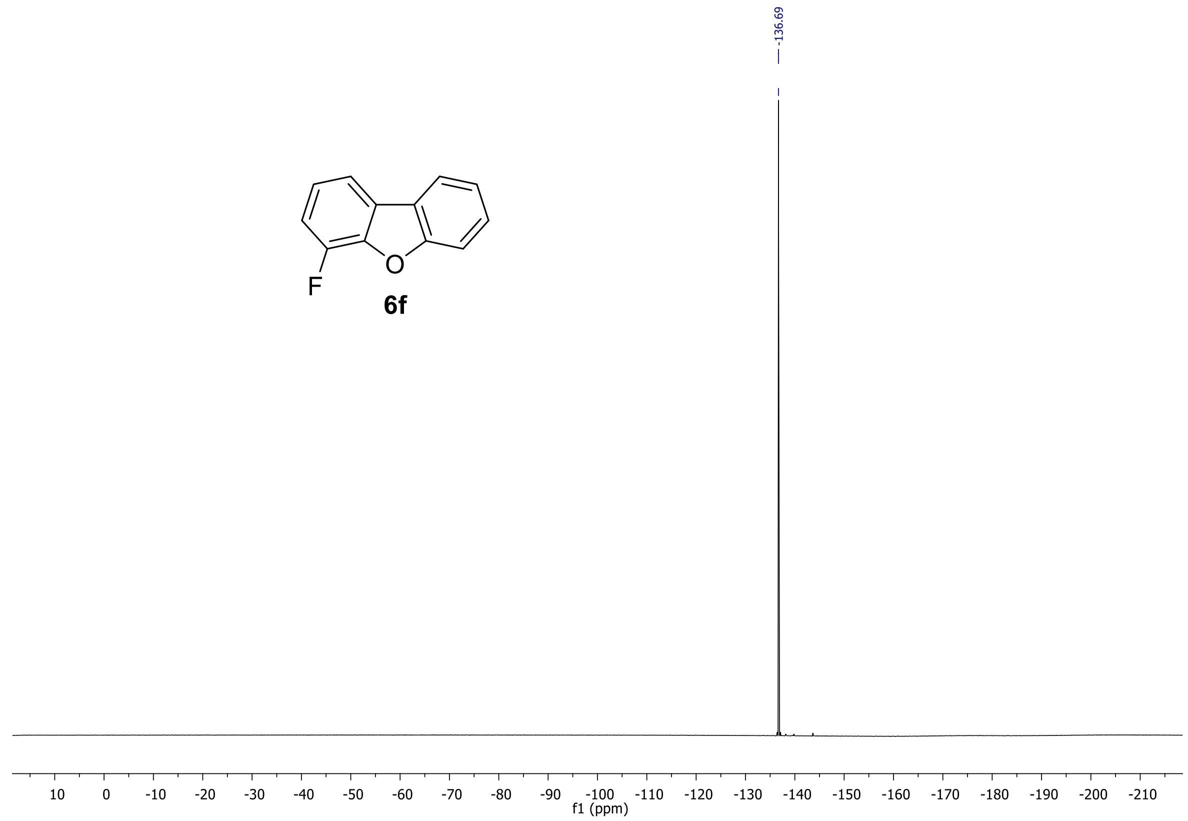

^1H NMR (500 MHz, $CDCl_3$)

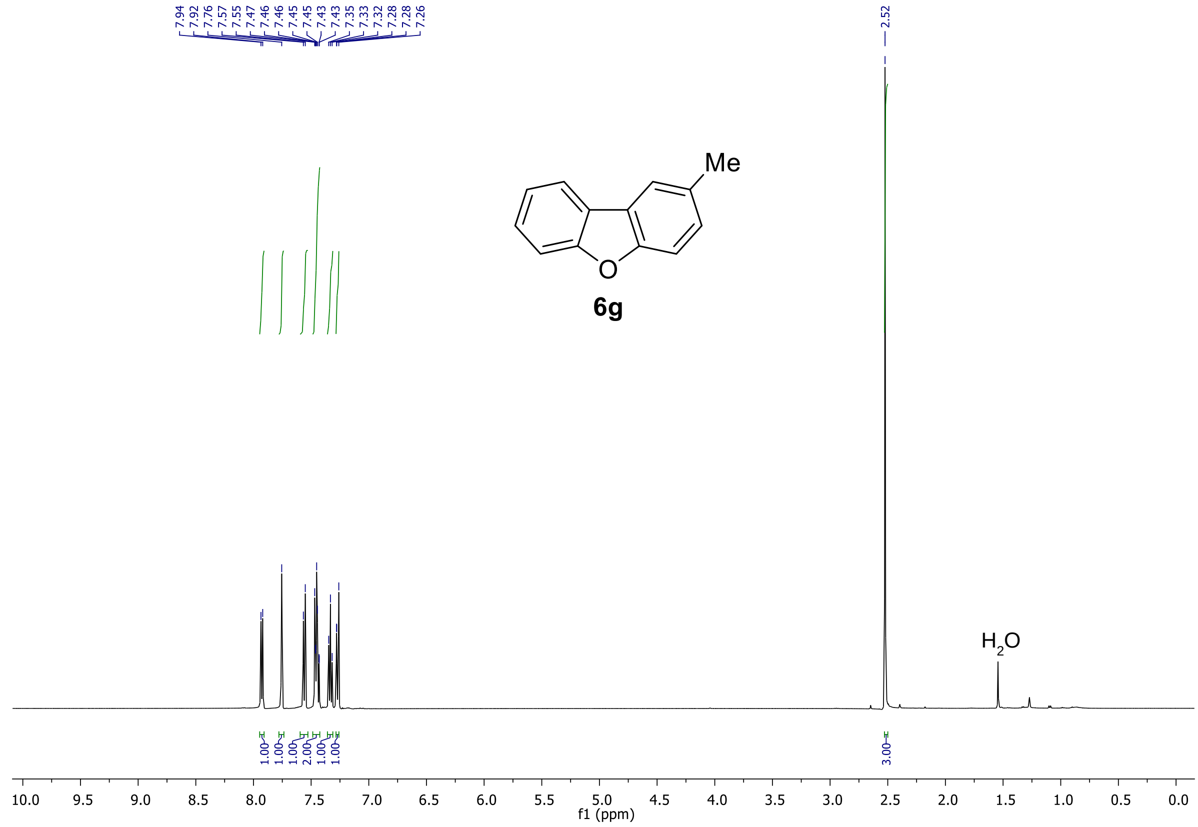

$^{13}C\{^{1}H\}$ NMR (126 MHz, $CDCl_3$)

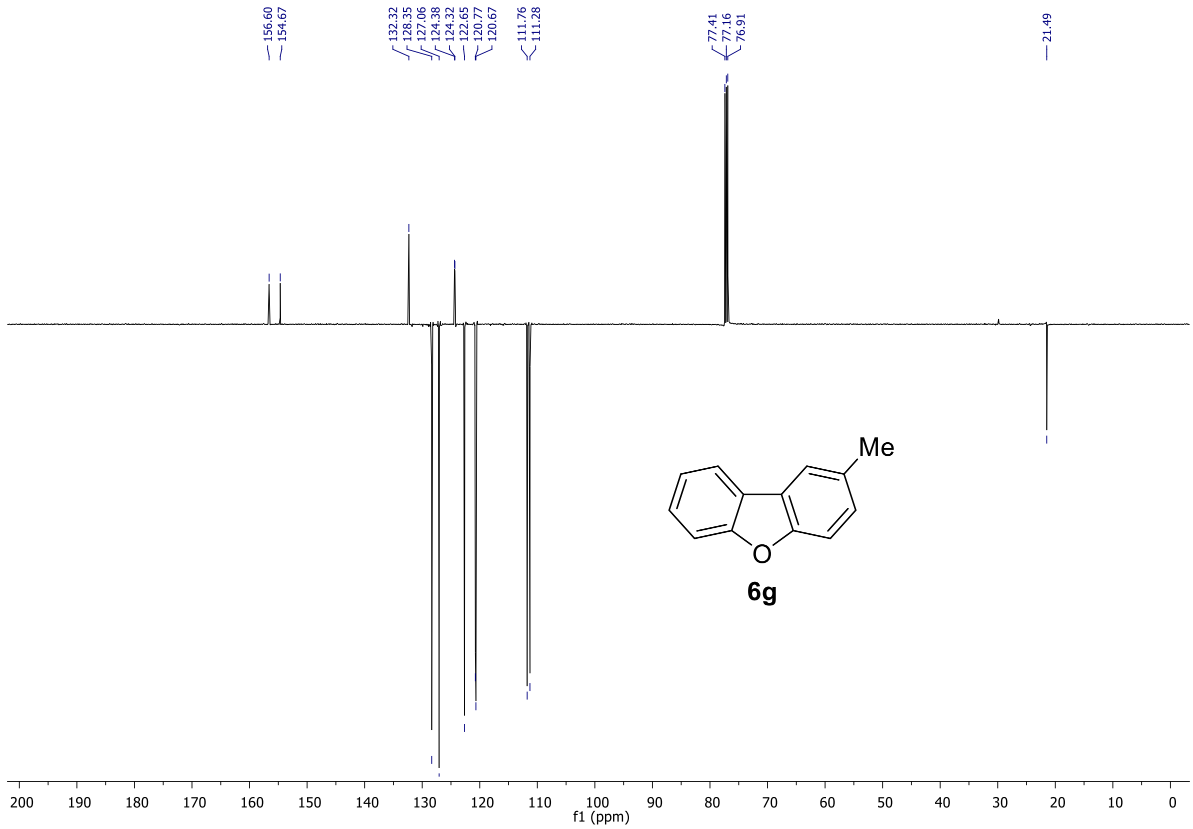

^{1}H NMR (500 MHz, $CDCl_3$)

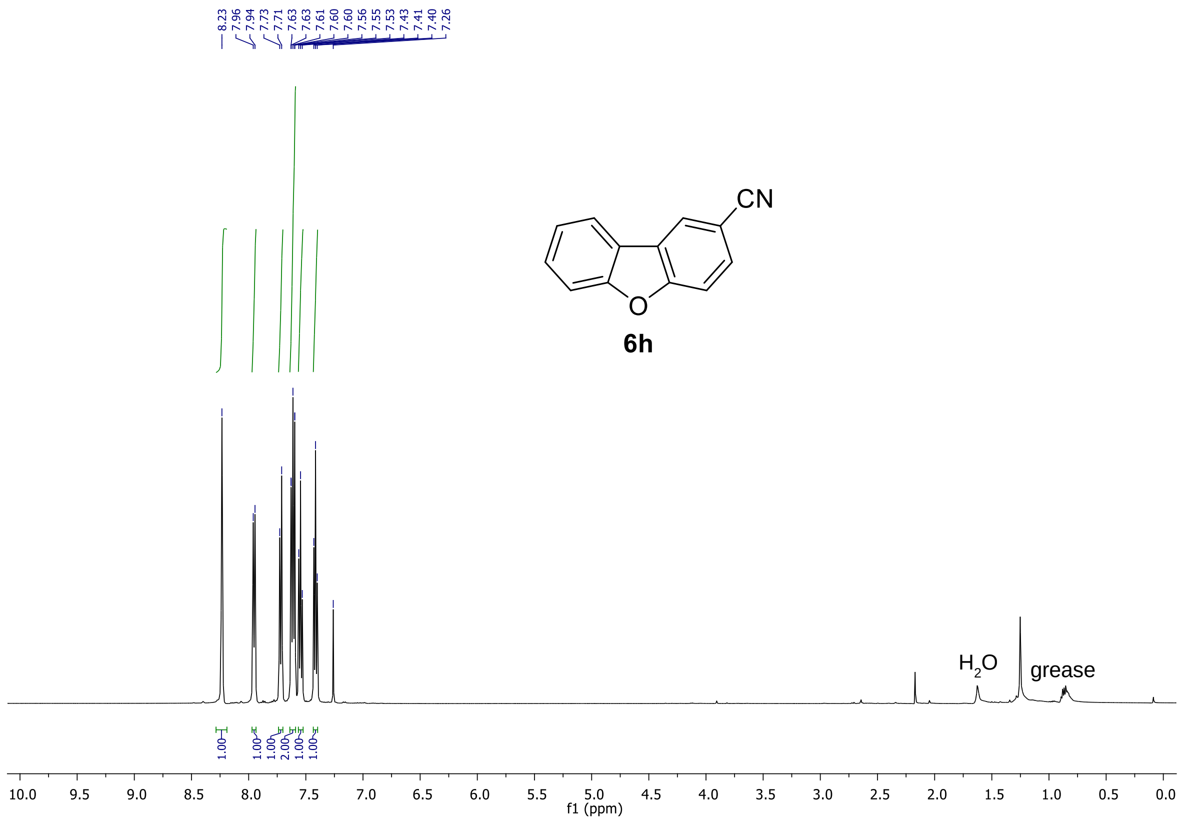

$^{13}C\{^{1}H\}$ NMR (126 MHz, $CDCl_3$)

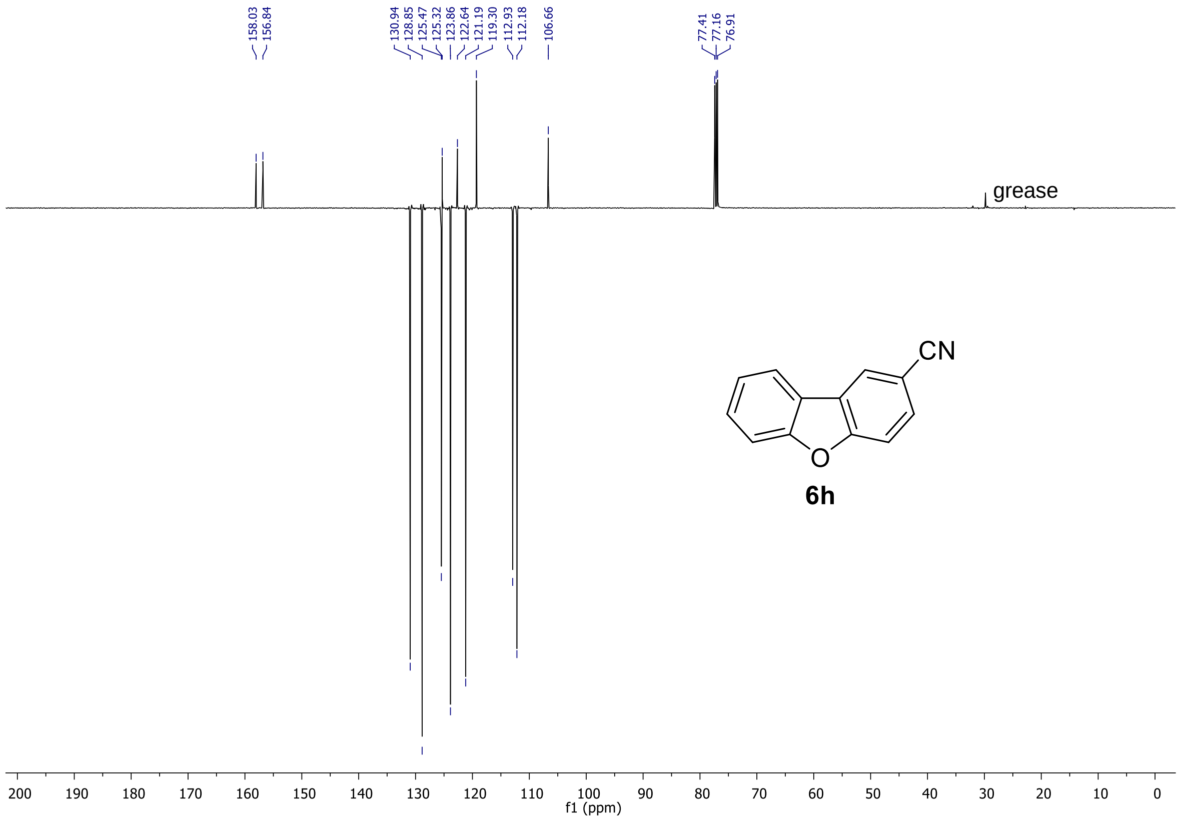

^{1}H NMR (500 MHz, $CDCl_3$)

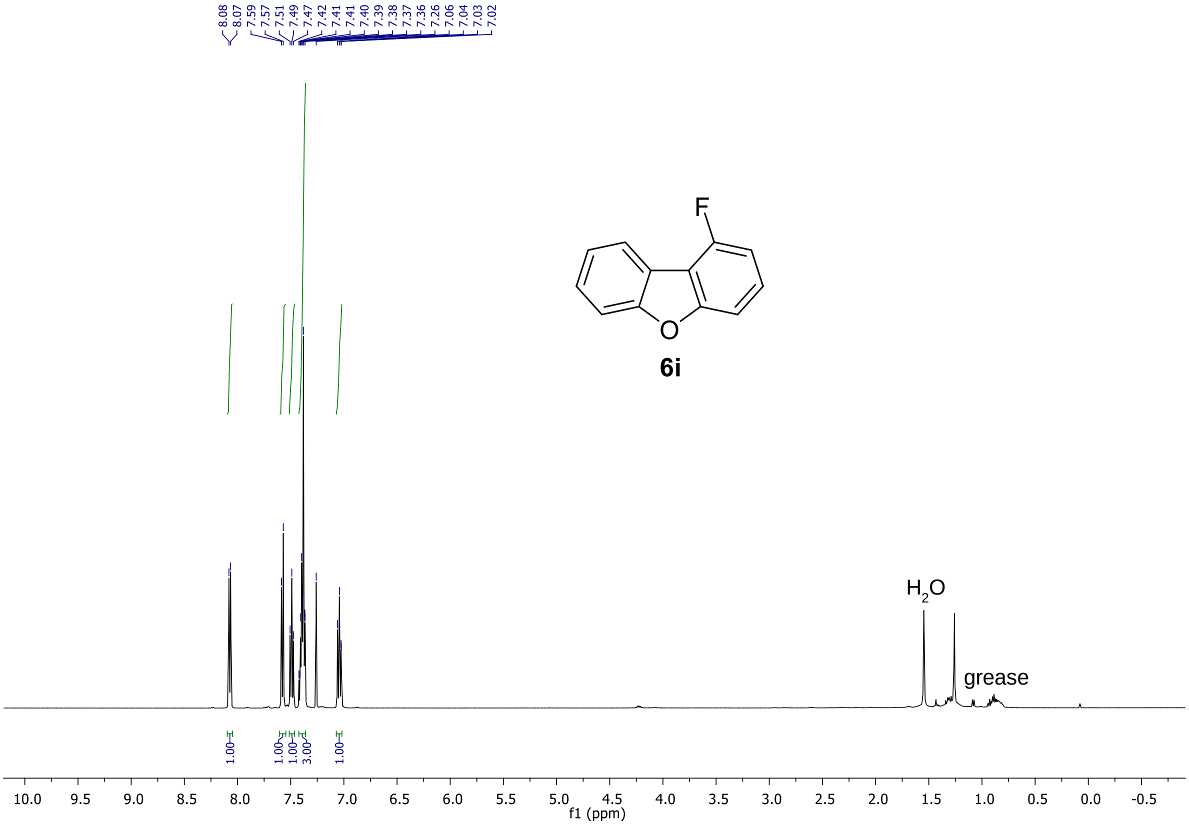

$^{13}C\{^{1}H\}$ NMR (126 MHz, $CDCl_3$)

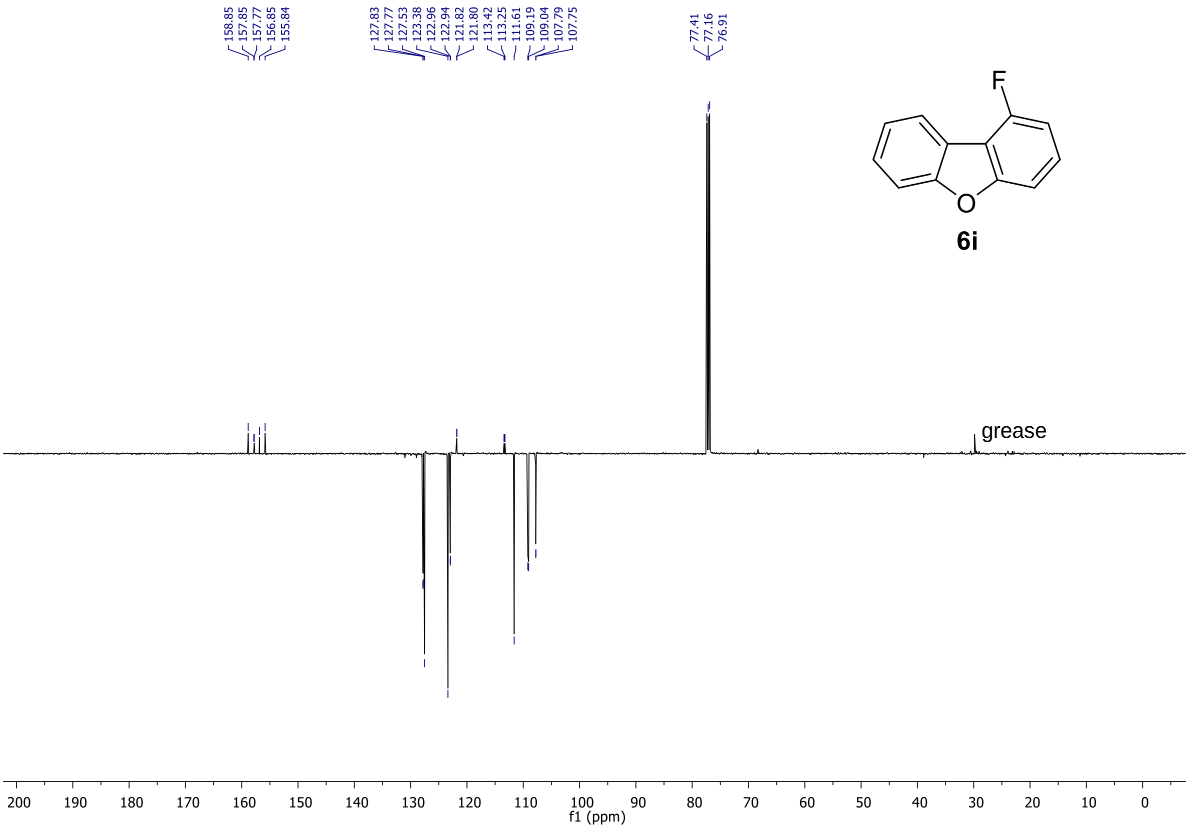

$^{19}F\{^{1}H\}$ NMR (376 MHz, $CDCl_3$)

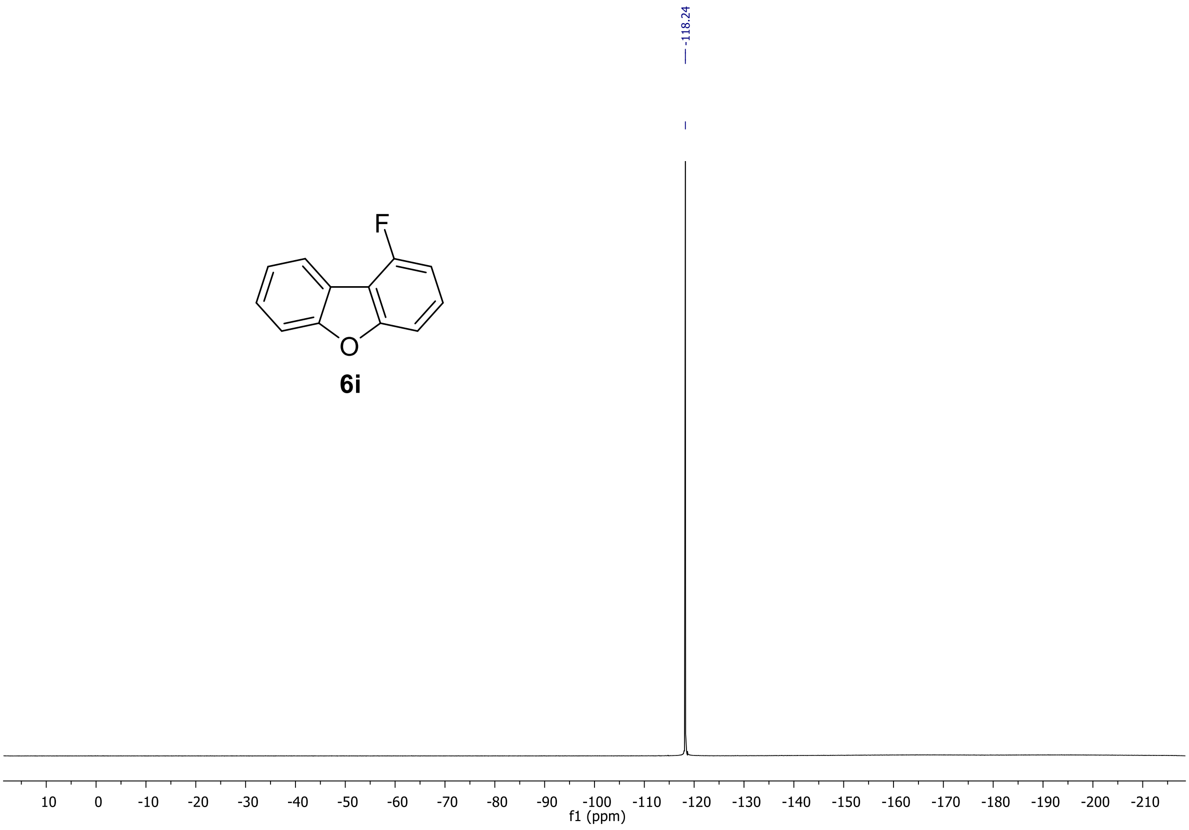

5. Single crystal X-ray structures

Single crystal X-ray structures of 5q. Single crystals of $C_{15}H_9NO$ were grown from $CHCl_3$. A suitable crystal was selected and mounted on a glass fibre with Fomblin oil and placed on a Rigaku Oxford Diffraction SuperNova diffractometer with a dual source (Cu at zero) equipped with an AtlasS2 CCD area detector. The crystal was kept at 150(2) K during data collection. Using Olex2, the structure was solved with the SHELXT structure solution program using Intrinsic Phasing and refined with the SHELXL refinement package using Least Squares minimisation.

Crystal Data for $C_{15}H_9NO$ (M =219.23 g/mol): orthorhombic, space group Pbca (no. 61), a = 13.68110(10) Å, b = 8.77410(10) Å, c = 17.31440(10) Å, V = 2078.41(3) Å^3, Z = 8, T = 150(2) K, μ(Cu Kα) = 0.705 mm^{-1}, $Dcalc$ = 1.401 g/cm^3, 39686 reflections measured (10.218° ≤ 2Θ ≤ 147.19°), 2096 unique (R_{int} = 0.0923, R_{sigma} = 0.0224) which were used in all calculations. The final R_1 was 0.0410 (I > 2σ(I)) and wR_2 was 0.1138 (all data).

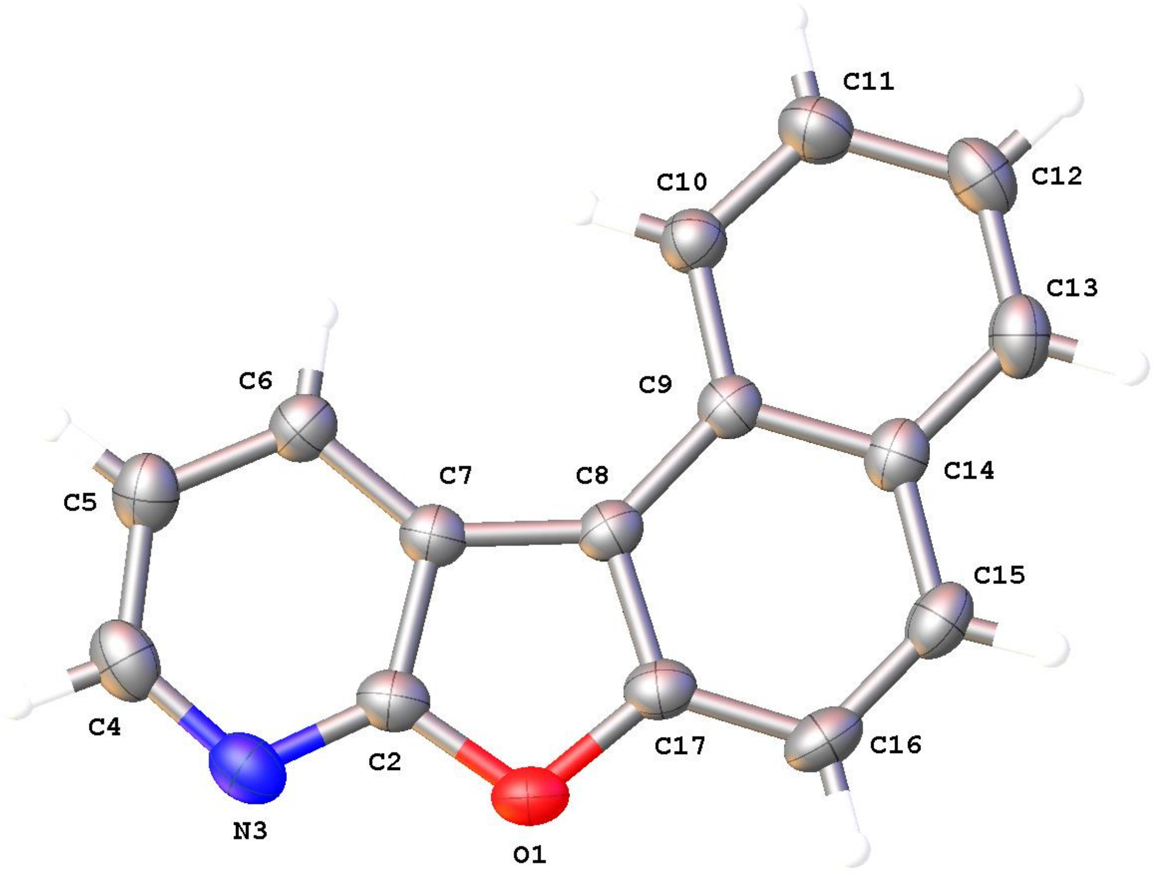

Single crystal X-ray structures of 6c. Single crystals of $C_{13}H_{10}O_2$ were grown from $CHCl_3$. A suitable crystal was selected and mounted on a glass fibre with Fomblin oil and placed on a Rigaku Oxford Diffraction SuperNova diffractometer with a dual source (Cu at zero) equipped with an AtlasS2 CCD area detector. The crystal was kept at 200(2) K during data collection. Using Olex2, the structure was solved with the SHELXT structure solution program using Intrinsic Phasing and refined with the SHELXL refinement package using Least Squares minimisation.

Crystal Data for $C_{13}H_{10}O_2$ (M =198.21 g/mol): triclinic, space group P-1 (no. 2), a = 9.54790(10) Å, b = 10.4956(2) Å, c = 29.6248(5) Å, α = 99.9330(10)°, β = 98.9940(10)°, γ = 91.5400(10)°, V = 2883.85(8) Å^3, Z = 12, T = 200(2) K, μ(Cu Kα) = 0.742 mm^{-1}, $Dcalc$ = 1.370 g/cm^3, 71984 reflections measured (6.14° ≤ 2Θ ≤ 147.24°), 11566 unique (R_{int} = 0.0364, R_{sigma} = 0.0280) which were used in all calculations. The final R_1 was 0.0438 (I > 2σ(I)) and wR_2 was 0.1281 (all data).

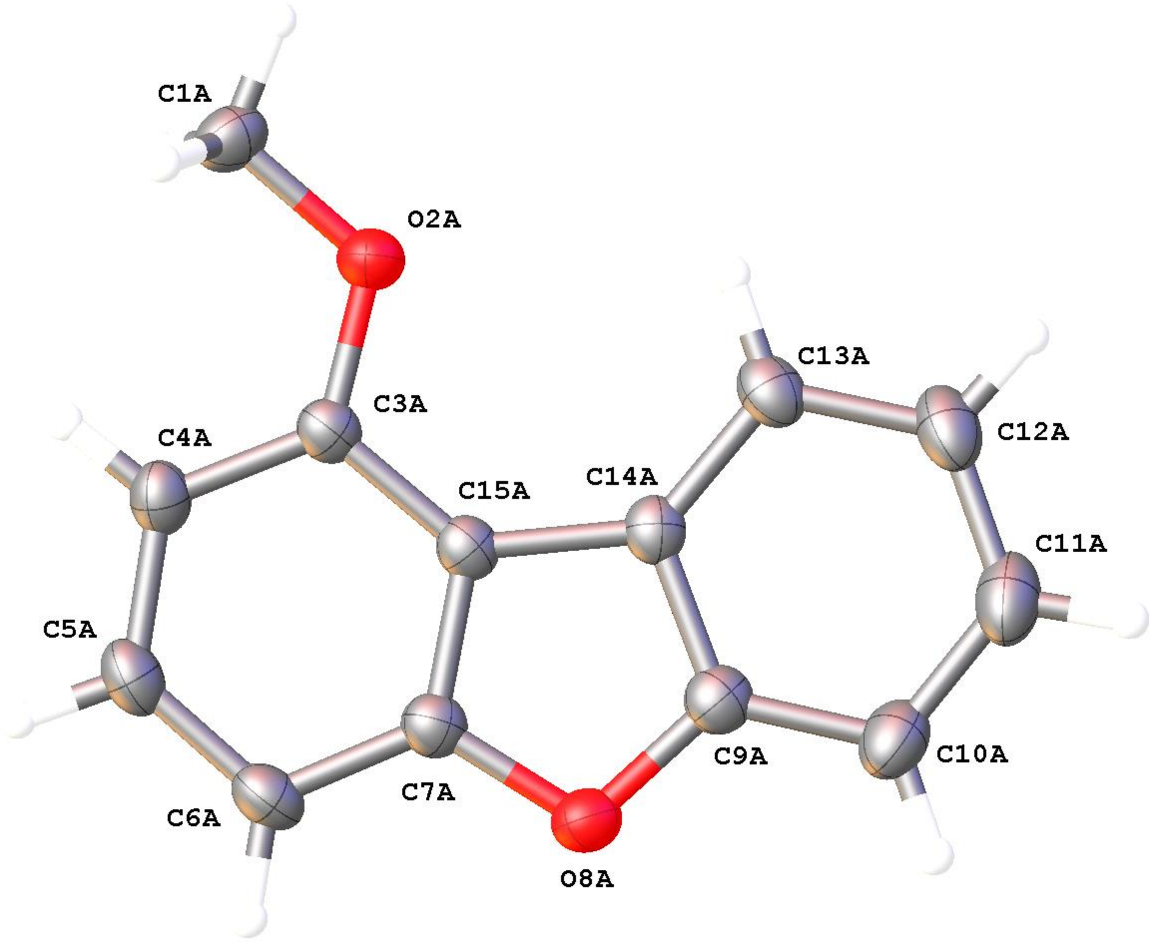

Supplement: Supplementary file 1 — jo2c02111_si_001.pdf [file jo2c02111_si_001.pdf]
